# Chemoselective Homologation–Deoxygenation Strategy Enabling the Direct Conversion of Carbonyls into (*n+1*)-Halomethyl-Alkanes

**DOI:** 10.1021/acs.orglett.0c02831

**Published:** 2020-09-10

**Authors:** Margherita Miele, Andrea Citarella, Thierry Langer, Ernst Urban, Martin Zehl, Wolfgang Holzer, Laura Ielo, Vittorio Pace

**Affiliations:** † Department of Pharmaceutical Chemistry, University of Vienna, Althanstrasse, 14, 1090 Vienna, Austria; ‡ Department of Chemical, Biological, Pharmaceutical and Environmental Sciences, University of Messina, Viale F. Stagno D’Alcontres, 31, 98166 Messina, Italy; § Faculty of Chemistry − Department of Analytical Chemistry, University of Vienna, Währinger Straße 38, 1090 Vienna, Austria; ∥ Department of Chemistry, University of Turin, Via P. Giuria 7, 10125 Turin, Italy

## Abstract

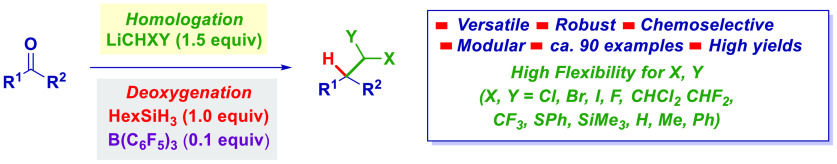

The sequential installation of a carbenoid and a hydride into a carbonyl, furnishing halomethyl alkyl derivatives, is reported. Despite the employment of carbenoids as nucleophiles in reactions with carbon-centered electrophiles, sp^3^-type alkyl halides remain elusive materials for selective one-carbon homologations. Our tactic levers on using carbonyls as starting materials and enables uniformly high yields and chemocontrol. The tactic is flexible and is not limited to carbenoids. Also, diverse carbanion-like species can act as nucleophiles, thus making it of general applicability.

Embodying a halogen-containing functionality within a carbon skeleton profoundly influences the physicochemical features, thus properly modulating the reactivity profile of the array.^1^ Accordingly, solid synthetic methodologies levered on different logics (e.g., radical, electrophilic, and nucleophilic) have been designed and thoroughly applied.^2^ In this sense, the introduction of metalated α-halogenated carbon species (MCR^1^R^2^Hal, i.e., the so-called carbenoid reagents) reacting under a nucleophilic or electrophilic regime (Scheme 1, path a), depending on the nature of the metal, has emerged as a valuable tool for delivering synthons featuring the exact degree of functionalization requested (i.e., halogen loading).^3^ As a result, common downsides associated with the use of conceptually different approaches, such as polyhalogenations, can be conveniently skipped. The initial installation of the CR^1^R^2^Hal unit, that is, a homologative event, is later exploited en route to the construction of more complex molecular architectures accessible through a single synthetic operation, as, for example, illustrated in the versatile Matteson homologation of sp^3^-hybridized boron electrophiles, elegantly adapted by Aggarwal to the assembly line concept.^4^ Regrettably, carbon-based platforms suitable for homologations with halocarbenoids are restricted to sp^2^-type systems: For example, our group demonstrated that homologations of carbonyl-type derivatives conduct, through a single operation, to more sophisticated architectures (quaternary aldehydes^5^ and aziridines).^6^ Also, olefins are amenable substrates for C1 insertions into cyclopropanes.^7^ In this scenario, the endeavored homologations of (primary) sp^3^-carbon platforms resulted in uncontrollable multi-insertion phenomena (up to four consecutive homologations) of questionable synthetic value, first noticed in the seminal works by Huisgen^8^ and later observed by Hahn^9^ (Scheme 1, path b). An initial solution to the polymethylene homologation problem is offered by the Knochel’s mixed copper–zinc mono-iodocarbenoids introduced in 1989, which, to the best of our knowledge, represent unique C1-halogenated units able to selectively control the process (Scheme 1, path c). Unfortunately, the attainable chemical space is narrowed by specific structural characteristics demanded of reactions partners, an allylic bromide as the recipient electrophile and an iodo-methyl-Cu-ZnI_2_ as the nucleophile, with the final result being the preparation of exclusively homoallylic iodides. This significant aspect is in contrast with the wide applications described for diverse halo methyl zinc carbenoids developed and thoroughly applied, for example, by Marek^11^ or different (non)-halomethyl Cu/Zn mixed carbenoids of Knochel.^10a−−10d^

**Scheme 1:**
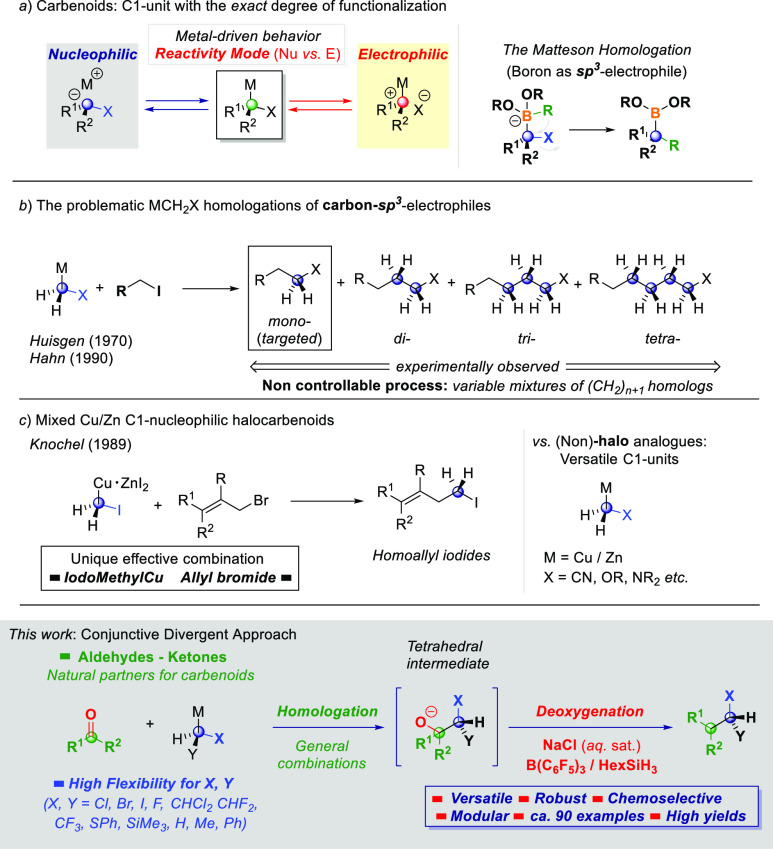
General Context of the Presented Work

We reasoned that realizing the carbenoid installation on a carbonyl sp^2^-carbon followed by the deoxygenation^12^ of the intermediate carbinol would represent a general and modular synthesis of homologous alkyl halides not dependent on the specific layout of reagents. Collectively, the strategy can be regarded as the employment of sp^2^-carbonyl systems as naked sp^3^-C-LG systems (LG = leaving group), which, after the envisaged sequence, would release the targeted motifs. We anticipate that this tactic will offer a robust and highly flexible solution for streamlining homologous (*n+*1)-haloalkyls that are tunable by selecting, at the operator’s discretion, both reaction partners: the electrophilic carbonyls and the nucleophilic carbenoids.

We selected benzaldehyde (**1**) as the model substrate for the homologative deoxygenation with LiCH_2_I to gain insights into both separate moments of the process (Table 1). In principle, installing an iodo-containing motif would be critical because, on one hand, it could trigger an internal nucleophilic displacement, giving an epoxide^5^ (**1b**, homologation side reduction), whereas, on the other hand, it could suffer from over-reduction to C–H (**1c**, deoxygenation side reduction).^13^ The optimized homologation step proceeded quantitatively within 0.5 h at −78 °C in THF using 1.4 equiv of LiCH_2_I, as deduced by ^1^H NMR and GC-MS analyses, thus yielding the tetrahedral intermediate **1a**. Leaving the reaction mixture for a longer time or increasing the temperature to −50 °C resulted in significant epoxidation. (For full details, see the SI.) Direct treatment under Barton–McCombie conditions^14^ gave iodoalkane **2** in low yield after a long time and at a high temperature (entry 1). We next applied the extremely versatile and convenient Oestreich’s formal reduction of alcohols,^15^ upon their conversion to tosylates, followed by B(C_6_F_5_)_3_-catalyzed dehydroxylation^16^ with Et_3_SiH and obtained a good 46% yield (entry 2). Further refinement was secured by simply quenching the homologation reaction crude product with water, thus making a formal iodohydrin that was directly suitable for deoxygenation after a trivial separation of the organic phases. Although the reduction took place in moderate yield (52%), we hypothesized that the THF (used for the homologation) still present in the reaction mixture, upon dilution with DCM, could interfere with the C–O breaking event (entry 3). Indeed, the prior complete removal of THF (washing of the homologation crude product with sat. NaCl (aq)) benefited the dehydroxylation, giving a 66% yield (entry 4). Less hindered silanes such as Ph_2_SiH_2_, Et_2_SiH_2_, PhSiH_3_, and hexSiH_3_ were also effective: Excellent selectivity (i.e., no side reduction was noticed) was observed, suggesting the latter as the ideal agent (entries 5–8). Replacing B(C_6_F_5_)_3_ with a different Lewis acid such as InCl_3_^17^ had a negative effect on the process (entry 9).

**Table 1:**
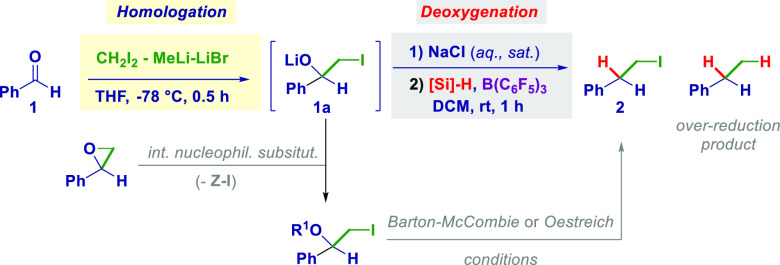
Model Reaction: Optimization^a^

entry	LiCH_2_I (equiv)/time (h)	deoxygenation reductant/solvent	Lewis acid	yield of **2** (%)^a^
1^b^	1.4/0.5	*BMC*		11
2^c^	1.4/0.5	*Oestreich*	B(C_6_F_5_)_3_	46
3^d^	1.4/0.5	Et_3_SiH/DCM	B(C_6_F_5_)_3_	52
4^e^	1.4/0.5	Et_3_SiH/DCM	B(C_6_F_5_)_3_	66
5	1.4/0.5	Ph_2_SiH_2_/DCM	B(C_6_F_5_)_3_	68
6	1.4/0.5	Et_2_SiH_2_/DCM	B(C_6_F_5_)_3_	77
7	1.4/0.5	PhSiH_3_/DCM	B(C_6_F_5_)_3_	84
8	*1.4/0.5*	*hexSiH*_*3*_*/DCM*	*B(C*_*6*_*F*_*5*_*)*_*3*_	89
9	1.4/0.5	hexSiH_3_/DCM	InCl_3_	60

a Isolated yield after the homologation/deoxygenation sequence.

b BMC, Barton–McCombie (R^1^ = PhCS, Bu_3_SnH, AIBN, toluene, reflux).

c Oestreich (R^1^ = Ts, Et_3_SiH, B(C_6_F_5_)_3_, DCM).

d Upon quenching with H_2_O, DCM was added, and the two phases were separated.

e Sat. NaCl (aq) and DCM were added prior to phase separation. Unless otherwise stated, B(C_6_F_5_)_3_ (0.1 equiv) was used.

Once the reaction conditions were set, we studied the scope of the sequential process (Scheme 2). The chemocontrol was superb, as illustrated in the case of sensitive substrates such as a cyclic enone (**3**) and an α,β-unsaturated ester (**4**): No over-reduction of the olefinic and ester carbonyl motifs was noticed. The protocol was highly flexible, as deduced when using a different carbenoid homologating agent. The chloromethylation–deoxygenation methodology was effective in the case of benzaldehyde derivatives decorated with several functionalities of diverse electronic behavior, including alkyl (**5**), amino (**9**), and polyaromatics (**10**), among others. Notably, the acetal-containing bromo derivative (**11**) did not interfere in either the homologation or the reduction steps. Positioning differently constituted halogen substituents is permitted (**6**–**8**, **12**–**14**), as is increasing the sterical hindrance close to the carbonyl (e.g., 2,6-disubstituted systems, **15** and **16**). Aliphatic aldehydes could be subjected to the reaction conditions, giving ω-chloro phenylalkanes (**17** and **18**) in high yields. Remarkably, a propargylic aldehyde smoothly gave the homologated analogue (**19**), preserving the chemical integrity of the alkyne. The protocol could be extended to ketones as starting substrates. Aliphatic derivatives reacted well, giving α-chloro tertiary centers in the case of both cyclic (**20**) and acyclic (**21**) derivatives. Analogously, indanone and tetralone derivatives (**22** and **23**) underwent the transformation; remarkably, scaling up to 15 mmol validated the method (**22**, 87% yield). During the reduction step, concomitant bis-demethoxylation was observed, thus affording the interesting biologically relevant dihydroxyphenyl (catechol-like) scaffold **23**.

**Scheme 2:**
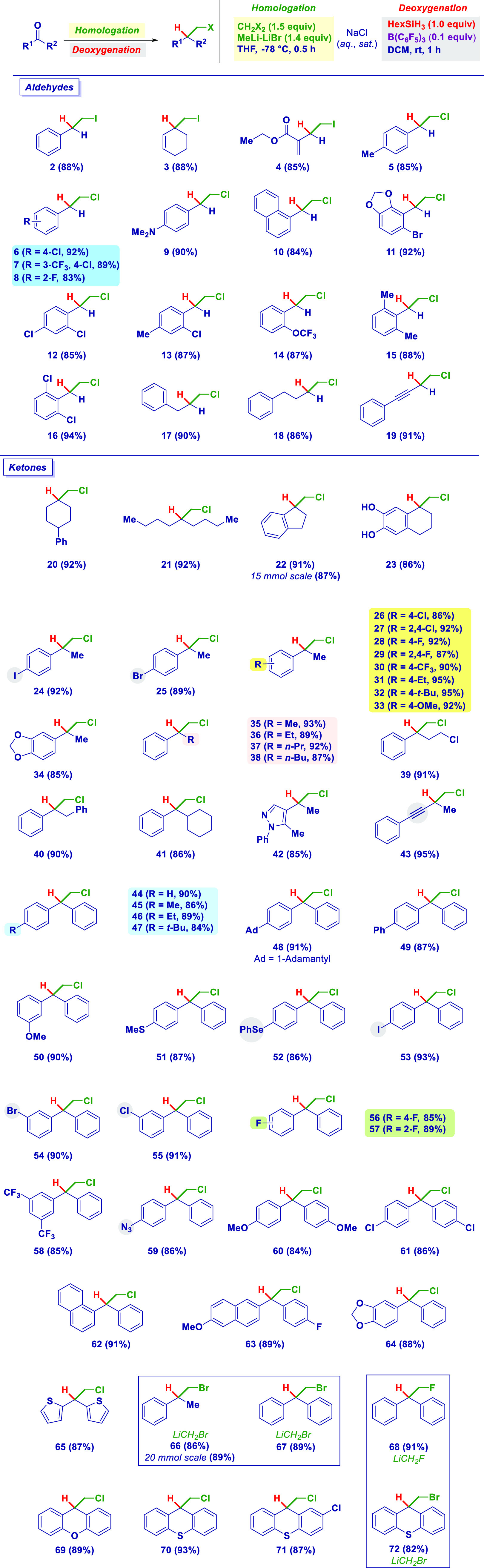
Scope of the Sequential LiCH_2_X Homologation/Deoxygenation

Acetophenone derivatives were excellent materials, further documenting the high degree of chemocontrol associated with the reductive homologation. The presence of sensitive groups is fully tolerated, as illustrated by sensitive halogen iodo (**24**), bromo (**25**), chloro (**26** and **27**), fluoro (**28** and **29**), and trifluoromethyl substituents (**30**). Substituents on the aromatic ring of the opposite electronic effect maintain an unaltered efficiency: ethyl (**31**), *tert*-butyl (**32**), methoxy (**33**), and acetal (**34**). The progressive enlargement of the aliphatic terminus of the acetophenone core (**35**–**38**) was not detrimental. The genuine homologative conditions were further deduced by the precise nucleophilic attack, reduction on the carbonyl of ω-chloro-propiophenone, without noticing any collateral effect (e.g., side homologation) on the constitutive CH_2_Cl appendix (**39**). Analogously, chloromethyl derivatives of 1,2-diphenylethane (**40**), cyclohexyl-toluene (**41**), and alkylpyrazol (**42**) could also be synthesized in high yield with high selectivity. Again, a propargyl fragment did not touch its integrity under the reaction conditions, giving **43**. Diaryl ketones proved to be highly effective substrates for the transformation, as indicated by a series of (mono)-substituted alkyls (**44**–**47**), including an adamantyl derivative (**48**) and aryl (**49**) benzophenone functionalities. Alkoxy (**50**), alkylthio (**51**), and arylseleno (**52**) groups could be opportunely incorporated on the benzophenone core, highlighting the fact that no simultaneous Se–Li exchange occurred during the carbenoid genesis. As a further confirmation of the chemoselectivity, potentially exchangeable halogens, such as iodine (**53**), bromine (**54**), chloro (**55**), and fluoro (**56** and **57**), or modifications thereof (trifluoromethyl (**58**)) were unambiguously endured. It is noteworthy that an azido substituent did not undergo a concomitant reduction and was intact at the end of the transformation (**59**), thus remarking on the chemoselectivity profile. Disubstituted symmetric (**60** and **61**) and asymmetric (**62** and **63**) benzophenones could react in high yields regardless of the electronic orientation of the substituents, including cases of heteroaromatic systems such as benzofuran (**64**) and dithienyl (**65**). The versatility of the method was also gathered by modifying the nature of nucleophilic carbenoids: When LiCH_2_Br^4g^ was conducted to the bromomethyl analogues (**66** and **67**), also on a higher scale (20 mmol, **66**), while using the highly unstable LiCH_2_F,^18^ an efficient synthesis of the fluoro derivative (**68**) could be performed. Notably, tricyclic-type ketones of xanthene (**69**) and thioxanthene (**70**–**72**) types also reacted under similar chloro- or bromo-methylation/deoxygenation conditions.

The successful outcome inferred by reacting monohalocarbenoids as the first nucleophiles spurred us to widen the method to dihalomethyl analogues, notoriously challenging entities for which unified, general, and reliable strategies are still underdeveloped.^19^ Benefiting from the tunable intrinsic versatility of carbenoid precursors, the simple switching from a halogen–lithium exchange (shown above) to a hydrogen–lithium exchange (i.e., deprotonation with lithium tetramethylpiperidide (LTMP)) resulted in the formation of diverse dihalomethyl fragments that expeditiously reacted with ketones and aldehydes prior to deoxygenation, thus giving dibromo (**73** and **74**, further suitable for scaling in the case of the former) and dichloro (**75** and **76**) derivatives. When a halo-halo′-methane (XCH_2_Y) was selected as the pro-carbenoid, the treatment with the same LTMP afforded the corresponding mixed carbenoids (LiCHXY)^20^ deliverable to carbonyls with comparable efficiency and chemoselectivity: After the deoxygenation, chlorobromo (**77** and **78**), chloroiodo (**79**), and bromoiodo (**80**) analogues were prepared in high yield with high control (Scheme 3). As an additional proof of the modularity of the concept, we were pleased to prepare difluoromethyl (**81**) and trifluoromethyl (**82**) derivatives. The well-known reluctance of using polyfluoromethyllithiums^21^ was circumvented with silylated suitable precursors (TMSCHF_2_^22^ and TMSCF_3_^23^), which, upon adequate activation, furnished the corresponding formal carbanions.

**Scheme 3:**
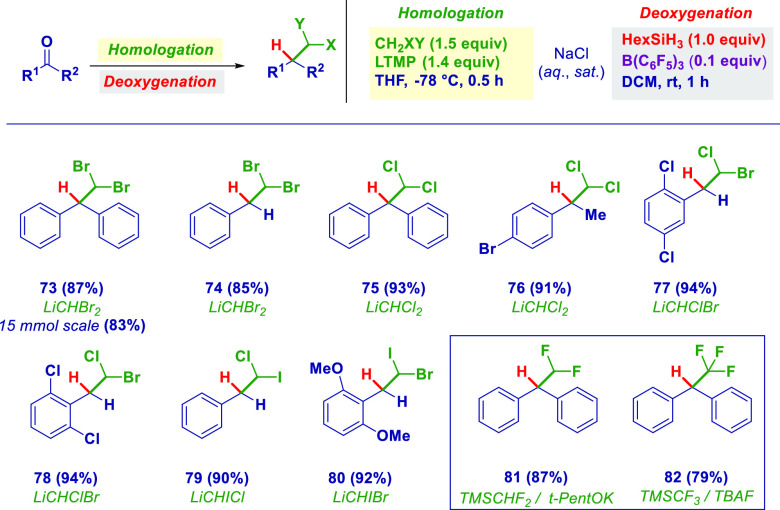
Dihalomethyl Homologation/Deoxygenation Sequence

This conceptually intuitive carbonyl nucleophilic addition–deoxygenation sequence represents a formidable tool for forging C–C bonds, as documented by the perfect extensibility to nonhalogenated carbanions (Scheme 4). Hence, by adding an α-silyl methyl carbanion (TMSCH_2_Li), terminal silanes were produced from both an aldehyde (**83**) and a ketone (**84**), whereas terminal thioethers were prepared through the reaction of carbonyls with an α-thio methyllithium reagent (**85**–**87**).^24^ More generally, two unfunctionalized organolithiums, MeLi and PhLi (selected as model representatives for alkyl and aryl species), were amenable to reaching the corresponding trisubstituted methanes (**88** and **89**).

**Scheme 4:**
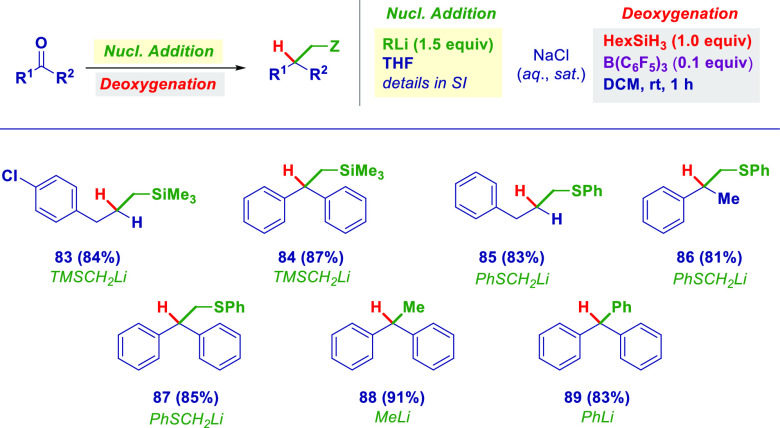
General Nucleophilic Addition/Deoxygenation Protocol with Various Carbanion-like Reagents

In summary, we have documented the high-yielding addition of two nucleophiles, a halo-carbenoid and a hydride, to the carbonyl carbon of aldehydes and ketones, thus increasing their (already) high potential and versatility in synthesis.^25^ The overall operation consisting of two distinct processes, namely, homologation and silane-mediated deoxygenation under B(C_6_F_5_)_2_ catalysis, enables access to a plethora of halomethyl–alkyl derivatives. The conditions established for both phases of the sequence feature very high chemocontrol, thus guaranteeing safe and reliable transformations in the presence of several sensitive functionalities, such as halogens, olefins, alkynes, esters, and so on. The robustness of the logic proposed, assessed across ca. 90 presented cases, entails adding not only a wide range of monohalo- and dihalomethyl carbenoids but also fluorinated, silylated, mercapto, and, more generally, simple alkyl and aryl organolithiums.

## Supporting Information Available

Experimental procedures, NMR spectra, and analytical data for all of the compounds (PDF)
