# Supplementary material for: Chemoselective Homologation–Deoxygenation Strategy Enabling the Direct Conversion of Carbonyls into (n+1)-Halomethyl-Alkanes
Source: Org Lett. 2020 Sep 10;22(19):7629–34. doi: 10.1021/acs.orglett.0c02831 (PMC8011987; doi:10.1021/acs.orglett.0c02831)

Supporting Information

Chemoselective Homologation-Deoxygenation Strategy Enabling the Direct Conversion of Carbonyls into (*n*+1)-Halomethyl-Alkanes

Margherita Miele,[a] † Andrea Citarella,[a, b] † Thierry Langer,[a] Ernst Urban,[a] Martin Zehl,[c] Wolfgang Holzer,[a] Laura Ielo[a] and Vittorio Pace*[a, d]

[a] University of Vienna - Department of Pharmaceutical Chemistry - Althanstrasse, 14 - 1090, Vienna, Austria. Tel. +43-1-4277-55035. http://drugsynthesis.univie.ac.at/ - e-mail: vittorio.pace@univie.ac.at

[b] University of Messina - Department of Chemical, Biological, Pharmaceutical and Environmental Sciences – Viale F. Stagno D'Alcontres, 31 - 98166 Messina, Italy.

[c] University of Vienna – Faculty of Chemistry - Department of Analytical Chemistry - Währinger Straße 38 - 1090, Vienna, Austria.

[d] University of Turin - Department of Chemistry – Via P. Giuria, 7 - 10125, Turin, Italy. e-mail: vittorio.pace@unito.it

† *Contributed equally*

Table of contents

1. Materials and methods. 3
2. General procedures. 4
3. Additional optimization studies – Table S1. 6
4. Spectral and Characterization Data. 7
5. ^{1}H-, ^{13}C- and ^{19}F-NMR Spectra. 48

1. Materials and methods

Melting Points were determined on a Reichert-Kofler hot-stage microscope and are uncorrected. Mass spectra were obtained on a Shimadzu QP 1000 instrument (EI, 70 eV) and on a Bruker maXis 4G instrument (ESI-TOF, HRMS). ^{1}H, ^{13}C and ^{19}F NMR spectra were recorded at 297 K on a Bruker Avance III 400 spectrometer (400 MHz for ^{1}H, 100 MHz for ^{13}C, 40 MHz for ^{15}N, 376 MHz for ^{19}F) equipped with a directly detecting broadband observe (BBFO) probe, with a Bruker Avance III 500 spectrometer (500 MHz for ^{1}H, 125 MHz for ^{13}C) using a Prodigy cryoprobe, and with a Bruker DRX 200 spectrometer (200 MHz for ^{1}H, 50 MHz for ^{13}C) with a $^{1}H/^{13}C$ dual probe.
The centre of the solvent signal was used as an internal standard which was related to TMS with δ 7.26 ppm (^{1}H in $CDCl_3$), δ 7.16 ppm (^{1}H in C_6D_6), δ 77.00 ppm (^{13}C in $CDCl_3$) and δ 128.06 ppm (^{13}C in C_6D_6). Absolute referencing via *Ξ* ratio was usd for the ^{19}F NMR spectra. Spin-spin coupling constants (*J*) are given in Hz.
In nearly all cases, full and unambiguous assignment of all resonances was performed by combined application of standard NMR techniques, such as APT, HSQC, HMBC, HSQC-TOCSY, COSY and NOESY experiments.
All the reactions were carried out under inert atmosphere of argon. THF was distilled over Na/benzophenone. Chemicals were purchased from Sigma-Aldrich, Acros, Alfa Aesar, Fluorochem and TCI Europe. Solutions were evaporated under reduced pressure with a rotary evaporator.
TLC was carried out on aluminium sheets precoated with silica gel 60F254 (Merchery-Nagel, Merk); the spots were visualised under UV light (λ = 254 nm).

2. General procedures

General Procedure 1

To a solution of carbonyl compound (aldehyde or ketone, 1 equiv) in dry THF (3 mL) cooled at -78 °C, the dihalomethane carbenoids precursor was added (1.5 equiv) under Argon atmosphere. After 10 min, MeLi-LiBr 2.2 M solution in Et_2O (1.4 equiv) was added *via* syringe pump (0.20 mL/min) during a period of 15 min and, then the stirring was continued for additional 0.5 h. Subsequently, a saturated (*aq.*) NaCl was added to the mixture and the cooling bath was removed; the organic phase was extracted with dichloromethane (3 x 3 mL) and, dried over anhydrous Na_2SO_4. The filtered solution was flushed under argon and tris(pentafluorophenyl)borane (0.1 equiv) was incorporated to it at room temperature. After 2 min, hexylsilane (1 equiv) was added in one pot and, the reaction was stirred for 1 h. Finally, the mixture was quenched with saturated (*aq.*) NH_4Cl (3 mL) and extracted with dichloromethane (3 mL). The organic layer was washed with saturated (*aq.*) NaCl (5 mL), dried over anhydrous Na_2SO_4, filtered and concentrated under reduced pressure (bath: rt) to give the crude compound eventually purified as indicated below.

General Procedure 2

Preparation of LTMP. Freshly distilled 2,2,6,6-tetramethylpiperidine (TMP) was added to THF (3 mL) and the resulting mixture was cooled at 0 °C. Then, MeLi-LiBr (2.2 M solution in Et_2O) was added dropwise over 10 min. The so obtained solution was transferred *via* cannula to the solution indicated below.

Homologation / Deoxygenation sequence. To a solution of carbonyl compound (aldehyde or ketone, 1 equiv) in dry THF (3 mL) cooled at -78 °C, the dihalomethane carbenoid precursor was added (1.5 equiv) under Argon atmosphere. After 10 min, the above prepared LTMP solution (1.4 equiv) was added *via* syringe pump (0.20 mL/min) and, then the stirring was continued for additional 0.5 h. Subsequently, a saturated (*aq.*) NaCl was added to the mixture and the cooling bath was removed; the organic phase was extracted with dichloromethane (3 x 3 mL) and, dried over anhydrous Na_2SO_4. The filtered solution was flushed under argon and tris(pentafluorophenyl)borane (0.1 equiv) was incorporated to it at room temperature. After 2 min, hexylsilane (1 equiv) was added in one pot and, the reaction was stirred for 1 h. Finally, the mixture was quenched with saturated (*aq.*) NH_4Cl (3 mL) and extracted with dichloromethane (3 mL). The organic layer was washed with saturated (*aq.*) NaCl (5 mL), dried over anhydrous Na_2SO_4, filtered and concentrated under reduced pressure (bath: rt) to give the crude compound eventually purified as indicated below.

General Procedure 3

To a solution of carbonyl compound (1 equiv) in dry THF (3 mL) cooled at 0 °C, difluoromethyltrimethylsilane (1.5 equiv) was added under Argon atmosphere. Then, potassium *tert*-pentoxide 0.9 M in toluene (1.4 equiv) was added *via* syringe pump (0.20 mL/min) at 0 °C during a period of 15 min. The reaction mixture was further stirred to reach rt within 4 h. Subsequently, a saturated (*aq.*) NaCl was added to the mixture and the cooling bath was removed; the organic phase was extracted with dichloromethane (3 x 3 mL) and, dried over anhydrous Na_2SO_4. The filtered solution was flushed under argon and tris(pentafluorophenyl)borane (0.1 equiv) was incorporated to it at room temperature. After 2 min, hexylsilane (1 equiv) was added in one pot and, the reaction was stirred for 1 h. Finally, the mixture was quenched with saturated (*aq.*) NH_4Cl (3 mL) and extracted with dichloromethane (3 mL). The organic layer was washed with saturated (*aq.*) NaCl (5 mL), dried over anhydrous Na_2SO_4, filtered and concentrated under reduced pressure (bath: rt) to give the crude compound eventually purified as indicated below.

General Procedure 4

To a solution of carbonyl compound (1 equiv) in dry THF (3 mL) cooled at 0 °C, trifluoromethyltrimethylsilane (1.5 equiv) was added under Argon atmosphere. Then, tetrabutylammonium fluoride (TBAF) solution 1.0 M in THF (1.4 equiv) was added *via* syringe pump (0.20 mL/min) at 0 °C during a period of 15 min. The reaction mixture was further stirred to reach rt within 6 h. Subsequently, a saturated (*aq.*) NaCl was added to the mixture and the cooling bath was removed; the organic phase was extracted with dichloromethane (3 x 3 mL) and, dried over anhydrous Na_2SO_4. The filtered solution was flushed under argon and tris(pentafluorophenyl)borane (0.1 equiv) was incorporated to it at room temperature. After 2 min, hexylsilane (1 equiv) was added in one pot and, the reaction was stirred for 1 h. Finally, the mixture was quenched with saturated (*aq.*) NH_4Cl (3 mL) and extracted with dichloromethane (3 mL). The organic layer was washed with saturated (*aq.*) NaCl (5 mL), dried over anhydrous Na_2SO_4, filtered and concentrated under reduced pressure (bath: rt) to give the crude compound eventually purified as indicated below.

General Procedure 5

To a solution of carbonyl compound (1 equiv) in dry THF (3 mL) cooled at -50 °C, the competent organolithium reagent (1.5 equiv) was added under Argon atmosphere *via* syringe pump (0.20 mL/min). The reaction mixture was further stirred to reach 0 °C within 2 h. Subsequently, a saturated (*aq.*) NaCl was added to the mixture and the cooling bath was removed; the organic phase was extracted with dichloromethane (3 x 3 mL) and, dried over anhydrous Na_2SO_4. The filtered solution was flushed under argon and tris(pentafluorophenyl)borane (0.1 equiv) was incorporated to it at room temperature. After 2 min, hexylsilane (1 equiv) was added in one pot and, the reaction was stirred for 1 h. Finally, the mixture was quenched with saturated (*aq.*) NH_4Cl (3 mL) and extracted with dichloromethane (3 mL). The organic layer was washed with saturated (*aq.*) NaCl (5 mL), dried over anhydrous Na_2SO_4, filtered and concentrated under reduced pressure (bath: rt) to give the crude compound eventually purified as indicated below.

General Procedure 6

Preparation of $LiCH_2SPh$. To a solution of thioanisole (1.5 equiv) in dry THF (3 mL) cooled at 0 °C, under Argon atmosphere, 1,4-diazabicyclo[2.2.2]octane (DABCO, 1.5 equiv) was added. Then, *n*-butyllitium 2.5 M in *n*-hexane (1.4 equiv) was added dropwise for 1.5 h, before transferring *via* cannula to the solution indicated below containing the carbonyl compound.

Addition to the carbonyl compound / deoxygenation sequence. To a solution of carbonyl compound (1 equiv) in dry THF (3 mL) cooled at 0 °C, the THF solution of $LiCH_2SPh$ prepared above (1.5 equiv) was added under Argon atmosphere was added *via* syringe pump (0.20 mL/min). The reaction mixture was further stirred for further 3 h at this same temperature. Subsequently, a saturated (*aq.*) NaCl was added to the mixture and the cooling bath was removed; the organic phase was extracted with dichloromethane (3 x 3 mL) and, dried over anhydrous Na_2SO_4. The filtered solution was flushed under argon and tris(pentafluorophenyl)borane (0.1 equiv) was incorporated to it at room temperature. After 2 min, hexylsilane (1 equiv) was added in one pot and, the reaction was stirred for 1 h. Finally, the mixture was quenched with saturated (*aq.*) NH_4Cl (3 mL) and extracted with dichloromethane (3 mL). The organic layer was washed with saturated (*aq.*) NaCl (5 mL), dried over anhydrous Na_2SO_4, filtered and concentrated under reduced pressure (bath: rt) to give the crude compound eventually purified as indicated below.

Table S1. Additional Optimization Studies

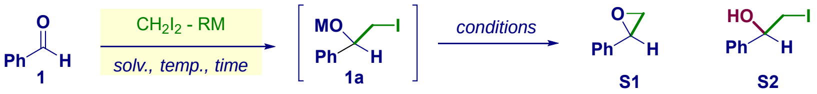

Entry	CH_2I_2 (equiv)	Metalating agent RM (equiv)	Solvent / Temp. (°C) / Time [h]	Conversion (%)[a]	Ratio **1: S1:S2**
1	1.3	MeLi-LiBr (1.2)	THF, -78 °C, 0.5	85%	0.2 : 0 : 0.8
2	1.1	MeLi-LiBr (1.0)	THF, -78 °C, 0.5	73%	0.3 : 0 : 0.7
3	2.5	MeLi-LiBr (2.4)	THF, -78 °C, 0.5	>99%	0 : 0 : 1
4	1.5	MeLi-LiBr (1.4)	THF, -78 °C, 0.5	92%	0.07 : 0 : 0.93
5	1.5	MeLi-LiBr (1.4)[b]	THF, -78 °C, 0.5	>98%	0 : 0 : 1
6	1.5	MeLi-LiBr (1.4)[b]	THF, -50 °C, 0.5	>98%	0 : 0.3 : 0.7
7	1.5	MeLi-LiBr (1.4)[b]	THF, -78 °C, 1	>98%	0 : 0.2 : 0.8
8	1.5	MeLi-LiBr (1.4)[b]	Et_2O, -78 °C, 0.5	>98%	0.1 : 0.2 : 0.7
9	1.5	MeLi-LiBr (1.4)[b]	Tol, -78 °C, 0.5	86%	0.15 : 0 : 0.85
11	1.5	*i*-PrMgBr (1.4)[b]	THF, -78 °C, 0.5	83%	0.18 : 0 : 0.82
12	1.5	*i*-PrMgCl (1.4)[b]	THF, -78 °C, 0.5	87%	0.12 : 0 : 0.88
13	1.5	*i*-PrMgCl-LiCl (1.4)[b]	THF, -78 °C, 0.5	91%	0.1 : 0 : 0.9
14	1.5	*i*-PrMgCl-LiCl (1.4)[b,c]	THF, -78 °C, 0.5	93%	0.1 : 0 : 0.9
15	1.5	*n*-BuLi (1.4)[b]	THF, -78 °C, 0.5	90%	0.1 : 0 : 0.9

[a] Determined *via* GC-MS analysis. [b] The metalating agent (MeLi-LiBr, *i*-PrMgBr, *i*-PrMgCl, *i*-PrMgCl-LiCl, *n*-BuLi) was added *via* syringe pump (0.2 mL/min rate). [c] Benzaldehyde was added 5 min after the addition of *i*-PrMgCl-LiCl was completed (*i.e.* non Barbier-type conditions). Otherless stated the following metalating agents were employed: MeLi-LiBr (2.2 M in Et_2O), *i*-PrMgBr (0.75 M in THF), *i*-PrMgCl (2.0 M in THF), *i*-PrMgCl-LiCl (1.3 M in THF), *n*-BuLi (1.6 M in hexanes). Prior to GC-MS injection, reactions crudes – quenched with NH_4Cl (*aq.*) – were extracted with Et_2O.

4. Spectral and Characterization Data

(2-Iodoethyl)benzene (2)

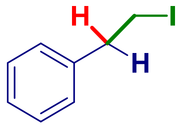

By following the **General procedure 1**, starting from benzaldehyde (200 mg, 1.88 mmol, 1 equiv) in dry THF (3 mL), diiodomethane (0.23 mL, 2.8 mmol, 1.5 equiv), MeLi-LiBr 2.2 M solution in Et_2O (1.2 mL, 2.6 mmol, 1.4 equiv), tris(pentafluorophenyl)borane (96 mg, 0.2 mmol, 0.1 equiv) and hexylsilane (0.3 mL, 1.88 mmol, 1 equiv), **compound 2** was obtained in 88% yield (384 mg) as colorless oil after column chromatography on silica gel (*n*-hexane as eluent).

^{1}H NMR (400 MHz, $CDCl_3$) δ: 7.33 (m, 2H, Ph H-3,5), 7.28 (m, 1H, Ph H-4), 7.20 (m, 2H, Ph H-2,6), 3.56 (m, 2H, CH_2I), 3.19 (m, 2H, CH_2).

^{13}C NMR (100 MHz, $CDCl_3$) δ: 140.6 (Ph C-1), 128.6 (Ph C-3,5), 128.3 (Ph C-2,6), 126.9 (Ph C-4), 40.4 (CH_2), 5.5 (CH_2I).

EI-MS m/z (%): 232.0 (M^+, 4), 105.1 (M^+, 100), 77.1 (M^+, 18), 51.2 (M^+, 9) .

3-(Iodomethyl)cyclohexene (3)

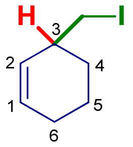

By following the **General procedure 1**, starting from cyclohex-2-enone (200 mg, 2.08 mmol, 1 equiv) in dry THF (3 mL), diiodomethane (0.3 mL, 3.12 mmol, 1.5 equiv), MeLi-LiBr 2.2 M solution in Et_2O (1.32 mL, 2.9 mmol, 1.4 equiv), tris(pentafluorophenyl)borane (107 mg, 0.2 mmol, 0.1 equiv) and hexylsilane (0.3 mL, 2.08 mmol, 1 equiv), **compound 3** was obtained in 88% yield (407 mg) as colorless oil after column chromatography on silica gel (*n*-hexane as eluent).

^{1}H NMR (400 MHz, $CDCl_3$) δ: 5.79 (m, 1H, Cyclohexene H-1), 5.56 (m, 1H, Cyclohexene H-2), 3.17 (dd, J = 9.6, 5.8 Hz, 1H, CH_2I), 3.12 (dd, J = 9.6, 7.3 Hz, 1H, CH_2I), 2.35 (m, 1H, Cyclohexene H-3), 1.96 (m, 2H, Cyclohexene H-6), 1.87 (m, 1H, Cyclohexene H-4), 1.71 (m, 1H, Cyclohexene H-5), 1.56 (m, 1H, Cyclohexene H-5), 1.39 (m, 1H, Cyclohexene H-4).

^{13}C NMR (100 MHz, $CDCl_3$) δ: 129.6 (Cyclohexene C-2), 129.4 (Cyclohexene C-1), 37.4 (Cyclohexene C-3), 29.5 (Cyclohexene C-4), 25.2 (Cyclohexene C-6), 20.8 (Cyclohexene C-5), 14.3 (CH_2I).

Ethyl 4-Iodo-2-methylidenebutanoate (4)

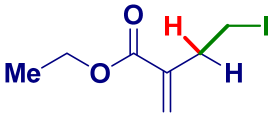

By following the **General procedure 1**, starting from ethyl 2-formylacrylate (200 mg, 1.56 mmol, 1 equiv) in dry THF (3 mL), diiodomethane (0.2 mL, 2.34 mmol, 1.5 equiv), MeLi-LiBr 2.2 M solution in Et_2O (1.0 mL, 2.2 mmol, 1.4 equiv), tris(pentafluorophenyl)borane (80 mg, 0.2 mmol, 0.1 equiv) and hexylsilane (0.3 mL, 1.56 mmol, 1 equiv), **compound 4** was obtained in 85% yield (337 mg) as colorless oil after column chromatography on silica gel (*n*-hexane/diethyl ether 95:5 as eluent).

^{1}H NMR (400 MHz, $CDCl_3$) δ: 6.30 (d, *J* = 1.2 Hz, 1H, C=CH_2, *cis* to ester group), 5.65 (q, *J* = 1.2 Hz, 1H, C=CH_2, *trans* to ester group), 4.22 (q, *J* = 7.1 Hz, 2H, $OC\underline{H}_2CH_3$), 3.32 (t, *J* = 7.2 Hz, 2H, $CH_2C\underline{H}_2I$), 2.86 (dt, J_t = 7.2 Hz, J_d = 1.2 Hz, 2H, $C\underline{H}_2CH_2I$), 1.31 (t, *J* = 7.1 Hz, 3H, $OCH_2C\underline{H}_3$).

^{13}C NMR (100 MHz, $CDCl_3$) δ: 166.2 (C=O), 138.8 ($\underline{C}$=CH_2), 127.2 (C=$\underline{C}H_2$), 60.9 ($O\underline{C}H_2CH_3$), 36.4 ($\underline{C}H_2CH_2I$), 14.2 ($OCH_2\underline{C}H_3$), 3.9 ($CH_2\underline{C}H_2I$).

1-(2-Chloroethyl)-4-methylbenzene (5)

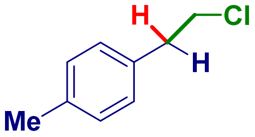

By following the **General procedure 1**, starting from 4-methylbenzaldehyde (200 mg, 1.66 mmol, 1 equiv) in dry THF (3 mL), chloroiodomethane (0.2 mL, 2.49 mmol, 1.5 equiv) , MeLi-LiBr 2.2 M solution in Et_2O (1.0 mL, 2.2 mmol, 1.4 equiv), tris(pentafluorophenyl)borane (85 mg, 0.17 mmol, 0.1 equiv) and hexylsilane (0.3 mL, 1.66 mmol, 1 equiv), **compound 5** was obtained in 85 % yield (218 mg) as colorless oil after column chromatography on silica gel (*n*-hexane as eluent).

^{1}H NMR (500 MHz, $CDCl_3$) δ: 7.14 (m, 2H, Ph H-3,5), 7.12 (m, 2H, Ph H-2,6), 3.70 (t, 2H, $^3J_{H,H}$ = 7.5 Hz, CH_2Cl), 3.04 (t, 2H, $^3J_{H,H}$ = 7.5 Hz, Ph-CH_2), 2.34 (s, 3H, CH_3).

^{13}C NMR (125 MHz, $CDCl_3$) δ: 136.5 (Ph C-4), 135.0 (Ph C-1), 129.3 (Ph C-3,5), 128.7 (Ph C-2,6), 45.2 (CH_2Cl), 38.8 (Ph-CH_2), 21.0 (s, 3H, CH_3).

1-Chloro-4-(2-chloroethyl)benzene (6)

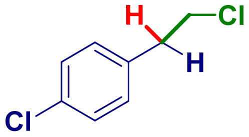

By following the **General procedure 1**, starting from 4-chlorobenzaldehyde (200 mg, 1.42 mmol, 1 equiv) in dry THF (3 mL), chloroiodomethane (0.16 mL, 2.13 mmol, 1.5 equiv) , MeLi-LiBr 2.2 M solution in Et_2O (0.9 mL, 1.99 mmol, 1.4 equiv), tris(pentafluorophenyl)borane (73 mg, 0.14 mmol, 0.1 equiv) and hexylsilane (0.23 mL, 1.41 mmol, 1 equiv), **compound 6** was obtained in 92% yield (229 mg) as colorless oil after column chromatography on silica gel (*n*-hexane as eluent).

^{1}**H NMR** (500 MHz, $CDCl_3$) δ: 7.29 (m, 2H, Ph H-2,6), 7.16 (m, 2H, Ph H-3,5), 3.69 (t, 2H, $^{3}J_{H,H}$ = 7.2 Hz, CH_2Cl), 3.04 (t, 2H, $^{3}J_{H,H}$ = 7.2 Hz, Ph-CH_2).

^{13}**C NMR** (125 MHz, $CDCl_3$) δ: 136.5 (Ph C-4), 132.7 (Ph C-1), 130.2 (Ph C-3,5), 128.7 (Ph C-2,6), 44.7 (CH_2Cl), 38.3 (Ph-CH_2).

EI-MS m/z (%): 174.0 (M^+, 17), 125.1 (M^+, 100), 77.1 (M^+, 10), 51.1 (M^+, 11).

1-Chloro-4-(2-chloroethyl)-2-(trifluoromethyl)benzene (7)

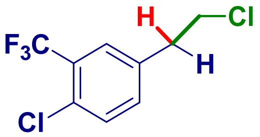

By following the **General procedure 1**, starting from 4-chloro-3-(trifluoromethyl)benzaldehyde (200 mg, 0.96 mmol, 1 equiv) in dry THF (3 mL), chloroiodomethane (0.11 mL, 1.44 mmol, 1.5 equiv), MeLi-LiBr 2.2 M solution in Et_2O (0.61 mL, 1.34 mmol, 1.4 equiv), tris(pentafluorophenyl)borane (49 mg, 0.1 mmol, 0.1 equiv) and hexylsilane (0.16 mL, 0.96 mmol, 1 equiv), compound **7** was obtained in 89% yield (208 mg) as colorless oil after column chromatography on silica gel (*n*-hexane as eluent).

^{1}**H NMR** (500 MHz, C_6D_6) δ: 7.06 (d, 1H, $^{4}J_{H,H}$ = 2.0 Hz, Ph H-3), 6.86 (d, 1H, $^{3}J_{H,H}$ = 8.2 Hz, Ph H-6), 6.40 (dd, 1H, $^{3}J_{H,H}$ = 8.2 Hz, $^{4}J_{H,H}$ = 2.0 Hz, Ph H-5), 2.91 (t, 2H, $^{3}J_{H,H}$ = 7.1 Hz, CH_2Cl), 2.24 (t, 2H, $^{3}J_{H,H}$ = 7.1 Hz, Ph-CH_2).

^{13}**C NMR** (125 MHz, C_6D_6) δ: 137.4 (Ph C-4), 133.4 (Ph C-5), 131.6 (Ph C-6), 130.8 (Ph C-1), 128.5 (q, $^{2}J_{C,F}$ = 31.2 Hz, Ph C-2), 127.9 (Ph C-3), 123.6 (q, $^{1}J_{C,F}$ = 273.0 Hz, CF_3), 43.9 (CH_2Cl), 37.8 (Ph-CH_2).

^{19}**F NMR** (376 MHz, $CDCl_3$) δ: -62.7 (CF_3).

EI-MS m/z (%): 241.9 (M^+, 19), 193.0 (M^+, 100).

1-(2-Chloroethyl)-2-fluorobenzene (8)

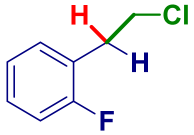

By following the **General procedure 1**, starting from 2-fluorobenzaldehyde (200 mg, 1.61 mmol, 1 equiv) in dry THF (3 mL), chloroiodomethane (0.18 mL, 2.42 mmol, 1.5 equiv), MeLi-LiBr 2.2 M solution in Et_2O (1.03 mL, 2.25 mmol, 1.4 equiv), tris(pentafluorophenyl)borane (82 mg, 0.2 mmol, 0.1 equiv) and hexylsilane (0.23 mL, 1.61 mmol, 1 equiv), **compound 8** was obtained in 83% yield (211 mg) as colorless oil after column chromatography on silica gel (*n*-hexane as eluent).

^{1}**H NMR** (500 MHz, $CDCl_3$) δ: 7.25 (m, 1H, Ph H-4), 7.23 (m, 1H, Ph H-6), 7.10 (m, 1H, Ph H-5), 7.04 (m, 1H, Ph H-3), 3.73 (t, 2H, $^{3}J_{H,H}$ = 7.3 Hz, CH_2Cl), 3.12 (t, 2H, $^{3}J_{H,H}$ = 7.3 Hz, Ph-CH_2).

^{13}**C NMR** (125 MHz, $CDCl_3$) δ: 161.2 (d, $^{1}J_{C,F}$ = 245.3 Hz, Ph C-2), 131.3 (d, $^{3}J_{C,F}$ = 4.6 Hz, Ph C-6), 128.7 (d, $^{3}J_{C,F}$ = 8.1 Hz Ph C-4), 124.9 (d, $^{2}J_{C,F}$ = 15.1 Hz, Ph C-1), 124.1 (d, $^{4}J_{C,F}$ = 3.5 Hz, Ph C-5), 115.4 (d, $^{2}J_{C,F}$ = 22.0 Hz, Ph C-3), 43.6 (CH_2Cl), 32.7 (Ph-CH_2).

4-(2-Chloroethyl)-*N*,*N*-dimethylaniline (9)

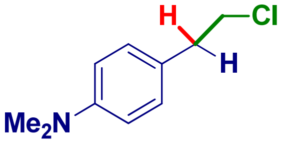

By following the **General procedure 1**, starting from 4-(dimethylamino)benzaldehyde (200 mg, 1.34 mmol, 1 equiv) in dry THF (3 mL), chloroiodomethane (0.15 mL, 2.01 mmol, 1.5 equiv) , MeLi-LiBr 2.2 M solution in Et_2O (0.85 mL, 1.9 mmol, 1.4 equiv), tris(pentafluorophenyl)borane (69 mg, 0.13 mmol, 0.1 equiv) and hexylsilane (0.13 mL, 1.34 mmol, 1 equiv), **compound 9** was obtained in 90% yield (222 mg) as colorless oil after column chromatography on silica gel (*n*-hexane/diethyl ether 9:1 as eluent).

^{1}H NMR (400 MHz, $CDCl_3$) δ: 7.10 (m, 2H, Ph H-3,5), 6.74 (m, 2H, Ph H-2,6), 3.66 (t, 2H, $^3J_{H,H}$ = 7.5 Hz, CH_2Cl), 2.98 (t, 2H, $^3J_{H,H}$ = 7.5 Hz, Ph-CH_2), 2.94 (s, 6H, N-CH_3).

^{13}C NMR (100 MHz, $CDCl_3$) δ: 149.4 (bs, Ph C-1), 129.5 (Ph C-3,5), 126.4 (Ph C-4), 113.0 (bs, Ph C-2,6), 45.4 (CH_2Cl), 40.9 (N-CH_3), 38.4 (Ph-CH_2)

HRMS (ESI), *m/z*: calcd. for $C_{10}H_{15}ClN^+$: 184.0888 $[M+H]^+$; found: 184.0892.

1-(2-Chloroethyl)naphthalene (10)

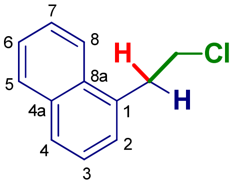

By following the **General procedure 1**, starting from 1-naphthaldehyde (200 mg, 1.28 mmol, 1 equiv) in dry THF (3 mL), chloroiodomethane (0.14 mL, 1.9 mmol, 1.5 equiv), MeLi-LiBr 2.2 M solution in Et_2O (0.82 mL, 1.8 mmol, 1.4 equiv), tris(pentafluorophenyl)borane (66 mg, 0.13 mmol, 0.1 equiv) and hexylsilane (0.21 mL, 1.28 mmol, 1 equiv), **compound 10** was obtained in 84% yield (205 mg) as colorless oil after column chromatography on silica gel (*n*-hexane as eluent).

^{1}H NMR (500 MHz, $CDCl_3$) δ: 8.05 (m, 1H, Naph H-8), 7.92 (m, 1H, Naph H-5), 7.82 (m, 1H, Naph H-4), 7.59 (m, H, Naph H-7), 7.54 (m, 1H, Naph H-6), 7.47 (m, 1H, Naph H-3), 7.42 (m, 1H, Naph H-2), 3.87 (t, 2H, $^3J_{H,H}$ = 7.8 Hz, CH_2Cl), 3.59 (t, 2H, $^3J_{H,H}$ = 7.8 Hz, Naph-CH_2).

^{13}C NMR (125 MHz, $CDCl_3$) δ: 133.9 (Naph C-4a), 133.8 (Naph C-8a), 131.6 (Naph C-1), 128.9 (Naph C-5), 127.7 (Naph C-4), 127.1 (Naph C-2), 126.3 (Naph C-7), 125.7 (Naph C-6), 125.4 (Naph C-3), 123.1 (Naph C-8), 44.1 (CH_2Cl), 36.4 (Naph-CH_2).

EI-MS m/z (%): 190.1 (M^+, 25), 141.1 (M^+, 100).

5-Bromo-4-(2-chloroethyl)-1,3-benzodioxole (11)

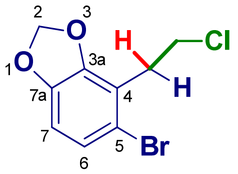

By following the **General procedure 1**, starting from 5-bromobenzo[*d*][1,3]dioxole-4-carbaldehyde (200 mg, 0.87 mmol, 1 equiv) in dry THF (3 mL), chloroiodomethane (0.1 mL, 1.3 mmol, 1.5 equiv) , MeLi-LiBr 2.2 M solution in Et_2O (0.55 mL, 1.2 mmol, 1.4 equiv), tris(pentafluorophenyl)borane (45 mg, 0.09 mmol, 0.1 equiv) and hexylsilane (0.14 mL, 0.87 mmol, 1 equiv), **compound 11** was obtained in 92% yield (211 mg) as colorless oil after column chromatography on silica gel (*n*-hexane as eluent).

^{1}H NMR (500 MHz, $CDCl_3$) δ: 7.02 (d, 1H, $^3J_{H,H}$ = 8.3 Hz, Benz H-6), 6.62 (d, 1H, $^3J_{H,H}$ = 8.3 Hz, Benz H-7), 5.99 (s, 2H, Benz H-2), 3.71 (m, 2H, CH_2Cl), 3.18 (m, 2H, Ph-CH_2).

^{13}C NMR (125 MHz, $CDCl_3$) δ: 147.4 (Benz C-3a), 146.7 (Benz C-7a), 125.3 (Benz C-6), 119.4 (Benz C-4), 115.7 (Benz C-5), 108.4 (Benz C-7), 101.6 (Benz C-2), 41.8 (CH_2Cl), 32.9 (Benz-CH_2).

2,4-Dichloro-1-(2-chloroethyl)benzene (12)

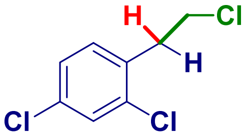

By following the **General procedure 1**, starting from 2,4-dichlorobenzaldehyde (200 mg, 0.89 mmol, 1 equiv) in dry THF (3 mL), chloroiodomethane (0.1 mL, 1.3 mmol, 1.5 equiv), MeLi-LiBr 2.2 M solution in Et_2O (0.57 mL, 1.3 mmol, 1.4 equiv), tris(pentafluorophenyl)borane (46 mg, 0.09 mmol, 0.1 equiv) and hexylsilane (0.14 mL, 0.89 mmol, 1 equiv), **compound 12** was obtained in 85% yield (158 mg) as colorless oil after column chromatography on silica gel (*n*-hexane as eluent).

^{1}H NMR (500 MHz, $CDCl_3$) δ: 7.39 (m, 1H, Ph H-3), 7.21 (m, 2H, Ph H-5,6), 3.73 (t, 2H, $^3J_{H,H}$ = 7.2 Hz, CH_2Cl), 3.17 (t, 2H, $^3J_{H,H}$ = 7.2 Hz Ph-CH_2).

^{13}C NMR (125 MHz, $CDCl_3$) δ: 134.7 (Ph C-2), 134.1 (Ph C-1), 133.5 (Ph C-4), 132.2 (Ph C-6), 129.4 (Ph C-6), 127.1 (Ph C-5), 42.8 (CH_2Cl), 36.3 (Ph-CH_2).

2-Chloro-1-(2-chloroethyl)-4-methylbenzene (13)

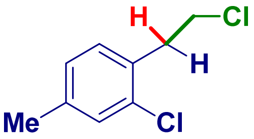

By following the **General procedure 1**, starting from 2-chloro-4-methylbenzaldehyde (200 mg, 1.29 mmol, 1 equiv) in dry THF (3 mL), chloroiodomethane (0.14 mL, 1.9 mmol, 1.5 equiv), MeLi-LiBr 2.2 M solution in Et_2O (0.82 mL, 1.8 mmol, 1.4 equiv), tris(pentafluorophenyl)borane (66 mg, 0.13 mmol, 0.1 equiv) and hexylsilane (0.21 mL, 1.29 mmol, 1 equiv), **compound 13** was obtained in 87% yield (212 mg) as colorless oil after column chromatography on silica gel (*n*-hexane as eluent).

^{1}**H NMR** (500 MHz, $CDCl_3$) δ: 7.20 (d, 1H, $^{4}J_{H,H}$ = 2.1 Hz, Ph H-3), 7.15 (d, 1H, $^{3}J_{H,H}$ = 7.7 Hz, Ph H-6), 7.03 (dd, 1H, $^{3}J_{H,H}$ = 7.7 Hz, $^{4}J_{H,H}$ = 2.1 Hz, Ph H-5), 3.73 (t, 2H, $^{3}J_{H,H}$ = 7.4 Hz, CH_2Cl), 3.16 (t, 2H, $^{3}J_{H,H}$ = 7.4 Hz, Ph-CH_2), 2.32 (s, 3H, CH_3).

^{13}**C NMR** (125 MHz, $CDCl_3$) δ: 138.6 (Ph C-4), 133.7 (Ph C-2), 132.4 (Ph C-1), 131.1 (Ph C-6), 130.1 (Ph C-3), 127.6 (Ph C-5), 43.3 (CH_2Cl), 36.5 (Ph-CH_2), 20.8 (CH_3).

1-(2-Chloroethyl)-2-(trifluoromethoxy)benzene (14)

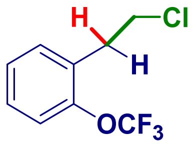

By following the **General procedure 1**, starting from 2-(trifluoromethoxy)benzaldehyde (200 mg, 1.05 mmol, 1 equiv) in dry THF (3 mL), chloroiodomethane (0.12 mL, 1.6 mmol, 1.5 equiv), MeLi-LiBr 2.2 M solution in Et_2O (0.67 mL, 1.5 mmol, 1.4 equiv), tris(pentafluorophenyl)borane (54 mg, 0.1 mmol, 0.1 equiv) and hexylsilane (0.17 mL, 1.05 mmol, 1 equiv), **compound 14** was obtained in 87% yield (205 mg) as colorless oil after column chromatography on silica gel (*n*-hexane as eluent).

^{1}**H NMR** (400 MHz, $CDCl_3$) δ: 7.32 (m, 1H, Ph H-6), 7.30 (m, 1H, Ph H-4), 7.26 (m, 2H, Ph H-3,5), 3.72 (t, $^{3}J_{H,H}$ = 7.3 Hz, 2H, CH_2Cl), 3.15 (m, $^{3}J_{H,H}$ = 7.3 Hz, 2H, Ph-CH_2).

^{13}**C NMR** (100 MHz, $CDCl_3$) δ: 147.7(q, $^{3}J_{H,F}$ = 1.5 Hz, Ph C-2), 131.5 (Ph C-6), 130.3 (Ph C-1), 128.5 (Ph C-4), 126.8 (1C, Ph C-5), 120.5 (q, $^{4}J_{H,F}$ = 1.5 Hz, 1C, Ph C-3), 120.5 (q, $^{1}J_{H,F}$ = 257.5 Hz, CF_3), 43.3 (CH_2Cl), 33.4 (Ph-CH_2).

^{19}**F NMR** (376 MHz, $CDCl_3$) δ: -57.0 (s, CF_3).

2-(2-Chloroethyl)-1,3-dimethylbenzene (15)

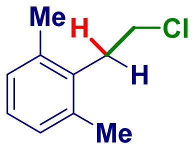

By following the **General procedure 1**, starting from 1,3-dimethylbenzaldehyde (200 mg, 1.49 mmol, 1 equiv) in dry THF (3 mL), chloroiodomethane (0.16 mL, 2.24 mmol, 1.5 equiv), MeLi-LiBr 2.2 M solution in Et_2O (1.0 mL, 2.1 mmol, 1.4 equiv), tris(pentafluorophenyl)borane (76 mg, 0.15 mmol, 0.1 equiv) and hexylsilane (0.24 mL, 1.49 mmol, 1 equiv), **compound 15** was obtained in 88% yield (221 mg) as colorless oil without any further purification.

^{1}**H NMR** (500 MHz, $CDCl_3$) δ: 7.05 (m, 1H, Ph H-5), 7.03 (m, 2H, Ph H-4,6), 3.56 (m, 2H, CH_2Cl), 3.14 (m, 2H, Ph-CH_2), 2.36 (s, 6H, CH_3).

^{13}**C NMR** (125 MHz, $CDCl_3$) δ: 136.7 (Ph C-1,3), 134.6 (Ph C-2), 128.4 (Ph C-4,6), 126.8 (Ph C-5), 42.2 (CH_2Cl), 33.4 (Ph-CH_2), 19.8 (CH_3).

1,3-Dichloro-2-(2-chloroethyl)benzene (16)

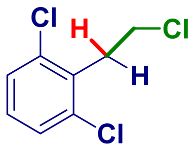

By following the **General procedure 1**, starting from 2,6-dichlorobenzaldehyde (200 mg, 1.14 mmol, 1 equiv) in dry THF (3 mL), chloroiodomethane (0.1 mL, 1.17 mmol, 1.5 equiv), MeLi-LiBr 2.2 M solution in Et_2O (0.7 mL, 1.6 mmol, 1.4 equiv), tris(pentafluorophenyl)borane (58 mg, 0.11 mmol, 0.1 equiv) and hexylsilane (0.18 mL, 1.14 mmol, 1 equiv), **compound 16** was obtained in 94% yield (224 mg) as colorless oil after column chromatography on silica gel (*n*-hexane as eluent).

^{1}H NMR (500 MHz, $CDCl_3$) δ: 7.30 (d, 2H, $^3J_{H,H}$ = 8.1 Hz, Ph H-4,6), 7.14 (m, 1H, Ph H-5), 3.69 (m, 2H, CH_2Cl), 3.43 (m, 2H, Ph-CH_2).

^{13}C NMR (125 MHz, $CDCl_3$) δ: 135.8 (Ph C-1,3), 133.8 (Ph C-2), 128.7 (Ph C-5), 128.3 (Ph C-4,6), 41.1 (CH_2Cl), 34.4 (Ph-CH_2).

EI-MS m/z (%): 208.0 (M^+, 24), 159.0 (M^+, 100).

(3-Chloropropyl)benzene (17)

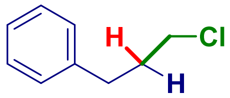

By following the **General procedure 1**, starting from 2-phenylacetaldehyde (200 mg, 1.66 mmol, 1 equiv) in dry THF (3 mL), chloroiodomethane (0.18 mL, 2.49 mmol, 1.5 equiv), MeLi-LiBr 2.2 M solution in Et_2O (1.1 mL, 2.3 mmol, 1.4 equiv), tris(pentafluorophenyl)borane (85 mg, 0.17 mmol, 0.1 equiv) and hexylsilane (0.27 mL, 1.66 mmol, 1 equiv), **compound 17** was obtained in 90% yield (231 mg) as colorless oil after column chromatography on silica gel (*n*-hexane as eluent).

^{1}H NMR (500 MHz, $CDCl_3$) δ: 7.33 (m, 2H, Ph H-3,5), 7.24 (m, 3H, Ph H-2,4,6), 3.56 (t, 2H, $^3J_{H,H}$ = 6.5 Hz, CH_2Cl), 2.81 (t, 2H, $^3J_{H,H}$ = 7.6 Hz, Ph-CH_2), 2.13 (m, 2H, CH_2-C$\underline{H}_2$-CH_2).

^{13}C NMR (125 MHz, $CDCl_3$) δ: 140.7 (Ph C-1), 128.5 (Ph C-2,3,5,6), 126.1 (Ph C-4), 44.2 (CH_2Cl), 34.0 (CH_2-$\underline{C}H_2$-CH_2), 32.7 (Ph-CH_2).

EI-MS m/z (%): 154.0 (M^+, 18), 105.1 (M^+, 100).

(4-Chlorobutyl)benzene (18)

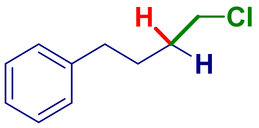

By following the **General procedure 1**, starting from 3-phenylpropanal (200 mg, 1.49 mmol, 1 equiv) in dry THF (3 mL), chloroiodomethane (0.16 mL, 2.24 mmol, 1.5 equiv), MeLi-LiBr 2.2 M solution in Et_2O (0.95 mL, 2.1 mmol, 1.4 equiv), tris(pentafluorophenyl)borane (76 mg, 0.15 mmol, 0.1 equiv) and hexylsilane (0.24 mL,

1.49 mmol, 1 equiv), **compound 18** was obtained in 86% yield (216 mg) as colorless oil after column chromatography on silica gel (*n*-hexane as eluent).

^{1}H NMR (500 MHz, $CDCl_3$) δ: 7.30 (m, 2H, Ph H-3,5), 7.21 (m, 1H, Ph H-4), 7.20 (m, 2H, Ph H-2,6), 3.56 (m, 2H, CH_2Cl), 2.66 (t, 2H, $^{3}J_{H,H}$ = 7.2 Hz, Ph-CH_2), 1.82 (m, 2H, H-3), 1.80 (m, 2H, H-2).

^{13}C NMR (125 MHz, $CDCl_3$) δ: 141.8 (Ph C-1), 128.3 (Ph C-2,3,5,6), 125.9 (Ph C-4), 44.9 (CH_2Cl), 35.1 (Ph-CH_2), 32.0 (C-3), 28.5 (C-2).

EI-MS m/z (%): 168.0 (M^+, 18), 91.1 (M^+, 100).

(4-Chloro-1-butyn-1-yl)benzene (19)

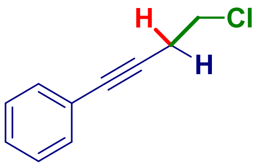

By following the **General procedure 1**, starting from 3-phenylpropionaldehyde (200 mg, 1.53 mmol, 1 equiv) in dry THF (3 mL), chloroiodomethane (0.17 mL, 2.3 mmol, 1.5 equiv), MeLi-LiBr 2.2 M solution in Et_2O (0.95 mL, 2,1 mmol, 1.4 equiv), tris(pentafluorophenyl)borane (78 mg, 0.15 mmol, 0.1 equiv) and hexylsilane (0.25 mL, 1.53 mmol, 1 equiv), **compound 19** was obtained in 91% yield (230 mg) as colorless oil after column chromatography on silica gel (*n*-hexane as eluent).

^{1}H NMR (500 MHz, C_6D_6) δ: 7.44 (m, 2H, Ph H-2,6), 6.97 (m, 3H, Ph H-3,4,5), 3.08 (t, 2H, $^{3}J_{H,H}$ = 7.0 Hz, CH_2Cl), 2.34 (t, 2H, $^{3}J_{H,H}$ = 7.0 Hz, H-3).

^{13}C NMR (125 MHz, C_6D_6) δ: 132.0 (Ph C-2,6), 128.6 (Ph C-3,5), Ph C-4 not found, 124.0 (Ph C-1), 86.4 (C-2), 83.0 (C-1), 42.2 (CH_2Cl), 23.8 (C-3).

[4-(Chloromethyl)cyclohexyl]benzene (20)

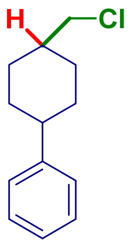

By following the **General procedure 1**, starting from 4-phenylcyclohexanone (200 mg, 1.15 mmol, 1 equiv) in dry THF (3 mL), chloroiodomethane (0.13 mL, 1.7 mmol, 1.5 equiv) , MeLi-LiBr 2.2 M solution in Et_2O (0.73 mL, 1.6 mmol, 1.4 equiv), tris(pentafluorophenyl)borane (59 mg, 0.12 mmol, 0.1 equiv) and hexylsilane (0.19 mL, 1.15 mmol, 1 equiv), **compound 20** was obtained in 92% yield (221 mg) as colorless oil after column chromatography on silica gel (*n*-hexane as eluent).

ISOMER 1 (major isomer)

^{1}**H NMR** (400 MHz, $CDCl_3$) δ: 7.32 (m, 2H, Ph H-3,5), 7.24 (m, 2H, Ph H-2,6), 7.21 (m, 1H, Ph H-4), 3.47 (d, 2H, $^3J_{H,H}$ = 6.3 Hz, CH_2Cl), 2.51 (m, 1H, Cyclo H-1), 2.02 (m, 2H, H-3), 2.02 and 1.23 (m, 4H, Cyclo H-3,5), 1.99 and 1.52 (m, 4H, Cyclo H-2,6), 1.75 (m, 1H, Cyclo H-4).

^{13}**C NMR** (100 MHz, $CDCl_3$) δ: 147.1 (Ph C-1), 128.4 (Ph C-3,5), 126.8 (Ph C-2,6), 126.0 (Ph C-4), 50.9 (CH_2Cl), 44.1 (Cyclo C-1), 39.9 (Cyclo C-4), 33.6 (Cyclo C-2,6), 30.9 (Cyclo C-3,5).

ISOMER 2 (minor isomer)

^{1}**H NMR** (400 MHz, $CDCl_3$) δ: 7.32 (m, 2H, Ph H-3,5), 7.24 (m, 2H, Ph H-2,6), 7.21 (m, 1H, Ph H-4), 3.65 (d, 2H, $^3J_{H,H}$ = 7.8 Hz, CH_2Cl), 2.65 (m, 1H, Cyc H-1), 2.09 (m, 1H, Cyc H-4), 1.87 and 1.74 (m, 4H, Cyc H-3,5), 1.74 (m, 4H, Cyc H-2,6).

^{13}**C NMR** (100 MHz, $CDCl_3$) δ: 146.6 (Ph C-1), 128.4 (Ph C-3,5), 126.9 (Ph C-2,6), 125.9 (Ph C-4), 47.3 (CH_2Cl), 43.1 (Cyc C-1), 36.1 (Cyc C-4), 28.7 (Cyc C-2,6), 27.9 (Cyc C-3,5).

5-(Chloromethyl)nonane (21)

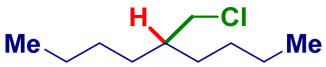

By following the **General procedure 1**, starting from nonan-5-one (200 mg, 1.41 mmol, 1 equiv) in dry THF (3 mL), chloroiodomethane (0.15 mL, 2.1 mmol, 1.5 equiv) , MeLi-LiBr 2.2 M solution in Et_2O (0.91 mL, 2.0 mmol, 1.4 equiv), tris(pentafluorophenyl)borane (72 mg, 0.14 mmol, 0.1 equiv) and hexylsilane (0.23 mL, 1.41 mmol, 1 equiv), **compound 21** was obtained in 92% yield (229 mg) as colorless oil after column chromatography on silica gel (*n*-hexane as eluent).

^{1}**H NMR** (400 MHz, $CDCl_3$) δ: 3.53 (d, 2H, $^3J_{H,H}$= 5.0 Hz, CH_2Cl), 1.65 (m, 1H, CH), 1.36 (m, 4H, H-4,6), 1.30 (m, 4H, H-2,8), 1.28 (m, 4H, H-3,7), 0.90 (t, 6H, $^3J_{H,H}$= 7.1 Hz, CH_3).

^{13}**C NMR** (100 MHz, $CDCl_3$) δ: 48.9 (CH_2Cl), 39.9 (CH), 31.3 (C-4,6), 28.8 (C-3,7), 22.9 (C-2,8), 14.0 (CH_3).

1-(Chloromethyl)indane (22)

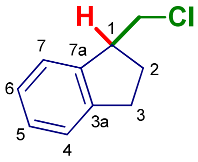

By following the **General procedure 1**, starting from 2,3-dihydro-1*H*-inden-1-one (200 mg, 1.51 mmol, 1 equiv) in dry THF (3 mL), chloroiodomethane (0.17 mL, 2.3 mmol, 1.5 equiv) , MeLi-LiBr 2.2 M solution in Et_2O (0.96 mL, 2.1 mmol, 1.4 equiv), tris(pentafluorophenyl)borane (77 mg, 0.15 mmol, 0.1 equiv) and hexylsilane (0.24 mL, 1.51 mmol, 1 equiv), **compound 22** was obtained in 91%yield (229 mg) as colorless oil after column chromatography on silica gel (*n*-hexane as eluent).

Scaling-up of the reaction (15 mmol) - By following the **General procedure 1**, employing 2,3-dihydro-1*H*-inden-1-one (1982 mg, 15.0 mmol, 1 equiv) in dry THF (30 mL), chloroiodomethane (1.66 mL, 22.5 mmol, 1.5 equiv) , MeLi-LiBr 2.2 M solution in Et_2O (9.6 mL, 21.0 mmol, 1.4 equiv), tris(pentafluorophenyl)borane (770 mg, 1.5 mmol, 0.1 equiv) and hexylsilane (2.4 mL, 15.0 mmol, 1 equiv), **compound 22** was obtained in 87%

yield (2459 mg) as as colorless oil after column chromatography on silica gel (*n*-hexane as eluent). *Spectroscopic and spectrometric data match with those reported for the 1.51 mmol scale reaction.*

^{1}H NMR (500 MHz, $CDCl_3$) δ: 7.31 (m, 1H, Ind H-7), 7.27 (m, 1H, Ind H-4), 7.23 (m, 1H, Ind H-5), 7.22 (m, 1H, Ind H-6), 3.87 (dd, 1H, $^2J_{H,H}$ = 10.4 Hz, $^3J_{H,H}$ = 4.7 Hz, CH_2Cl), 3.62 (dd, 1H, $^2J_{H,H}$ = 10.4 Hz, $^3J_{H,H}$ = 8.2 Hz, CH_2Cl), 3.57 (m, 1H, Ind H-1), 3.01 (m, 1H, Ind H-3), 2.92 (m, 1H, Ind H-3), 2.39 (m, 1H, Ind H-2), 2.02 (m, 1H, Ind H-2).

^{13}C NMR (125 MHz, $CDCl_3$) δ: 144.4 (Ind C-3a), 143.3 (Ind C-7a), 127.3 (Ind C-5), 126.3 (Ind C-6), 124.8 (Ind C-4), 124.0 (Ind C-), 48.2 (CH_2Cl), 47.4 (Ind C-1), 30.9 (Ind C-3), 30.0 (Ind C-2).

5-(Chloromethyl)-5,6,7,8-tetrahydro-2,3-naphthalenediol (23)

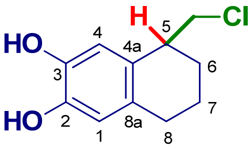

By following the **General procedure 1**, starting from 6,7-dihydroxy-3,4-dihydro-2*H*-naphthalen-1-one (200 mg, 1.12 mmol, 1 equiv) in dry THF (3 mL), chloroiodomethane (0.12 mL, 1.7 mmol, 1.5 equiv) , MeLi-LiBr 2.2 M solution in Et_2O (0.71 mL, 1.6 mmol, 1.4 equiv), tris(pentafluorophenyl)borane (57 mg, 0.11 mmol, 0.1 equiv), and hexylsilane (0.18 mL, 1.12 mmol, 1 equiv), **compound 23** was obtained in 86% yield (205 mg) as colorless oil after column chromatography on silica gel (*n*-hexane/dichloromethane 9:1 as eluent).

^{1}H NMR (400 MHz, $CDCl_3$) δ: 6.69 (s, 1H, Naph H-4), 6.59 (s, 1H, Naph H-1), 5.24 (bs, 2H, OH), 3.71 (A-part of an AB system, $^2J_{H,H}$ = 11.0 Hz, $^3J_{H,H}$ = 4.1 Hz, 1H, CH_2Cl), 3.58 (B-part of an AB system, $^2J_{H,H}$ = 11.0 Hz, $^3J_{H,H}$ = 9.9 Hz, 1H, CH_2Cl), 3.00 (m, 1H, Naph H-5), 2.68 – 2.60 (m, 2H, Naph H-8), 2.03 and 1.84 (m, 2H, Naph H-6), 1.79 – 1.67 (m, 2H, Naph H-7).

^{13}C NMR (100 MHz, $CDCl_3$) δ: 142.2 (Naph C-3), 141.5 (Naph C-2), 130.5 (Naph C-8a), 129.0 (Naph C-4a), 115.6 (Naph C-1), 115.5 (Naph C-4), 49.1 (CH_2Cl), 39.7 (Naph C-5), 28.9 (Naph C-8), 25.3 (Naph C-6), 19.0 (Naph C-7).

HRMS (ESI), *m/z*: calcd. for $C_{11}H_{13}ClNaO_2^+$: 235.0496 $[M+Na]^+$; found: 235.0498.

1-(1-Chloro-2-propanyl)-4-iodobenzene (24)

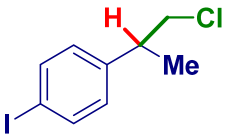

By following the **General procedure 1**, starting from 1-(4-iodophenyl)ethanone (200 mg, 0.81 mmol, 1 equiv) in dry THF (3 mL), chloroiodomethane (0.1 mL, 1.2 mmol, 1.5 equiv) , MeLi-LiBr 2.2 M solution in Et_2O (0.5 mL, 1.1 mmol, 1.4 equiv), tris(pentafluorophenyl)borane (42 mg, 0.1 mmol, 0.1 equiv) and hexylsilane (0.13 mL, 0.81 mmol, 1 equiv), **compound 24** was obtained in 92% yield (209 mg) as colorless oil after column chromatography on silica gel (*n*-hexane as eluent).

^{1}H NMR (500 MHz, $CDCl_3$) δ: 7.65 (m, 2H, Ph H-3,5), 6.98 (m, 2H, Ph H-2,6), 3.64 (dd, 1H, $^{2}J_{H,H}$ = 10.8 Hz, $^{3}J_{H,H}$ = 6.5 Hz, H-1a), 3.57 (dd, 1H, $^{2}J_{H,H}$ = 10.8 Hz, $^{3}J_{H,H}$ = 7.3 Hz, H-1b), 3.05 (m, 1H, CH), 1.36 (d, 3H, $^{3}J_{H,H}$ = 7.0 Hz, CH_3).

^{13}C NMR (125 MHz, $CDCl_3$) δ: 142.9 (Ph C-1), 137.6 (Ph C-3,5), 129.3 (Ph C-2,6), 92.2 (Ph C-4), 50.3 (CH_2Cl), 41.8 (CH), 18.9 (CH_3).

EI-MS m/z (%): 280.0 (M^+, 30), 231.0 (M^+, 100).

1-Bromo-4-(1-chloro-2-propanyl)benzene (25)

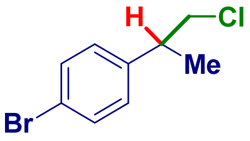

By following the **General procedure 1**, starting from 1-(4-bromophenyl)ethanone (200 mg, 1.0 mmol, 1 equiv) in dry THF (3 mL), chloroiodomethane (0.11 mL, 1.5 mmol, 1.5 equiv) , MeLi-LiBr 2.2 M solution in Et_2O (0.64 mL, 1.4 mmol, 1.4 equiv), tris(pentafluorophenyl)borane (51 mg, 0.1 mmol, 0.1 equiv) and hexylsilane (0.2 mL, 1.0 mmol, 1 equi), **compound 25** was obtained in 89% yield (208 mg) as colorless oil after column chromatography on silica gel (*n*-hexane as eluent).

^{1}H NMR (500 MHz, $CDCl_3$) δ: 7.45 (m, 2H, Ph H-2,6), 7.11 (m, 2H, Ph H-3,5), 3.64 (dd, 1H, $^{2}J_{H,H}$ = 10.8 Hz, $^{3}J_{H,H}$ = 6.5 Hz, CH_2), 3.58 (dd, 1H, $^{2}J_{H,H}$ = 10.8 Hz, $^{3}J_{H,H}$ = 7.2 Hz, CH_2), 3.07 (m, 1H, CH), 1.37 (d, 3H, $^{3}J_{H,H}$ = 7.0Hz, CH_3).

^{13}C NMR (125 MHz, $CDCl_3$) δ: 142.2 (Ph C-4), 131.6 (Ph C-2,6), 128.9 (Ph C-3,5), 120.7 (Ph C-1), 50.4 (CH_2Cl), 41.7 (CH), 18.9 (CH_3).

EI-MS m/z (%): 232.0 (M^+, 20), 183.0 (M^+, 100).

1-Chloro-4-(1-chloropropan-2-yl)benzene (26)

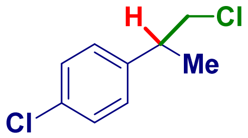

By following the **General procedure 1**, starting from 1-(4-chlorophenyl)ethanone (200 mg, 1.3 mmol, 1 equiv) in dry THF (3 mL), chloroiodomethane (0.14 mL, 2.0 mmol, 1.5 equiv), MeLi-LiBr 2.2 M solution in Et_2O (0.82 mL, 1.8 mmol, 1.4 equiv), tris(pentafluorophenyl)borane (67 mg, 0.1 mmol, 0.1 equiv) and hexylsilane (0.21 mL, 1.3 mmol, 1 equiv), **compound 26** was obtained in 86% yield (211 mg) as colorless oil after column chromatography on silica gel (*n*-hexane as eluent).

^{1}H NMR (500 MHz, $CDCl_3$) δ: 7.30 (m, 2H, Ph H-2,6), 7.16 (m, 2H, Ph H-3,5), 3.64 (dd, 1H, $^{2}J_{H,H}$ = 10.8 Hz, $^{3}J_{H,H}$ = 6.4 Hz, CH_2), 3.58 (dd, 1H, $^{2}J_{H,H}$ = 10.8 Hz, $^{3}J_{H,H}$ = 7.3 Hz, CH_2), 3.09 (m, 1H, CH), 1.37 (d, 3H, $^{3}J_{H,H}$ = 7.0 Hz, CH_3).

^{13}C NMR (125 MHz, $CDCl_3$) δ: 141.7 (Ph C-4), 132.6 (Ph C-1), 128.7 (Ph C-2,6), 128.6 (Ph C-3,5), 50.5 (CH_2Cl), 41.7 (CH), 19.0 (CH_3).

2,4-Dichloro-1-(1-chloropropan-2-yl)benzene (27)

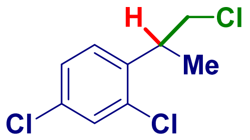

By following the **General procedure 1**, starting from 1-(2,4-dichlorophenyl)ethanone (200 mg, 1.06 mmol, 1 equiv) in dry THF (3 mL), chloroiodomethane (0.1 mL, 1.6 mmol, 1.5 equiv), MeLi-LiBr 2.2 M solution in Et_2O (0.68 mL, 1.5 mmol, 1.4 equiv), tris(pentafluorophenyl)borane (54 mg, 0.1 mmol, 0.1 equiv) and hexylsilane (0.17 mL, 1.06 mmol, 1 equiv), **compound 27** was obtained in 92% yield (218 mg) as colorless oil after column chromatography on silica gel (*n*-hexane as eluent).

^{1}H NMR (500 MHz, $CDCl_3$) δ: 7.40 (d, 1H, $^4J_{H,H}$ = 2.1 Hz, Ph H-3), 7.25 (dd, 1H, $^3J_{H,H}$ = 8.4 Hz, $^4J_{H,H}$ = 2.1 Hz, Ph H-5), 7.21 (d, 1H, $^3J_{H,H}$ = 8.4 Hz, Ph H-6), 3.72 (dd, 1H, $^2J_{H,H}$ = 10.1 Hz, $^3J_{H,H}$ = 5.2 Hz, CH_2), 3.65 (m, 1H, CH), 3.60 (dd, 1H, $^2J_{H,H}$ = 10.1 Hz, $^3J_{H,H}$ = 6.7 Hz, CH_2), 1.38 (d, 3H, $^3J_{H,H}$ = 6.7 Hz, CH_3).

^{13}C NMR (125 MHz, $CDCl_3$) δ: 138.8 (Ph C-1), 134.5 (Ph C-2), 133.1 (Ph C-4), 129.5 (Ph C-3), 128.6 (Ph C-6), 127.3 (Ph C-5), 49.0 (CH_2Cl), 37.4 (CH), 17.8 (CH_3).

1-(1-Chloro-2-propanyl)-4-fluorobenzene (28)

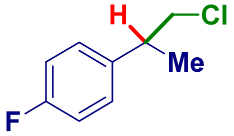

By following the **General procedure 1**, starting from 1-(4-fluorophenyl)ethanone (200 mg, 1.5 mmol, 1 equiv) in dry THF (3 mL), chloroiodomethane (0.16 mL, 2.3 mmol, 1.5 equiv), MeLi-LiBr 2.2 M solution in Et_2O (1.0 mL, 2.1 mmol, 1.4 equiv), tris(pentafluorophenyl)borane (102 mg, 0.2 mmol, 0.1 equiv) and hexylsilane (0.24 mL, 1.5 mmol, 1 equiv), **compound 28** was obtained in 92% yield (238 mg) as colorless oil after column chromatography on silica gel (*n*-hexane as eluent).

^{1}H NMR (500 MHz, C_6D_6) δ: 6.75 (m, 2H, Ph H-3,5), 6.63 (m, 2H, Ph H-2,6), 3.17 (dd, 1H, $^2J_{H,H}$ = 10.8 Hz, $^3J_{H,H}$ = 6.2 Hz, CH_2), 3.07 (dd, 1H, $^2J_{H,H}$ = 10.8 Hz, $^3J_{H,H}$ = 7.7 Hz, CH_2), 2.62 (m, 1H, CH), 1.00 (d, 3H, $^3J_{H,H}$ = 7.0 Hz, CH_3).

^{13}C NMR (125 MHz, C_6D_6) δ: 162.2 (d, $^1J_{C,F}$ = 244.1 Hz, Ph C-4), 139.2 (d, $^4J_{C,F}$ = 2.8 Hz, Ph C-1), 128.9 (d, $^3J_{C,F}$ = 8.1 Hz, Ph C-2,6), 115.5 (d, $^2J_{C,F}$ = 21.6 Hz, Ph C-3,5), 50.6 (CH_2Cl), 41.6 (CH), 18.9 (CH_3).

1-(1-Chloro-2-propanyl)-2,4-difluorobenzene (29)

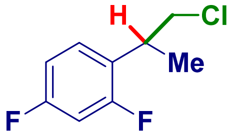

By following the **General procedure 1**, starting from 1-(2,4-difluorophenyl)ethanone (200 mg, 1.28 mmol, 1 equiv) in dry THF (3 mL), chloroiodomethane (0.14 mL, 1.9 mmol, 1.5 equiv), MeLi-LiBr 2.2 M solution in Et_2O (0.8 mL, 1.8 mmol, 1.4 equiv), tris(pentafluorophenyl)borane (66 mg, 0.13 mmol, 0.1 equiv) and hexylsilane (0.21 mL, 1.28 mmol, 1 equiv), **compound 29** was obtained in 87% yield (212 mg) as colorless oil after column chromatography on silica gel (*n*-hexane as eluent).

^{1}H NMR (500 MHz, $CDCl_3$) δ: 7.20 (m, 1H, Ph H-6), 6.85 (m, 1H, Ph H-5), 6.80 (m, 1H, Ph H-3), 3.71 (dd, 1H, $^{2}J_{H,H}$ = 10.7 Hz, $^{3}J_{H,H}$ = 6.3 Hz, CH_2), 3.63 (dd, 1H, $^{2}J_{H,H}$ = 10.7 Hz, $^{3}J_{H,H}$ = 7.0 Hz, CH_2), 3.41 (m, 1H, CH), 1.39 (d, 3H, $^{3}J_{H,H}$ = 7.0 Hz, CH_3).

^{13}C NMR (125 MHz, $CDCl_3$) δ: 161.9 (dd, $^{1}J_{C,F}$ = 248.0 Hz, $^{3}J_{C,F}$ = 12.8 Hz, Ph C-4), 160.7 (dd, $^{1}J_{C,F}$ = 248.6 Hz, $^{3}J_{C,F}$ = 11.6 Hz, Ph C-2), 129.2 (dd, $^{3}J_{C,F}$ = 9.9 Hz, $^{3}J_{C,F}$ = 6.5 Hz, Ph C-6), 125.8 (dd, $^{2}J_{C,F}$ = 15.0 Hz, $^{4}J_{C,F}$ = 3.6 Hz, Ph C-1), 111.2 (dd, $^{2}J_{C,F}$ = 20.7 Hz, $^{4}J_{C,F}$ = 3.6 Hz, Ph C-5), 104.0 (dd, $^{2}J_{C,F}$ = 26.6 Hz, $^{2}J_{C,F}$ = 25.2 Hz, Ph C-3), 49.2 (CH_2Cl), 35.3 (CH), 17.8 (CH_3).

^{19}F NMR (470 MHz, $CDCl_3$) δ: -112.4 (1F, F-4), -114.3 (m, 1F, F-2),.

1-(1-Chloro-2-propanyl)-4-(trifluoromethyl)benzene (30)

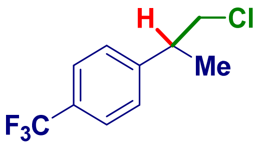

By following the **General procedure 1**, starting from 1-(4-(trifluoromethyl)phenyl)ethanone (200 mg, 0.83 mmol, 1 equiv) in dry THF (3 mL), chloroiodomethane (0.1 mL, 1.3 mmol, 1.5 equiv) , MeLi-LiBr 2.2 M solution in Et_2O (0.5 mL, 1.2 mmol, 1.4 equiv), tris(pentafluorophenyl)borane (43 mg, 0.1 mmol, 0.1 equiv) and hexylsilane (0.13 mL, 0.83 mmol, 1 equiv), **compound 30** was obtained in 90% yield (166 mg) as colorless oil after column chromatography on silica gel (*n*-hexane as eluent).

^{1}H NMR (500 MHz, $CDCl_3$) δ: 7.59 (m, 2H, Ph H-3,5), 7.35 (m, 2H, Ph H-2,6), 3.68 (dd, 1H, $^{2}J_{H,H}$ = 10.9 Hz, $^{3}J_{H,H}$ = 6.7 Hz, CH_2), 3.63 (dd, 1H, $^{2}J_{H,H}$ = 10.9 Hz, $^{3}J_{H,H}$ = 7.0 Hz, CH_2), 3.18 (m, 1H, CH), 1.41 (d, 3H, $^{3}J_{H,H}$ = 7.0 Hz, CH_3).

^{13}C NMR (125 MHz, $CDCl_3$) δ: 147.9 (Ph C-1), 129.3 (q, $^{2}J_{C,F}$ = 32.4 Hz, Ph C-4), 127.6 (Ph C-2,6), 125.5 (q, $^{3}J_{C,F}$ = 3.5 Hz, Ph C-3,5), 124.1 (q, $^{1}J_{C,F}$ = 271.9 Hz, CF_3), 50.1 (CH_2Cl), 42.1 (CH), 19.0 (CH_3).

^{19}F NMR (376 MHz, $CDCl_3$) δ: -62.5 (3F, CF_3).

1-(1-Chloro-2-propanyl)-4-ethylbenzene (31)

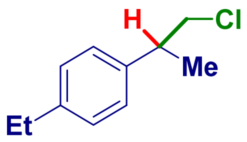

By following the **General procedure 1**, starting from 1-(4-ethylphenyl)ethanone (200 mg, 1.35 mmol, 1 equiv) in dry THF (3 mL), chloroiodomethane (0.15 mL, 2.03 mmol, 1.5 equiv) , MeLi-LiBr 2.2 M solution in Et_2O (0.9 mL, 1.9 mmol, 1.4 equiv), tris(pentafluorophenyl)borane (69 mg, 0.14 mmol, 0.1 equiv) and hexylsilane (0.22 mL, 1.35 mmol, 1 equiv), **compound 31** was obtained in 95 % yield (234 mg) as colorless oil after column chromatography on silica gel (*n*-hexane as eluent).

^{1}H NMR (500 MHz, $CDCl_3$) δ: 7.16 (m, 2H, Ph H-3,5), 7.15 (m, 2H, Ph H-2,6), 3.68 (dd, 1H, $^{2}J_{H,H}$ = 10.7 Hz $^{3}J_{H,H}$ = 6.0 Hz, CH_2Cl), 3.57 (dd, 1H, $^{2}J_{H,H}$ = 10.7 Hz, $^{3}J_{H,H}$ = 8.0 Hz, CH_2Cl), 3.08 (m, 1H, CH), 2.64 (q, $^{3}J_{H,H}$ = 7.7 Hz, Ph-CH_2), 1.39 (d, 3H, $^{3}J_{H,H}$ = 6.9 Hz, CHC<u>H</u>$_3$), 1.27 (t, 3H, $^{3}J_{H,H}$ = 7.7 Hz, CH_3).

^{13}**C NMR** (125 MHz, $CDCl_3$) δ: 142.9 (Ph C-4), 140.6 (Ph C-1), 128.0 (Ph C-3,5), 127.1 (Ph C-2,6), 51.0 (CH_2Cl), 41.9 (CH), 28.4 (Ph-CH_2), 18.9 (CH$\underline{C}H_3$), 15.5 ($CH_2\underline{C}H_3$).

1-(1-chloro-2-propanyl)-4-(2-methyl-2-propanyl)benzene (32)

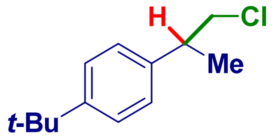

By following the **General procedure 1**, starting from 1-[4-(*tert*-butyl)phenyl]ethanone (200 mg, 1.14 mmol, 1 equiv) in dry THF (3 mL), chloroiodomethane (0.13 mL, 1.7 mmol, 1.5 equiv) , MeLi-LiBr 2.2 M solution in Et_2O (0.73 mL, 1.6 mmol, 1.4 equiv), tris(pentafluorophenyl)borane (58 mg, 0.12 mmol, 0.1 equiv) and hexylsilane (0.2 mL, 1.14 mmol, 1 equiv), **compound 32** was obtained in 95 % yield (228 mg) as colorless oil after column chromatography on silica gel (*n*-hexane as eluent).

^{1}**H NMR** (500 MHz, $CDCl_3$) δ: 7.35 (m, 2H, Ph H-3,5), 7.16 (m, 2H, Ph H-2,6), 3.69 (dd, 1H, $^{2}J_{H,H}$ = 10.7 Hz, $^{3}J_{H,H}$ = 5.8 Hz,CH_2), 3.56 (dd, 1H, $^{2}J_{H,H}$ = 10.7 Hz, $^{3}J_{H,H}$ = 8.2 Hz, CH_2), 3.08 (m, 1H, CH), 1.39 (d, 3H, $^{3}J_{H,H}$ = 7.0 Hz, CHC$\underline{H}_3$), 1.32 (s, 9H, C-C$\underline{H}_3$).

^{13}**C NMR** (125 MHz, $CDCl_3$) δ: 149.7 (Ph C-4), 140.2 (Ph C-1), 126.8 (Ph C-2,6), 125.4 (Ph C-3,5), 51.0 (CH_2Cl), 41.8 (CH), 34.4 [$\underline{C}(CH_3)_3$], 31.3 [$C(\underline{C}H_3)_3$], 18.8 (CH$\underline{C}H_3$).

EI-MS m/z (%): 210.1 (M^+, 17), 195.1 (M^+, 100).

1-(1-Chloro-2-propanyl)-4-methoxybenzene (33)

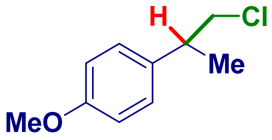

By following the **General procedure 1**, starting from 1-(4-methoxyphenyl)ethanone (200 mg, 1.33 mmol, 1 equiv) in dry THF (3 mL), chloroiodomethane (0.15 mL, 2.0 mmol, 1.5 equiv) , MeLi-LiBr 2.2 M solution in Et_2O (0.9 mL, 1.9 mmol, 1.4 equiv), tris(pentafluorophenyl)borane (68 mg, 0.13 mmol, 0.1 equiv) and hexylsilane (0.22 mL, 1.33 mmol, 1 equiv), **compound 33** was obtained in 92 % yield (226 mg) as colorless oil after column chromatography on silica gel (*n*-hexane as eluent).

^{1}**H NMR** (500 MHz, $CDCl_3$) δ: 7.15 (m, 2H, Ph H-2,6), 6.87 (m, 2H, Ph H-3,5), 3.80 (s, 3H, OCH_3), 3.65 (dd, 1H, $^{2}J_{H,H}$ = 10.7 Hz, $^{3}J_{H,H}$ = 6.1 Hz, CH_2), 3.55 (dd, 1H, $^{2}J_{H,H}$ = 10.7 Hz, $^{3}J_{H,H}$ = 7.9 Hz, CH_2), 3.06 (m, 1H, CH), 1.38 (d, 3H, $^{3}J_{H,H}$ = 7.0 Hz, CH_3).

^{13}**C NMR** (125 MHz, $CDCl_3$) δ: 158.5 (Ph C-4), 135.4 (Ph C-1), 128.1 (Ph C-2,6), 113.9 (Ph C-3,5), 55.2 (OCH_3), 51.1 (CH_2Cl), 41.4 (CH), 19.0 (CH_3).

5-(1-Chloro-2-propanyl)-1,3-benzodioxole (34)

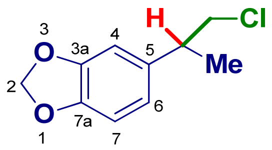

By following the **General procedure 1**, starting from 1-(1,3-benzodioxol-5-yl)ethanone (200 mg, 1.22 mmol, 1 equiv) in dry THF (3 mL), chloroiodomethane (0.13 mL, 1.8 mmol, 1.5 equiv), MeLi-LiBr 2.2 M solution in Et_2O (0.78 mL, 1.7 mmol, 1.4 equiv), tris(pentafluorophenyl)borane (63 mg, 0.12 mmol, 0.1 equiv) and hexylsilane (0.2 mL, 1.22 mmol, 1 equiv), **compound 34** was obtained in 85 % yield (206 mg) as colorless oil after column chromatography on silica gel (*n*-hexane/dicloromethane 9:1 as eluent).

^{1}H NMR (500 MHz, $CDCl_3$) δ: 6.76 (d, 1H, $^3J_{H,H}$ = 8.0 Hz, Benz H-7), 6.71 (d, 1H, $^4J_{H,H}$ = 1.8 Hz, Benz H-4), 6.68 (dd, 1H, $^3J_{H,H}$ = 8.0 Hz, $^4J_{H,H}$ = 1.8 Hz, Benz H-6), 5.94 (s, 2H, -OCH_2O-), 3.63 (dd, 1H, $^2J_{H,H}$ = 10.7 Hz, $^3J_{H,H}$ = 6.3 Hz, CH_2), 3.54 (dd, 1H, $^2J_{H,H}$ = 10.7 Hz, $^3J_{H,H}$ = 7.7 Hz, CH_2), 3.02 (m, 1H, CH), 1.35 (d, 3H, $^3J_{H,H}$ = 7.0 Hz, CH_3).

^{13}C NMR (125 MHz, $CDCl_3$) δ: 147.7 (Benz C-3a), 146.4 (Benz C-7a), 137.2 (Benz C-5), 120.3 (Benz C-6), 108.3 (Benz C-7), 107.4 (Benz C-4), 101.0 (O-CH_2-O), 50.9 (CH_2Cl), 42.0 (CH), 19.1 (CH_3).

(1-Chloropropan-2-yl)benzene (35)

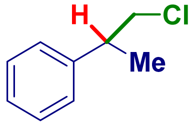

By following the **General procedure 1**, starting from acetophenone (200 mg, 1.66 mmol, 1 equiv) in dry THF (3 mL), chloroiodomethane (0.18 mL, 2.5 mmol, 1.5 equiv), MeLi-LiBr 2.2 M solution in Et_2O (1.1 mL, 2.3 mmol, 1.4 equiv), tris(pentafluorophenyl)borane (85 mg, 0.17 mmol, 0.1 equiv) and hexylsilane (0.27 mL, 1.66 mmol, 1 equiv), **compound 35** was obtained in 93 % yield (238 mg) as colorless oil after column chromatography on silica gel (*n*-hexane/diethyl ether 9:1 as eluent).

^{1}H NMR (500 MHz, $CDCl_3$) δ: 7.34 (m, 2H, Ph H-3,5), 7.26 (m, 1H, Ph H-4), 7.24 (m, 2H, Ph H-2,6), 3.70 (dd, 1H, $^2J_{H,H}$ = 10.7 Hz, $^3J_{H,H}$ = 6.1 Hz, CH_2), 3.60 (dd, 1H, $^2J_{H,H}$ = 10.7 Hz, $^3J_{H,H}$ = 7.9 Hz, CH_2), 3.11 (m, 1H, CH), 1.41 (d, 3H, $^3J_{H,H}$ = 7.0 Hz, CH_3).

^{13}C NMR (125 MHz, $CDCl_3$) δ: 143.3 (Ph C-1), 128.6 (Ph C-3,5), 127.2 (Ph C-2,6), 126.9 (Ph C-4), 50.8 (CH_2Cl), 42.3 (CH), 19.0 (CH_3).

(1-Chloro-2-butanyl)benzene (36)

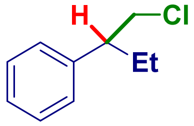

By following the **General procedure 1**, starting from propiophenone (200 mg, 1.49 mmol, 1 equiv) in dry THF (3 mL), chloroiodomethane (0.16 mL, 2.2 mmol, 1.5 equiv) , MeLi-LiBr 2.2 M solution in Et_2O (1.0 mL, 2.1 mmol, 1.4 equiv), tris(pentafluorophenyl)borane (76 mg, 0.15 mmol, 0.1 equiv) and hexylsilane (0.24 mL,

1.49 mmol, 1 equiv), **compound 36** was obtained in 89 % yield (224 mg) as colorless oil after column chromatography on silica gel (*n*-hexane as eluent).

^{1}H NMR (500 MHz, $CDCl_3$) δ: 7.33 (m, 2H, Ph H-3,5), 7.25 (m, Ph H-4), 7.19 (m, 2H, Ph H-2,6), 3.69 (d, 2H, $^3J_{H,H}$ = 6.9 Hz, CH_2Cl), 2.82 (m, 1H, CH), 1.96 (m, 1H, C$\underline{H}_2CH_3$), 1.64 (m, 1H, C$\underline{H}_2CH_3$), 0.83 (t, 3H, $^3J_{H,H}$ = 7.4 Hz, CH_3).

^{13}C NMR (125 MHz, $CDCl_3$) δ: 141.9 (Ph C-1), 128.5 (Ph C-3,5), 127.8 (Ph C-2,6), 126.9 (Ph C-4), 49.9 (CH), 49.5 (CH_2Cl), 26.1 ($\underline{C}H_2CH_3$), 11.8 (CH_3).

EI-MS m/z (%): 168.1 (M^+, 21), 91.1 (M^+, 100).

(1-Chloro-2-pentanyl)benzene (37)

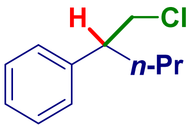

By following the **general procedure 1**, starting from 1-phenylbutan-1-one (200 mg, 1.35 mmol, 1 equiv) in dry THF (3 mL), chloroiodomethane (0.15 mL, 2.0 mmol, 1.5 equiv), MeLi-LiBr 2.2 M solution in Et_2O (0.9 mL, 1.9 mmol, 1.4 equiv), tris(pentafluorophenyl)borane (69 mg, 0.14 mmol, 0.1 equiv) and hexylsilane (0.22 mL, 1.35 mmol, 1 equiv), **compound 37** was obtained in 92 % yield (227 mg) as colorless oil after column chromatography on silica gel (*n*-hexane as eluent).

^{1}H NMR (500 MHz, $CDCl_3$) δ: 7.33 (m, 2H, Ph H-3,5), 7.25 (m, Ph H-4), 7.19 (m, 2H, Ph H-2,6), 3.67 (d, 2H, $^3J_{H,H}$ = 6.9 Hz, CH_2Cl), 2.91 (m, 1H, CH), 1.86 (m, 1H, H-3a), 1.61 (m, 1H, H-3b), 1.21 (m, 2H, H-4), 0.88 (t, 3H, $^3J_{H,H}$ = 7.3 Hz, CH_3).

^{13}C NMR (125 MHz, $CDCl_3$) δ: 142.1 (Ph C-1), 128.5 (Ph C-3,5), 127.8 (Ph C-2,6), 126.9 (Ph C-4), 49.7 (CH_2Cl), 48.0 (CH), 35.4 (C-3), 20.4 (C-4), 14.0 (CH_3).

EI-MS m/z (%): 182.1 (M^+, 10), 91.1 (M^+, 100).

(1-Chloro-2-hexanyl)benzene (38)

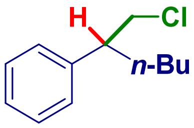

By following the **General procedure 1**, starting from 1-phenylbutan-1-one (200 mg, 1.23 mmol, 1 equiv) in dry THF (3 mL), chloroiodomethane (0.14 mL, 1.85 mmol, 1.5 equiv) , MeLi-LiBr 2.2 M solution in Et_2O (0.8 mL, 1.7 mmol, 1.4 equiv), tris(pentafluorophenyl)borane (63 mg, 0.12 mmol, 0.1 equiv) and hexylsilane (0.2 mL, 1.23 mmol, 1 equiv), **compound 38** was obtained in 87% yield (196 mg) as colorless oil after column chromatography on silica gel (*n*-hexane as eluent).

^{1}H NMR (500 MHz, $CDCl_3$) δ: 7.33 (m, 2H, Ph H-3,5), 7.25 (m, Ph H-4), 7.19 (m, 2H, Ph H-2,6), 3.67 (d, 2H, $^3J_{H,H}$ = 6.9 Hz, H-1), 2.89 (m, 1H, CH), 1.90 (m, 1H, H-3a), 1.62 (m, 1H, H-3b), 1.16 (m, 2H, H-4), 1.27 (m, 2H, H-5), 0.84 (t, 3H, $^3J_{H,H}$ = 7.2 Hz, CH_3).

^{13}C NMR (125 MHz, $CDCl_3$) δ: 142.2 (Ph C-1), 128.5 (Ph C-3,5), 127.8 (Ph C-2,6), 126.9 (Ph C-4), 49.8 (CH_2Cl), 48.2 (CH), 32.9 (C-3), 29.4 (C-4), 22.6 (C-5), 13.9 (CH_3).

EI-MS m/z (%): 196.1 (M^+, 13), 91.1 (M^+, 100).

(1,4-Dichloro-2-butanyl)benzene (39)

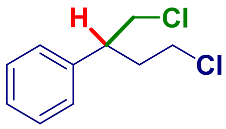

By following the **General procedure 1**, starting from 4-chloro-1-phenylbutan-1-one (200 mg, 1.19 mmol, 1 equiv) in dry THF (3 mL), chloroiodomethane (0.13 mL, 1.8 mmol, 1.5 equiv), MeLi-LiBr 2.2 M solution in Et_2O (0.8 mL, 1.7 mmol, 1.4 equiv), tris(pentafluorophenyl)borane (61 mg, 0.12 mmol, 0.1 equiv) and hexylsilane (0.2 mL, 1.19 mmol, 1 equiv), **compound 39** was obtained in 91 % yield (219 mg) as colorless oil after column chromatography on silica gel (*n*-hexane/diethyl ether 95:5 as eluent).

^{1}H NMR (500 MHz, $CDCl_3$) δ: 7.36 (m, 2H, Ph H-3,5), 7.29 (m, 1H, Ph H-4), 7.22 (m, Ph, H-2,6), 3.73 (dd, 1H, $^2J_{H,H}$ = 10.9 Hz, $^3J_{H,H}$ = 6.4 Hz, H-1a), 3.69 (dd, 1H, $^2J_{H,H}$ = 10.9 Hz, $^3J_{H,H}$ = 7.3 Hz, H-1b), 3.49 (m, 1H, H-4a), 3.28 (m, 1H, H-4b), 3.23 (m, 1H, CH), 2.40 (m, 1H, H-3a), 2.09 (m, 1H, H-3b).

^{13}C NMR (125 MHz, $CDCl_3$) δ: 140.1 (Ph C-1), 128.9 (Ph C-3,5), 127.8 (Ph C-2,6), 127.5 (Ph C-4), 48.9 (C-1), 45.1 (CH), 42.5 (C-4), 35.7 (C-3).

EI-MS m/z (%): 202.0 (M^+, 22), 91.1 (M^+, 100).

1,1'-(3-Chloro-1,2-propanediyl)dibenzene (40)

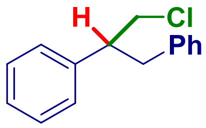

By following the **General procedure 1**, starting from 1,2-diphenylethanone (200 mg, 1.02 mmol, 1 equiv) in dry THF (3 mL), chloroiodomethane (0.11 mL, 1.5 mmol, 1.5 equiv) , MeLi-LiBr 2.2 M solution in Et_2O (0.7 mL, 1.4 mmol, 1.4 equiv), tris(pentafluorophenyl)borane (52 mg, 0.1 mmol, 0.1 equiv) and hexylsilane (0.2 mL, 1.02 mmol, 1 equiv), **compound 40** was obtained in 90 % yield (212 mg) as colorless oil after column chromatography on silica gel (*n*-hexane/dichloromethane 9:1 as eluent).

^{1}H NMR (500 MHz, $CDCl_3$) δ: 7.32 (m, 2H, Ph H-3,5), 7.26 (m, 1H, Ph H-4), 7.24 (m, 2H, Bn H-3,5), 7.19 (m, 1H, Bn H-4), 7.18 (m, 2H, Ph H-2,6), 7.09 (m, 2H, Bn H-2,6), 3.73 (m, 2H, CH_2Cl), 3.25 (m, 1H, CH), 3.20 (m, 1H, CH_2Ph), 2.98 (dd, 1H, $^2J_{H,H}$ = 13.1 Hz, $^3J_{H,H}$ = 7.0 Hz, CH_2Ph).

^{13}C NMR (125 MHz, $CDCl_3$) δ: 141.4 (Ph C-1), 139.1 (Bn C-1), 129.1 (Bn C-2,6), 128.4 (Ph C-3,5), 128.3 (Bn C-3,5), 127.8 (Ph C-2,6), 127.0 (Ph C-4), 126.2 (Bn C-4), 49.6 (CH), 48.5 (CH_2Cl), 39.5 (CH_2Ph).

EI-MS m/z (%): 230.1 (M^+, 14), 104.1 (M^+, 100).

(2-Chloro-1-cyclohexylethyl)benzene (41)

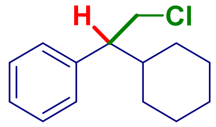

By following the **General procedure 1**, starting from cyclohexyl(phenyl)methanone (200 mg, 1.06 mmol, 1 equiv) in dry THF (3 mL), chloroiodomethane (0.12 mL, 1.6 mmol, 1.5 equiv), MeLi-LiBr 2.2 M solution in Et_2O (0.7 mL, 1.5 mmol, 1.4 equiv), tris(pentafluorophenyl)borane (54 mg, 0.11 mmol, 0.1 equiv) and hexylsilane (0.2 mL, 1.06 mmol, 1 equiv), **compound 41** was obtained in 86% yield (203 mg) as colorless oil after column chromatography on silica gel (*n*-hexane as eluent).

^{1}H NMR (400 MHz, $CDCl_3$) δ: 7.32(m, 2H, Ph H-3,5), 7.26 (m, 1H, Ph H-4), 7.17 (m, 2H, Ph H-2,6), 3.90 (m, 1H, CH_2Cl) 3.80 (m, 1H, CH_2Cl), 2.73 (m, 1H, Ph-C̲H), 1.74 (m, 1H, Cyclo H-1), 1.89-0.70 (m, 10H, Cyclo H-2,3,4,5,6).

^{13}C NMR (100 MHz, $CDCl_3$) δ: 141.3 (Ph C-1), 128.5 (Ph C-2,6), 128.1 (Ph C-3,5), 126.7 (Ph C-4), 53.9 (Ph-C̲H), 47.5 (CH_2Cl), 40.5 (Cyclo C-1), 31.2, 30.5, 26.3 (Cyclo C-2,3,4,5,6).

EI-MS m/z (%): 222.1(M^+, 7), 104.1 (M^+, 100).

4-(1-Chloro-2-propanyl)-5-methyl-1-phenyl-1*H*-pyrazole (42)

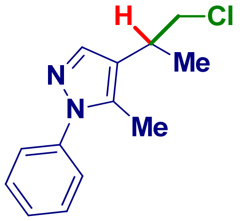

By following the **General procedure 1**, starting from 1-(5-methyl-1-phenyl-1H-pyrazol-4-yl)ethan-1-one (200 mg, 0.72 mmol, 1 equiv) in dry THF (3 mL), chloroiodomethane (0.07 mL, 1.1 mmol, 1.5 equiv), MeLi-LiBr 2.2 M solution in Et_2O (0.5 mL, 1.0 mmol, 1.4 equiv), tris(pentafluorophenyl)borane (37 mg, 0.1 mmol, 0.1 equiv) and hexylsilane (0.12 mL, 0.72 mmol, 1 equiv), **compound 42** was obtained in 85 % yield (203 mg) as colorless oil after column chromatography on silica gel (*n*-hexane/ethyl acetate 7:3 as eluent).

^{1}H NMR (400 MHz, $CDCl_3$) δ: 7.53 (s, 1H, Pyr H-3), 7.47 (m, 2H, Ph H-3,5), 7.43 (m, 2H, Ph H-2,6), 7.38 (m, 1H, Ph H-4), 3.64 (A-part of AB system, $^2J_{H,H}$ = 10.6 Hz, $^3J_{H,H}$ = 5.9 Hz, 1H, CH_2), 3.56 (B-part of AB system, $^2J_{H,H}$ = 10.6 Hz, $^3J_{H,H}$ = 7.8 Hz, 1H, CH_2), 3.05 (m, 1H, CH), 2.28 (s, 3H, Pyr-C$\underline{H_3}$), 1.41 (d, 3H, $^3J_{H,H}$ = 7.0 Hz, CH_3).

^{13}C NMR (100 MHz, $CDCl_3$) δ: 139.9 (Ph C-1), 137.8 (Pyr C-3), 135.5 (Pyr C-5), 129.0 (Ph C-3,5), 127.6 (Ph C-4), 125.0 (Ph C-2,6), 121.8 (Pyr C-4), 50.7 (CH_2Cl), 32.7 (CH), 18.9 (CH_3), 10.9 (Pyr-C̲H_3).

HRMS (ESI), *m/z*: calcd. for $C_{13}H_{16}ClN_2^+$: 235.0997 $[M+H]^+$; found: 235.0995.

(4-Chloro-3-methyl-1-butyn-1-yl)benzene (43)

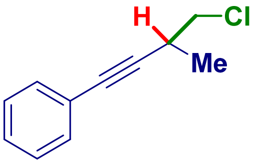

By following the **General procedure 1**, starting from (200 mg, 1.39 mmol, 1 equiv) in dry THF (3 mL), chloroiodomethane (0.14 mL, 2.1 mmol, 1.5 equiv), MeLi-LiBr 2.2 M solution in Et_2O (0.9 mL, 2.0 mmol, 1.4 equiv), tris(pentafluorophenyl)borane (71 mg, 0.14 mmol, 0.1 equiv) and hexylsilane (0.23 mL, 1.39 mmol, 1 equiv), **compound 43** was obtained in 95 % yield (236 mg) as colorless oil after column chromatography on silica gel (cyclohexane as eluent).

^{1}H NMR (500 MHz, $CDCl_3$) δ: 7.42 (m, 2H, Ph H-2,6), 7.29 (m, 3H, Ph H-3,4,5), 3.70 (dd, 1H, $^2J_{H,H}$ = 10.6 Hz, $^3J_{H,H}$ = 5.6 Hz, CH_2), 3.54 (dd, 1H, $^2J_{H,H}$ = 10.6 Hz, $^3J_{H,H}$ = 7.5 Hz, CH_2), 3.03 (m, 1H, CH), 1.39 (d, 3H, $^3J_{H,H}$ = 6.9 Hz, CH_3).

^{13}C NMR (125 MHz, $CDCl_3$) δ: 131.7 (Ph C-2,6), 128.2 (Ph C-3,5), 128.0 (Ph C-4), 123.1 (Ph C-1), 90.3 (C-2), 82.3 (C-1), 48.7 (CH_2Cl), 29.6 (CH), 18.6 (CH_3).

1,1'-(2-Chloro-1,1-ethanediyl)dibenzene (44)

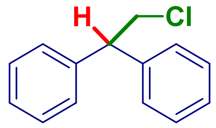

By following the **General procedure 1**, starting from benzophenone (200 mg, 1.1 mmol, 1 equiv) in dry THF (3 mL), chloroiodomethane (0.12 mL, 1.7 mmol, 1.5 equiv), MeLi-LiBr 2.2 M solution in Et_2O (0.7 mL, 1.5 mmol, 1.4 equiv), tris(pentafluorophenyl)borane (56 mg, 0.11 mmol, 0.1 equiv) and hexylsilane (0.18 mL, 1.1 mmol, 1 equiv), **compound 44** was obtained in 90% yield (214 mg) as colorless oil after column chromatography on silica gel (*n*-hexane/chloroform 1:1 as eluent).

^{1}H NMR (500 MHz, $CDCl_3$) δ: 7.34 (m, 4H, Ph H-3,5), 7.27 (m, 4H, Ph H-2,6), 7.26 (m, 2H, Ph H-4), 4.36 (t, 1H, $^3J_{H,H}$ = 7.8 Hz, CH), 4.09 (d, 2H, $^3J_{H,H}$= 7.8 Hz, CH_2Cl).

^{13}C NMR (125 MHz, $CDCl_3$) δ: 141.2 (Ph C-1), 128.6 (Ph C-3,5) 128.0 (Ph C-2,6), 127.0 (Ph C-4), 53.6 (CH), 47.2 (CH_2Cl).

1-(2-Chloro-1-phenylethyl)-4-methylbenzene (45)

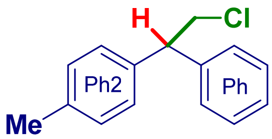

By following the **General procedure 1**, starting from phenyl(*p*-tolyl)methanone (200 mg, 1.02 mmol, 1 equiv) in dry THF (3 mL), chloroiodomethane (0.11 mL, 1.5 mmol, 1.5 equiv), MeLi-LiBr 2.2 M solution in Et_2O (0.65 mL, 1.4 mmol, 1.4 equiv), tris(pentafluorophenyl)borane (52 mg, 0.11 mmol, 0.1 equiv) and hexylsilane (0.17 mL, 1.02 mmol, 1 equiv), **compound 45** was obtained in 86% yield (201 mg) as colorless oil after column chromatography on silica gel (*n*-hexane/dichloromethane 9:1 as eluent).

^{1}H NMR (500 MHz, $CDCl_3$) δ: 7.32 (m, 2H, Ph H-3,5), 7.24 (m, 2H, Ph H-2,6), 7.23 (m, 1H, Ph H-4), 7.14 ('s', 4H, Ph2 H-2,3,5,6), 4.30 (t, 1H, $^3J_{H,H}$= 7.8 Hz, CH), 4.05 (d, 2H, $^3J_{H,H}$= 7.8 Hz, CH_2Cl), 2.32 (s, 3H, CH_3).

^{13}C NMR (125 MHz, $CDCl_3$) δ: 141.5 (Ph C-1), 138.3 (Ph2 C-1), 136.7 (Ph2 C-4), 129.4 (Ph2 C-3,5), 128.6 (Ph C-3,5), 128.0 (Ph C-2,6), 127.9 (Ph2 C-2,6), 126.9 (Ph C-4), 53.2 (CH), 47.3 (CH_2Cl), 21.0 (CH_3).

EI-MS m/z (%): 230.1(M^+, 13), 181.1 (M^+, 100).

1-(2-Chloro-1-phenylethyl)-4-ethylbenzene (46)

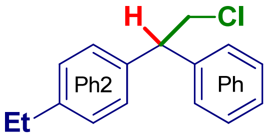

By following the **General procedure 1**, starting from (4-ethylphenyl)(phenyl)methanone (200 mg, 0.95 mmol, 1 equiv) in dry THF (3 mL), chloroiodomethane (0.1 mL, 1.4 mmol, 1.5 equiv), MeLi-LiBr 2.2 M solution in Et_2O (0.6 mL, 1.3 mmol, 1.4 equiv), tris(pentafluorophenyl)borane (49 mg, 0.1 mmol, 0.1 equiv) and hexylsilane (0.15 mL, 0.95 mmol, 1 equiv), **compound 46** was obtained in 89% yield (207 mg) as colorless oil after column chromatography on silica gel (*n*-hexane as eluent).

^{1}H NMR (500 MHz, $CDCl_3$) δ: 7.35 (m, 2H, Ph H-3,5), 7.28 (m, 2H, Ph H-2,6), 7.26 (m, 1H, Ph H-4), 7.18 (m, 4H, Ph2 H-2,3,5,6), 4.34 (t, 1H, $^3J_{H,H}$= 7.8 Hz, CH), 4.08 (d, 2H, $^3J_{H,H}$= 7.8 Hz, CH_2Cl), 2.65 (q, 2H, $^3J_{H,H}$= 7.6 Hz, Ph2-C$\underline{H}_2$), 1.24 (t, 3H, $^3J_{H,H}$= 7.6 Hz, CH_3).

^{13}C NMR (125 MHz, $CDCl_3$) δ: 142.9 (Ph2 C-4), 141.4 (Ph C-1), 138.5 (Ph2 C-1), 128.6 (Ph C-3,5), 128.1 (Ph2 C-3,5), 128.0 (Ph C-2,6), 127.9 (Ph2 C-2,6), 126.9 (Ph C-4), 53.3 (CH), 47.3 (CH_2Cl), 28.4 (Ph2-$\underline{C}H_2$), 15.4 (CH_3).

EI-MS m/z (%): 244.1(M^+, 11), 195.1 (M^+, 100).

1-(2-chloro-1-phenylethyl)-4-(2-methyl-2-propanyl)benzene (47)

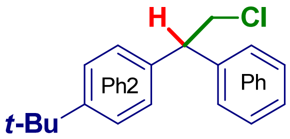

By following the **General procedure 1**, starting from (4-(tert-butyl)phenyl)(phenyl)methanone (200 mg, 0.56 mmol, 1 equiv) in dry THF (3 mL), chloroiodomethane (0.1 mL, 0,9 mmol, 1.5 equiv), MeLi-LiBr 2.2 M solution in Et_2O (0.4 mL, 0.8 mmol, 1.4 equiv), tris(pentafluorophenyl)borane (29 mg, 0.06 mmol, 0.1 equiv) and hexylsilane (0.1 mL, 0.56 mmol, 1 equiv), **compound 47** was obtained in 84% yield (184 mg) as colorless oil after column chromatography on silica gel (*n*-hexane as eluent).

^{1}H NMR (500 MHz, $CDCl_3$) δ: 7.29 (m, 4H, Ph2 H-3,5, Ph H-2,6), 7.28 (m, 2H, Ph H-3,5), 7.26 (m, 2H, Ph H-2,6), 7.24 (m, 1H, Ph H-4), 7.17 (m, 2H, Ph2 H-3,5), 4.26 (t, 1H, $^3J_{H,H}$= 7.8 Hz, CH), 4.01 (d, 2H, $^3J_{H,H}$= 7.8 Hz, CH_2), 1.24 (s, 9H, CCH_3).

^{13}C NMR (125 MHz, $CDCl_3$) δ: 149.8 (Ph2 C-1), 141.4 (Ph C-1), 138.2 (Ph2 C-4), 128.6 (Ph C-3,5), 128.0 (Ph C-2,6), 127.5 (Ph2 C-3,5), 126.9 (Ph C-4), 125.6 (Ph2 C-2,6), 53.2 (CH), 47.3 (CH_2Cl), 34.4 [$\underline{C}(CH_3)_3$], 31.3 [$C(\underline{C}H_3)_3$].

1-[4-(2-Chloro-1-phenylethyl)phenyl]adamantane (48)

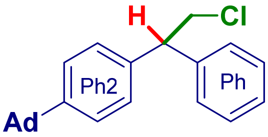

By following the **General procedure 1**, starting from (4-(adamantan-1-yl)phenyl)(phenyl)methanone (200 mg, 0.63 mmol, 1 equiv) in dry THF (3 mL), chloroiodomethane (0.1 mL, 1.0 mmol, 1.5 equiv), MeLi-LiBr 2.2 M solution in Et_2O (0.4 mL, 0.9 mmol, 1.4 equiv), tris(pentafluorophenyl)borane (32 mg, 0.06 mmol, 0.1 equiv) and hexylsilane (0.10 mL, 0.63 mmol, 1 equiv), **compound 48** was obtained in 91 % yield (201 mg) as colorless oil after column chromatography on silica gel (*n*-hexane/diethyl ether 98:2 as eluent).

^{1}H NMR (400 MHz, $CDCl_3$) δ: 7.33 (m, 2H, Ph H-3,5), 7.30 (m, 2H, Ph2 H-2,6), 7.27 (m, 2H, Ph H-2,6), 7.24 (m, 1H, Ph H-4), 7.18 (m, 1H, Ph2 H-3,5), 4.31 (t, $^3J_{H,H}$ = 7.8 Hz, 1H, CH), 4.07 (d, $^3J_{H,H}$ = 7.8 Hz, 2H, CH_2Cl), 2.08 - 1.82 (15H, Ad).

^{13}C NMR (100 MHz, $CDCl_3$) δ: 150.1 (Ph2 C-1), 141.4 (Ph C-1), 138.3 (Ph2 C-4), 128.6 (Ph C-3,5), 128.1 (Ph C-2,6), 127.6 (Ph2 C-3,5), 126.9 (Ph C-4), 125.1 (Ph2 C-2,6), 53.3 (CH), 47.4 (CH_2Cl), 43.1 (Ad C-2,8,9), 36.8 (Ad C-4,6,10), 35.9 (Ad C-1), 28.9 (Ad C-3,5,7).

EI-MS m/z (%): 350.2 (M^+, 11), 301.2 (M^+, 100).

4-(2-Chloro-1-phenylethyl)biphenyl (49)

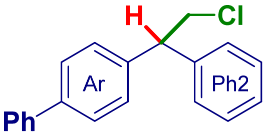

By following the **General procedure 1**, starting from [1,1'-biphenyl]-4-yl(phenyl)methanone (200 mg, 0.77 mmol, 1 equiv) in dry THF (3 mL), chloroiodomethane (0.1 mL, 1.2 mmol, 1.5 equiv), MeLi-LiBr 2.2 M solution in Et_2O (0.5 mL, 1.1 mmol, 1.4 equiv), tris(pentafluorophenyl)borane (39 mg, 0.08 mmol, 0.1 equiv) and hexylsilane (0.13 mL, 0.77 mmol, 1 equiv), **compound 49** was obtained in 87% yield (196 mg) as colorless oil after column chromatography on silica gel (*n*-hexane as eluent).

^{1}H NMR (500 MHz, $CDCl_3$) δ: 7.57 (m, 2H, Ph H-2,6), 7.56 (m, 2H, Ar H-2,6), 7.43 (m, 2H, Ph H-3,5), 7.35 (m, 2H, Ph2 H-3,5), 7.34 (m, 1H, Ph H-4), 7.33 (m, 2H, Ar H-3,5), 7.29 (m, 2H, Ph2 H-2,6), 7.27 (m, 1H, Ph2 H-4), 4.40 (t, 1H, $^3J_{H,H}$= 7.8 Hz, CH), 4.11 (d, 2H, $^3J_{H,H}$= 7.8 Hz, CH_2Cl).

^{13}C NMR (125 MHz, $CDCl_3$) δ: 141.2 (Ph2 C-1), 140.6 (Ph C-1), 140.3 (Ar C-4), 139.9 (Ar C-1), 128.7 (Ph C-3,5, Ph2 C-3,5), 128.4 (Ar C-3,5), 128.0 (Ph2 C-2,6), 127.4 (Ar C-2,6), 127.3 (Ph C-4), 127.1 (Ph2 C-4), 127.0 (Ph C-2,6), 53.3 (CH), 47.1 (CH_2Cl).

1-(2-Chloro-1-phenylethyl)-3-methoxybenzene (50)

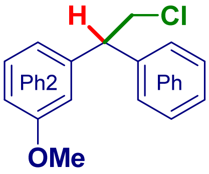

By following the **General procedure 1**, starting from 1-(3-methoxyphenyl)ethanone (200 mg, 0.94 mmol, 1 equiv) in dry THF (3 mL), chloroiodomethane (0.1 mL, 1.4 mmol, 1.5 equiv), MeLi-LiBr 2.2 M solution in THF (0.6 mL, 1.4 mmol, 1.4 equiv), tris(pentafluorophenyl)borane (48 mg, 0.1 mmol, 0.1 equiv) and hexylsilane (0.15 mL, 0.94 mmol, 1 equiv), **compound 50** was obtained in 90 % yield (156 mg) as colorless oil after column chromatography on silica gel (*n*-hexane as eluent).

^{1}H NMR (500 MHz, $CDCl_3$) δ: 7.33 (m, 2H, Ph H-3,5), 7.26 (m, 2H, Ph H-2,6), 7.25 (m, 2H, Ph H-4, Ph2 H-5), 6.85 (m, 1H, Ph2 H-6), 6.79 (s, 1H, Ph2 H-2), 6.78 (m, 1H, Ph2 H-4), 4.31 (t, 1H, $^3J_{H,H}$ = 7.8 Hz, CH), 4.06 (d, 2H, $^3J_{H,H}$ = 7.8 Hz, CH_2Cl), 3.78 (s, 3H, CH_3).

^{13}C NMR (125 MHz, $CDCl_3$) δ: 159.7 (Ph2 C-3), 142.8 (Ph2 C-1), 141.1 (Ph C-1), 129.6 (Ph2 C-5), 128.7 (Ph C-3,5), 128.0 (Ph C-2,6), 127.1 (Ph C-4), 120.3 (Ph2 C-6), 114.3 (Ph2 C-2), 111.9 (Ph2 C-4), 55.2 (OCH_3), 53.6 (CH), 47.1 (CH_2Cl).

EI-MS m/z (%): 246.1 (M^+, 31), 197.1 (M^+, 100).

1-(2-Chloro-1-phenylethyl)-4-(methylsulfanyl)benzene (51)

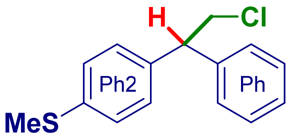

By following the **General procedure 1**, starting from (4-(methylthio)phenyl)(phenyl)methanone (200 mg, 0.88 mmol, 1 equiv) in dry THF (3 mL), chloroiodomethane (0.1 mL, 1.3 mmol, 1.5 equiv), MeLi-LiBr 2.2 M solution in Et_2O (0.6 mL, 1.2 mmol, 1.4 equiv), tris(pentafluorophenyl)borane (45 mg, 0.1 mmol, 0.1 equiv) and hexylsilane (0.14 mL, 0.63 mmol, 1 equiv), **compound 51** was obtained in 87 % yield (201 mg) as colorless oil after column chromatography on silica gel (*n*-hexane/diethyl ether 98:2 as eluent).

^{1}H NMR (400 MHz, C_6D_6) δ: 7.08 (m, 2H, Ph H-3,5), 7.02 (m, 2H, Ph2 H-3,5), 7.01 (m, 1H, Ph H-4), 6.96 (m, 2H, Ph H-2,6), 6.83 (m, 2H, Ph2 H-2,6), 4.04 (t, $^3J_{H,H}$ = 7.7 Hz, 1H, CH), 3.67 (m, 2H, CH_2Cl), 1.96 (s, 3H, SCH_3).

^{13}C NMR (100 MHz, C_6D_6) δ: 141.8 (Ph C-1), 138.5 (Ph2 C-1), 137.7 (Ph2 C-4), 128.9 (Ph2 C-2,6), 128.8 (Ph C-3,5), 128.4 (Ph C-2,6), 127.1 (Ph2 C-3,5), 53.2 (CH), 47.1 (CH_2Cl).

EI-MS m/z (%): 262.1 (M^+, 25), 213.1 (M^+, 100).

1-(2-Chloro-1-phenylethyl)-4-(phenylselanyl)benzene (52)

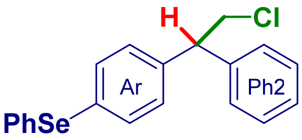

By following the **General procedure 1**, starting from phenyl(4-(phenylselanyl)phenyl)methanone (200 mg, 0.59 mmol, 1 equiv) in dry THF (3 mL), chloroiodomethane (0.7 mL, 0.9 mmol, 1.5 equiv), MeLi-LiBr 2.2 M solution in Et_2O (0.4 mL, 0.8 mmol, 1.4 equiv), tris(pentafluorophenyl)borane (31 mg, 0.06 mmol, 0.1 equiv) and hexylsilane (0.1 mL, 0.59 mmol, 1 equiv), **compound 52** was obtained in 86 % yield (189 mg) as colorless oil after column chromatography on silica gel (*n*-hexane/dichloromethane 9:1 as eluent).

^{1}H NMR (500 MHz, $CDCl_3$) δ: 7.48 (m, 2H, Ph H-2,6), 7.39 (m, 2H, Ar H-3,5), 7.33 (m, 2H, Ph2 H-3,5), 7.28 (m, 3H, Ph H-3,4,5), 7.25 (m, 1H, Ph2 H-4), 7.23 (m, 2H, Ph2 H-2,6), 7.15 (m, 2H, Ar H-2,6), 4.30 (t, 1H, $^3J_{H,H}$ = 7.8 Hz, CH), 4.04 (d, 2H, $^3J_{H,H}$ = 7.8 Hz, CH_2Cl).

^{13}C NMR (125 MHz, $CDCl_3$) δ: 140.9 (Ph2 C-1), 140.4 (Ar C-1), 133.3 (Ph C-2,6), 132.8 (Ar C-3,5), 130.6 (Ph C-1), 129.4 (Ph C-3,5), 129.0 (Ar C-2,6), 128.7 (Ph2 C-3,5), 128.0 (Ph2 C-2,6), 127.5 (Ph C-4), 127.2 (Ph2 C-4), 53.2 (CH), 47.0 (CH_2Cl).

EI-MS m/z (%): 372.1 (M^+, 54), 323.1 (M^+, 100).

1-(2-Chloro-1-phenylethyl)-4-iodobenzene (53)

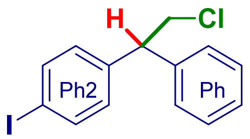

By following the **General procedure 1**, starting from (4-iodophenyl)(phenyl)methanone (200 mg, 0.65 mmol, 1 equiv) in dry THF (3 mL), chloroiodomethane (0.07 mL, 1.0 mmol, 1.5 equiv) , MeLi-LiBr 2.2 M solution in Et_2O (0.4 mL, 0.9 mmol, 1.4 equiv), tris(pentafluorophenyl)borane (33 mg, 0.07 mmol, 0.1 equiv) and hexylsilane (0.10 mL, 0.65 mmol, 1 equiv), **compound 53** was obtained in 93 % yield (207 mg) as colorless oil after column chromatography on silica gel (*n*-hexane/dichloromethane 9:1 as eluent).

^{1}H NMR (200 MHz, $CDCl_3$) δ: 7.66 (m, 2H, Ph2 H-3,5), 7.34 (m, 2H, Ph H-3,5), 7.26 (m, 1H, Ph H-4), 7.22 (m, 2H, Ph H-2,6), 7.02 (m, 2H, Ph2 H-2,6), 4.31 (t, 1H, $^3J_{H,H}$ = 7.8 Hz, CH), 4.04 (m, 2H, CH_2Cl).

^{13}C NMR (125 MHz, $CDCl_3$) δ: 140.9 (Ph C-1), 140.7 (Ph2 C-1), 137.7 (Ph2 C-3,5), 130.1 (Ph2 C-2,6), 128.8 (Ph C-3,5), 127.9 (Ph C-2,6), 127.2 (Ph C-4), 92.5 (Ph2 C-4), 53.1 (CH), 46.7 (CH_2Cl).

EI-MS m/z (%): 342.0(M^+, 19), 293.0 (M^+, 100).

1-Bromo-3-(2-chloro-1-phenylethyl)benzene (54)

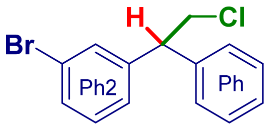

By following the **General procedure 1**, starting from (3-bromophenyl)(phenyl)methanone (200 mg, 0.77 mmol, 1 equiv) in dry THF (3 mL), chloroiodomethane (0.08 mL, 1.2 mmol, 1.5 equiv), MeLi-LiBr 2.2 M solution in Et_2O (0.5 mL, 1.1 mmol, 1.4 equiv), tris(pentafluorophenyl)borane (39 mg, 0.08 mmol, 0.1 equiv) and hexylsilane (0.13 mL, 0.77 mmol, 1 equiv), **compound 54** was obtained in 90 % yield (205 mg) as colorless oil after column chromatography on silica gel (*n*-hexane/dichloromethane 9:1 as eluent).

^{1}H NMR (500 MHz, $CDCl_3$) δ: 7.42 (m, 1H, Ph2 H-2), 7.40 (m, 1H, Ph2 H-6), 7.35 (m, 2H, Ph H-3,5), 7.28 (m, 1H, Ph H-4), 7.24 (m, 2H, Ph H-2,6), 7.21 (m, 2H, Ph2 H-4,5), 4.32 (t, 1H, $^3J_{H,H}$ = 7.8 Hz, CH), 4.05 (d, 2H, $^3J_{H,H}$ = 7.8 Hz, CH_2Cl).

^{13}C NMR (125 MHz, $CDCl_3$) δ: 143.5 (Ph2 C-3), 140.5 (Ph C-1), 131.1 (Ph2 C-2), 130.2 (Ph2 C-5,6), 128.8 (Ph C-3,5), 127.9 (Ph C-2,6), 127.3 (Ph C-4), 126.7 (Ph2 C-4), 122.7 (Ph2 C-1), 53.2 (CH), 46.7 (CH_2Cl).

EI-MS m/z (%): 294.0 (M^+, 19), 245.0 (M^+, 100).

1-Chloro-3-(2-chloro-1-phenylethyl)benzene (55)

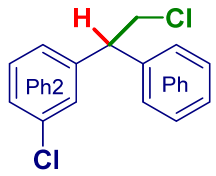

By following the **General procedure 1**, starting from (3-chlorophenyl)(phenyl)methanone (200 mg, 0.92 mmol, 1 equiv) in dry THF (3 mL), chloroiodomethane (0.10 mL, 1.4 mmol, 1.5 equiv), MeLi-LiBr 2.2 M solution in Et_2O (0.6 mL, 1.3 mmol, 1.4 equiv), tris(pentafluorophenyl)borane (47 mg, 0.1 mmol, 0.1 equiv) and hexylsilane (0.15 mL, 0.92 mmol, 1 equiv), **compound 55** was obtained in 91 % yield (210 mg) as colorless oil after column chromatography on silica gel (*n*-hexane/dichloromethane 9:1 as eluent).

^{1}H NMR (500 MHz, $CDCl_3$) δ: 7.34 (m, 2H, Ph H-3,5), 7.26 (m, 2H; Ph H-4, Ph2 H-4), 7.25 (m, 1H, Ph2 H-5), 7.23 (m, 1H, Ph2 H-2), 7.22 (m, 2H, Ph H-2,6), 7.15 (m, 1H, Ph2 H-4), 4.31 (t, 1H, $^3J_{H,H}$ = 7.7 Hz, CH), 4.04 (d, 2H, $^3J_{H,H}$ = 7.7 Hz, CH_2Cl).

^{13}C NMR (125 MHz, $CDCl_3$) δ: 143.2 (Ph2 C-3), 140.6 (Ph C-1), 134,5 (Ph2 C-1), 128.9 (Ph2 C-5), 128.8 (Ph C-3,5), 128.2 (Ph2 C-2), 127.9 (Ph C-2,6), 127.3 (Ph C-4), 126.2 (Ph2 C-4), 53.3 (CH), 46.8 (CH_2Cl).

EI-MS m/z (%): 250.0 (M^+, 20), 201.0 (M^+, 100).

1-(2-Chloro-1-phenylethyl)-4-fluorobenzene (56)

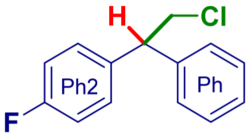

By following the **General procedure 1**, starting from (4-fluorophenyl)(phenyl)methanone (200 mg, 1.00 mmol, 1 equiv) in dry THF (3 mL), chloroiodomethane (0.11 mL, 1.5 mmol, 1.5 equiv), MeLi-LiBr 2.2 M solution in Et_2O (0.64 mL, 1.4 mmol, 1.4 equiv), tris(pentafluorophenyl)borane (51 mg, 0.1 mmol, 0.1 equiv) and hexylsilane (0.16 mL, 1.00 mmol, 1 equiv), **compound 56** was obtained in 85 % yield (199 mg) as colorless oil after column chromatography on silica gel (*n*-hexane/diethyl ether 95:5 as eluent).

^{1}H NMR (500 MHz, $CDCl_3$) δ: 7.35 (m, 2H, Ph H-3,5), 7.27 (m, 1H, Ph H-4), 7.24 (m, 4H, Ph H-2,6, Ph2 H-2,6), 7.03 (m, 2H, Ph2 H-3,5), 4.35 (m, 1H, CH), 4.06 (m, 2H, CH_2Cl).

^{13}C NMR (125 MHz, $CDCl_3$) δ: 161.7 (d, $^1J_{C,F}$ = 245.8 Hz, Ph2 C-4), 141.1 (Ph C-1), 136.9 (d, $^4J_{C,F}$ = 3.5 Hz, Ph2 C-1), 129.5 (d, $^3J_{C,F}$ = 8.1 Hz, Ph2 C-2,6), 128.7 (Ph C-3,5), 127.8 (Ph C-2,6), 127.1 (Ph C-4), 115.5 (d, $^2J_{C,F}$ = 21.8 Hz, Ph2 C-3,5), 52.8 (CH), 47.1 (CH_2Cl).

1-(2-Chloro-1-phenylethyl)-2-fluorobenzene (57)

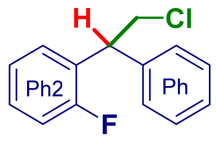

By following the **General procedure 1**, starting from (2-fluorophenyl)(phenyl)methanone (200 mg, 1.00 mmol, 1 equiv) in dry THF (3 mL), chloroiodomethane (0.11 mL, 1.5 mmol, 1.5 equiv), MeLi-LiBr 2.2 M solution in Et_2O (0.6 mL, 1.4 mmol, 1.4 equiv), tris(pentafluorophenyl)borane (51 mg, 0.1 mmol, 0.1 equiv) and hexylsilane (0.16 mL, 1.00 mmol, 1 equiv), **compound 57** was obtained in 89 % yield (208 mg) as colorless oil after column chromatography on silica gel (*n*-hexane/ethyl acetate 9:1 as eluent).

^{1}H NMR (500 MHz, $CDCl_3$) δ: 7.33 (m, 2H, Ph H-3,5), 7.28 (m, 2H, Ph H-2,6),7.26 (m, 1H, Ph H-4), 7.25 (m, 1H, Ph2 H-6), 7.24 (m, 1H, Ph2 H-4), 7.13 (m, 1H, Ph2 H-5), 7.05 (m, 1H, Ph2 H-3), 4.66 (t, 1H, $^3J_{H,H}$ = 7.9 Hz, CH), 4.12 (dd, 1H, $^2J_{H,H}$ = 11.1 Hz, $^3J_{H,H}$ = 8.1 Hz, CH_2Cl), 4.06 (dd, 1H, $^2J_{H,H}$ = 11.1 Hz, $^3J_{H,H}$ = 7.6 Hz, CH_2Cl).

^{13}C NMR (125 MHz, $CDCl_3$) δ: 160.8 (d, $^1J_{C,F}$ = 246.4 Hz, Ph2 C-2), 140.1 (Ph C-1), 128.8 (d, $^4J_{C,F}$ = 3.6 Hz, Ph2 C-6), 128.7 (Ph C-3,5, Ph2 C-4), 128.2 (d, $^2J_{C,F}$ = 14.5 Hz, Ph2 C-1), 128.0 (Ph C-2,6), 127.2 (Ph C-4), 124.2 (d, $^4J_{C,F}$ = 3.4 Hz, Ph2 C-5), 115.8 (d, $^2J_{C,F}$ = 22.9 Hz, Ph2 C-3), 46.9 (d, $^3J_{C,F}$ = 2.3 Hz, CH), 46.0 (d, $^4J_{C,F}$ = 1.5 Hz, CH_2Cl).

^{19}F NMR (470 MHz, $CDCl_3$) δ: -117.1 ppm.

EI-MS m/z (%): 234.1(M^+, 12), 185.1 (M^+, 100).

1-(2-Chloro-1-phenylethyl)-3,5-bis(trifluoromethyl)benzene (58)

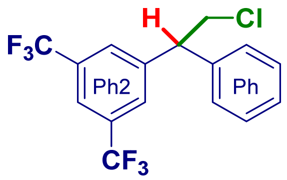

By following the **General procedure 1**, starting from (3,5-bis(trifluoromethyl)phenyl)(phenyl)methanone (200 mg, 0.63 mmol, 1 equiv) in dry THF (3 mL), chloroiodomethane (0.1 mL, 0.9 mmol, 1.5 equiv) , MeLi-LiBr 2.2 M solution in Et_2O (0.40 mL, 0.9 mmol, 1.4 equiv), tris(pentafluorophenyl)borane (32 mg, 0.06 mmol, 0.1 equiv) and hexylsilane (0.10 mL, 0.63 mmol, 1 equiv), **compound 58** was obtained in 85 % yield (189 mg) as colorless oil after column chromatography on silica gel (*n*-hexane/diethyl ether 99:1 as eluent).

^{1}H NMR (500 MHz, $CDCl_3$) δ: 7.78 (s, 1H, Ph2 H-4), 7.71 (s, 2H, Ph2 H-2,6), 7.37 (m, 2H, Ph H-3,5), 7.30 (m, 1H, Ph H-4), 7.20 (m, 2H, Ph H-2,6), 4.47 (t, $^3J_{H,H}$ = 7.7 Hz, 1H, CH), 4.09 (d, $^3J_{H,H}$ = 7.7 Hz, 2H, CH_2Cl).

^{13}C NMR (125 MHz, $CDCl_3$) δ: 143.6 (Ph2 C-1), 139.6 (Ph C-1), 129.2 (Ph C-3,5), 128.4 (Ph2 C-2,6), 127.9 (Ph C-4), 127.8 (Ph C-2,6), 121.2 (Ph2 C-4), 53.1 (CH), 46.3 (CH_2Cl), Ph2 C-3,5 not found.

^{19}F NMR (376 MHz, $CDCl_3$) δ: -62.8 (CF_3).

EI-MS m/z (%): 352.1 (M^+, 10), 303.1 (M^+, 100).

1-Azido-4-(2-chloro-1-phenylethyl)benzene (59)

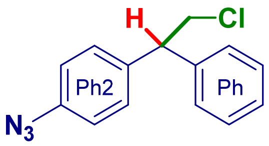

By following the **General procedure 1**, starting from (4-azidophenyl)(phenyl)methanone (200 mg, 0.89 mmol, 1 equiv) in dry THF (3 mL), chloroiodomethane (0.1 mL, 1.34 mmol, 1.5 equiv), MeLi-LiBr 2.2 M solution in Et_2O (0.6 mL, 1.3 mmol, 1.4 equiv), tris(pentafluorophenyl)borane (46 mg, 0.1 mmol, 0.1 equiv) and hexylsilane (0.15 mL, 0.89 mmol, 1 equiv), **compound 59** was obtained in 86% yield (189 mg) as colorless oil after column chromatography on silica gel (*n*-hexane/diethyl ether 9:1 as eluent).

^{1}H NMR (500 MHz, $CDCl_3$) δ: 7.33 (m, 2H, Ph H-3,5), 7.25 (m, 1H, Ph H-4), 7.24 (m, 2H, Ph2 H-3,5), 7.21 (m, 2H, Ph H-2,6), 6.99 (m, 2H, Ph2 H-2,6), 4.32 (m, 1H, CH), 4.08-4.00 (m, 2H, CH_2Cl).
^{13}C NMR (125 MHz, $CDCl_3$) δ: 141.0 (Ph C-1), 138.8 (Ph2 C-1), 138.0 (Ph2 C-4), 129.5 (Ph2 C-3,5), 128.8 (Ph C-3,5), 127.9 (Ph C-2,6), 127.2 (Ph C-4), 119.3 (Ph2 C-2,6), 52.9 (CH), 47.0 (CH_2Cl).

1,1'-(2-Chloroethane-1,1-diyl)bis(4-methoxybenzene) (60)

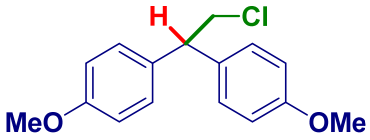

By following the **General procedure 1**, starting from bis(4-methoxyphenyl)methanone (200 mg, 0.83 mmol, 1 equiv) in dry THF (3 mL), chloroiodomethane (0.1 mL, 1.3 mmol, 1.5 equiv), MeLi-LiBr 2.2 M solution in Et_2O (0.5 mL, 1.2 mmol, 1.4 equiv), tris(pentafluorophenyl)borane (43 mg, 0.1 mmol, 0.1 equiv) and hexylsilane (0.13 mL, 0.77 mmol, 1 equiv), **compound 60** was obtained in 84 % yield (192 mg) as colorless oil after column chromatography on silica gel (*n*-hexane/ethyl acetate 8:2 as eluent).

^{1}H NMR (500 MHz, $CDCl_3$) δ: 7.15 (m, 4H, Ph H-2,6), 6.86 (m, 4H, Ph H-3,5), 4.25 (t, 1H, $^3J_{H,H}$= 7.8 Hz, CH), 4.01 (d, 2H, $^3J_{H,H}$= 7.8 Hz, CH_2Cl), 3.79 (s, 6H, OCH_3).

^{13}C NMR (125 MHz, $CDCl_3$) δ: 158.4 (Ph C-4), 133.7 (Ph C-1), 128.9 (Ph C-2,6), 114.0 (Ph C-3,5), 55.2 (OCH_3), 51.9 (CH), 47.6 (CH_2Cl).

EI-MS m/z (%): 276.1 (M^+, 10), 277.1 (M^+, 100).

1,1'-(2-Chloro-1,1-ethanediyl)bis(4-chlorobenzene) (61)

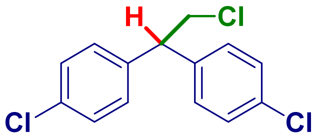

By following the **General procedure 1**, starting from (4-chlorophenyl)(phenyl)methanone (200 mg, 0.80 mmol, 1 equiv) in dry THF (3 mL), chloroiodomethane (0.1 mL, 1.2 mmol, 1.5 equiv), MeLi-LiBr 2.2 M solution in Et_2O (0.5 mL, 1.1 mmol, 1.4 equiv), tris(pentafluorophenyl)borane (41 mg, 0.1 mmol, 0.1 equiv) and hexylsilane (0.13 mL, 0.80 mmol, 1 equiv), **compound 61** was obtained in 86 % yield (173 mg) as colorless oil after column chromatography on silica gel (*n*-hexane/dichloromethane 8:2 as eluent).

^{1}H NMR (500 MHz, C_6D_6) δ: 7.03 (m, 4H, Ph H-3,5), 6.66 (m, 4H, Ph H-2,6), 3.83 (t, 1H, $^3J_{H,H}$ = 7.8 Hz, CH), 3.51 (d, 2H, $^3J_{H,H}$ = 7.8 Hz, CH_2Cl).

^{13}C NMR (125 MHz, C_6D_6) δ: 139.6 (Ph C-1), 133.3 (Ph C-4), 129.6 (Ph C-2,6), 129.1 (Ph C-3,5), 52.3 (CH), 46.6 (CH_2Cl).

EI-MS m/z (%): 284.0 (M^+, 11), 235.0 (M^+, 100).

1-(2-Chloro-1-phenylethyl)naphthalene (62)

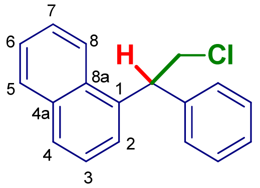

By following the **General procedure 1**, starting from naphthalen-1-yl(phenyl)methanone (200 mg, 0.86 mmol, 1 equiv) in dry THF (3 mL), chloroiodomethane (0.1 mL, 1.3 mmol, 1.5 equiv), MeLi-LiBr 2.2 M solution in Et_2O (0.6 mL, 1.2 mmol, 1.4 equiv), tris(pentafluorophenyl)borane (44 mg, 0.1 mmol, 0.1 equiv) and hexylsilane (0.13 mL, 0.77 mmol, 1 equiv), **compound 62** was obtained in 91 % yield (208 mg) as colorless oil after column chromatography on silica gel (*n*-hexane/dichloromethane 9:1 as eluent).

^{1}H NMR (500 MHz, $CDCl_3$) δ: 8.11 (d, 1H, , $^3J_{H,H}$= 8.0 Hz, Naph H-8), 7.90 (d, 1H, , $^3J_{H,H}$= 7.9 Hz, Naph H-5), 7.84 (d, 1H, $^3J_{H,H}$= 8.0 Hz, Naph H-4), 7.53 (m, 1H, Naph H-3), 7.52 (m, 1H, Naph H-7), 7.51 (m, 1H, Naph H-6), 7.47 (d, 1H, $^3J_{H,H}$= 7.2 Hz, Naph H-2), 7.35 (m, 4H, Ph H-2,3,5,6), 7.28 (m, 1H, Ph H-4), 5.21 (m, 1H, CH), 4.26 (m, 1H, CH_2Cl), 4.20 (m, 1H, CH_2Cl).

^{13}C NMR (125 MHz, $CDCl_3$) δ: 141.1 (Ph C-1), 136.4 (Naph C-1), 134.1 (Naph C-4a), 131.6 (Naph C-8a), 128.9 (Naph C-5), 128.6 (Ph C-3,5), 128.3 (Ph C-2,6), 127.8 (Naph C-4), 127.1 (Ph C-4), 126.4 (Naph C-7), 125.6 (Naph C-6), 125.2 (Naph C-3), 124.6 (Naph C-2), 123.2 (Naph C-8), 49.0 (CH), 47.0 (CH_2Cl).

EI-MS m/z (%): 266.1 (M^+, 23), 217.1 (M^+, 100).

2-[2-Chloro-1-(4-fluorophenyl)ethyl]-6-methoxynaphthalene (63)

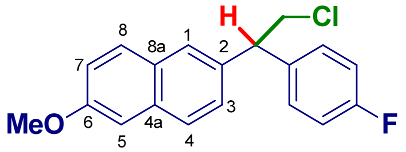

By following the **General procedure 1**, starting from (4-fluorophenyl)(6-methoxynaphthalen-2-yl)methanone (200 mg, 0.71 mmol, 1 equiv) in dry THF (3 mL), chloroiodomethane (0.1 mL, 1.1 mmol, 1.5 equiv) , MeLi-LiBr 2.2 M solution in Et_2O (0.5 mL, 1.0 mmol, 1.4 equiv), tris(pentafluorophenyl)borane (36 mg, 0.1 mmol, 0.1 equiv) and hexylsilane (0.12 mL, 0.71 mmol, 1 equiv), **compound 63** was obtained in 89 % yield (199 mg) as colorless oil after column chromatography on silica gel (*n*-hexane/diethyl ether 9:1 as eluent).

^{1}H NMR (500 MHz, $CDCl_3$) δ: 7.71 (d, 1H, $^3J_{H,H}$ = 8.9 Hz, Naph H-8), 7.70 (d, 1H, $^3J_{H,H}$ = 8.5 Hz, Naph H-4), 7.62 (d, 1H, $^3J_{H,H}$ = 1.1 Hz, Naph H-1), 7.27 (m, 1H, Naph H-3), 7.26 (m, 2H, Ph H-2,6), 7.16 (dd, 1H, $^3J_{H,H}$ = 8.9 Hz, $^4J_{H,H}$ = 2.6 Hz, Naph H-7), 7.11 (d, 1H, $^4J_{H,H}$ = 2.6 Hz, Naph H-5), 7.02 (m, 2H, Ph H-3,5), 4.46 (t, 1H, $^3J_{H,H}$ = 7.8 Hz, CH), 4.15 (dd, 1H, $^2J_{H,H}$ = 11.2 Hz, $^3J_{H,H}$ = 7.4 Hz, CH_2Cl), 4.10 (dd, 1H, $^2J_{H,H}$ = 11.2 Hz, $^3J_{H,H}$ = 8.1 Hz, CH_2Cl), 3.91 (s, 3H, OCH_3).

^{13}C NMR (125 MHz, $CDCl_3$) δ: 161.7 (d, $^1J_{C,F}$ = 245.0 Hz, Ph C-4), 157.7 (Naph C-6), 137.1 (d, $^4J_{C,F}$ = 2.8 Hz, Ph C-1), 136.2 (Naph C-2), 133.6 (Naph C-4a), 129.7 (d, $^3J_{C,F}$ = 8.1 Hz, Ph C-2,6), 129.3 (Naph C-8), 128.8 (Naph C-

8a), 127.4 (Naph C-4), 126.7 (Naph C-3), 126.2 (Naph C-1), 119.1 (Naph C-7), 115.5 (d, $^{2}J_{C,F}$ = 21.3 Hz, Ph C-3,5), 105.6 (Naph C-5), 55.3 (OCH_3), 52.7 (CH), 47.2 (CH_2Cl).

^{19}F NMR (470 MHz, $CDCl_3$) δ: -115.6 (m).

EI-MS m/z (%): 314.1 (M^+, 22), 265.1 (M^+, 100).

5-(2-Chloro-1-phenylethyl)-1,3-benzodioxole (64)

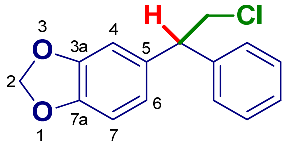

By following the **General procedure 1**, starting from benzo[d][1,3]dioxol-5-yl(phenyl)methanone (200 mg, 0.88 mmol, 1 equiv) in dry THF (3 mL), chloroiodomethane (0.1 mL, 1.3 mmol, 1.5 equiv), MeLi-LiBr 2.2 M solution in Et_2O (0.6 mL, 1.2 mmol, 1.4 equiv), tris(pentafluorophenyl)borane (45 mg, 0.1 mmol, 0.1 equiv) and hexylsilane (0.14 mL, 0.88 mmol, 1 equiv), **compound 64** was obtained in 88 % yield (201 mg) as colorless oil after column chromatography on silica gel (*n*-hexane/dichloromethane 8:2 as eluent).

^{1}H NMR (500 MHz, $CDCl_3$) δ: 7.33 (m, 2H, Ph H-3,5), 7.24 (m, 3H, Ph H-2,4,6), 6.76 (d, 1H, , $^{3}J_{H,H}$= 8.0 Hz, Benz H-7), 6.73 (dd, 1H, , $^{3}J_{H,H}$= 8.0 Hz, $^{4}J_{H,H}$= 1.8 Hz, Benz H-6), 6.70 (dd, 1H, $^{4}J_{H,H}$= 1.8 Hz, Benz H-4), 5.93 (AB system, 2H, $^{2}J_{H,H}$= 1.5 Hz, Benz H-2), 4.25 (t, 1H, $^{3}J_{H,H}$= 7.8 Hz, CH), 4.01 (m, 2H, CH_2Cl).

^{13}C NMR (125 MHz, $CDCl_3$) δ: 147.9 (Benz C-3a), 146.5 (Benz C-7a), 141.3 (Ph C-1), 135.2 (Benz C-5), 128.7 (Ph C-3,5), 127.8 (Ph C-2,6), 127.1 (Ph C-4), 121.2 (Benz C-6), 108.4 (Benz C-4), 108.3 (Benz C-7), 101.1 (Benz C-2), 53.2 (CH), 47.2 (CH_2Cl).

EI-MS m/z (%): 260.1 (M^+, 27), 211.1 (M^+, 100).

2,2'-(2-Chloro-1,1-ethanediyl)dithiophene (65)

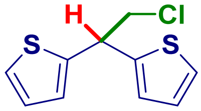

By following the **General procedure 1**, starting from di(thiophen-2-yl)methanone (200 mg, 1.03 mmol, 1 equiv) in dry THF (3 mL), chloroiodomethane (0.11 mL, 1.6 mmol, 1.5 equiv), MeLi-LiBr 2.2 M solution in Et_2O (0.66 mL, 1.4 mmol, 1.4 equiv), tris(pentafluorophenyl)borane (53 mg, 0.1 mmol, 0.1 equiv) and hexylsilane (0.17 mL, 1.03 mmol, 1 equiv), **compound 65** was obtained in 87 % yield (205 mg) as colorless oil after column chromatography on silica gel (*n*-hexane as eluent).

^{1}H NMR (500 MHz, $CDCl_3$) δ: 7.24 (dd, 1H, $^{3}J_{H,H}$ = 4.9 Hz, $^{4}J_{H,H}$ = 1.5 Hz, Th H-5), 6.99 (m, 1H, Th H-3), 6.98 (m, 1H, Th H-4), 4.83 (t, 1H, $^{3}J_{H,H}$ = 7.3 Hz, CH), 4.03 (d, 2H, $^{3}J_{H,H}$ = 7.3 Hz, CH_2Cl).

^{13}C NMR (125 MHz, $CDCl_3$) δ: 144.1 (Th C-2), 126.8 (Th C-4), 125.4 (Th C-3), 124.7 (Th C-5), 49.0 (CH_2Cl), 44.5 (CH).

EI-MS m/z (%): 228.0 (M^+, 10), 179.0 (M^+, 100).

(1-Bromo-2-propanyl)benzene (66)

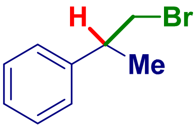

By following the **General procedure 1**, starting from acetophenone (200 mg, 1.66 mmol, 1 equiv) in dry THF (3 mL), bromoiodomethane (0.12 mL, 1.7 mmol, 1.5 equiv), MeLi-LiBr 2.2 M solution in Et_2O (1.1 mL, 2.3 mmol, 1.4 equiv), tris(pentafluorophenyl)borane (85 mg, 0.2 mmol, 0.1 equiv), and hexylsilane (0.3 mL, 1.66 mmol, 1 equiv), **compound 66** was obtained in 86% yield (280 mg) as colorless oil after column chromatography on silica gel (*n*-hexane/dichloromethane 9:1 as eluent).

Scaling-up of the reaction (20 mmol) - By following the **General procedure 1**, starting from acetophenone (2403 mg, 20.0 mmol, 1 equiv) in dry THF (30 mL), bromoiodomethane (2.26 mL, 30.0 mmol, 1.5 equiv), MeLi-LiBr 2.2 M solution in Et_2O (12.7 mL, 28.0 mmol, 1.4 equiv), tris(pentafluorophenyl)borane (1024 mg, 2.0 mmol, 0.1 equiv), and hexylsilane (3.2 mL, 20.0 mmol, 1 equiv), **compound 66** was obtained in 89% yield (3543 mg) as as colorless oil after column chromatography on silica gel (*n*-hexane/dichloromethane 9:1 as eluent). *Spectroscopic and spectrometric data match with those reported for the 1.66 mmol scale reaction.*

^{1}H NMR (500 MHz, $CDCl_3$) δ: 7.34 (m, 2H, Ph H-3,5), 7.26 (m, 1H, Ph H-4), 7.22 (m, Ph, H-2,6), 3.59 (dd, 1H, $^2J_{H,H}$ = 9.9 Hz,$^3J_{H,H}$ = 6.0 Hz, CH_2Br), 3.49 (dd, 1H, $^2J_{H,H}$ = 9.9 Hz, $^3J_{H,H}$ = 8.1 Hz, CH_2Br), 3.14 (m, 1H, CH), 1.43 (d, 3H, $^3J_{H,H}$ = 6.9 Hz, CH_3).

^{13}C NMR (125 MHz, $CDCl_3$) δ: 143.7 (Ph C-1), 128.6 (Ph C-3,5), 127.03 (Ph C-2,6), 126.98 (Ph C-4), 42.2 (CH), 40.0 (CH_2Br), 20.0 (CH_3).

1,1'-(2-Bromo-1,1-ethanediyl)dibenzene (67)

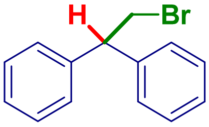

By following the **General procedure 1**, starting from benzophenone (200 mg, 1.1 mmol, 1 equiv) in dry THF (3 mL), bromoiodomethane (0.12 mL, 1.7 mmol, 1.5 equiv), MeLi-LiBr 2.2 M solution in Et_2O (0.7 mL, 1.5 mmol, 1.4 equiv), tris(pentafluorophenyl)borane (56 mg, 0.1 mmol, 0.1 equiv) and hexylsilane (0.2 mL, 1.1 mmol, 1 equiv), **compound 67** was obtained in 89% yield (256 mg) as colorless oil after column chromatography on silica gel (*n*-hexane as eluent).

^{1}H NMR (500 MHz, $CDCl_3$) δ: 7.34 (m, 4H, Ph H-3,5), 7.27 (m, 6H, Ph H-2,4,6), 4.41 (t, 1H, $^3J_{H,H}$ = 8.0 Hz, CH), 3.96 (d, 2H, $^3J_{H,H}$ = 8.0 Hz, CH_2Br).

^{13}C NMR (125 MHz, $CDCl_3$) δ: 141.7 (Ph C-1), 128.7 (Ph C-3,5), 127.9 (Ph C-2,6), 127.0 (Ph C-4), 53.6 (CH), 35.5 (CH_2Br).

EI-MS m/z (%): 260.0 (M^+, 9), 167.1 (M^+, 100).

1,1'-(2-Fluoro-1,1-ethanediyl)dibenzene (68)

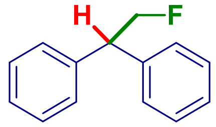

By following the **General procedure 2**, starting from benzophenone (273 mg, 1.5 mmol, 1.5 equiv) in dry mixture of THF (3 mL), fluoroiodomethane (0.1 mL, 1.0 mmol, 1 equiv), MeLi-LiBr 2.2 M solution in Et_2O (1.0 mL, 2.0 mmol, 2 equiv), tris(pentafluorophenyl)borane (56 mg, 0.1 mmol, 0.1 equiv) and hexylsilane (0.12 mL, 0.73 mmol, 1 equiv), **compound 68** in 91% yield (200 mg) as colorless oil after column chromatography on silica gel (*n*-hexane/dichloromethane 9:1 as eluent).

^{1}H NMR (200 MHz, $CDCl_3$) δ: 7.34-7.21 (m, 10H, Ph H-2,3,4,5,6), 4.92 (dd, 2H, $^2J_{H,F}$ = 47.1 Hz, $^3J_{H,H}$ = 7.0 Hz, CH_2F), 4.40 (m, 1H, CH).

^{13}C NMR (125 MHz, $CDCl_3$) δ: 140.3 (d, $^3J_{C,F}$ = 5.0 Hz, Ph C-1), 128.6 (Ph C-3,5), 128.3 (Ph C-2,6), 126.9 (Ph C-4), 85.3 (d, $^1J_{C,F}$ = 175.0 Hz, CH_2F), 51.3 (d, $^2J_{C,F}$ = 19.6 Hz, CH).

^{19}F NMR (376 MHz, $CDCl_3$) δ: -214.7 (dt, $^2J_{H,F}$ = 47.1 Hz, $^3J_{H,F}$ = 15.0 Hz).

EI-MS m/z (%): 200.1(M^+, 23), 167.1 (M^+, 100).

9-(Chloromethyl)-9*H*-xanthene (69)

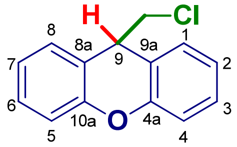

By following the **General procedure 1**, starting from 9H-xanthen-9-one (200 mg, 1.02 mmol, 1 equiv) in dry THF (3 mL), chloroiodomethane (0.11 mL, 1.53 mmol, 1.5 equiv), MeLi-LiBr 2.2 M solution in Et_2O (0.65 mL, 1.42 mmol, 1.4 equiv), tris(pentafluorophenyl)borane (52 mg, 0.10 mmol, 0.1 equiv) and hexylsilane (0.16 mL, 1.02 mmol, 1 equiv), **compound 69** was obtained in 89 % yield (209 mg) as colorless oil after column chromatography on silica gel (*n*-hexane as eluent).

^{1}H NMR (500 MHz, $CDCl_3$) δ: 7.35 (m, 2H, Ar H-1,8), 7.32 (m, 2H, Ar H-3,6), 7.16 (m, 2H, Ar H-4,5), 7.14 (m, 2H, Ar H-2,7), 4.29 (t, 1H, $^3J_{H,H}$ = 6.1 Hz, CH), 3.69 (d, 2H, $^3J_{H,H}$ = 6.1 Hz, CH_2Cl).

^{13}C NMR (125 MHz, $CDCl_3$) δ: 152.2 (Ar C-4a,10a), 129.2 (Ar C-1,8), 128.6 (Ar C-3,6), 123.2 (Ar C-2,7), 121.7 (Ar C-8a,9a), 116.6 (Ar C-4,5), 51.1 (CH_2Cl), 41.5 (CH).

EI-MS m/z (%): 230.0 (M^+, 5), 181.1 (M^+, 100).

9-(Chloromethyl)-9*H*-thioxanthene (70)

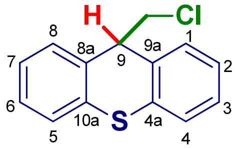

By following the **General procedure 1**, starting from 9H-thioxanthen-9-one (200 mg, 0.94 mmol, 1 equiv) in dry THF (3 mL), chloroiodomethane (0.10 mL, 1.41 mmol, 1.5 equiv), MeLi-LiBr 2.2 M solution in Et_2O (0.6

mL, 1.3 mmol, 1.4 equiv), tris(pentafluorophenyl)borane (46 mg, 0.09 mmol, 0.1 equiv) and hexylsilane (0.15 mL, 0.94 mmol, 1 equiv), **compound 70** was obtained in 93 % yield (215 mg) as colorless oil after column chromatography on silica gel (*n*-hexane as eluent).

^{1}H NMR (500 MHz, $CDCl_3$) δ: 7.43- 7.23 (m, 8H, Ar H-1,2,3,4,5,6,7,8), 4.31 (t, 1H, $^3J_{H,H}$ = 7.9 Hz, CH), 3.72 (d, 2H, $^3J_{H,H}$ = 7.9 Hz, CH_2Cl).

^{13}C NMR (125 MHz, $CDCl_3$) δ: 134.4 (C_q), 132.2 (C_q), 130.2 (Ar CH), 127.5 (Ar CH), 126.9 (Ar CH), 126.6 (Ar CH), 51.6 (CH), 44.1 (CH_2Cl).

EI-MS m/z (%): 246.0 (M^+, 11), 197.0 (M^+, 100).

2-Chloro-9-(chloromethyl)-9*H*-thioxanthene (71)

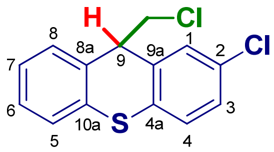

By following the **General procedure 1**, starting from 2-chloro-9H-thioxanthen-9-one (200 mg, 0.81 mmol, 1 equiv) in dry THF (3 mL), chloroiodomethane (0.08 mL, 1.21 mmol, 1.5 equiv), MeLi-LiBr 2.2 M solution in Et_2O (0.51 mL, 1.13 mmol, 1.4 equiv), tris(pentafluorophenyl)borane (40 mg, 0.08 mmol, 0.1 equiv) and hexylsilane (0.13 mL, 0.81 mmol, 1 equiv), **compound 71** was obtained in 87 % yield (198 mg) as colorless oil after column chromatography on silica gel (*n*-hexane as eluent).

^{1}H NMR (400 MHz, $CDCl_3$) δ: 7.42 (m, 1H, Ar H-5), 7.37 (m, 2H, Ar H-1,8), 7.34 (m, 1H, Ar H-4), 7.28 (2H, Ar H-6,7), 7.24 (dd, $^3J_{H,H}$ = 8.4 Hz, $^4J_{H,H}$ = 2.2 Hz, 1H, Ar H-3), 4.26 (t, $^3J_{H,H}$ = 7.9 Hz, 1H, Ar H-9), 3.72 (dd, $^2J_{H,H}$ = 10.8 Hz, $^3J_{H,H}$ = 8.0 Hz, 1H, CH_2Cl), 3.68 (dd, $^2J_{H,H}$ = 10.8 Hz, $^3J_{H,H}$ = 7.7 Hz, 1H, CH_2Cl).

^{13}C NMR (100 MHz, $CDCl_3$) δ: 136.1 (Ar C-9a),133.8 (Ar C-8a), 132.2 (Ar C-2), 131.8 (Ar C-10a), 130.8 (Ar C-4a), 130.1 (Ar C-1), 130.0 (Ar C-8), 128.0 (Ar C-4), 127.7 (Ar C-6), 127.6 (Ar C-3), 127.0 (Ar C-5), 126.9 (Ar C-7), 51.4 (Ar C-9), 43.7 (CH_2Cl).

EI-MS m/z (%): 280.0 (M^+, 11), 231.0 (M^+, 100).

9-(Bromomethyl)-9*H*-thioxanthene (72)

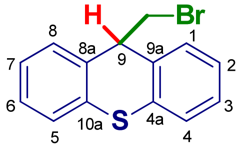

By following the **General procedure 1**, starting from 9H-thioxanthen-9-one (200 mg, 0.94 mmol, 1 equiv) in dry THF (3 mL), bromoiodomethane (0.11 mL, 1.41 mmol, 1.5 equiv), MeLi-LiBr 2.2 M solution in Et_2O (0.6 mL, 1.3 mmol, 1.4 equiv), tris(pentafluorophenyl)borane (48 mg, 0.1 mmol, 01 equiv) and hexylsilane (0.2 mL, 0.94 mmol, 1 equiv), **compound 72** was obtained in 82% yield (225 mg) as colorless oil after column chromatography on silica gel (*n*-hexane as eluent).

^{1}H NMR (400 MHz, C_6D_6) δ: 7.19 (m, 2H, Ar H-4,5), 6.98 (m, 2H, Ar H-1,8), 6.94 (m, 2H, Ar H-2,7), 6.87 (m, 2H, Ar H-3,6), 4.05 (t, $^3J_{H,H}$ = 7,9 Hz, 1H, CH) , 3.38 (d, $^3J_{H,H}$ = 7.9 Hz, 2H, CH_2Br).

^{13}C NMR (100 MHz, C_6D_6) δ: 135.3 (Ar C-8a,9a), 132.6 (Ar C-4a,10a),130.4 (Ar C-1,8), 127.6 (Ar C-3,6), 127.2 (Ar C-4,5), 126.6 (Ar C-2,7), 51.7 (Ar C-9), 33.2 (CH_2Br).

EI-MS m/z (%): 289.9 (M^+, 5), 197.0 (M^+, 100).

1,1'-(2,2-Dibromo-1,1-ethanediyl)dibenzene (73)

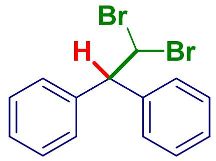

By following the **General procedure 2**, starting from benzophenone (200 mg, 1.09 mmol, 1 equiv) in dry THF (3 mL), dibromomethane (0.11 mL, 1.63 mmol, 1.5 equiv), TMP (0.26 mL, 1.53 mmol, 1.4 equiv), MeLi-LiBr 2.2 M solution in Et_2O (0.69 mL, 1.53 mmol, 1.4 equiv), tris(pentafluorophenyl)borane (56 mg, 0.1 mmol, 0.1 equiv) and hexylsilane (0.17 mL, 1.09 mmol, 1 equiv), **compound 73** was obtained in 87% yield (322 mg) as colorless oil after column chromatography on silica gel (*n*-hexane/diethyl ether 95:5 as eluent).

Scaling-up of the reaction (15 mmol) - By following the **General procedure 2**, starting from benzophenone (2733 mg, 15.0 mmol, 1 equiv) in dry THF (30 mL), dibromomethane (1.6 mL, 22.5 mmol, 1.5 equiv), TMP (3.6 mL, 21.0 mmol, 1.4 equiv), MeLi-LiBr 2.2 M solution in Et_2O (9.5 mL, 21.0 mmol, 1.4 equiv), tris(pentafluorophenyl)borane (768 mg, 1.5 mmol, 0.1 equiv) and hexylsilane (2.4 mL, 15.0 mmol, 1 equiv), **compound 74** was obtained in 83% yield (4207 mg) as as colorless oil after column chromatography on silica gel (*n*-hexane/diethyl ether 95:5 as eluent). *Spectroscopic and spectrometric data match with those reported for the 1.09 mmol scale reaction.*

^{1}H NMR (500 MHz, $CDCl_3$) δ: 7.35 (m, 4H, Ph H-3,5), 7.33 (m, 4H, Ph H-2,6), 7.27 (m, 2H, Ph H-4), 6.31 (d, 1H, $^3J_{H,H}$ = 9.7 Hz, $CHBr_2$), 4.69 (d, 1H, $^3J_{H,H}$ = 9.7 Hz, Ph-C$\underline{H}$).

^{13}C NMR (125 MHz, $CDCl_3$) δ: 140.8 (Ph C-1), 128.7 (Ph C-3,5) 127.9 (Ph C-2,6), 127.5 (Ph C-4), 63.7 (CH), 47.5 ($CHBr_2$).

EI-MS m/z (%): 337.9 (M^+, 5), 167.1 (M^+, 100).

(2,2-Dibromoethyl)benzene (74)

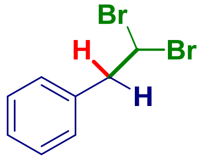

By following the **General procedure 2**, starting from benzaldehyde (200 mg, 1.88 mmol, 1 equiv) in dry THF (3 mL), dibromomethane (0.2 mL, 2.82 mmol, 1.5 equiv), TMP (0.44 mL, 2.63 mmol, 1.4 equiv), MeLi-LiBr 2.2 M solution in Et_2O (1.19 mL, 2.63 mmol, 1.4 equiv), tris(pentafluorophenyl)borane (96 mg, 0.19 mmol, 0.1 equiv) and hexylsilane (0.3 mL, 1.88 mmol, 1 equiv), **compound 74** was obtained in 85% yield (422 mg) as colorless oil after column chromatography on silica gel (*n*-hexane as eluent).

^{1}H NMR (500 MHz, $CDCl_3$) δ: 7.36 (m, 2H, Ph H-3,5), 7.35 (m, 1H, Ph H-4), 7.27 (m, 2H, Ph H-2,6), 5.77 (t, 1H, $^3J_{H,H}$ = 6.8 Hz, $CHBr_2$), 3.72 (d, $^3J_{H,H}$ = 6.8 Hz, CH_2).

^{13}**C NMR** (125 MHz, $CDCl_3$) δ: 136.7 (Ph C-1), 129.5 (Ph C-2,6), 128.6 (Ph C-3,5), 127.7 (Ph C-4), 51.4 (CH_2), 45.1 ($CHBr_2$).

1,1'-(2,2-Dichloro-1,1-ethanediyl)dibenzene (75)

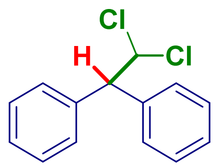

By following the **General procedure 2**, starting from benzophenone (200 mg, 1.09 mmol, 1 equiv) in dry THF (3 mL), dichloromethane (0.1 mL, 1.63 mmol, 1.5 equiv), TMP (0.26 mL, 1.53 mmol, 1.4 equiv), MeLi-LiBr 2.2 M solution in Et_2O (0.69 mL, 1.53 mmol, 1.4 equiv), tris(pentafluorophenyl)borane (56 mg, 0.1 mmol, 0.1 equiv) and hexylsilane (0.17 mL, 1.09 mmol, 1 equiv), **compound 75** was obtained in 93% yield (256 mg) as colorless oil after column chromatography on silica gel (*n*-hexane/diethyl ether 9:1 as eluent).

^{1}**H NMR** (200 MHz, $CDCl_3$) δ: 7.34 (m, 4H, Ph H-2,6), 7.33 (m, 4H, Ph H-3,5), 7.26 (m, 2H, Ph H-4), 6.38 (d, 1H, $^{3}J_{H,H}$ = 8.7 Hz, $CHCl_2$), 4.56 (d, 1H, $^{3}J_{H,H}$ = 8.7 Hz, Ph-C$\underline{H}$).

^{13}**C NMR** (125 MHz, $CDCl_3$) δ: 139.8 (Ph C-1), 128.7 (Ph C-3,5) 128.3 (Ph C-2,6), 127.5 (Ph C-4), 74.6 ($CHCl_2$), 62.7 (Ph-$\underline{C}$H).

EI-MS m/z (%): 250.0 (M^+, 5), 167.1 (M^+, 100).

1-Bromo-4-(1,1-dichloro-2-propanyl)benzene (76)

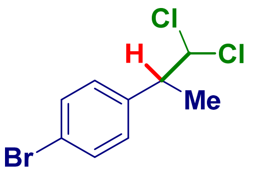

By following the **General procedure 2**, starting from 4'-bromoacetophenone (200 mg, 1.01 mmol, 1 equiv) in dry THF (3 mL), dichloromethane (0.1 mL, 1.51 mmol, 1.5 equiv), TMP (0.24 mL, 1.41 mmol, 1.4 equiv), MeLi-LiBr 2.2 M solution in Et_2O (0.64 mL, 1.41 mmol, 1.4 equiv), tris(pentafluorophenyl)borane (52 mg, 0.1 mmol, 0.1 equiv) and hexylsilane (0.16 mL, 1.01 mmol, 1 equiv), **compound 76** was obtained in 91% yield (245 mg) as colorless oil after column chromatography on silica gel (*n*-hexane 100% as eluent).

^{1}**H NMR** (500 MHz, $CDCl_3$) δ: 7.47 (m, 2H, Ph H-2,6), 7.17 (m, 2H, Ph H-3,5), 5.84 (d, 1H, $^{3}J_{H,H}$ = 4.9 Hz, $CHCl_2$), 3.41 (dq, 1H, q: $^{3}J_{H,H}$ = 7.0 Hz, d: $^{3}J_{H,H}$ = 4.9 Hz, CH), 1.52 (d, $^{3}J_{H,H}$ = 7.0 Hz, CH_3).

^{13}**C NMR** (125 MHz, $CDCl_3$) δ: 139.2 (Ph C-1), 131.6 (Ph C-2,6), 130.0 (Ph C-3,5), 121.7 (Ph C-1), 77.5 ($CHCl_2$), 49.6 (CH), 15.9 (CH_3).

1-(2-Bromo-2-chloroethyl)-2,4-dichlorobenzene (77)

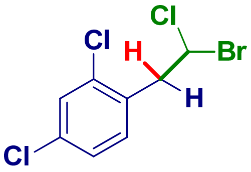

By following the **General procedure 2**, starting from 2,4-dichlorobenzaldehyde (200 mg, 1.14 mmol, 1 equiv) in dry THF (3 mL), bromochloromethane (0.14 mL, 1.71 mmol, 1.5 equiv), TMP (0.27 mL, 1.6 mmol, 1.4 equiv), MeLi-LiBr 2.2 M solution in Et_2O (0.72 mL, 1.6 mmol, 1.4 equiv), tris(pentafluorophenyl)borane (58 mg, 0.11 mmol, 0.1 equiv) and hexylsilane (0.2 mL, 1.14 mmol, 1 equiv), **compound 77** was obtained in 94% yield (303 mg) as colorless oil after column chromatography on silica gel (*n*-hexane as eluent).

^{1}H NMR (500 MHz, $CDCl_3$) δ: 7.41 (d, 1H, , $^4J_{H,H}$= 1.9 Hz, Ph H-3), 7.27 (d, 1H, $^3J_{H,H}$= 8.2 Hz, Ph H-6), 7.24 (dd, 1H, $^3J_{H,H}$= 8.2 Hz, $^4J_{H,H}$= 1.9 Hz, Ph H-5), 5.95 (dd, 1H, $^3J_{H,H}$= 7.1 Hz, $^3J_{H,H}$= 6.6 Hz, CHClBr), 3.72 (dd, 1H, $^2J_{H,H}$= 14.4 Hz, $^3J_{H,H}$= 6.6 Hz, CH_2), 3.67 (dd, 1H, $^2J_{H,H}$= 14.4 Hz, $^3J_{H,H}$= 7.1 Hz, CH_2).

^{13}C NMR (125 MHz, $CDCl_3$) δ: 134.9 (Ph C-2), 134.6 (Ph C-4), 133.2 (Ph C-6), 132.4 (Ph C-1), 129.5 (Ph C-3), 127.3 (Ph C-5), 57.3 (CHClBr), 47.9 (CH_2).

2-(2-Bromo-2-chloroethyl)-1,3-dichlorobenzene (78)

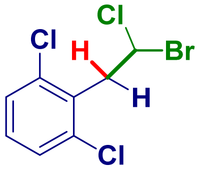

By following the **General procedure 2**, starting from 2,6-dichlorobenzaldehyde (200 mg, 1.14 mmol, 1 equiv) in dry THF (3 mL), bromochloromethane (0.14 mL, 1.71 mmol, 1.5 equiv), TMP (0.27 mL, 1.6 mmol, 1.4 equiv), MeLi-LiBr 2.2 M solution in Et_2O (0.72 mL, 1.6 mmol, 1.4 equiv), tris(pentafluorophenyl)borane (58 mg, 0.11 mmol, 0.1 equiv) and hexylsilane (0.2 mL, 1.14 mmol, 1 equiv), **compound 78** was obtained in 94% yield (303 mg) as colorless oil after column chromatography on silica gel (*n*-hexane as eluent).

^{1}H NMR (500 MHz, $CDCl_3$) δ: 7.33 (d, 2H, Ph H-3,5), 7.20 (t, 1H, Ph H-4), 6.17 (t, 1H, CHClBr), 3.99 (dd, 2H, CH_2).

^{13}C NMR (125 MHz, $CDCl_3$) δ: 136.2 (Ph C-1), 132.5 (2C, Ph C-2,6), 129.5 (2C, Ph C-4), 128.6 (Ph C-3), 56.6 (CH_2), 45.0 (CHClBr).

(2-Chloro-2-iodoethyl)benzene (79)

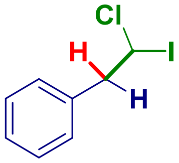

By following the **General procedure 2**, starting from benzaldehyde (200 mg, 1.88 mmol, 1 equiv) in dry THF (3 mL), chloroiodomethane (0.2 mL, 2.83 mmol, 1.5 equiv), TMP (0.44 mL, 2.63 mmol, 1.4 equiv), MeLi-LiBr 2.2 M solution in Et_2O (1.19 mL, 2.63 mmol, 1.4 equiv), tris(pentafluorophenyl)borane (96 mg, 0.19 mmol,

0.1 equiv) and hexylsilane (0.3 mL, 1.88 mmol, 1 equiv), **compound 79** was obtained in 90% yield (453 mg) as colorless oil after column chromatography on silica gel (*n*-hexane as eluent).

^{1}H NMR (400 MHz, $CDCl_3$) δ: 7.34 (m, 3H, Ph H-3,4,5), 7.25 (m, 2H, Ph H-2,6), 5.81 (m, 1H, CHICl), 3.68 (dd, $^2J_{H,H}$ = 14.4 Hz, $^3J_{H,H}$ = 6.5 Hz, $^1J_{H,H}$ = 1H, CH_2) 3.58 (dd, $^2J_{H,H}$ = 14.4 Hz, $^3J_{H,H}$ = 7.2 Hz, 1H, CH_2).

^{13}C NMR (100 MHz, $CDCl_3$) δ: 137.4 (1C, Ph C-1), 129.4 (Ph C-2,6), 128.6 (Ph C-3,5), 127.7 (Ph C-4), 52.7 (CH_2), 29.5 (CHClI).

EI-MS m/z (%): 265.9 (M^+, 6), 139.1 (M^+, 100).

2-(2-Bromo-2-iodoethyl)-1,3-dimethoxybenzene (80)

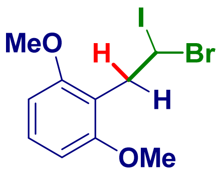

By following the **General procedure 2**, starting from 2,6-dimethoxybenzaldehyde (200 mg, 1.2 mmol, 1 equiv) in dry THF (3 mL), bromoiodomethane (0.13 mL, 1.8 mmol, 1.5 equiv), TMP (0.28 mL, 1.68 mmol, 1.4 equiv), MeLi-LiBr 2.2 M solution in Et_2O (0.76 mL, 1.68 mmol, 1.4 equiv), tris(pentafluorophenyl)borane (61 mg, 0.12 mmol, 0.1 equiv) and hexylsilane (0.2 mL, 1.2 mmol, 1 equiv), **compound 80** was obtained in 92% yield (409 mg) as colorless oil after column chromatography on silica gel (*n*-hexane/diethyl ether 9:1 as eluent).

^{1}H NMR (400 MHz, $CDCl_3$) δ: 7.18 (t, $^2J_{H,H}$ = 8.3 Hz, 1 H, Ph H-5), 6.54 (d, 2H, $^2J_{H,H}$ = 8.3 Hz, Ph H-4,6), 3.82 (s, 6H, OCH_3), 3.46 (m, 1H, CHBrI), 3.23 (m, 2H, CH_2).

^{13}C NMR (100 MHz, $CDCl_3$) δ: 158.4 (Ph C-1,3), 128.0 (Ph C-5), 115.3 (Ph C-2), 103.6 (Ph C-4,6), 55.6 (OCH_3), 31.3 (CHBrI), 27.1 (CH_2).

1,1'-(2,2-Difluoro-1,1-ethanediyl)dibenzene (81)

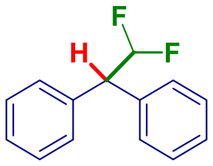

By following the **General procedure 3**, starting from benzophenone (200 mg, 1.09 mmol, 1 equiv) in dry THF (3 mL), difluoromethyltrimethylsilane (0.23 mL, 1.64 mmol, 1.5 equiv), potassium *tert*-pentoxide 0.9 M in toluene (1.7 mL, 1.53 mmol, 1.4 equiv), tris(pentafluorophenyl)borane (56 mg, 0.11 mmol, 0.1 equiv) and hexylsilane (0.17 mL, 1.09 mmol, 1 equiv), **compound 81** was obtained in 87% yield (209 mg) as colorless oil after column chromatography on silica gel (*n*-hexane as eluent).

^{1}H NMR (400 MHz, $CDCl_3$) δ: 7.35 (m, 4H, Ph H-3,5), 7.32 (m, 4H, Ph H-2,6), 7.29 (m, 2H, Ph H-4), 6.33 (dt, 1H, $^2J_{H,F}$ = 55.8 Hz, $^3J_{H,H}$ = 4.5 Hz, CH, CHF_2), 4.42 (dt, 1H, $^3J_{H,F}$ = 15.8 Hz, $^3J_{H,H}$ = 4.5 Hz, CH).

^{13}C NMR (100 MHz, $CDCl_3$) δ: 137.1 (t, $^3J_{H,F}$ = 3.7 Hz, Ph C-1), 129.0 (Ph C-2,6), 128.7 (Ph C-3,5), 127.5 (Ph C-4), 116.9 (t, $^1J_{C,F}$ = 244.4 Hz, CHF_2), 55.0 (t, $^2J_{C,F}$ = 20.8 Hz, CH).

^{19}**F NMR** (376 MHz, $CDCl_3$) δ: -118.1 (dd, $^2J_{H,F}$ = 55.8 Hz, $^3J_{H,F}$ = 15.8 Hz, CHF_2).

1,1'-(2,2,2-Trifluoro-1,1-ethanediyl)dibenzene (82)

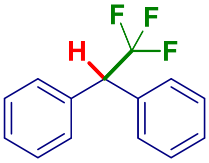

By following the **General procedure 4**, starting from Benzophenone (140 mg, 0.77 mmol, 1 equiv) in dry THF (3 mL), trifluoromethyltrimethylsilane (0.17 mL, 1.16 mmol, 1.5 equiv), tetrabutylammonium fluoride (TBAF) solution 1.0 M in THF (1.1 mL, 1.09 mmol, 1.4 equiv), tris(pentafluorophenyl)borane (39 mg, 0.08 mmol, 0.1 equiv), and hexylsilane (0.12 mL, 0.77 mmol, 1 equiv), **compound 82** was obtained in 79% yield (144 mg) as colorless oil after column chromatography on silica gel (*n*-hexane/dichloromethane 1:1 as eluent).

^{1}**H NMR** (400 MHz, $CDCl_3$) δ: 7.41 (m, 4H, Ph H-2,6), 7.38 (m, 4H, Ph H-3,5), 7.36 (m, 2H, Ph H-4), 4.73 (q, $^3J_{H,F}$ = 10.0 Hz, 1H, CH).

^{13}**C NMR** (100 MHz, $CDCl_3$) δ: 135,4 (q, $^3J_{C,F}$ = 1.6 Hz, Ph C-1), 129.1 (q, $^4J_{C,F}$ = 1.2 Hz, Ph C-2,6), 128.7 (Ph C-3,5), 127,9 (Ph C-4), 126.2 (q, $^1J_{C,F}$ = 280.5 Hz, CF_3), 55.5 (q, $^2J_{C,F}$ = 27.5 Hz, CH).

^{19}**F NMR** (376 MHz, $CDCl_3$) δ: -65.8 (d, $^3J_{F,H}$ = 10.0 Hz, CF_3).

[2-(4-Chlorophenyl)ethyl](trimethyl)silane (83)

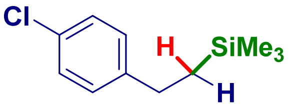

By following the **General procedure 5**, starting from 4-chlorobenzaldehyde (200 mg, 1.42 mmol, 1 equiv) in dry THF (3 mL), trimethylsilyl-methyllithium solution 0.7 M in pentane (3.0 mL, 2.13 mmol, 1.5 equiv), tris(pentafluorophenyl)borane (73 mg, 0.14 mmol, 0.1 equiv) and hexylsilane (0.23 mL, 1.4 mmol, 1 equiv), **compound 83** was obtained in 84% yield (250 mg) as colorless oil after column chromatography on silica gel (*n*-hexane as eluent).

^{1}**H NMR** (500 MHz, $CDCl_3$) δ: 7.23 (m, 2H, Ph H-3,5), 7.12 (m, 2H, Ph H-2,6), 2.59 (m, 2H, Ph-C$\underline{H_2}$), 0.84 (m, 2H, C$\underline{H_2}$Si), 0.01 (s, 9H, Me_3Si).

^{13}**C NMR** (125 MHz, $CDCl_3$) δ: 143.7 (Ph C-1), 131.0 (Ph C-4), 129.1 (Ph C-2,6), 128.3 (Ph C-3,5), 29.5 (Ph-$\underline{C}H_2$), 18.6 ($\underline{C}H_2Si$), -1.8 (Me_3Si).

(2,2-Diphenylethyl)trimethylsilane (84)

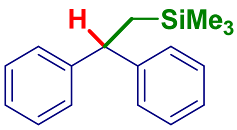

By following the **General procedure 5**, starting from benzophenone (200 mg, 1.09 mmol, 1 equiv) in dry THF (3 mL), trimethylsilyl-methyllithium solution 0.7 M in pentane (2.3 mL, 1.64 mmol, 1.5 equiv),

tris(pentafluorophenyl)borane (56 mg, 0.11 mmol, 0.1 equiv) and hexylsilane (0.17 mL, 1.09 mmol, 1 equiv), **compound 84** was obtained in 87% yield (244 mg) as colorless oil after column chromatography on silica gel (*n*-hexane as eluent).

^{1}H NMR (500 MHz, C_6D_6) δ: 7.19 (m, 4H, Ph H-2,6), 7.12 (m, 4H, Ph H-3,5), 7.01 (m, 2H, Ph H-4), 4.01 (t, 1H, $^3J_{H,H}$ = 8.1 Hz, CH), 1.29 (d, 2H, $^3J_{H,H}$ = 8.1 Hz, CH_2), -0.16 (s, 3H, CH_3).

^{13}C NMR (125 MHz, C_6D_6) δ: 147.5 (Ph C-1), 128.6 (Ph C-3,5), 128.0 (Ph C-2,6), 126.3 (Ph C-4), 47.7 (CH), 24.3 (CH_2), -1.1 ($SiCH_3$).

[(2-Phenylethyl)sulfanyl]benzene (85)

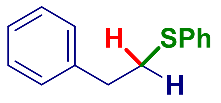

By following the **General procedure 6**, starting from benzaldehyde (150 mg, 1.41 mmol, 1 equiv) in dry THF (3 mL), thioanisole (0.25 mL, 2.16 mmol, 1.5 equiv), DABCO (242 mg, 2.16 mmol, 1.5 equiv), *n*-butyllithium 2.5 M in *n*-hexane (0.78 mL, 1.96 mmol, 1.4 equiv), tris(pentafluorophenyl)borane (72 mg, 0.14 mmol, 0.1 equiv) and hexylsilane (0.23 mL, 1.41 mmol, 1 equiv), **compound 85** was obtained in 83% yield (250 mg) as colorless oil after column chromatography on silica gel (*n*-hexane as eluent).

^{1}H NMR (500 MHz, $CDCl_3$) δ: 7.36 (m, 2H, SPh H-2,6), 7.31 (m, 2H, Ph H-3,5), 7.30 (m, 2H, SPh H-3,5), 7.23 (m, Ph H-4), 7.20 (m, 3H, SPh H-4, Ph H-2,6), 3.18 (m, 2H, $C\underline{H_2}S$), 2.93 (m, 2H, Ph-$C\underline{H_2}$).

^{13}C NMR (125 MHz, $CDCl_3$) δ: 140.2 (Ph C-1), 136.3 (SPh C-1), 129.2 (2C, SPh C-2,6), 128.9 (2C, Ph C-3,5), 128.5 (4C, SPh C-3,5, Ph C-2,6), 126.4 (Ph C-4), 126.0 (SPh C-4), 35.6 (Ph-$\underline{C}H_2$), 35.1 (SCH_2).

[(2-Phenylpropyl)sulfanyl]benzene (86)

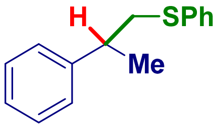

By following the **General procedure 6**, starting from acetophenone (150 mg, 1.25 mmol, 1 equiv) in dry THF (3 mL), thioanisole (0.22 mL, 1.88 mmol, 1.5 equiv), DABCO (210 mg, 1.88 mmol, 1.5 equiv), *n*-butyllithium 2.5 M in *n*-hexane (0.7 mL, 1.75 mmol, 1.4 equiv), tris(pentafluorophenyl)borane (66 mg, 0.13 mmol, 0.1 equiv) and hexylsilane (0.2 mL, 1.25 mmol, 1 equiv), **compound 86** was obtained in 81% yield (231 mg) as colorless oil after column chromatography on silica gel (*n*-hexane/diethyl ether 95:5 as eluent).

^{1}H NMR (500 MHz, C_6D_6) δ: 7.22 (m, 2H, SPh H-2,6), 7.13 (m, 2H, Ph H-3,5), 7.06 (m, 1H, Ph H-4), 7.01 (m, 2H, SPh H-3,5), 6.97 (m, 2H, Ph H-2,6), 6.92 (m, 1H, SPh H-4), 3.03 (dd, 1H, $^2J_{H,H}$= 12.0 Hz, $^3J_{H,H}$= 5.3 Hz, CH_2S), 2.83 (m, 1H, CH), 2.79 (dd, 1H, $^2J_{H,H}$= 12.0 Hz, , $^3J_{H,H}$= 8.4 Hz, CH_2S), 1.22 (d, 3H, $^3J_{H,H}$= 6.7 Hz, CH_3).

^{13}C NMR (125 MHz, C_6D_6) δ: 145.9 (Ph C-1), 137.7 (SPh C-1), 129.3 (SPh C-2,6), 129.2 (SPh C-3,5), 128.8 (Ph C-3,5), 127.3 (Ph C-2,6), 126.8 (Ph C-4), 125.9 (SPh C-4), 42.0 (CH_2S), 39.7 (CH), 21.1 (CH_3).

1,1'-[2-(Phenylsulfanyl)-1,1- ethanediyl]dibenzene (87)

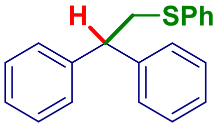

By following the **General procedure 6**, starting from benzophenone (150 mg, 0.8 mmol, 1 equiv) in dry THF (3 mL), thioanisole (0.14 mL, 1.2 mmol, 1.5 equiv), DABCO (134 mg, 1.2 mmol, 1.5 equiv), *n*-butyllitium 2.5 M in *n*-hexane (0.45 mL, 1.22 mmol, 1.4 equiv), tris(pentafluorophenyl)borane (41 mg, 0.08 mmol, 0.1 equiv) and hexylsilane (0.13 mL, 0.8 mmol, 1 equiv), **compound 87** was obtained in 85% yield (197 mg) as colorless oil after column chromatography on silica gel (*n*-hexane as eluent *n*-hexane/diethyl ether 95:5 as eluent).

^{1}H NMR (400 MHz, $CDCl_3$) δ: 7.33(m, 6H, Ph H-3,5, SPh H-2,6), 7.30 (m, 2H, SPh H-3,5), 7.27 (m, 4H, Ph H-2,6), 7.24 (m, 2H, Ph H-4), 7.21 (m, 1H, SPh H-4), 4.24 (t, $^3J_{H,H}$ = 7.9 Hz, 1H, CH), 3.63 (d, $^3J_{H,H}$ = 7.9 Hz, 2H, CH_2Cl).

^{13}C NMR (100 MHz, $CDCl_3$) δ: 143.0 (Ph C-1),136.5 (SPh C-1), 129.4 (SPh C-2,6), 128.9 (SPh C-3,5), 128.5 (Ph C-3,5), 127.9 (Ph C-2,6), 126.7 (Ph C-4), 126.0 (SPh C-4), 50.5 (CH), 39.6 (CH_2).

1,1'-(1,1-ethanediyl)dibenzene (88)

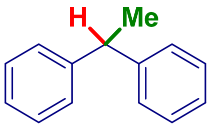

By following the **General procedure 5**, starting from benzophenone (200 mg, 1.09 mmol, 1 equiv) in dry THF (3 mL), MeLi solution 1.6 M in THF (1.02 mL, 1.64 mmol, 1.5 equiv) , tris(pentafluorophenyl)borane (55 mg, 0.11 mmol, 0.1 equiv) and hexylsilane (0.17 mL, 1.09 mmol, 1 equiv), **compound 88** was obtained in 91% yield (183 mg) as colorless oil after column chromatography on silica gel (*n*-hexane as eluent).

^{1}H NMR (500 MHz, $CDCl_3$) δ: 7.32 (m, 4H, Ph H-3,5), 7.26 (m, 4H, Ph H-2,6), 7.22 (m, 2H, Ph H-4), 4.19 (q, 1H, $^3J_{H,H}$ = 7.3 Hz, CH), 1.68 (d, 3H, $^3J_{H,H}$ = 7.3 Hz, CH_3).

^{13}C NMR (125 MHz, $CDCl_3$) δ: 146.3 (Ph C-1), 128.3 (Ph C-3,5) 127.6 (Ph C-2,6), 126.0 (Ph C-4), 44.7 (CH), 21.8 (CH_3).

Triphenylmethane (89)

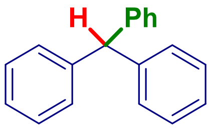

By following the **General procedure 5**, starting from benzophenone (200 mg, 1.09 mmol, 1 equiv) in dry THF (3 mL), phenyllithium solution 1.9 M in *n*-Bu_2O (0.79 mL, 1.64 mmol, 1.5 equiv), tris(pentafluorophenyl)borane (55 mg, 0.11 mmol, 0.1 equiv) and hexylsilane (0.17 mL, 1.09 mmol, 1 equiv), **compound 89** was obtained in 83% yield (222 mg) as colorless oil after column chromatography on silica gel (*n*-hexane as eluent).

^{1}H-NMR (400 MHz, CDCl3) δ: 7.31 (m, 6H, Ph H-3,5), 7.23 (m, 3H, Ph H-4), 7.15 (m, 6H, Ph H-2,6), 5.58 (s, 1H, CH).

^{13}C NMR (100 MHz, CDCl3) δ: 143.9 (Ph C-1), 129.4 (Ph C-2,6), 128.3 (Ph C-3,5), 126.3 (Ph C-4), 56.8 (CH).

5. ^{1}H-, ^{13}C- and ^{19}F-NMR Spectra.

(2-Iodoethyl)benzene (2)

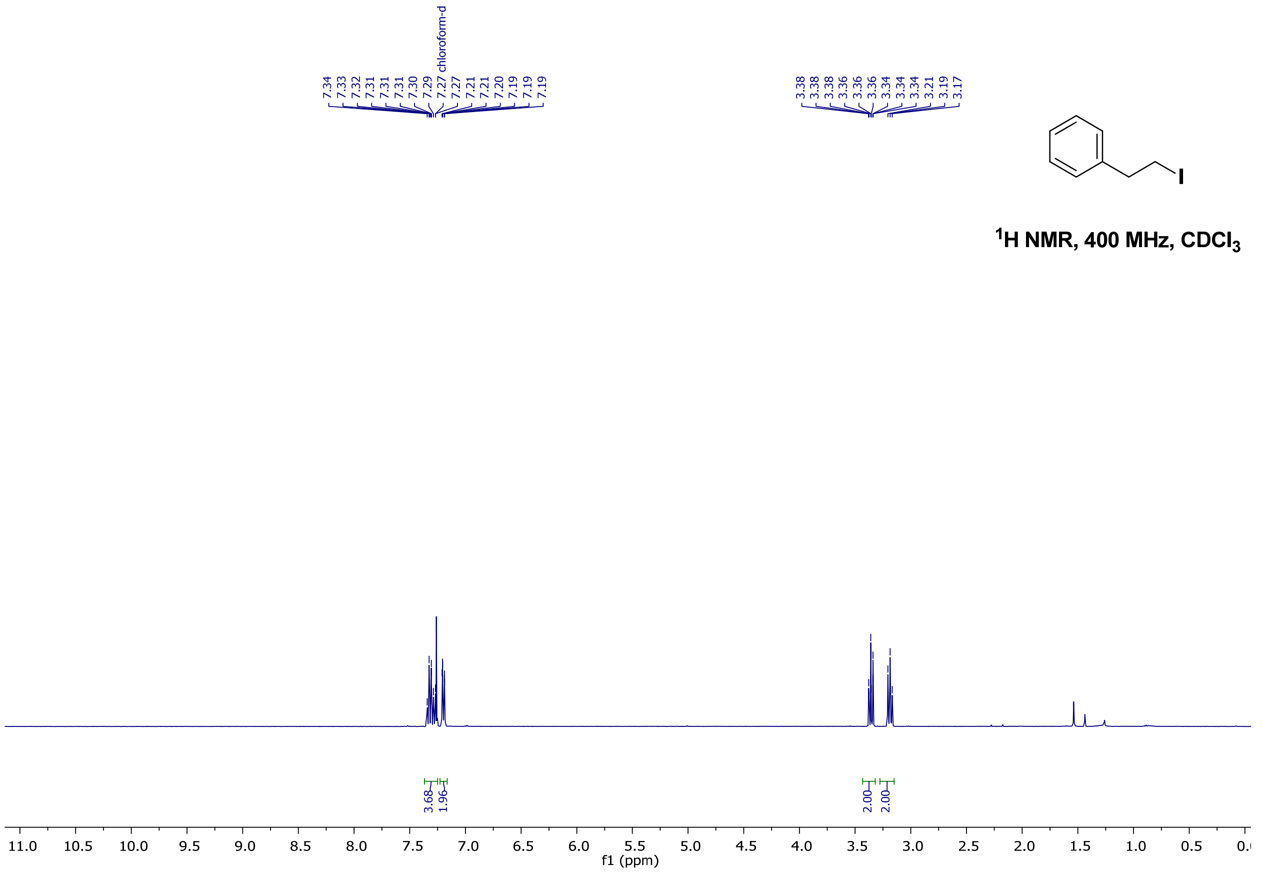

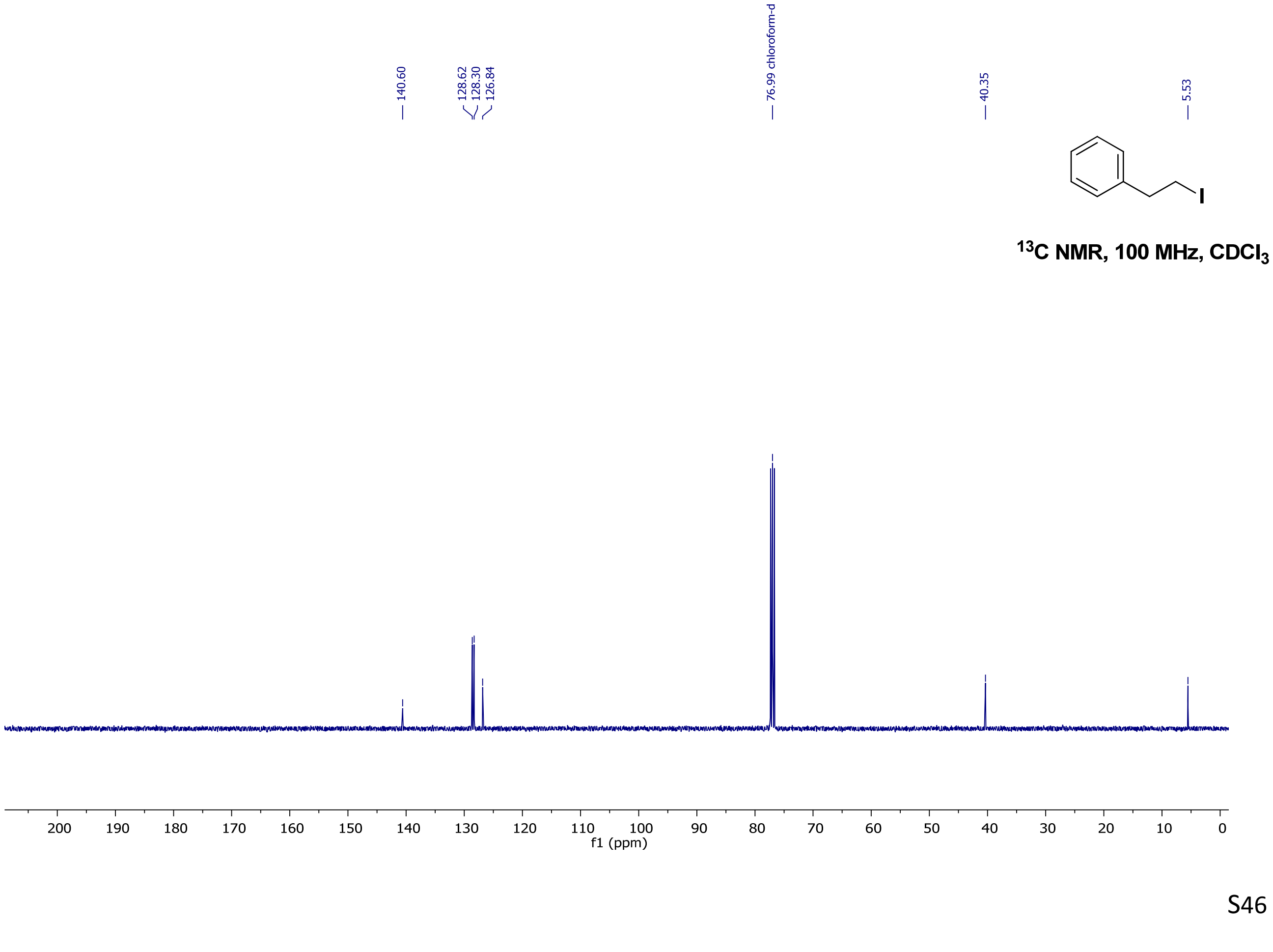

3-(Iodomethyl)cyclohexene (3)

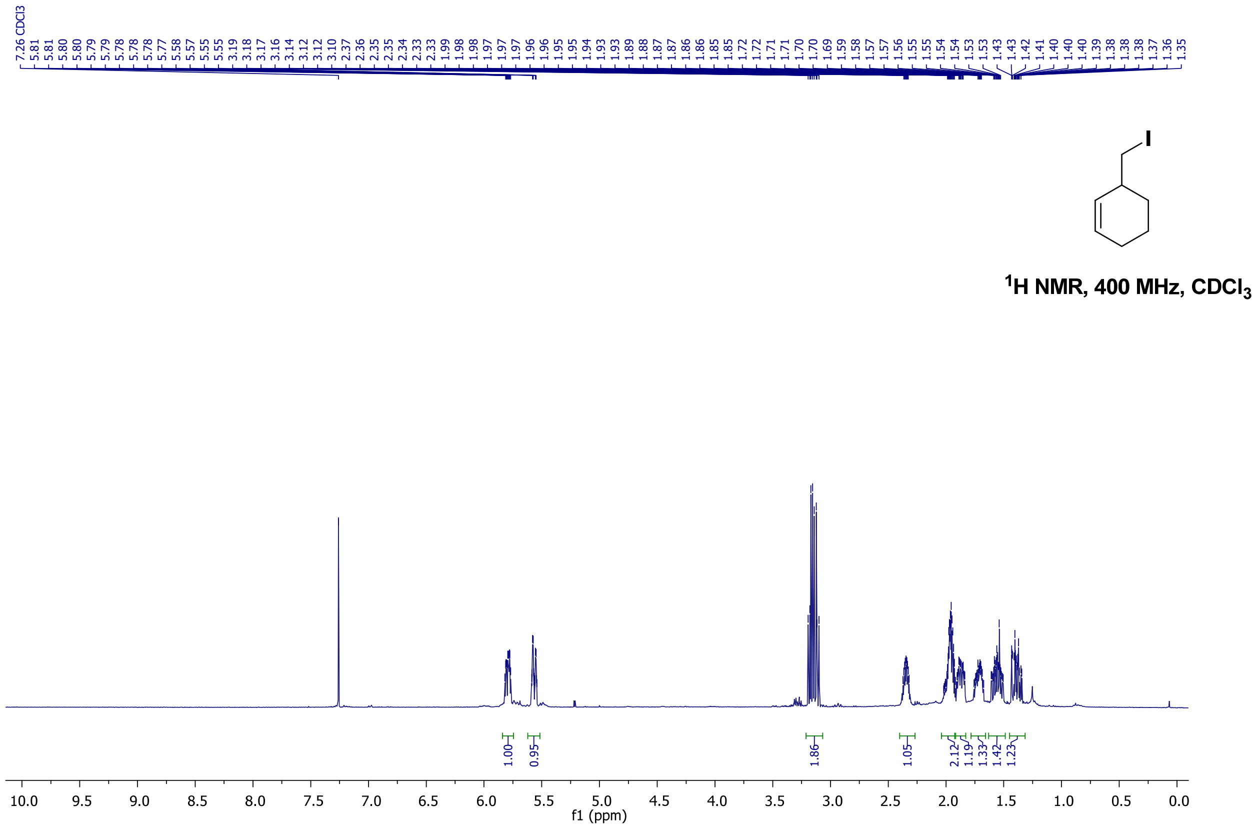

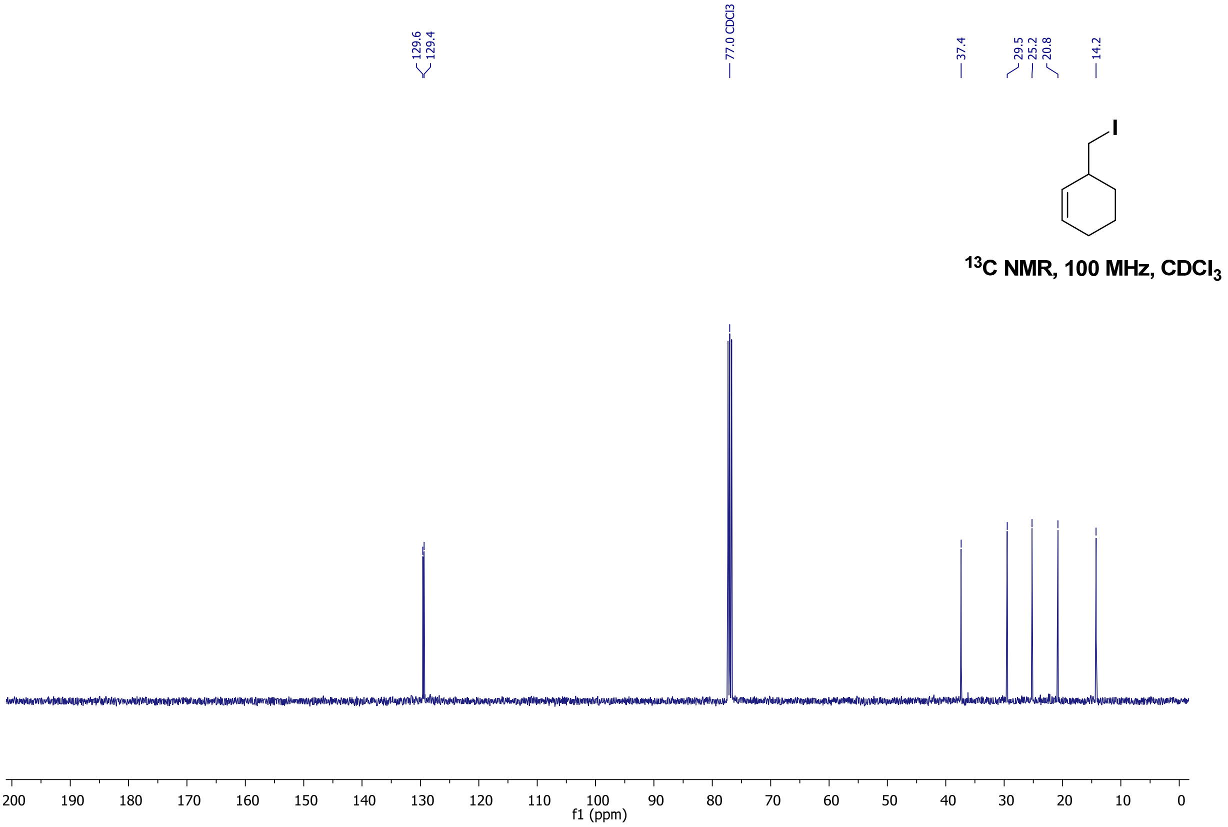

Ethyl 4-iodo-2-methylidenebutanoate (4)

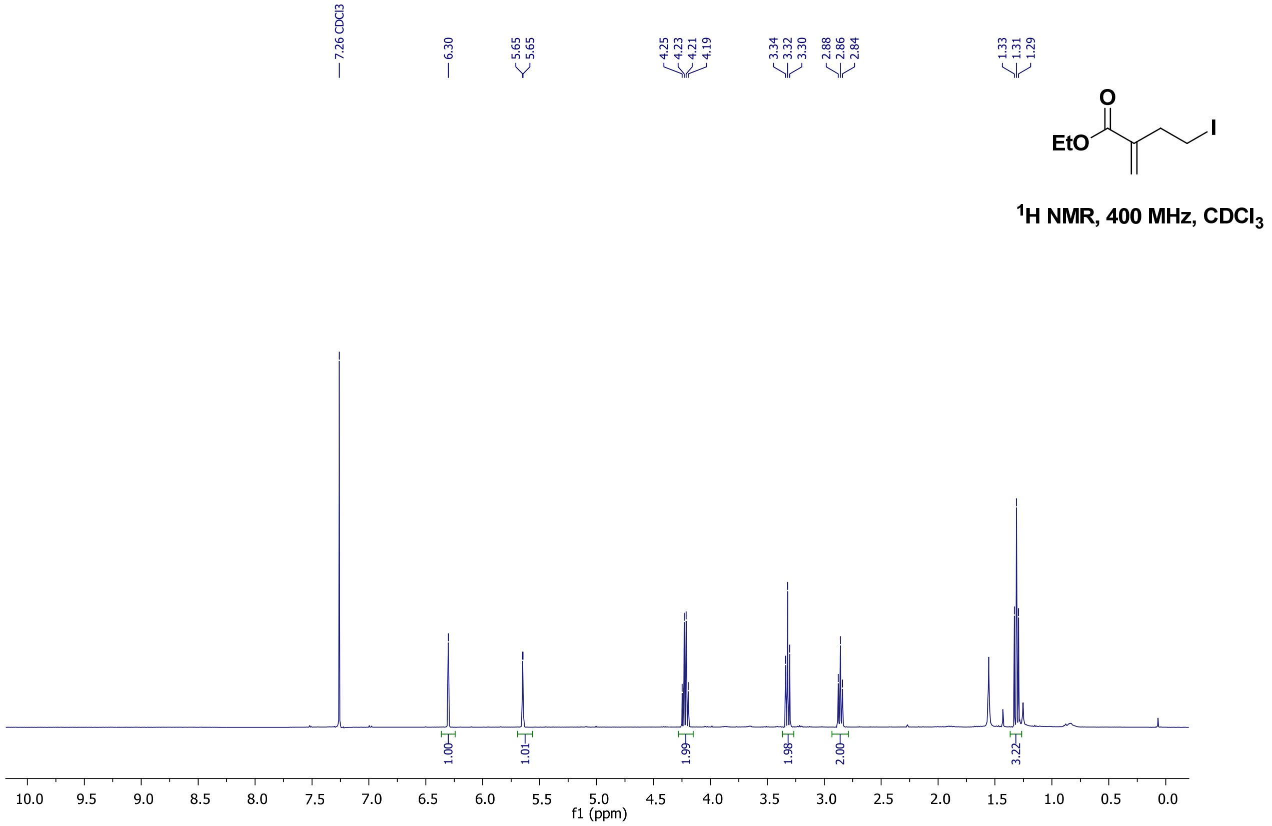

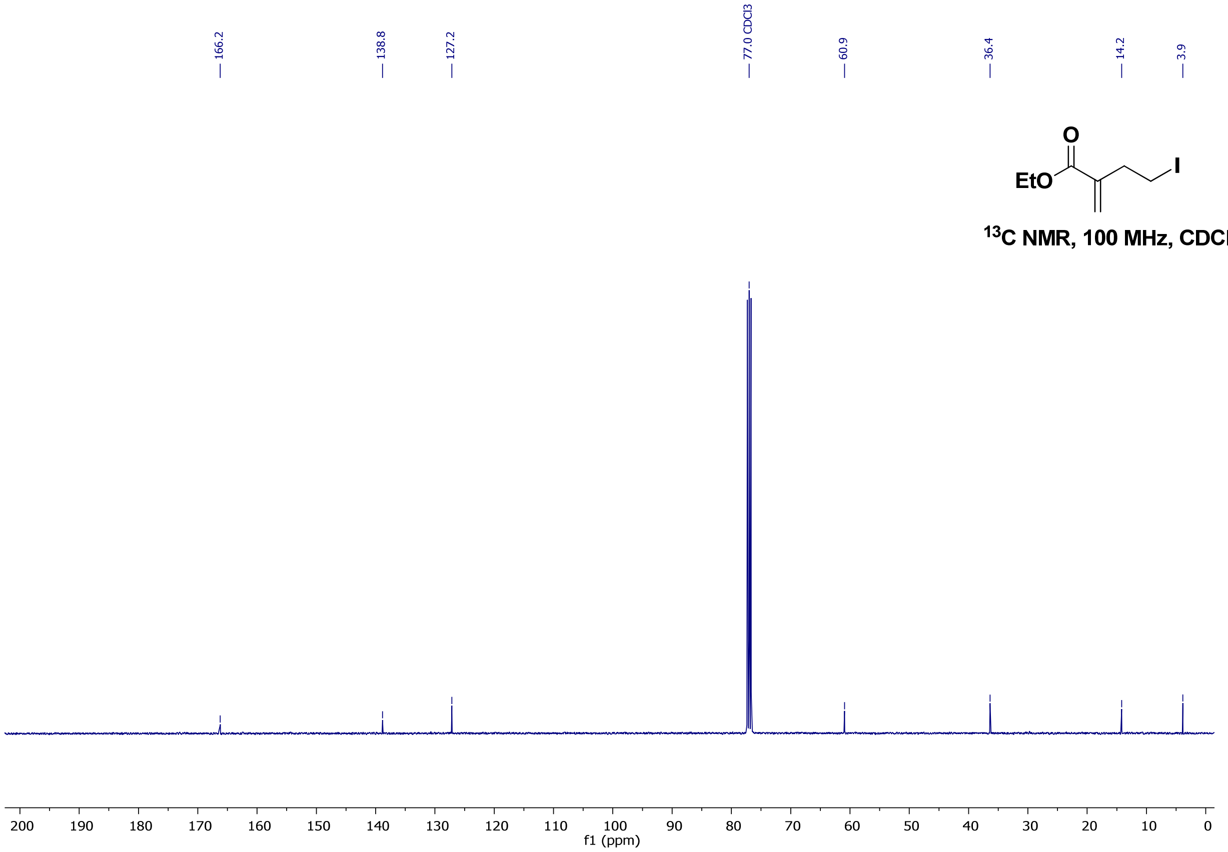

1-(2-Chloroethyl)-4-methylbenzene (5)

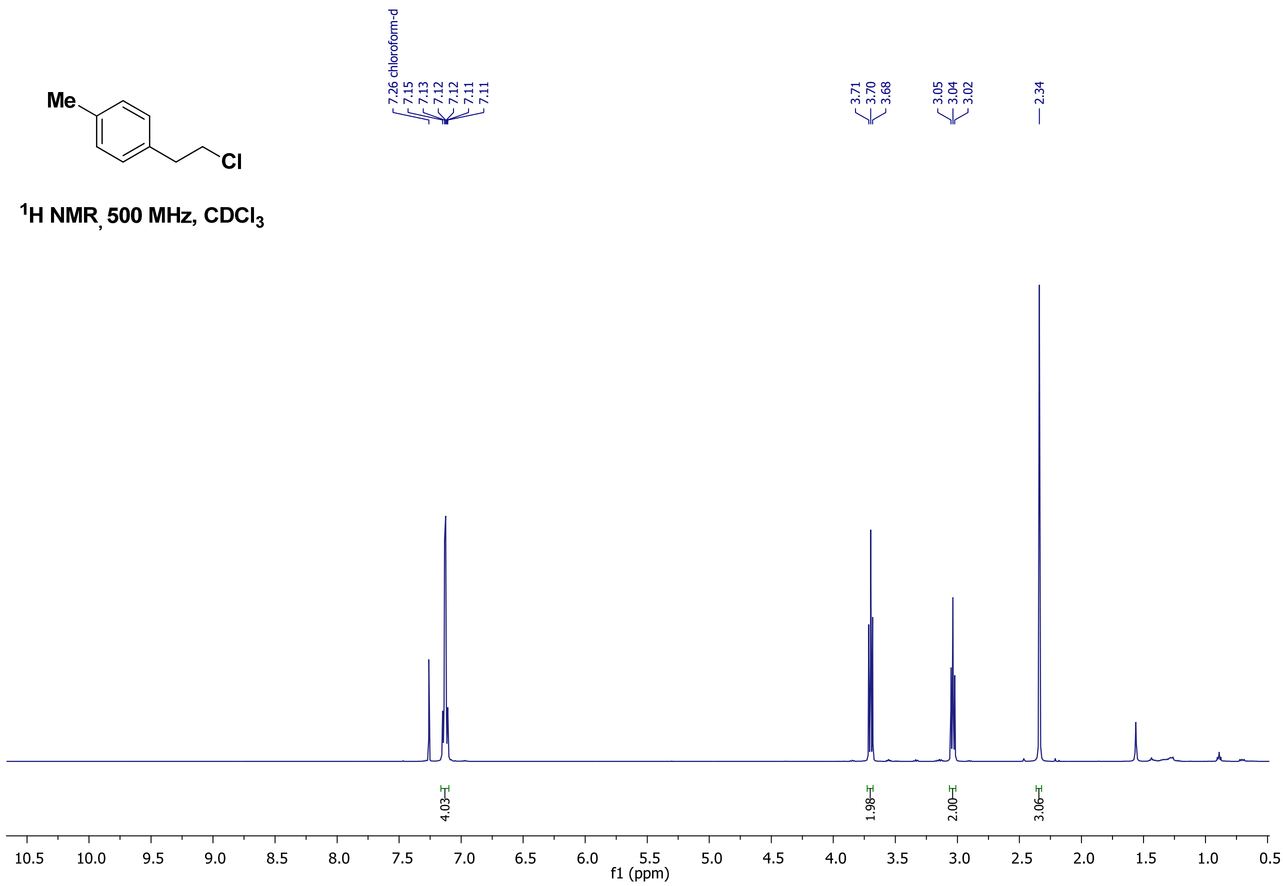

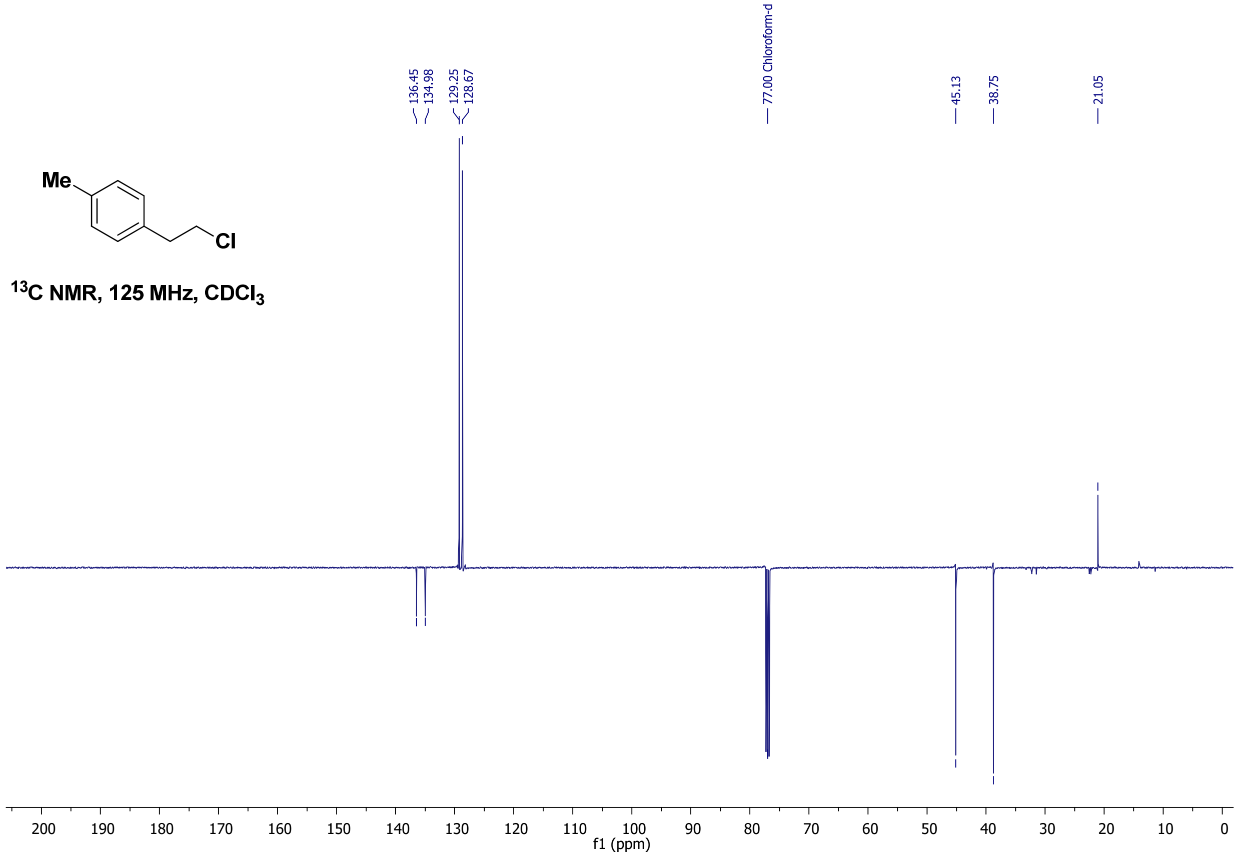

1-Chloro-4-(2-Chloroethyl)benzene (6)

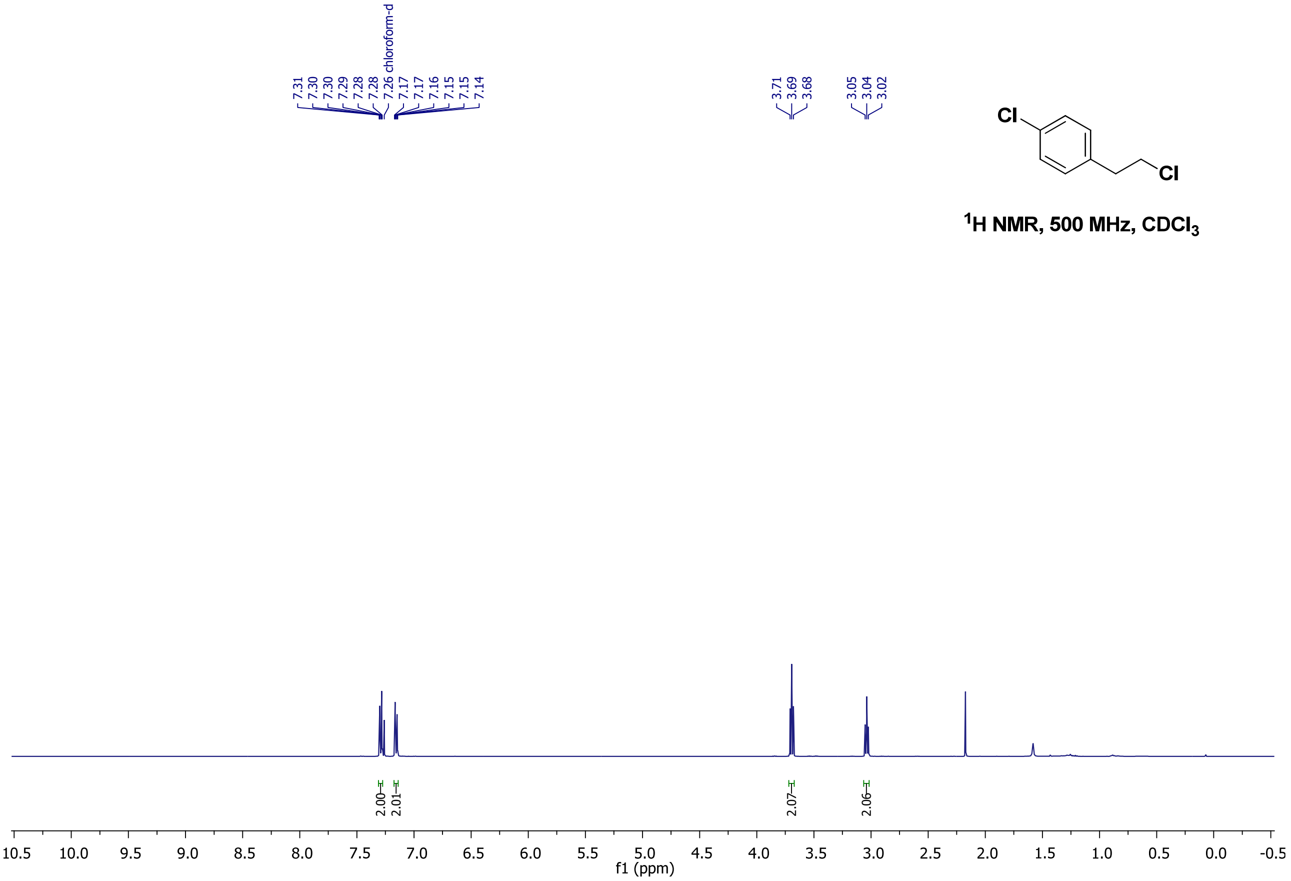

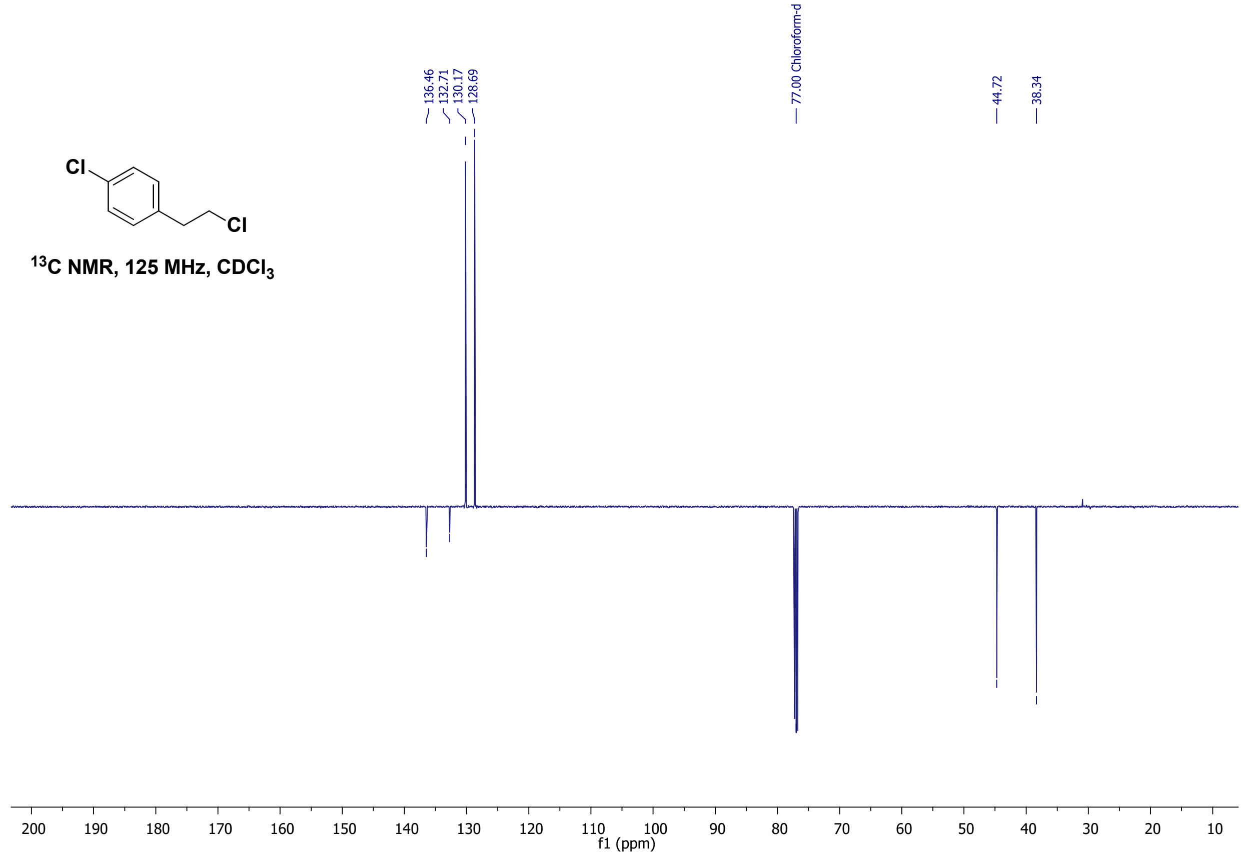

1-Chloro-4-(2-chloroethyl)-2-(trifluoromethyl)benzene (7)

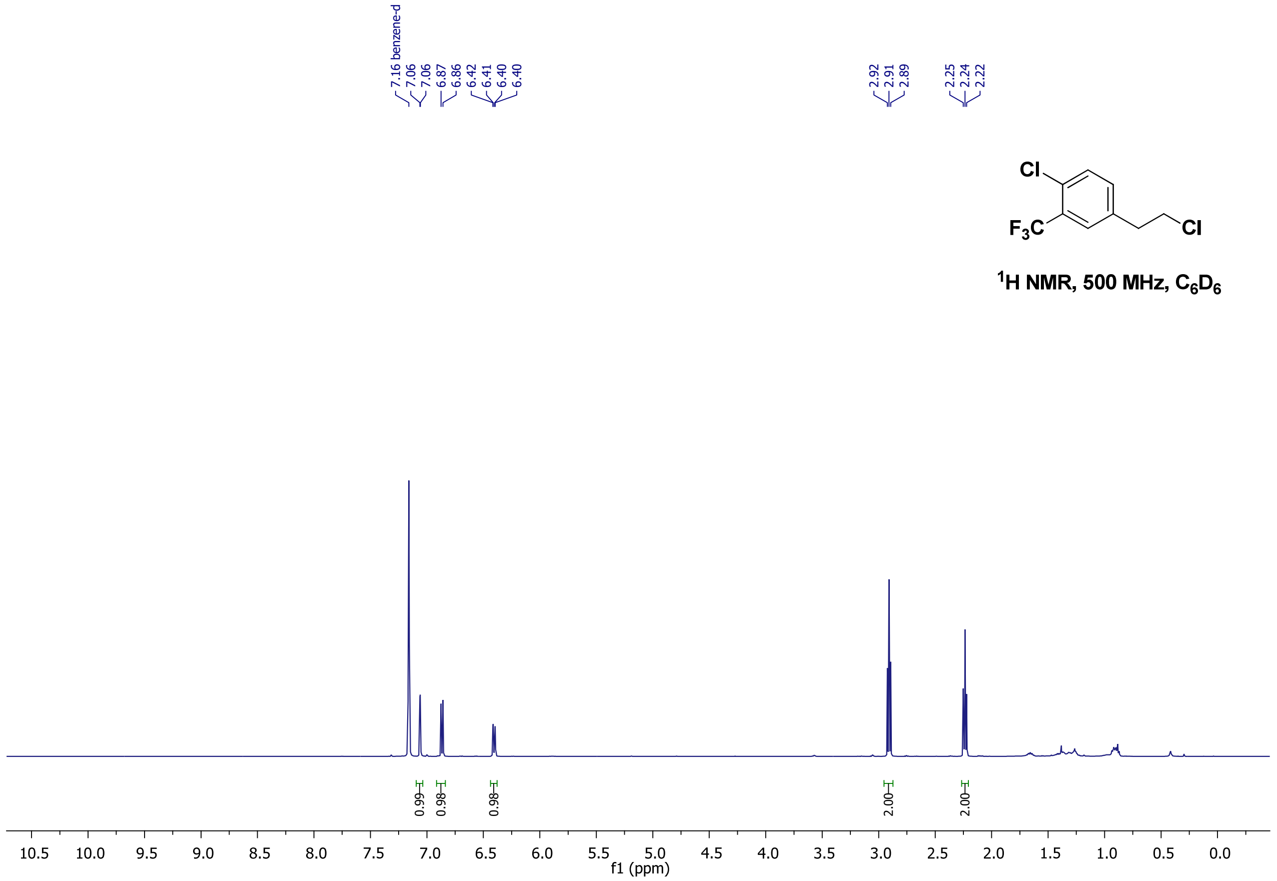

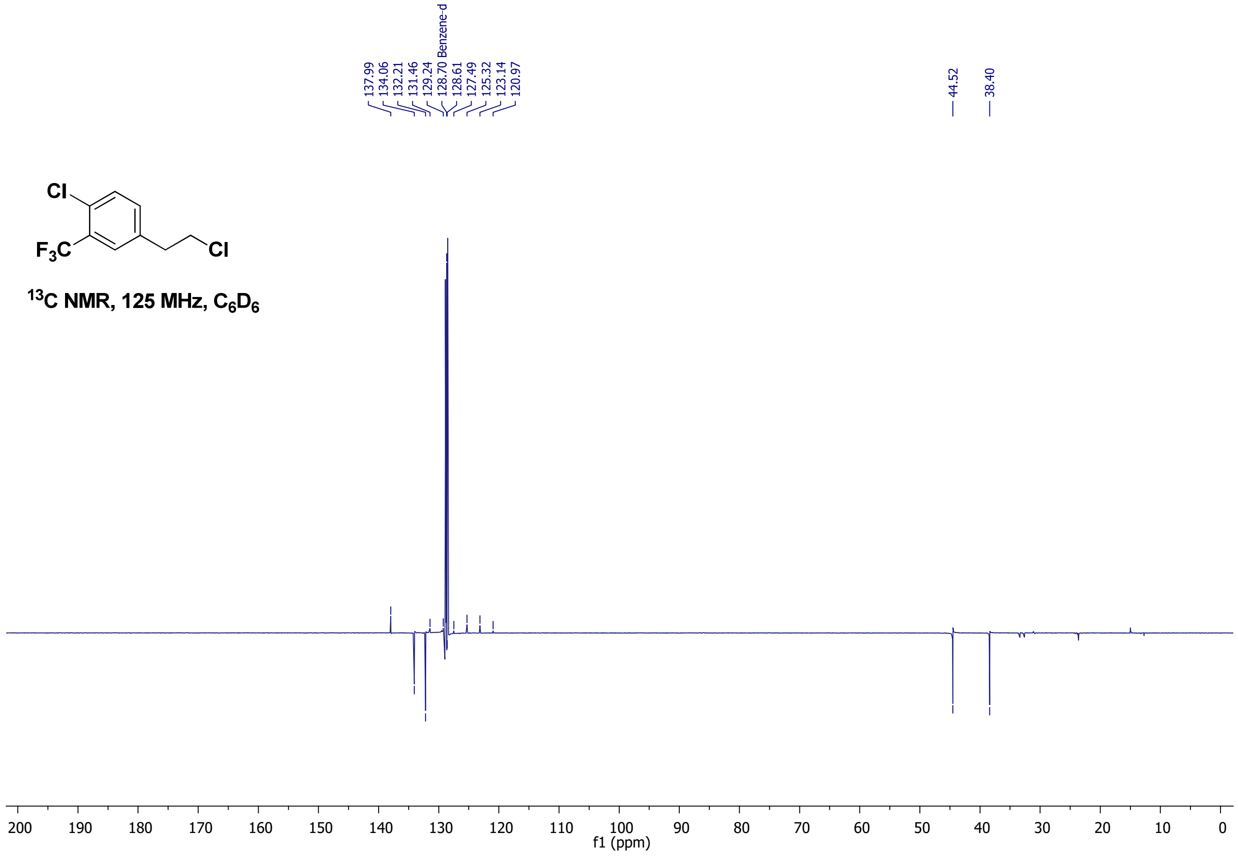

1-(2-Chloroethyl)-2-fluorobenzene (8)

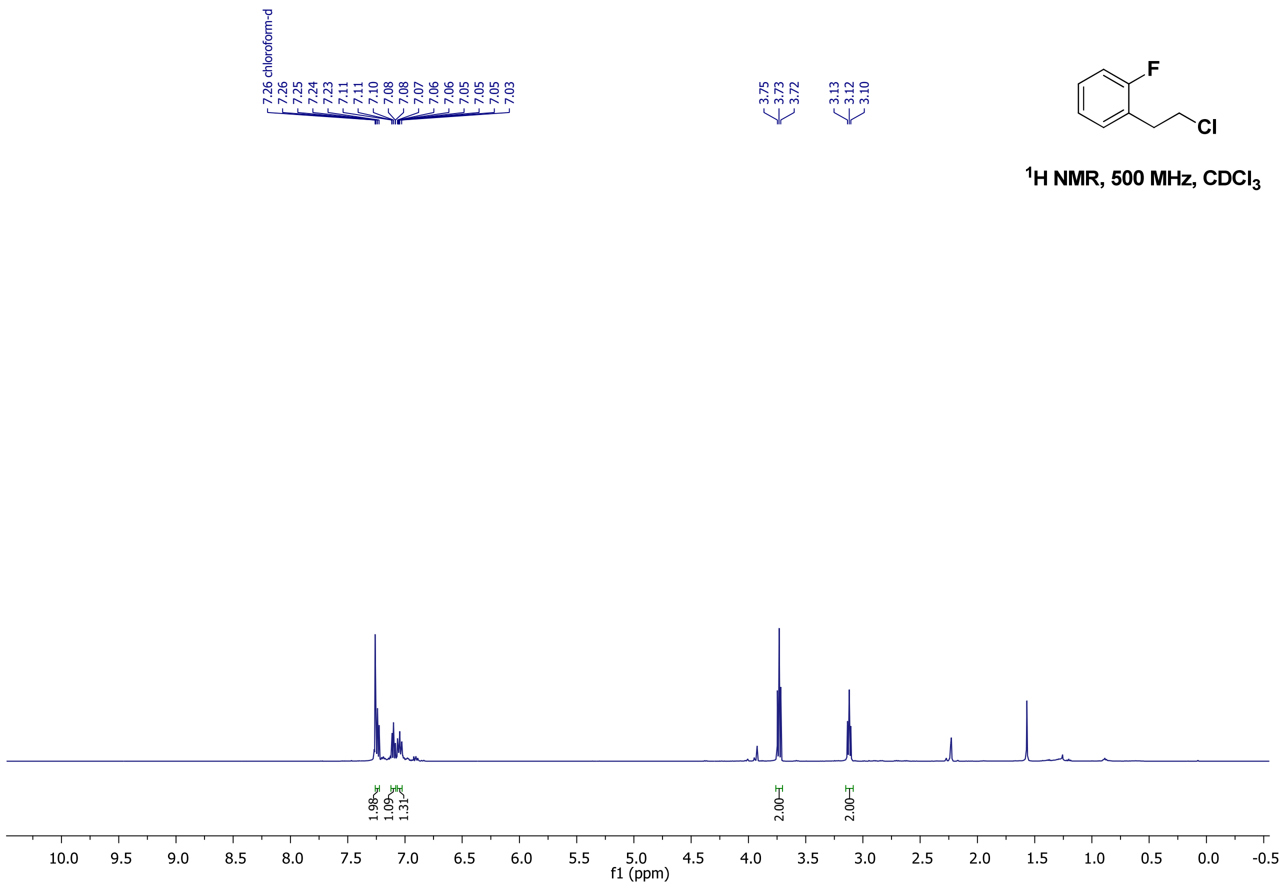

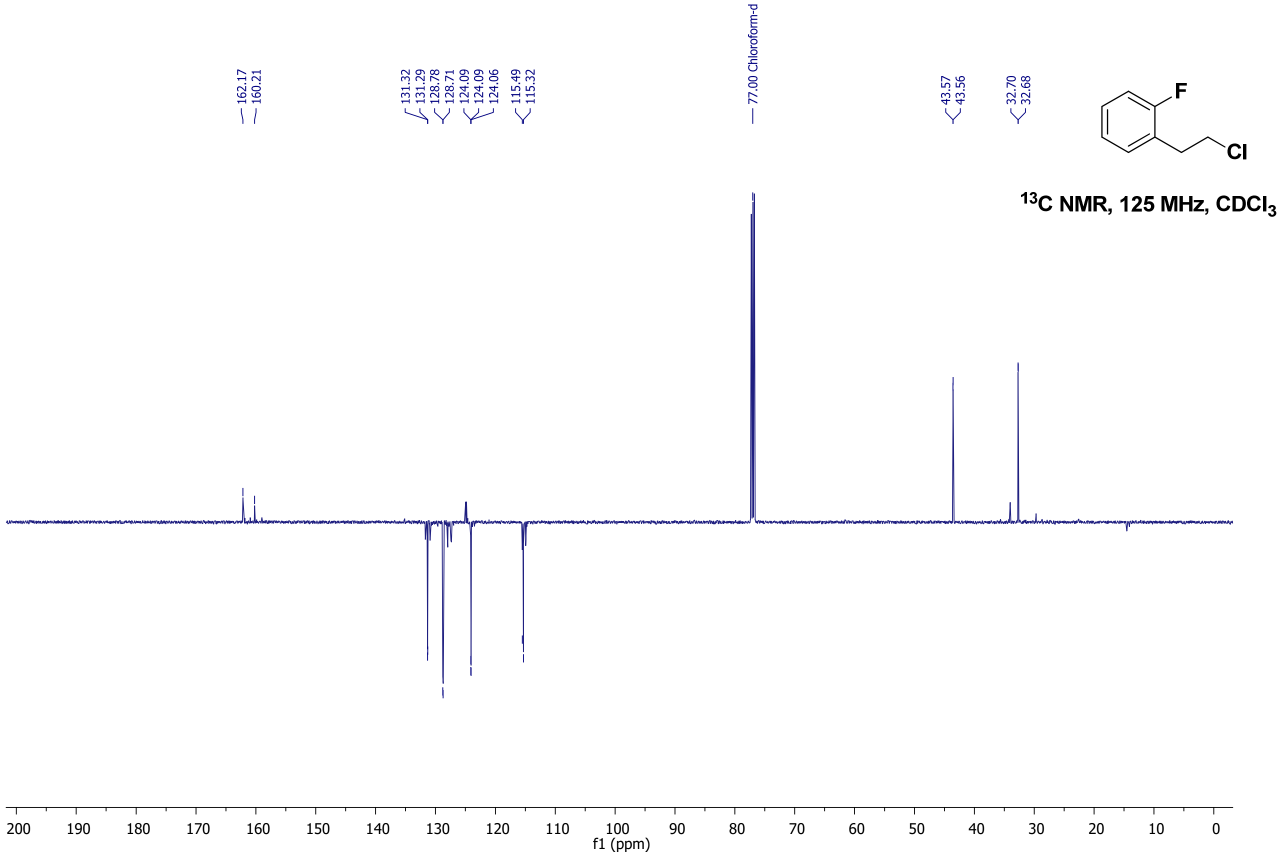

4-(2-Chloroethyl)-*N*,*N*-dimethylaniline (9)

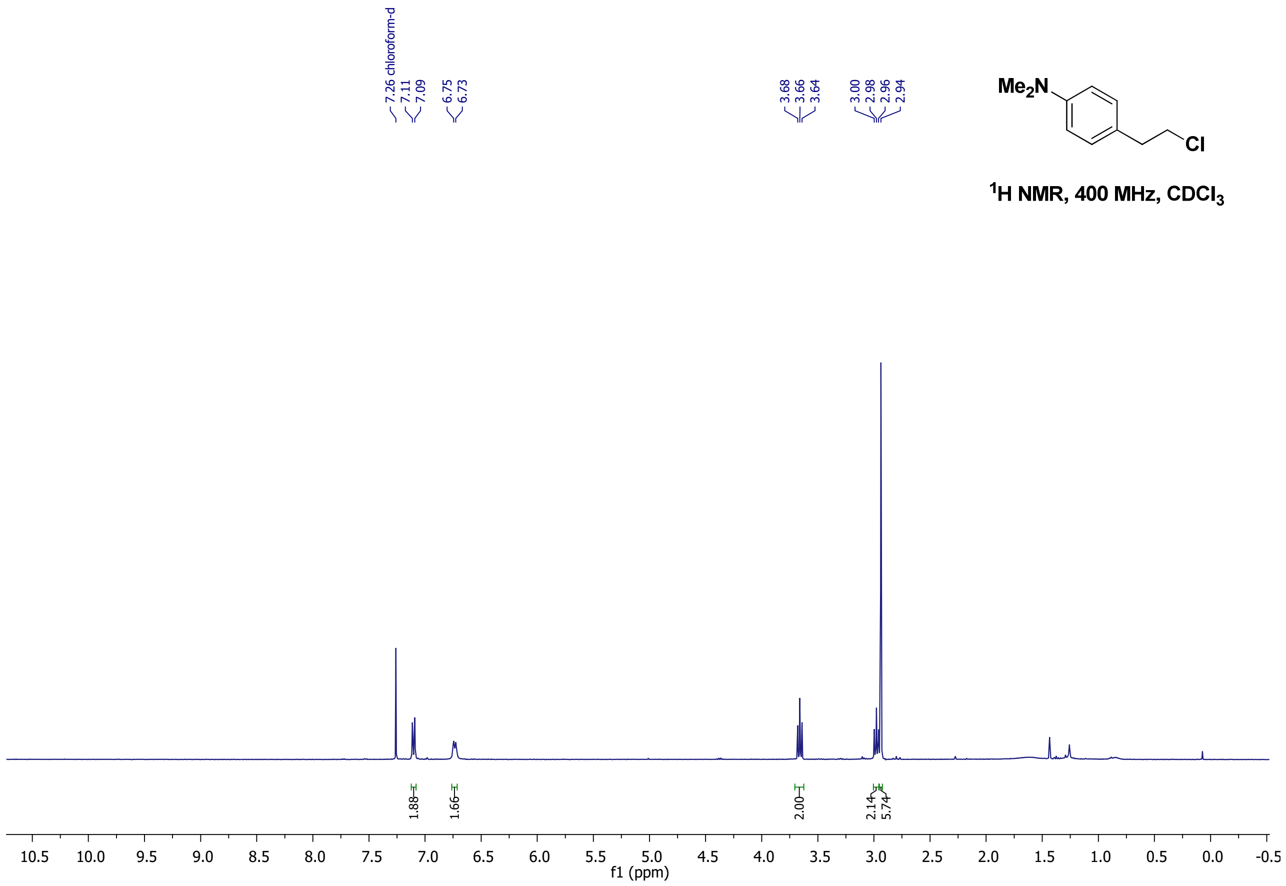

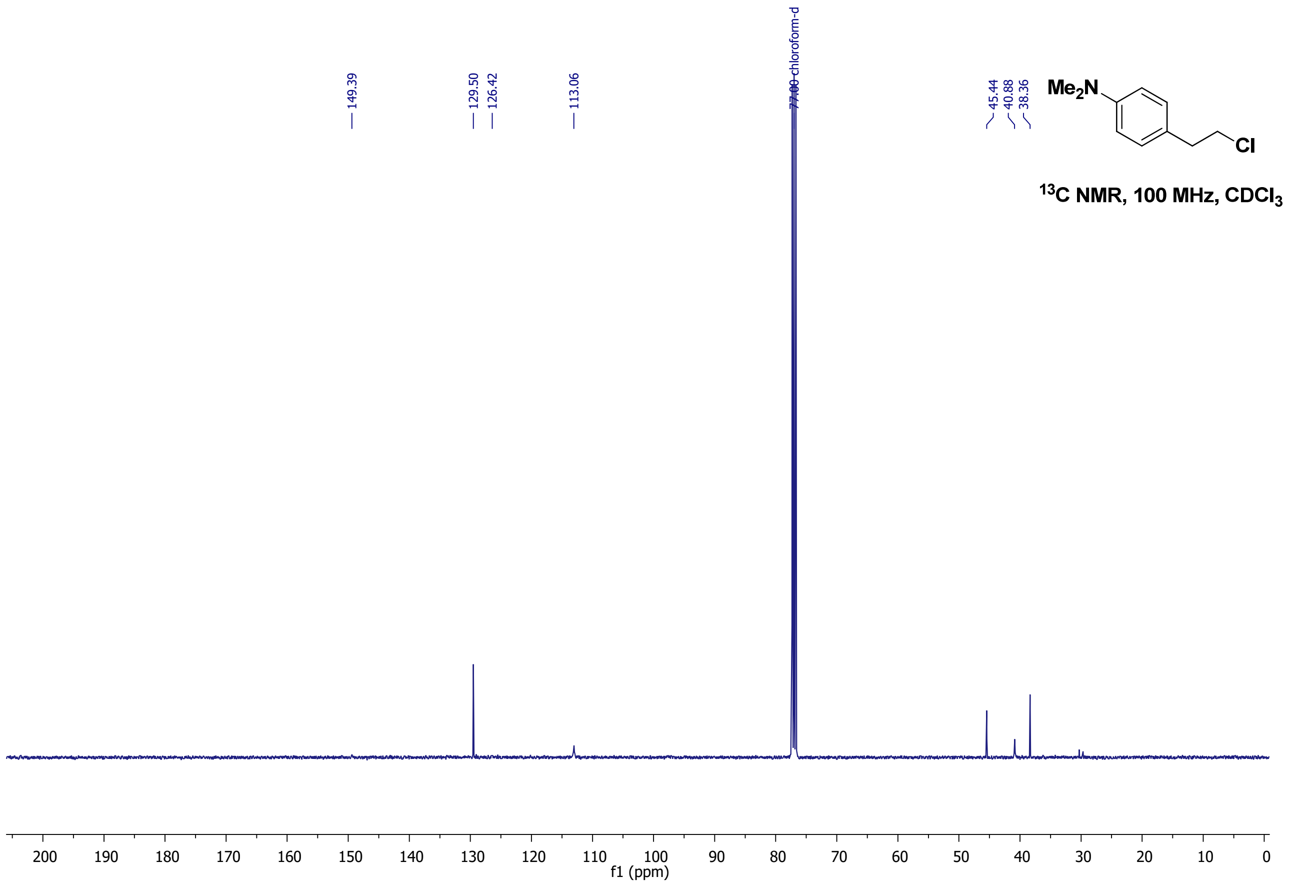

1-(2-Chloroethyl)naphthalene (10)

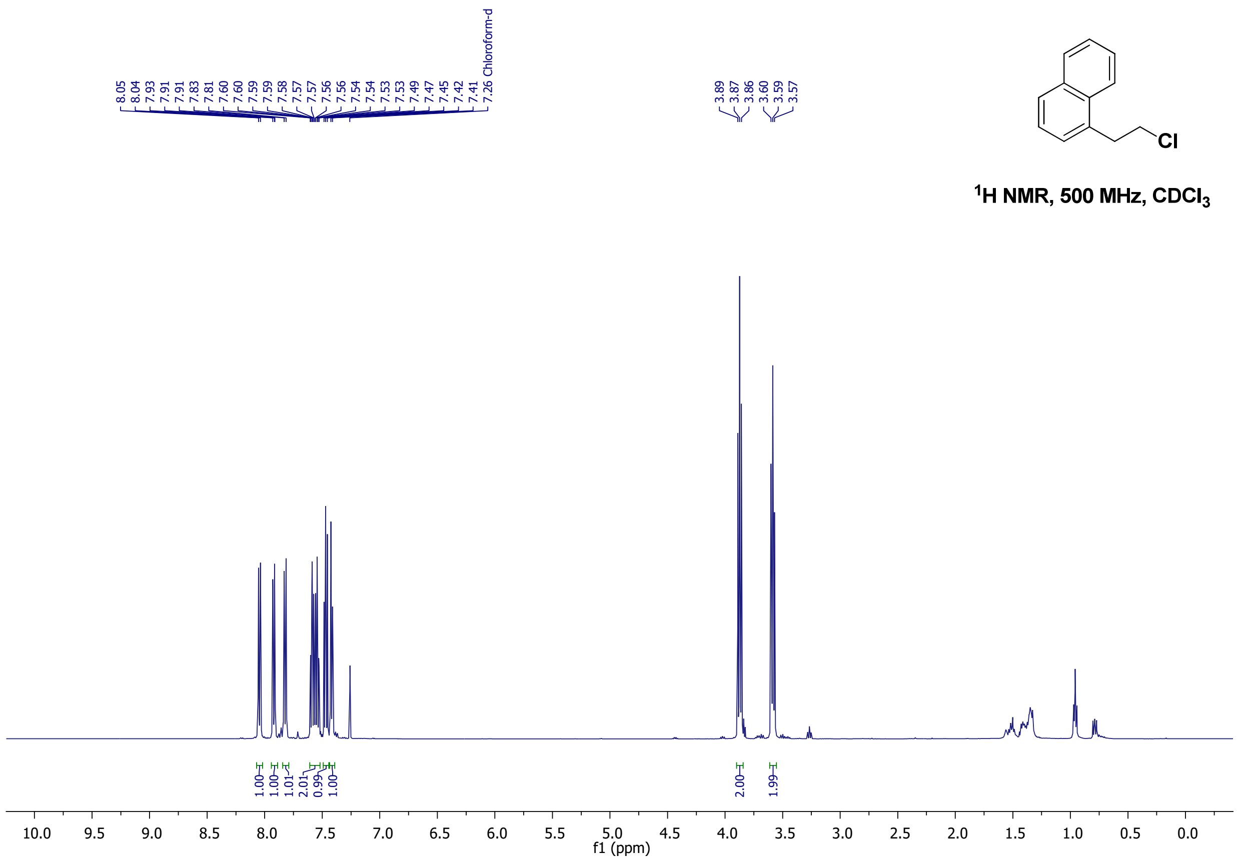

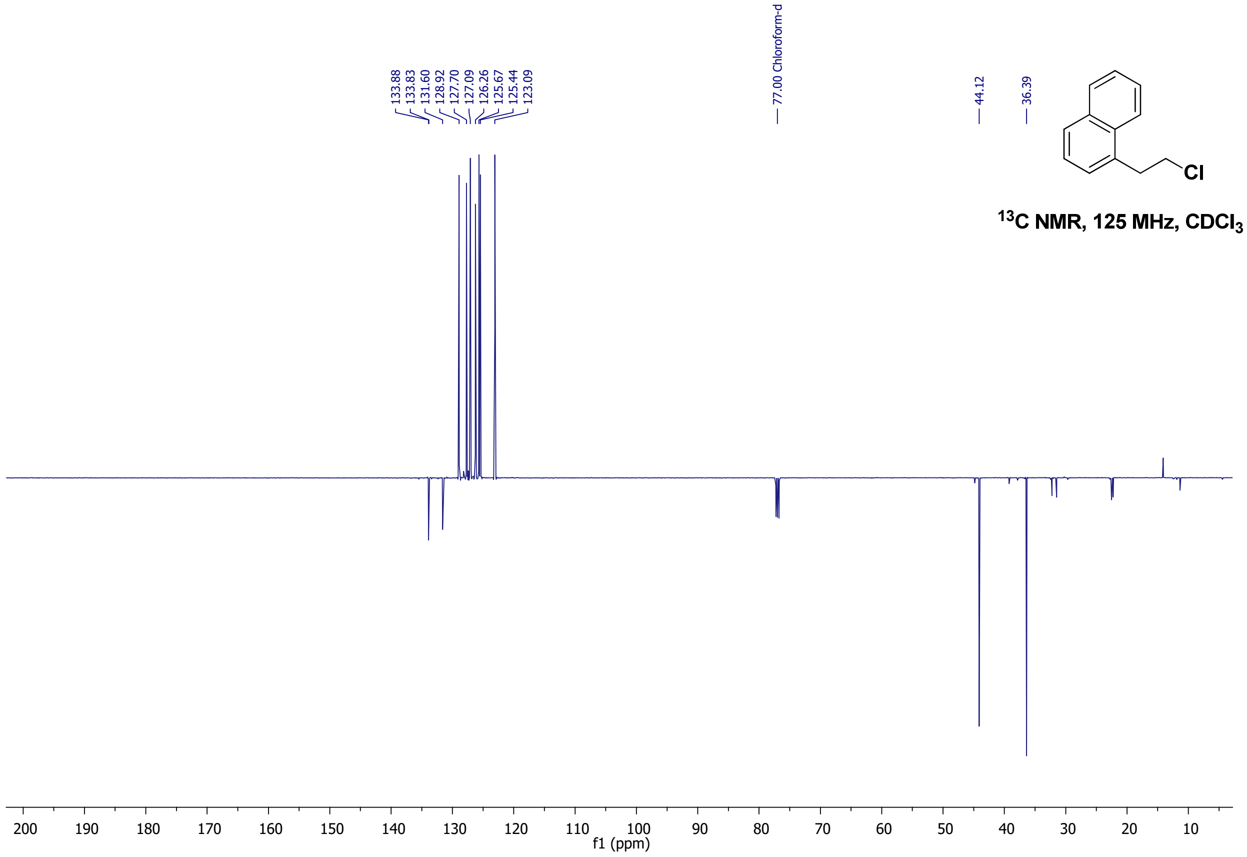

5-Bromo-4-(2-chloroethyl)-1,3-benzodioxole (11)

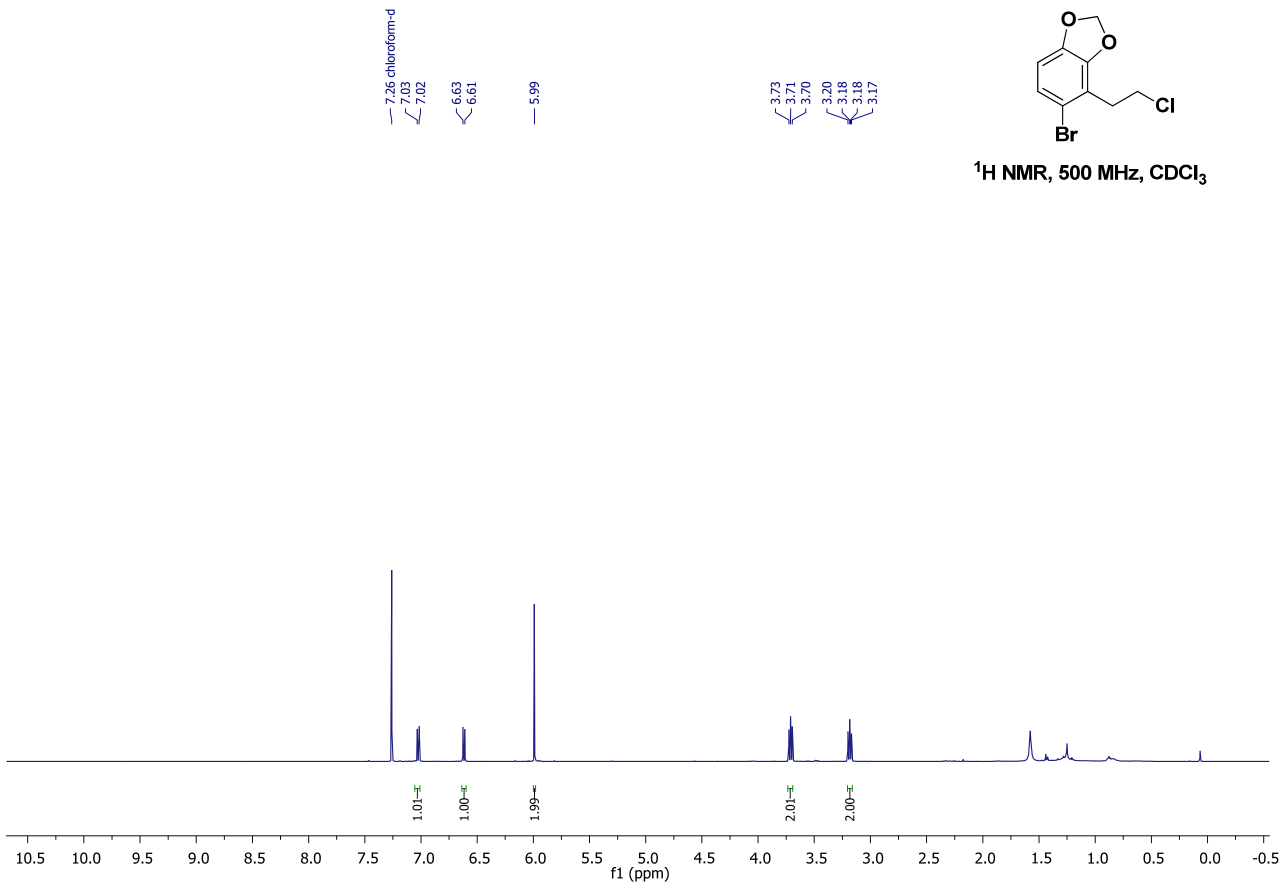

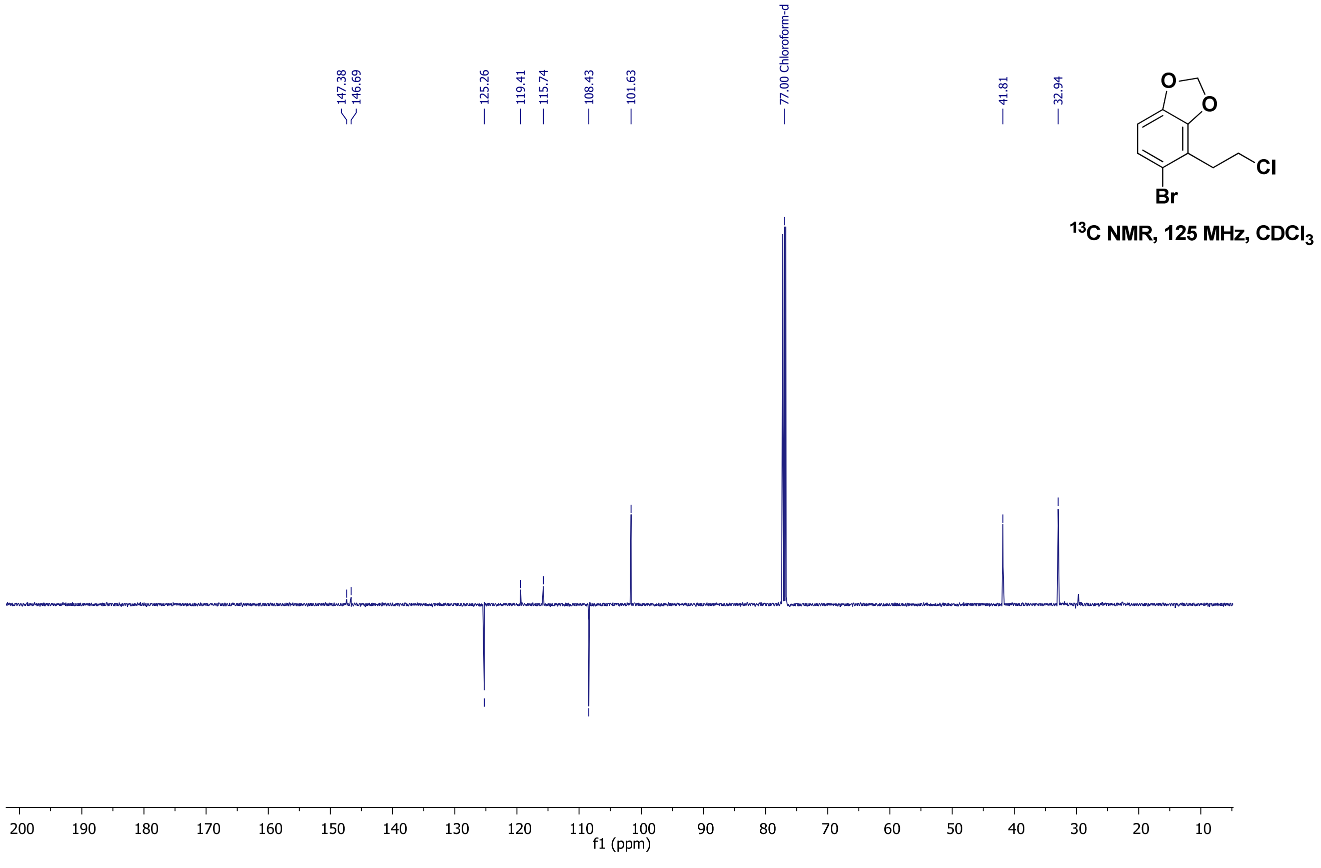

2,4-Dichloro-1-(2-chloroethyl)benzene (12)

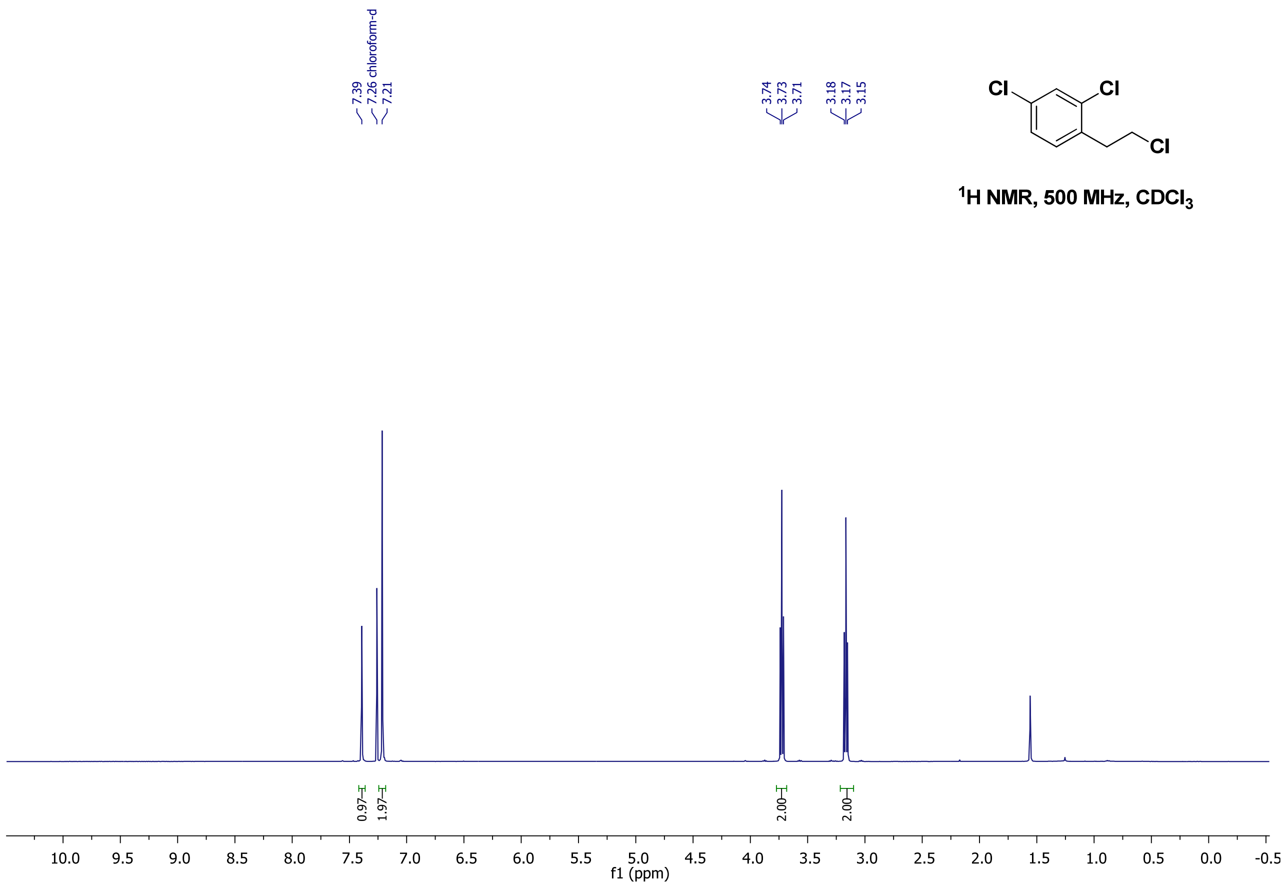

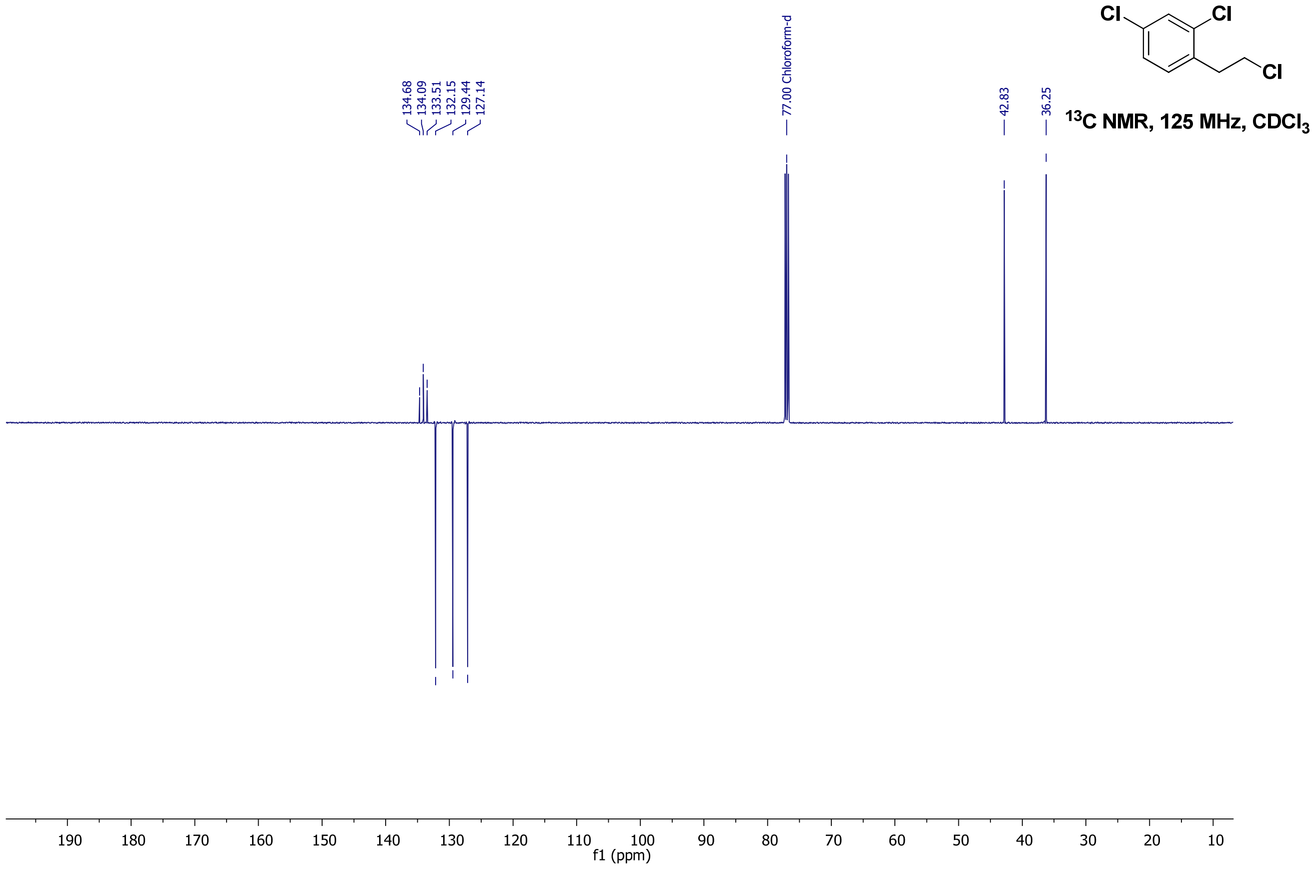

2-Chloro-1-(2-chloroethyl)-4-methylbenzene (13)

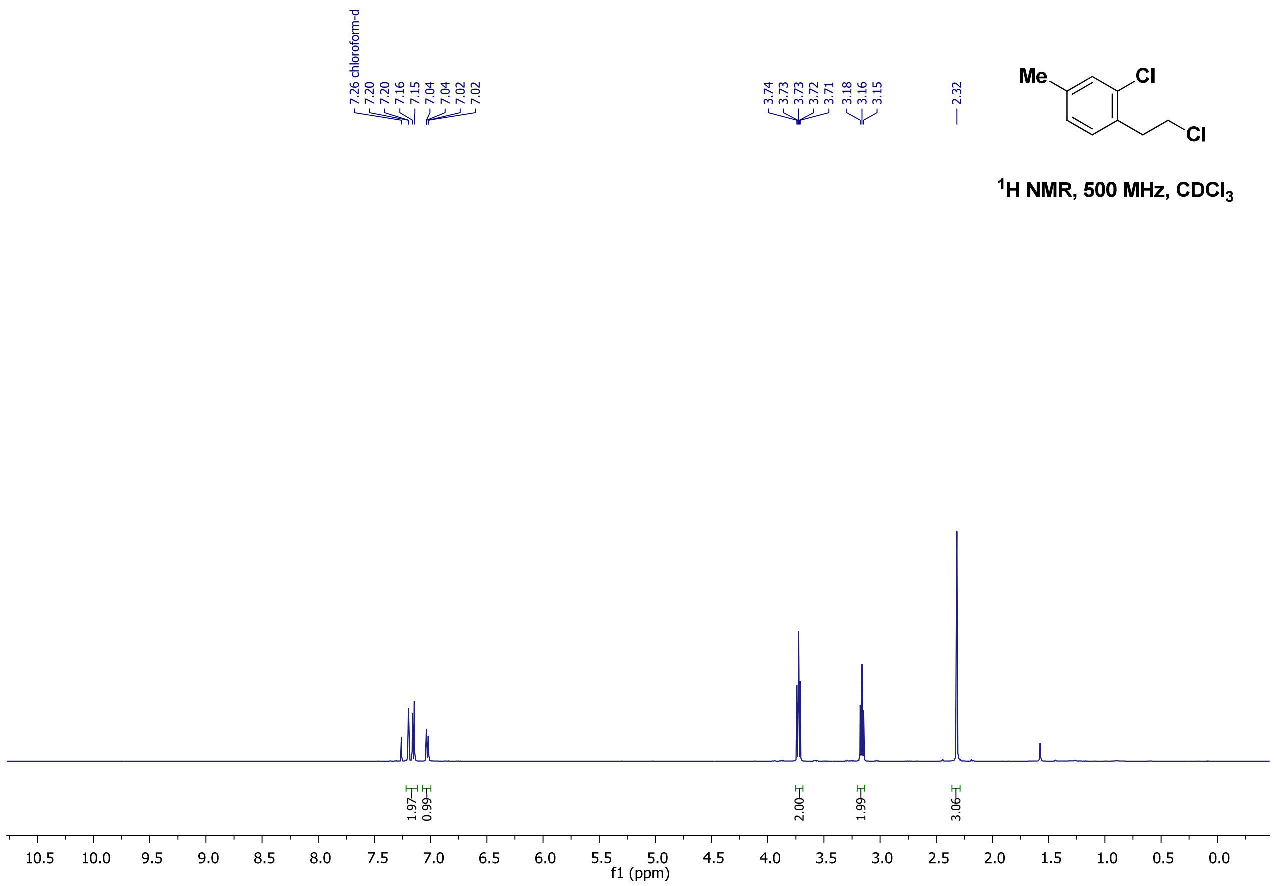

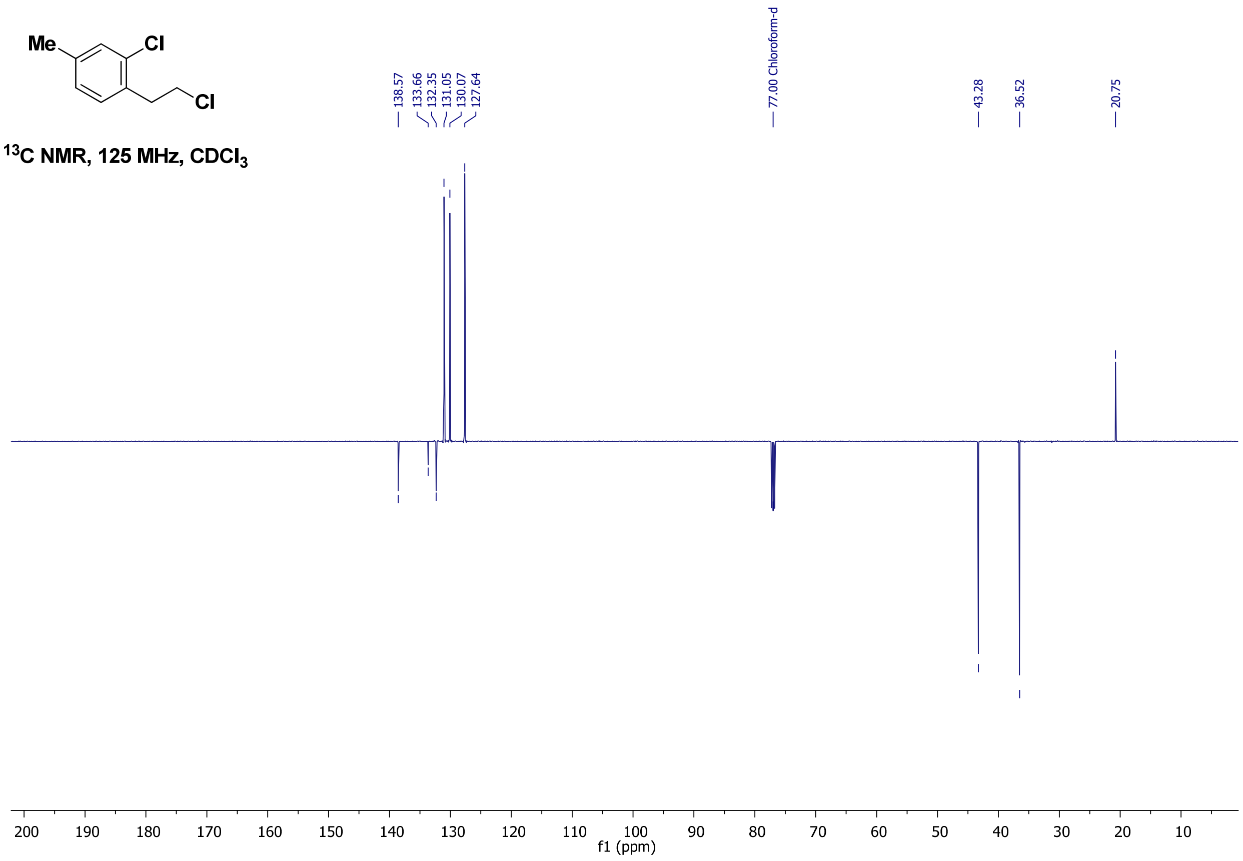

1-(2-Chloroethyl)-2-(trifluoromethoxy)benzene (14)

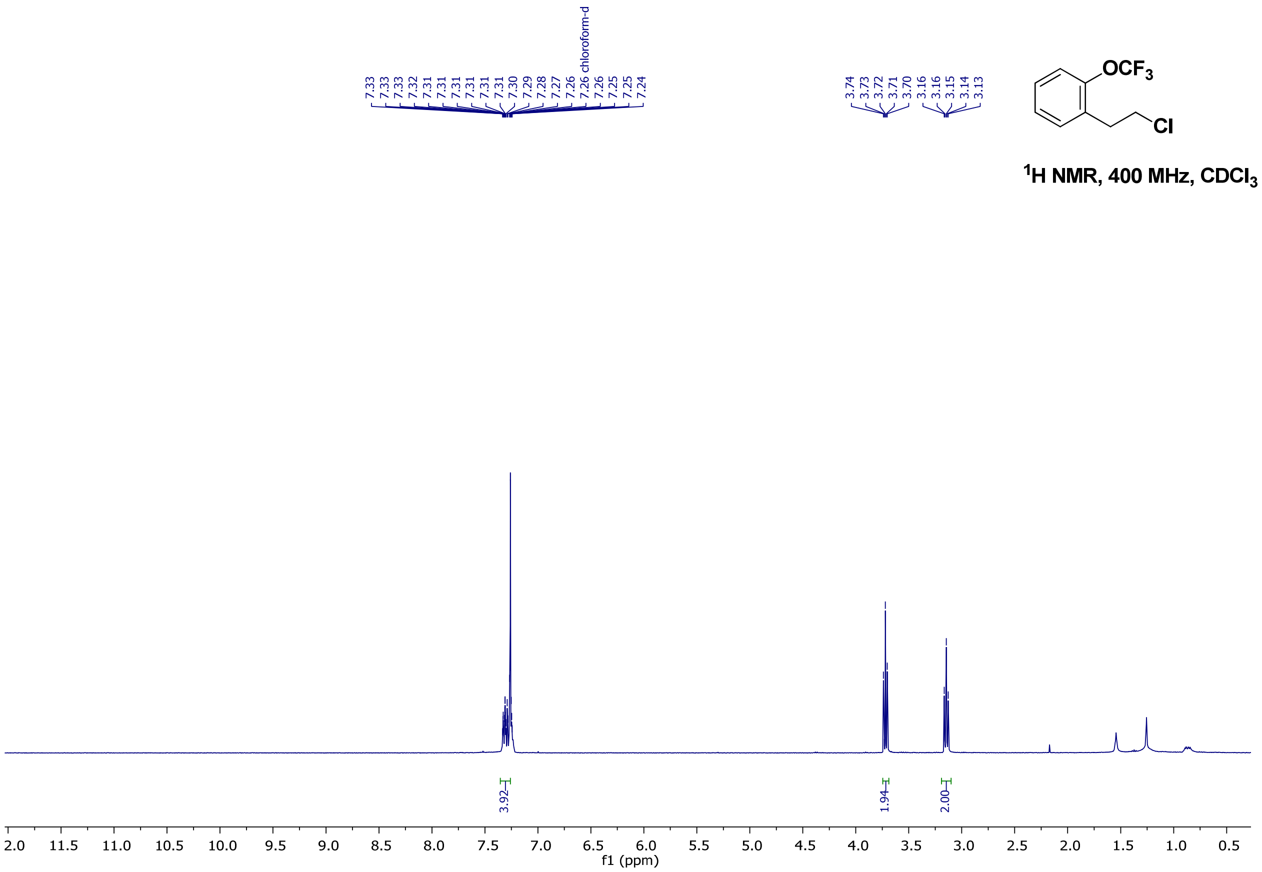

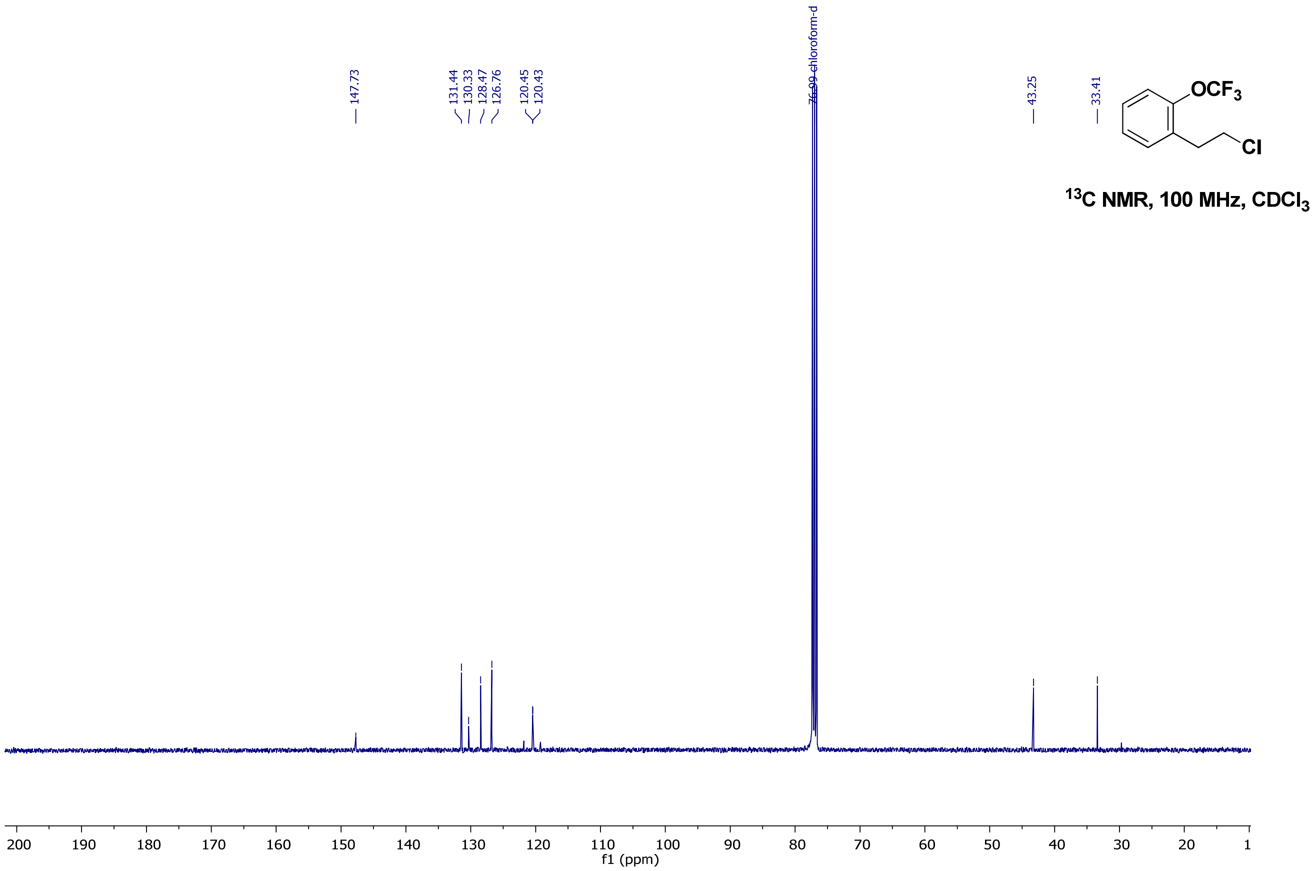

2-(2-Chloroethyl)-1,3-dimethylbenzene (15)

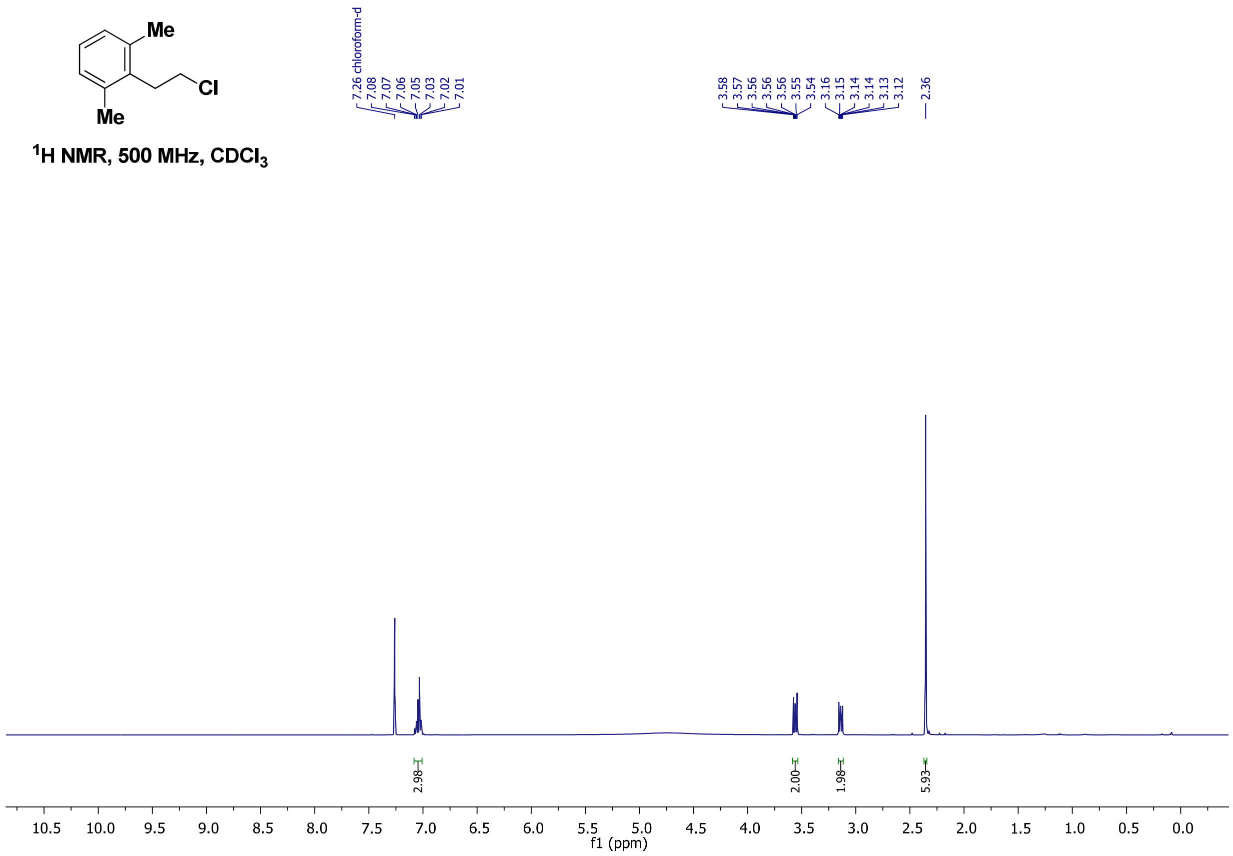

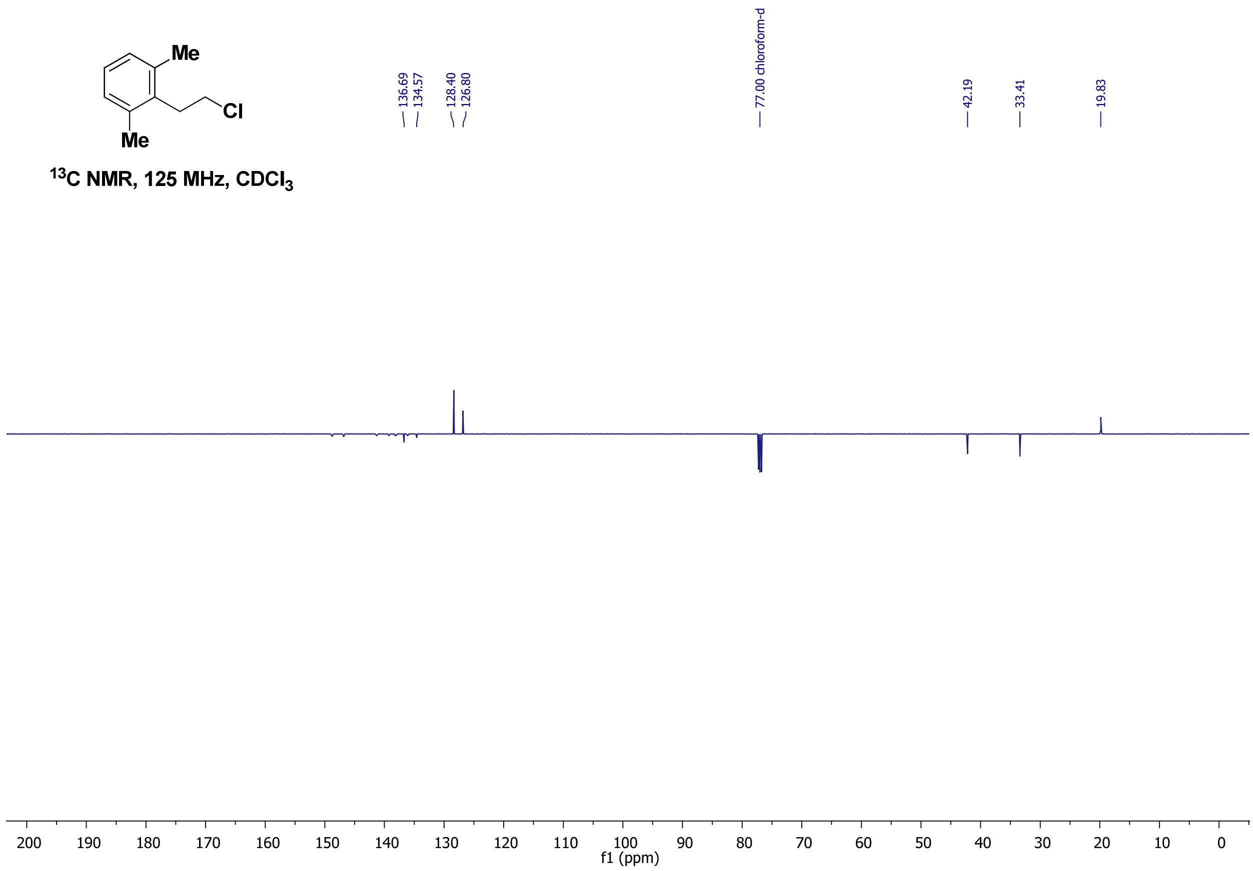

1,3-Dichloro-2-(2-chloroethyl)benzene (16)

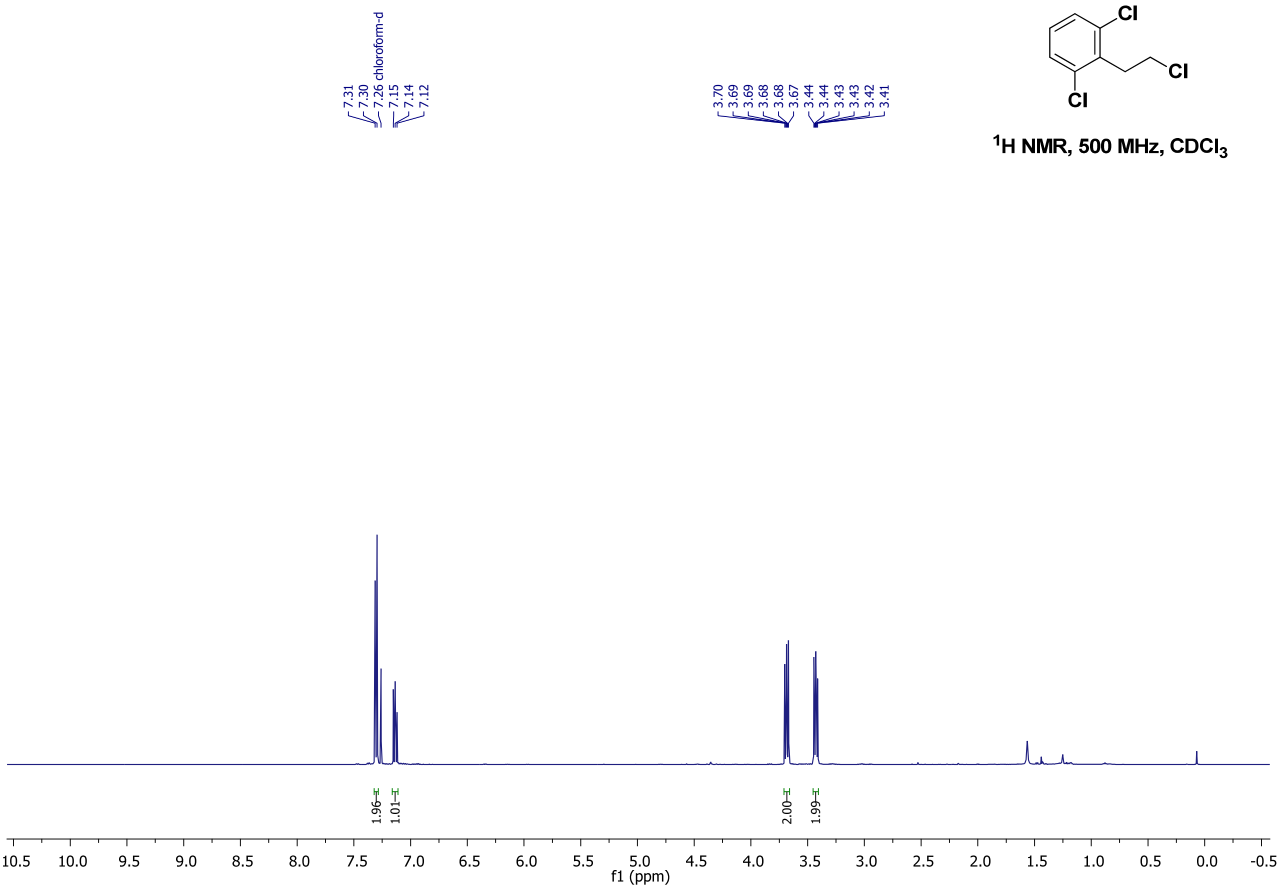

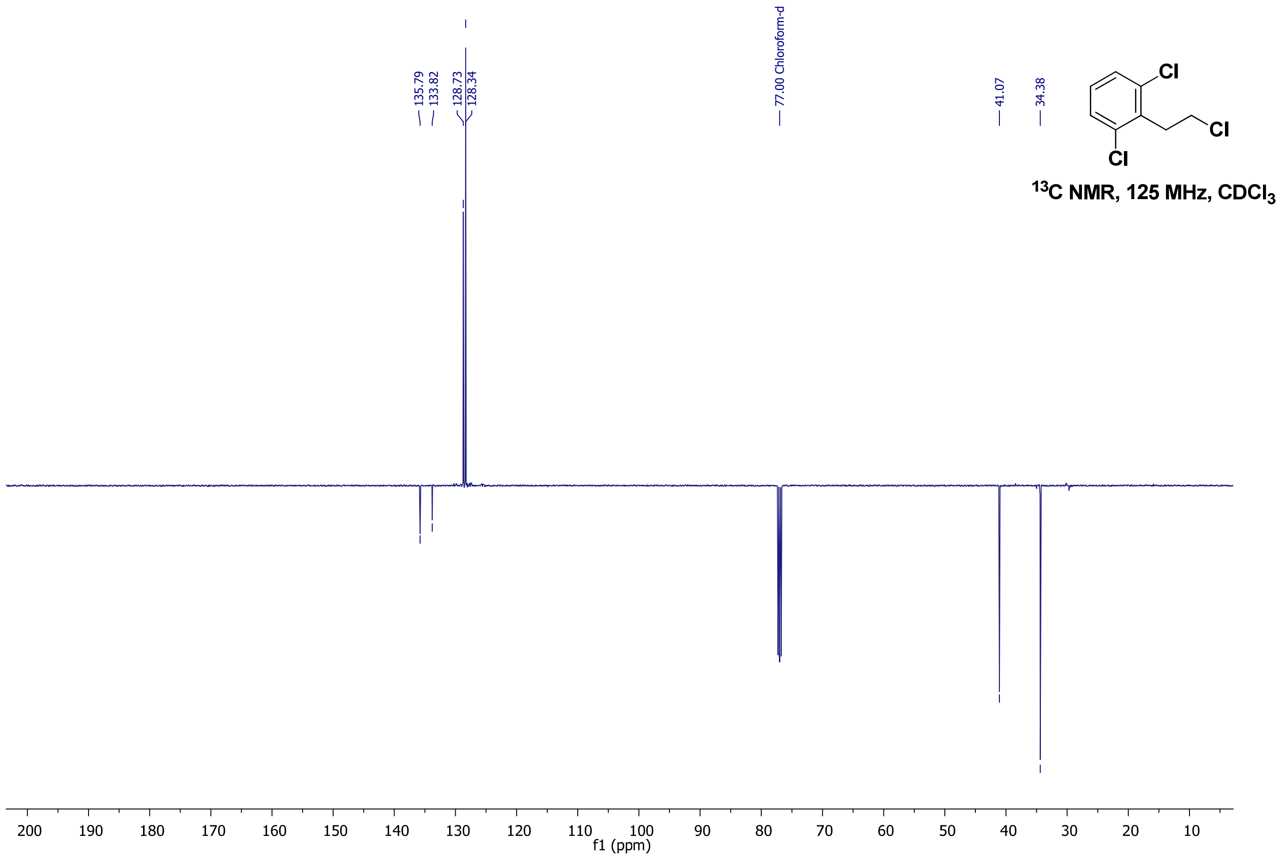

(3-Chloropropyl)benzene (17)

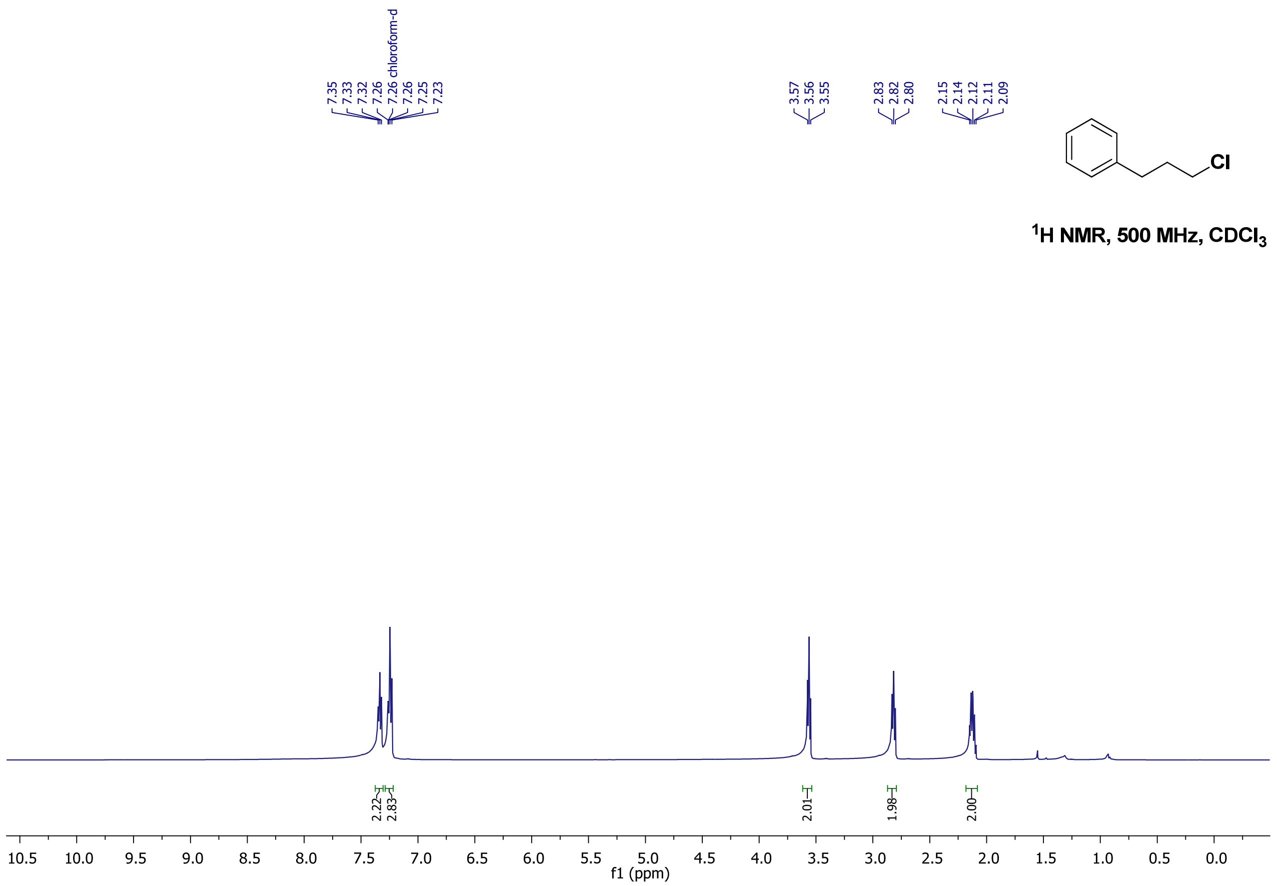

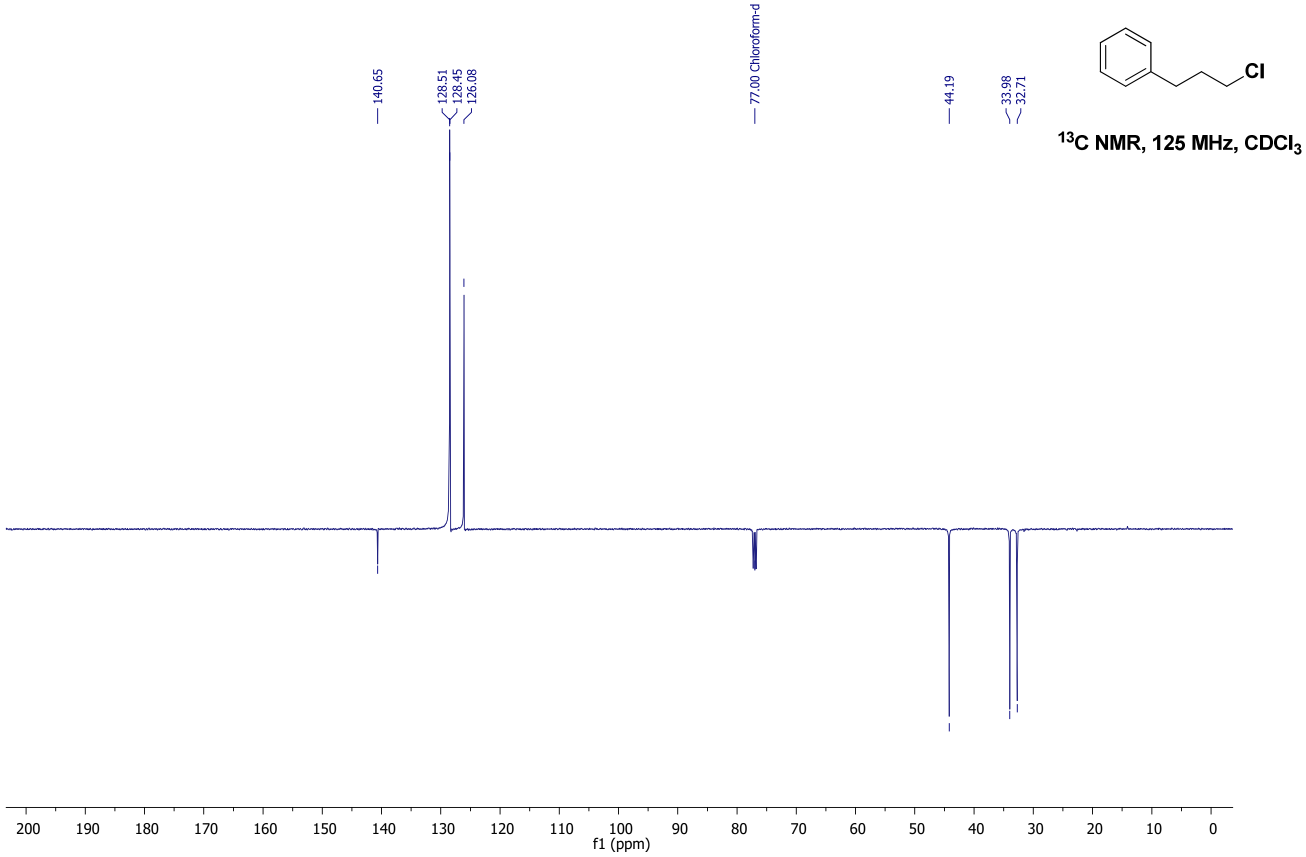

(4-Chlorobutyl)benzene (18)

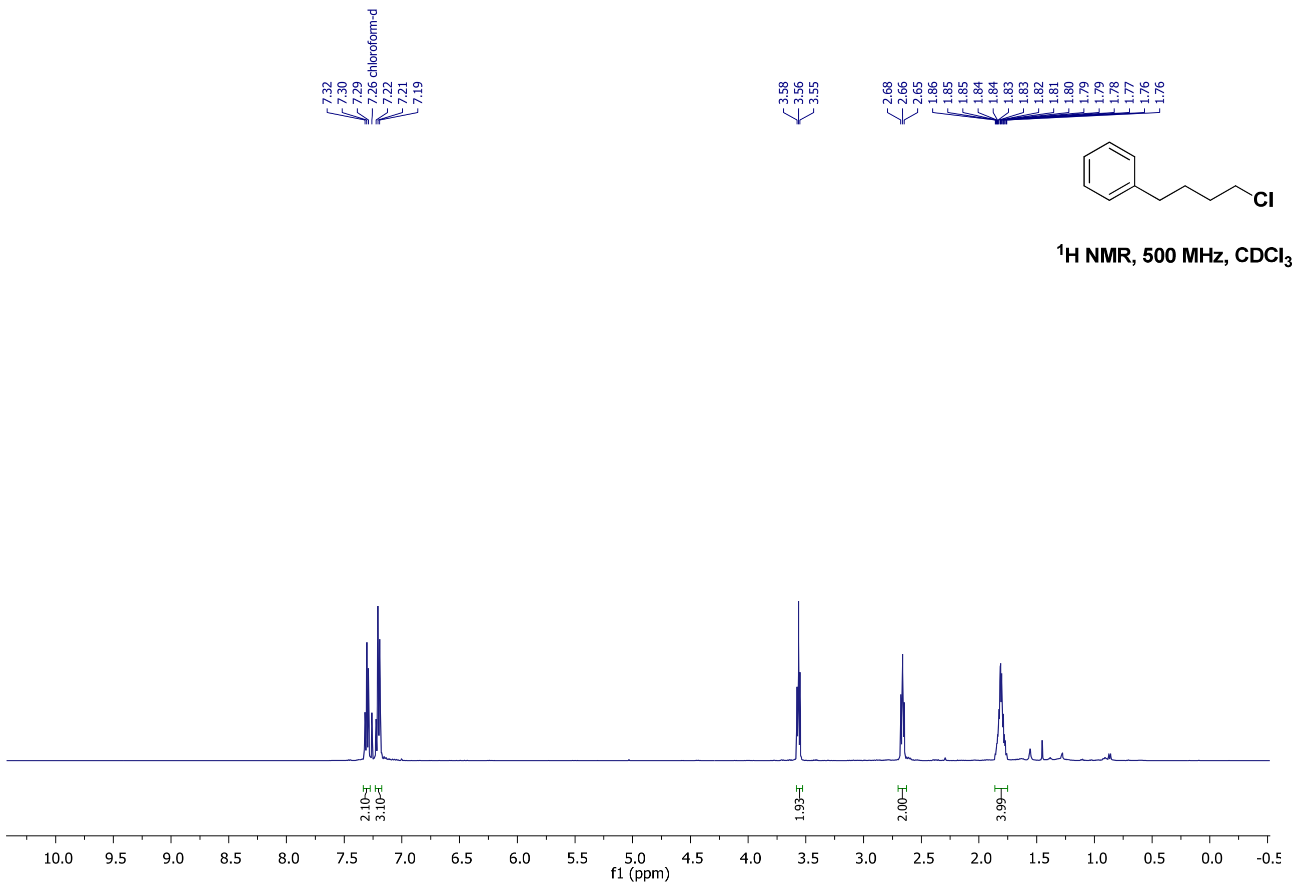

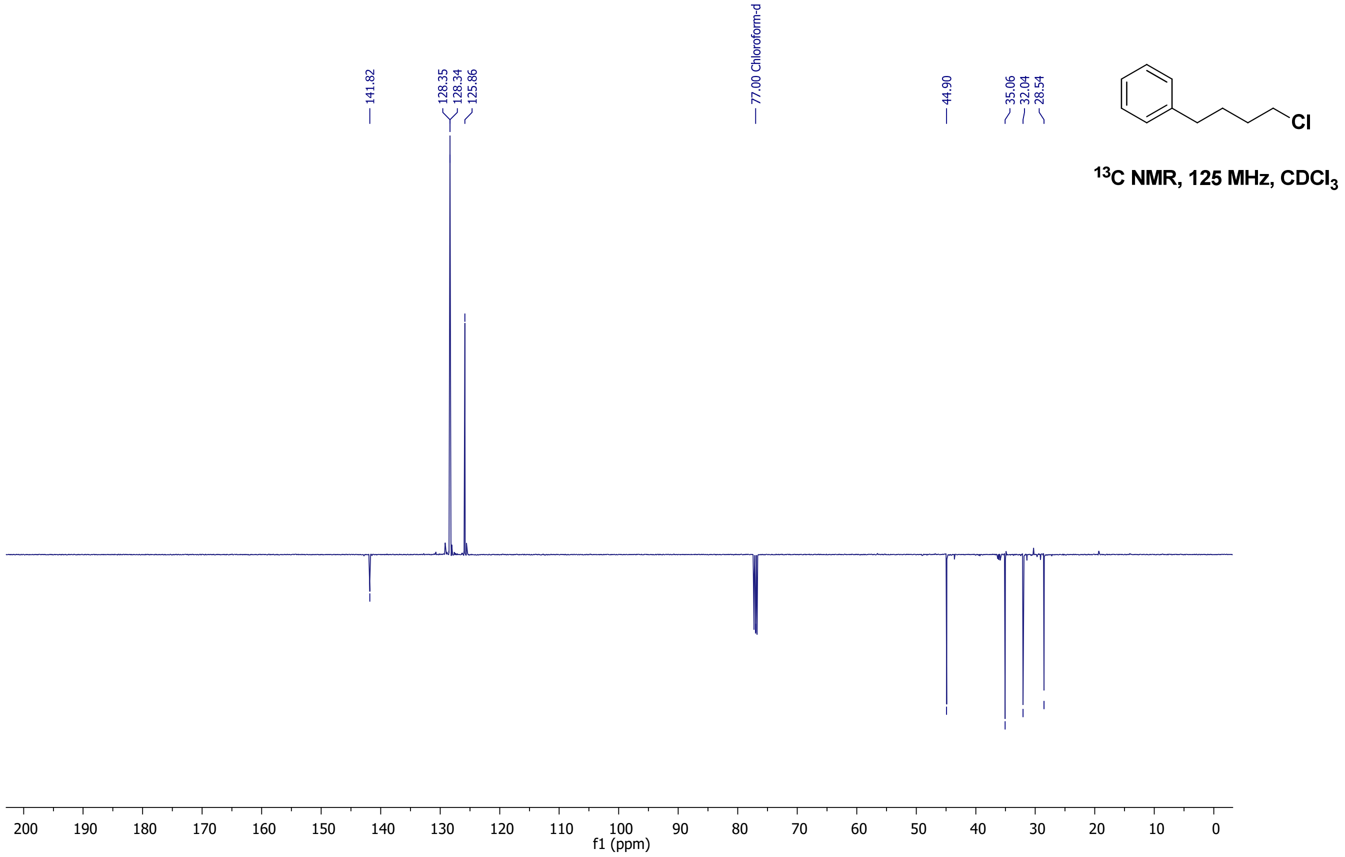

(4-Chloro-1-butyn-1-yl)benzene (19)

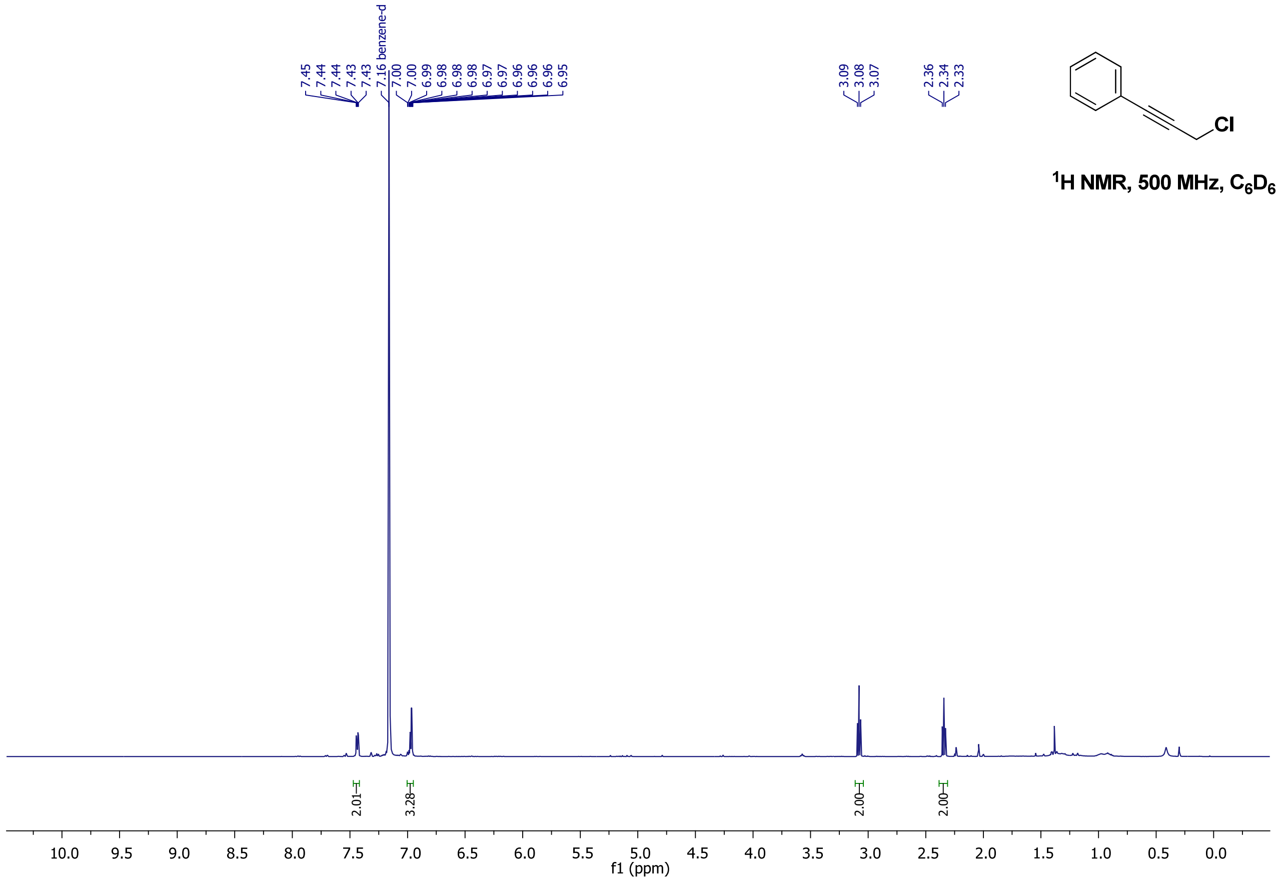

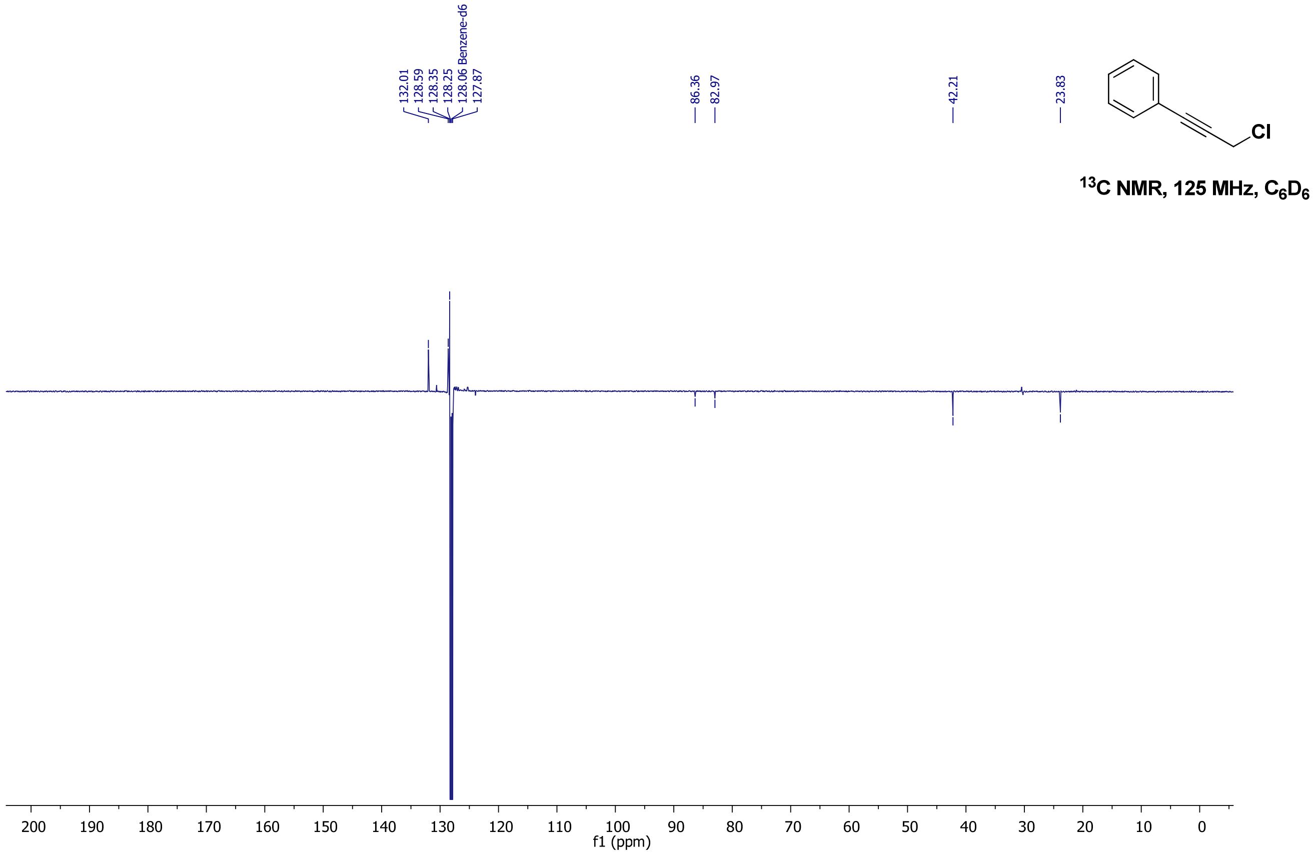

[4-(Chloromethyl)cyclohexyl]benzene (20)

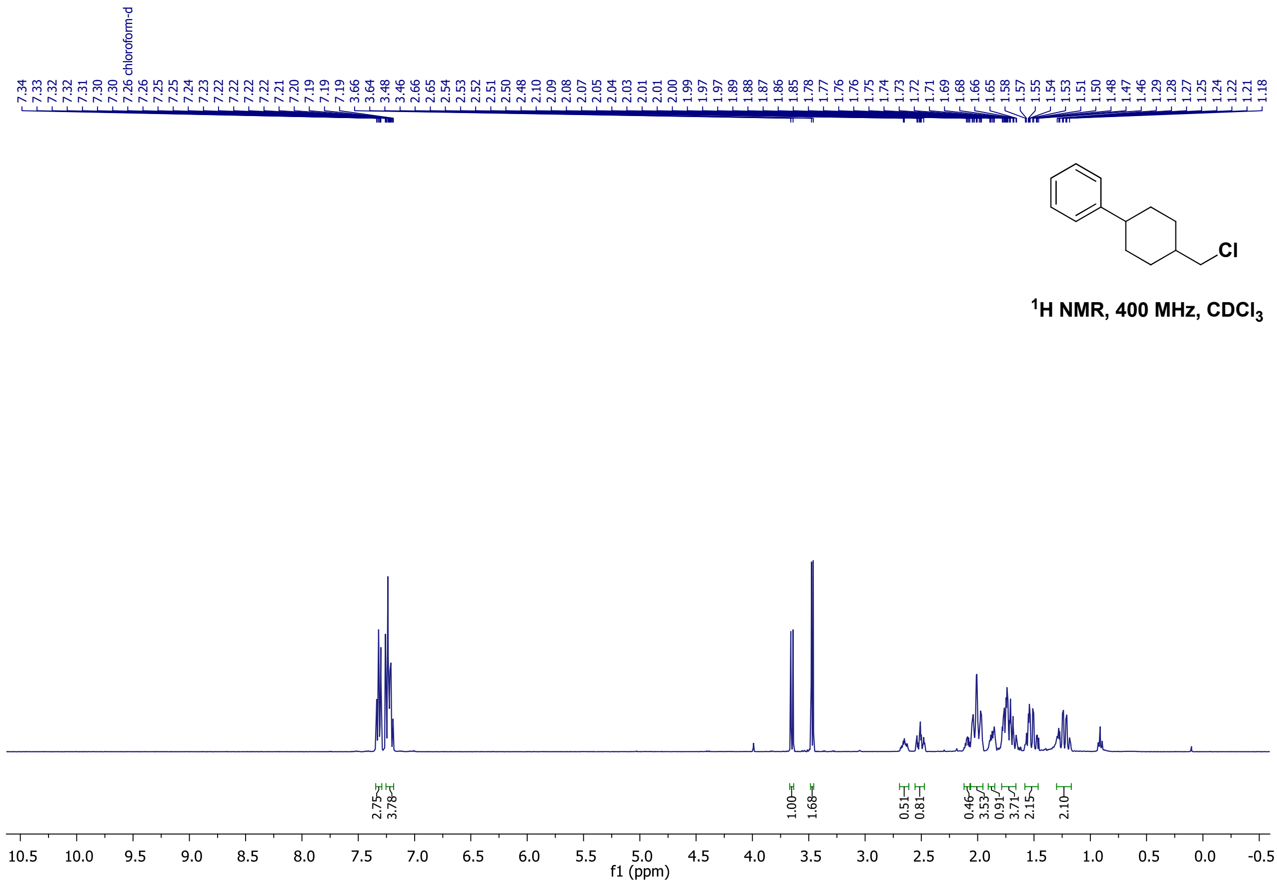

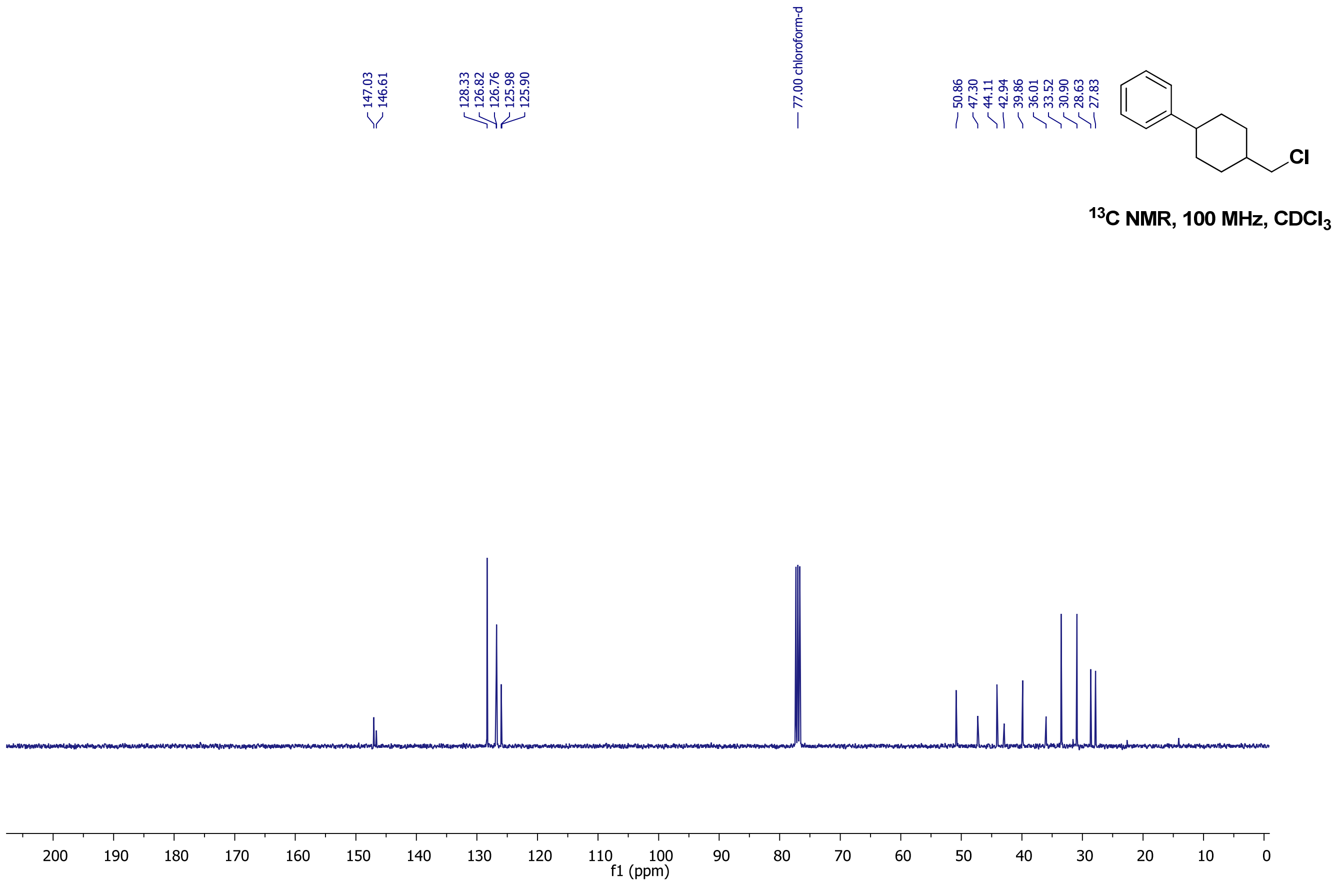

5-(Chloromethyl)nonane (21)

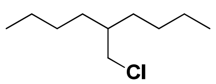

^{1}H NMR, 400 MHz, $CDCl_3$

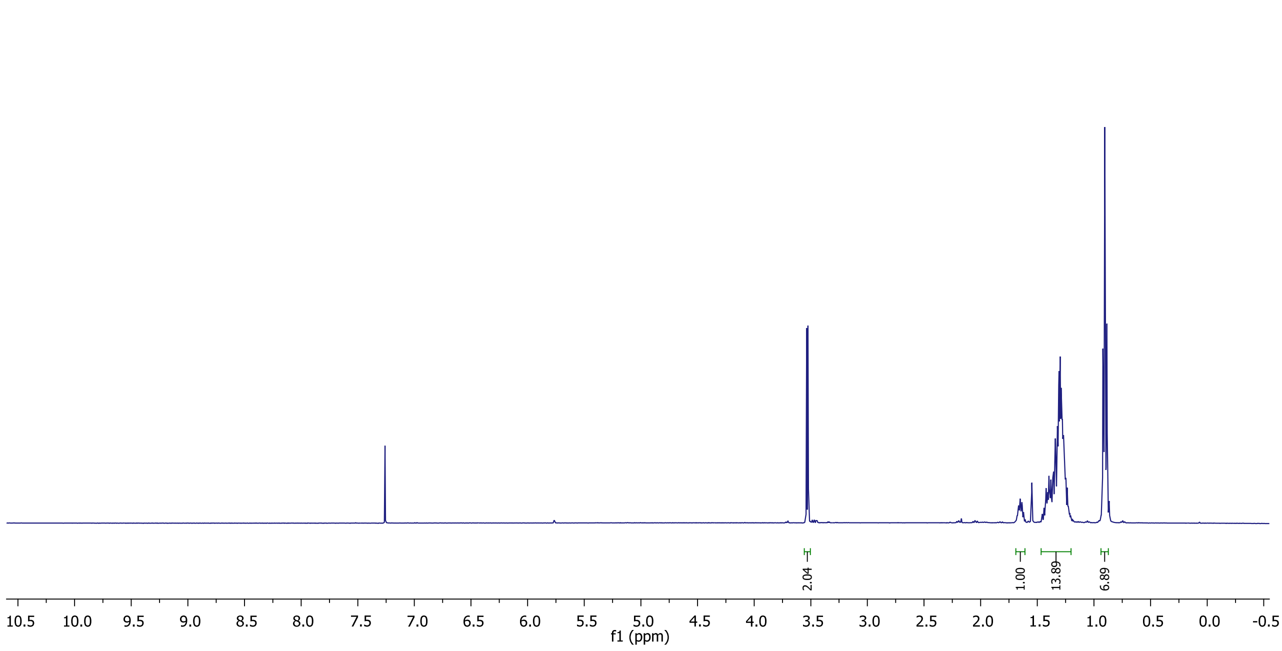

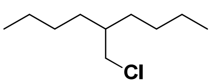

^{13}C NMR, 100 MHz, $CDCl_3$

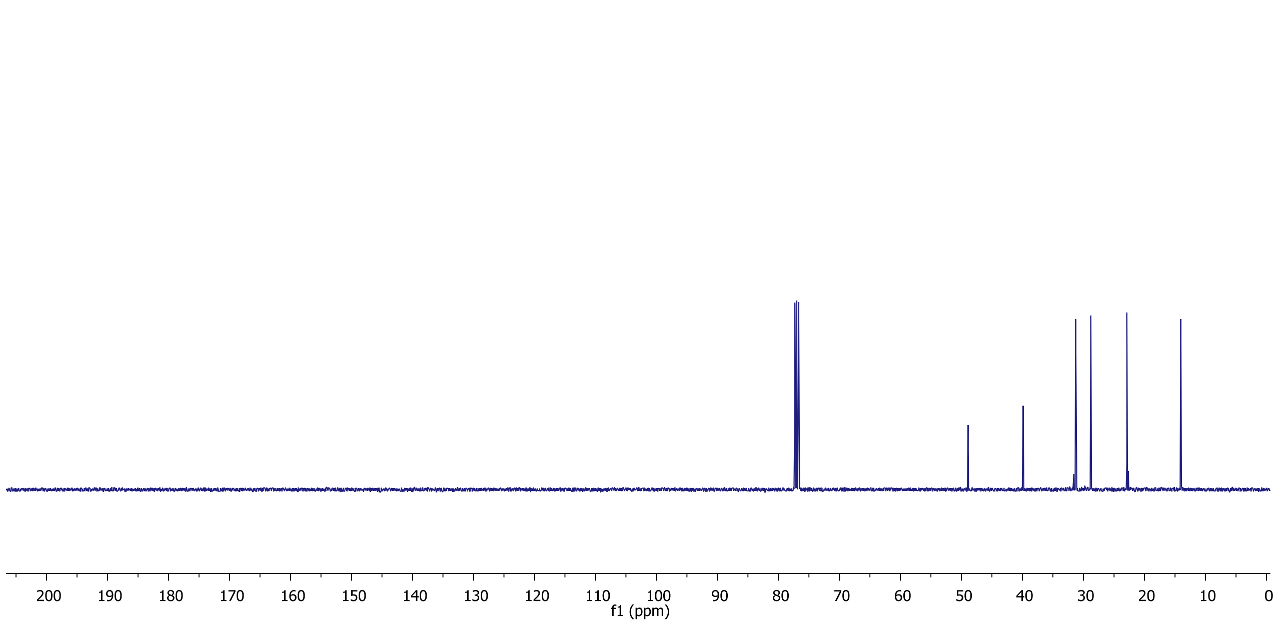

1-(Chloromethyl)indane (22)

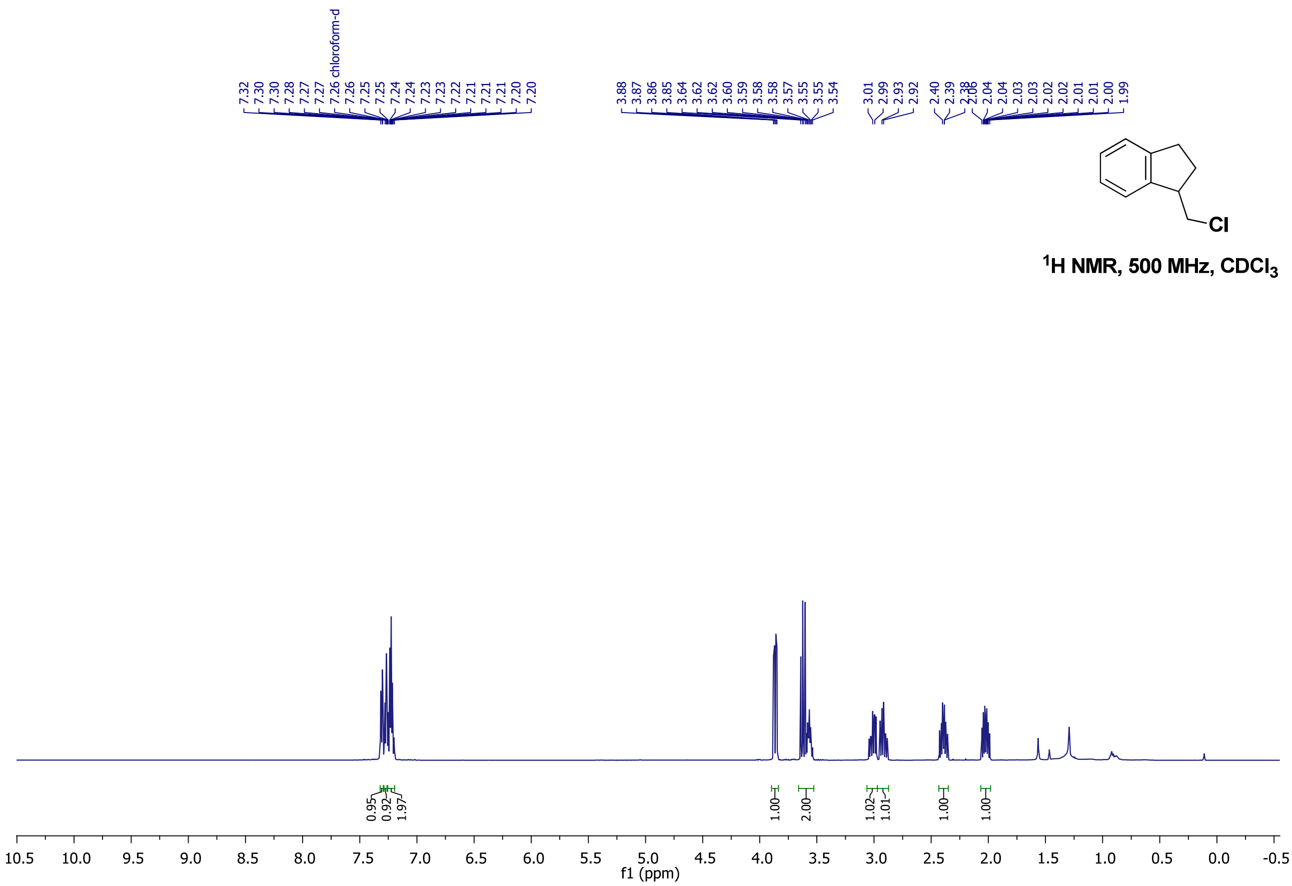

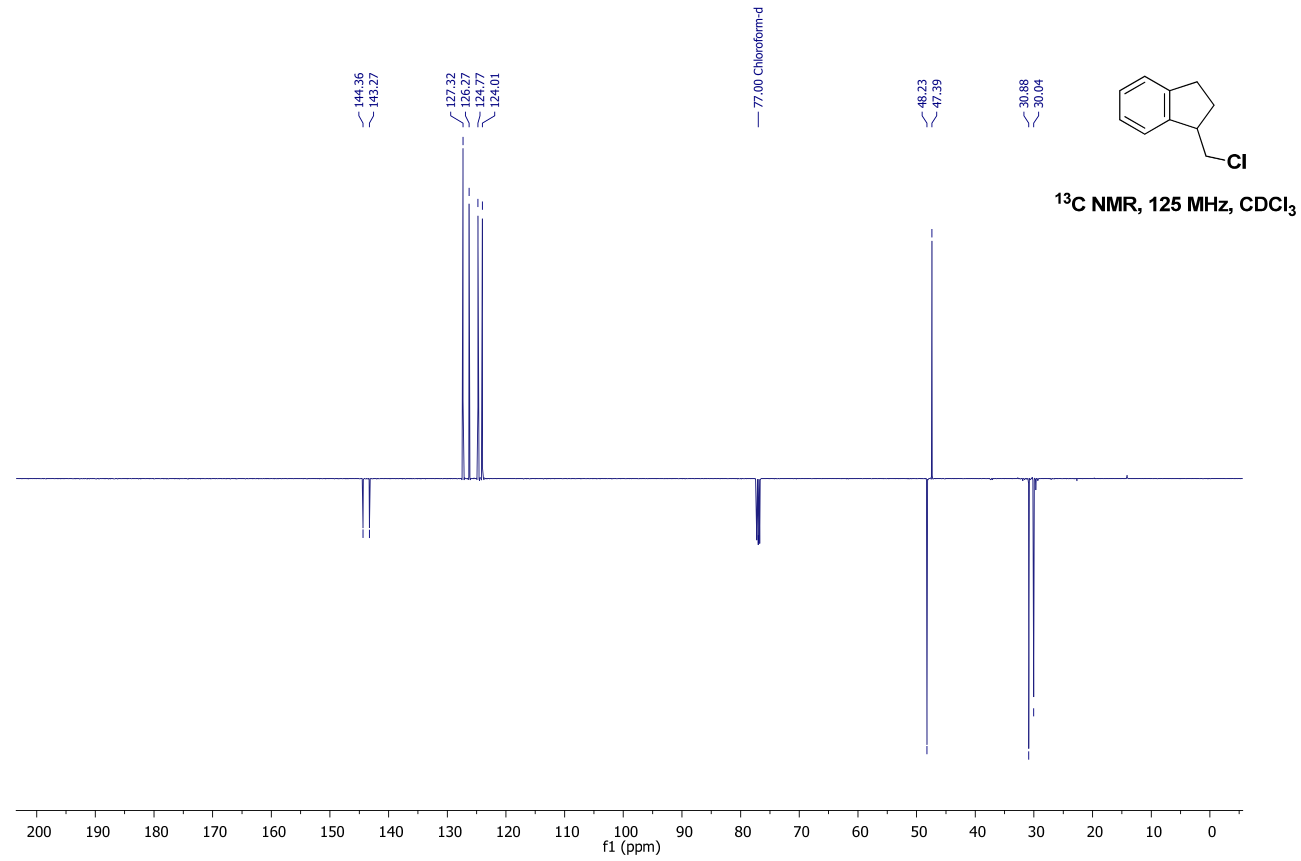

5-(Chloromethyl)-5,6,7,8-tetrahydro-2,3-naphthalenediol (23)

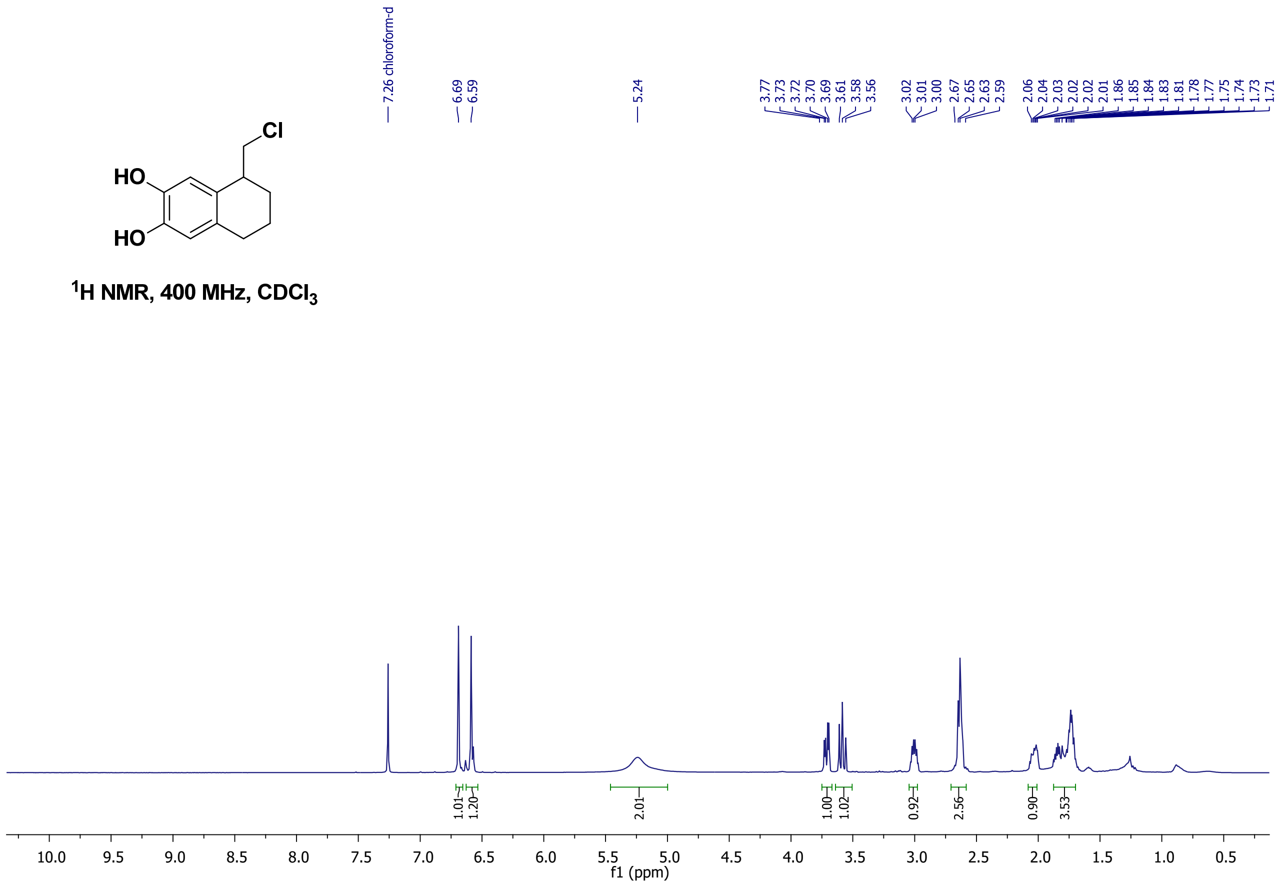

^{1}H NMR, 400 MHz, $CDCl_3$

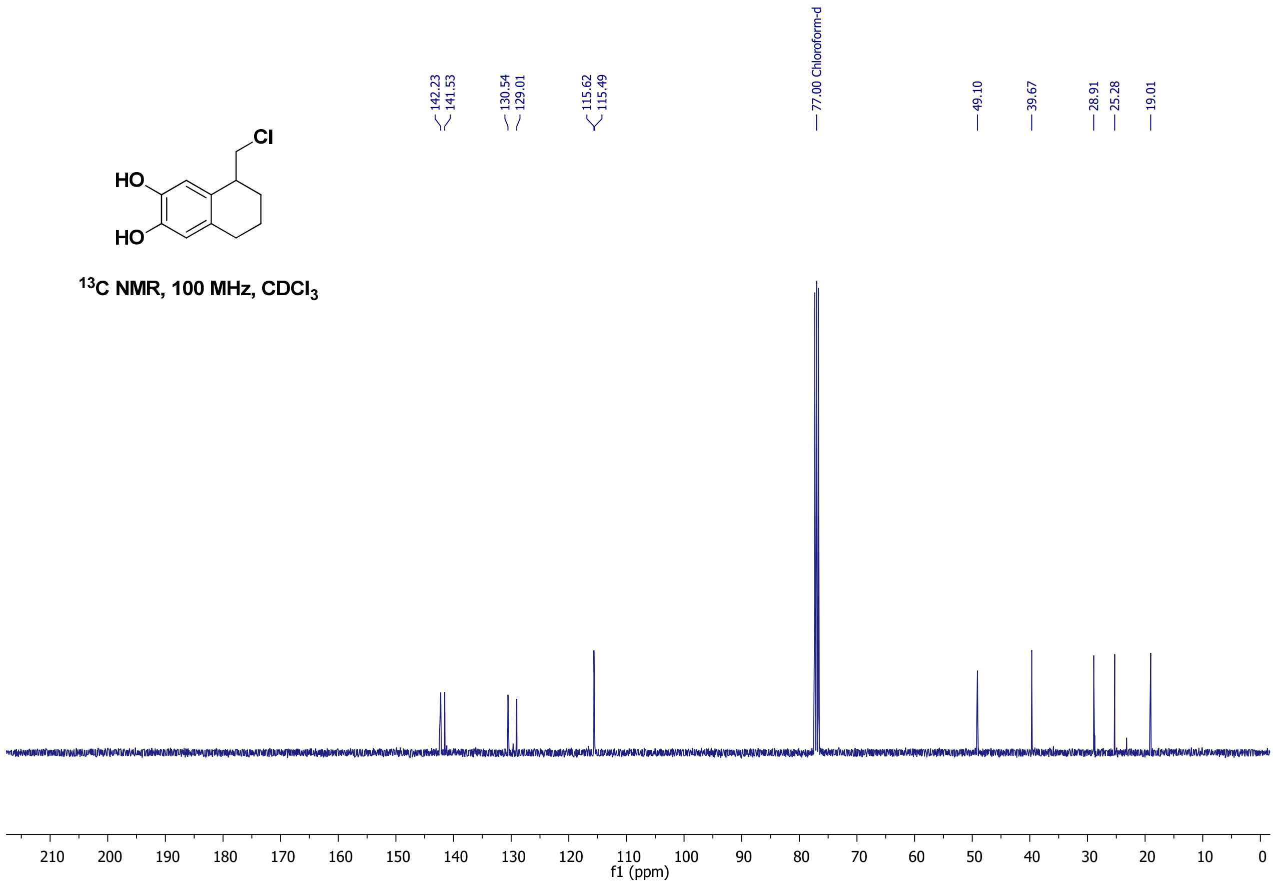

^{13}C NMR, 100 MHz, $CDCl_3$

1-(1-Chloro-2-propanyl)-4-iodobenzene (24)

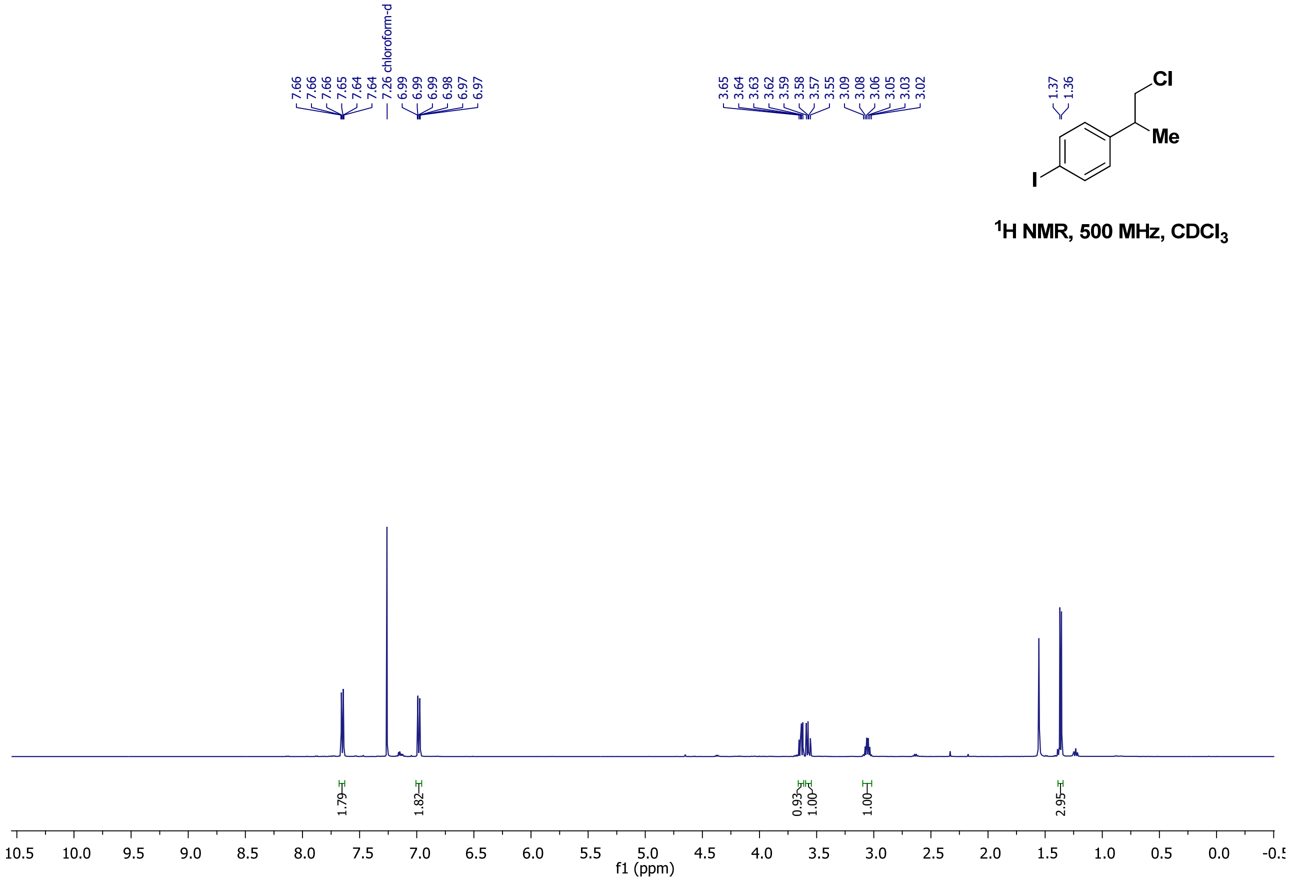

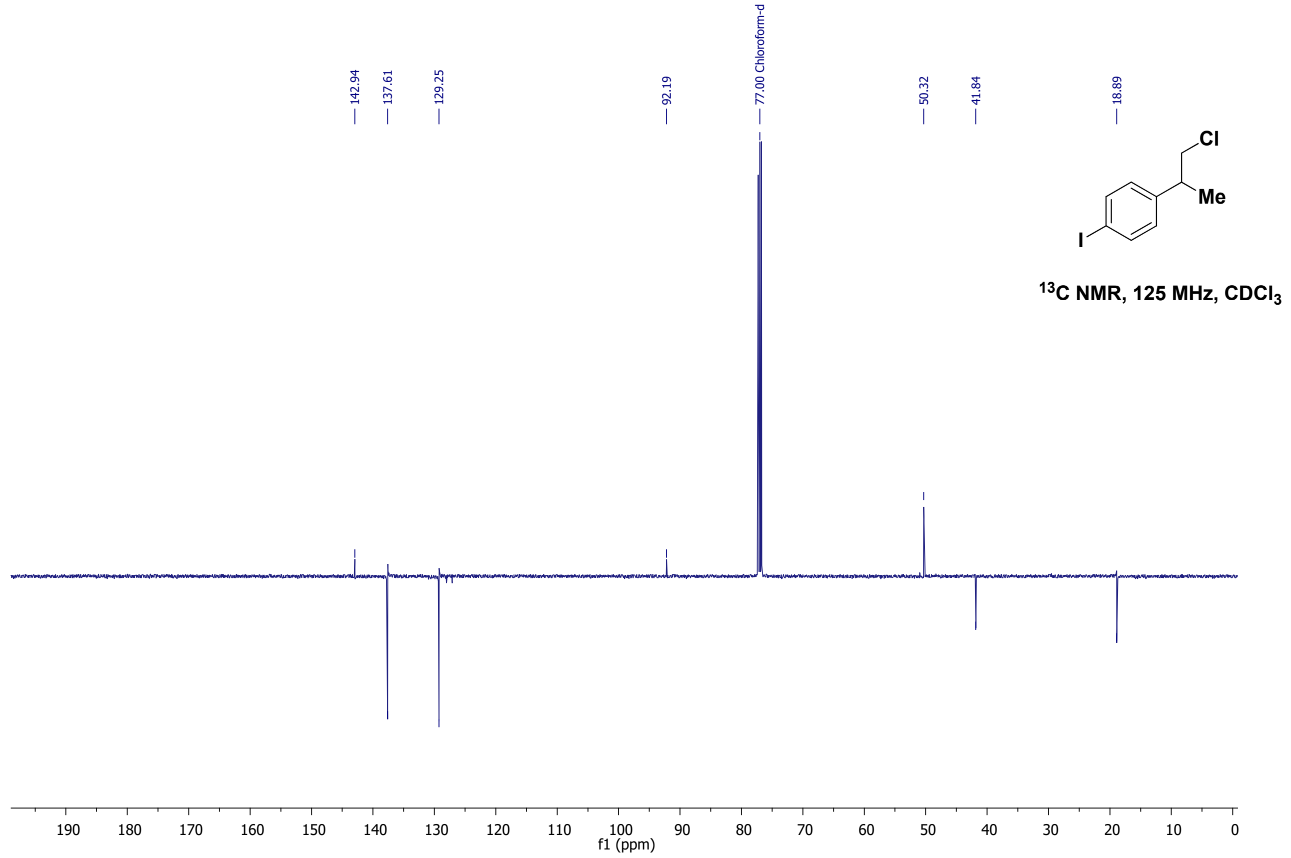

1-Bromo-4-(1-chloro-2-propanyl)benzene (25)

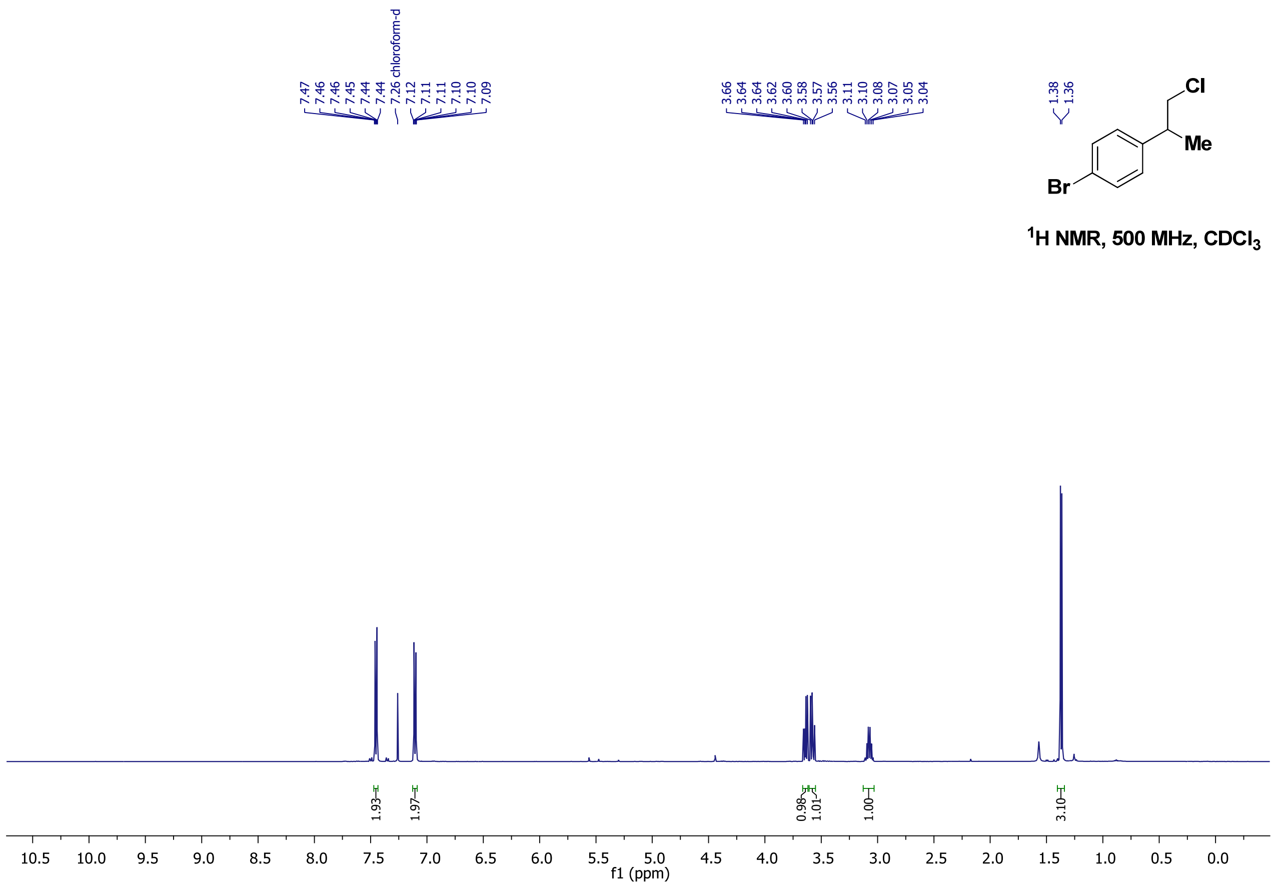

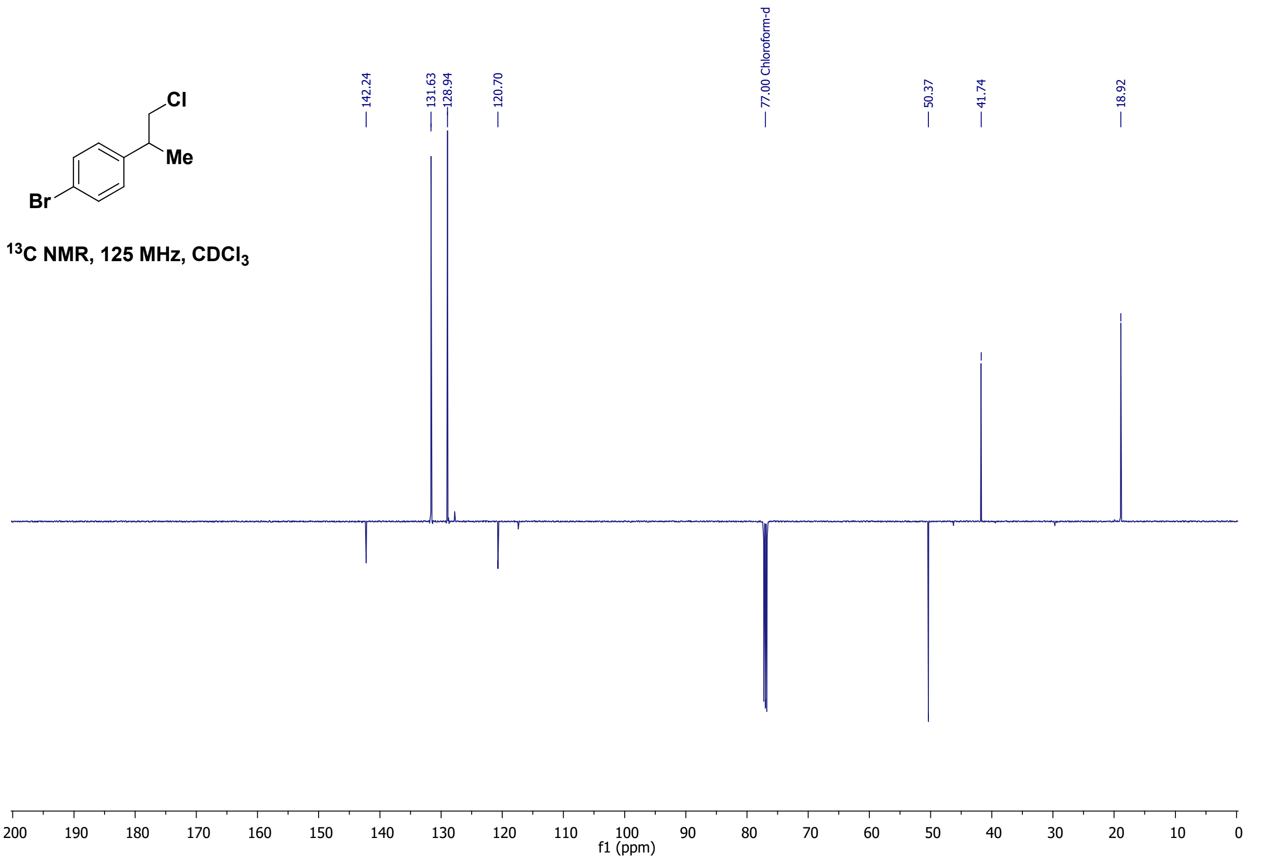

1-Chloro-4-(1-chloro-2-propanyl)benzene (26)

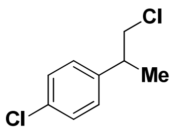

^{1}H NMR, 500 MHz, $CDCl_3$

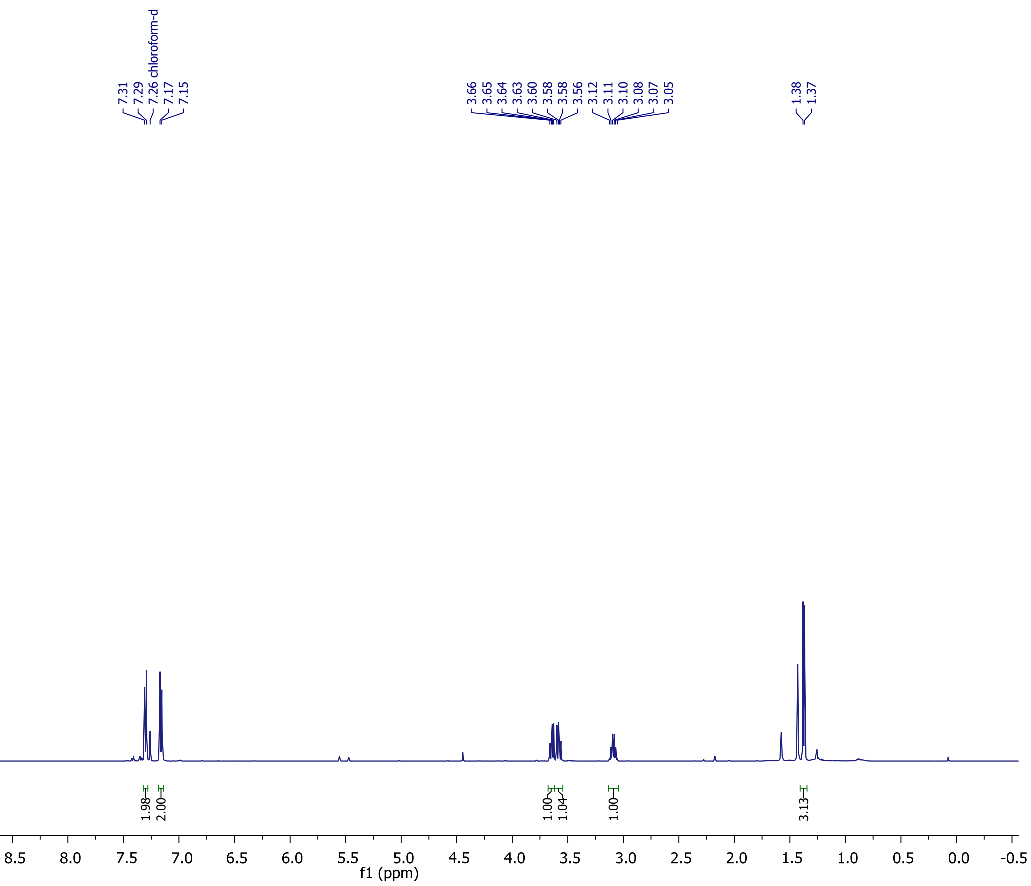

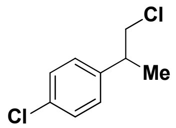

^{13}C NMR, 125 MHz, $CDCl_3$

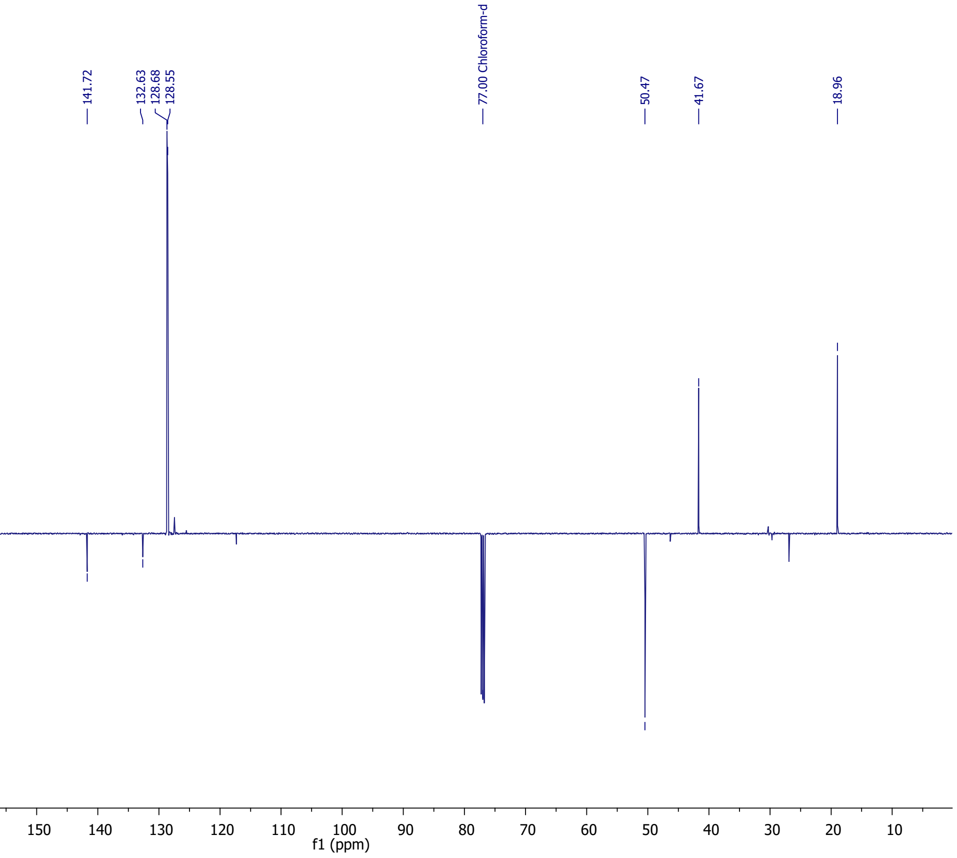

2,4-Dichloro-1-(1-chloro-2-propanyl)benzene (27)

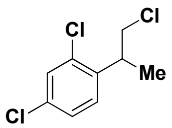

^{1}H NMR, 500 MHz, $CDCl_3$

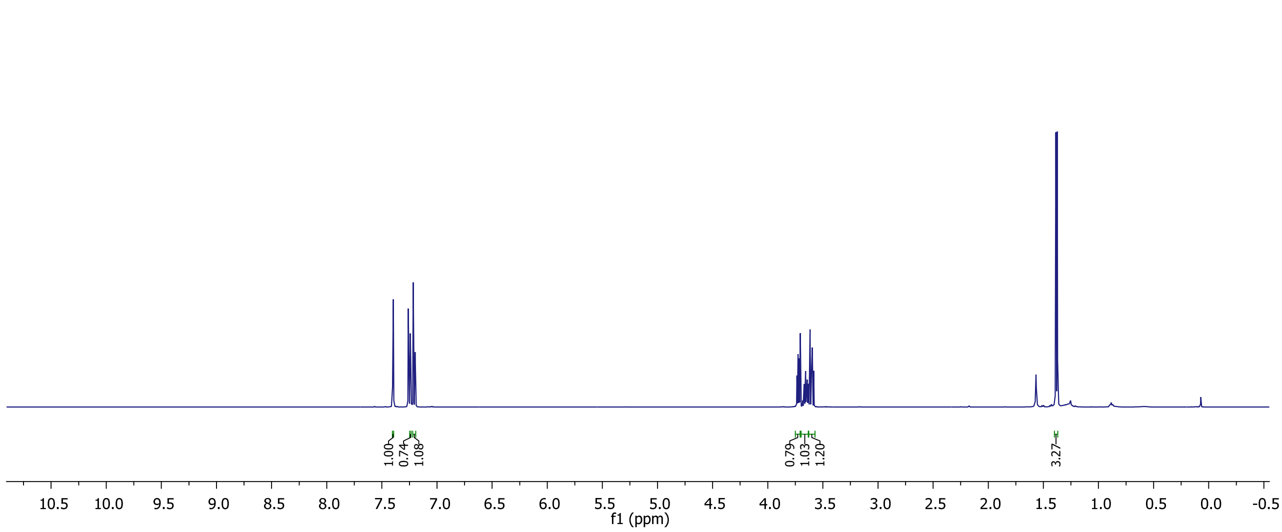

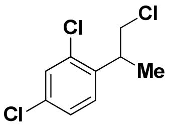

^{13}C NMR, 125 MHz, $CDCl_3$

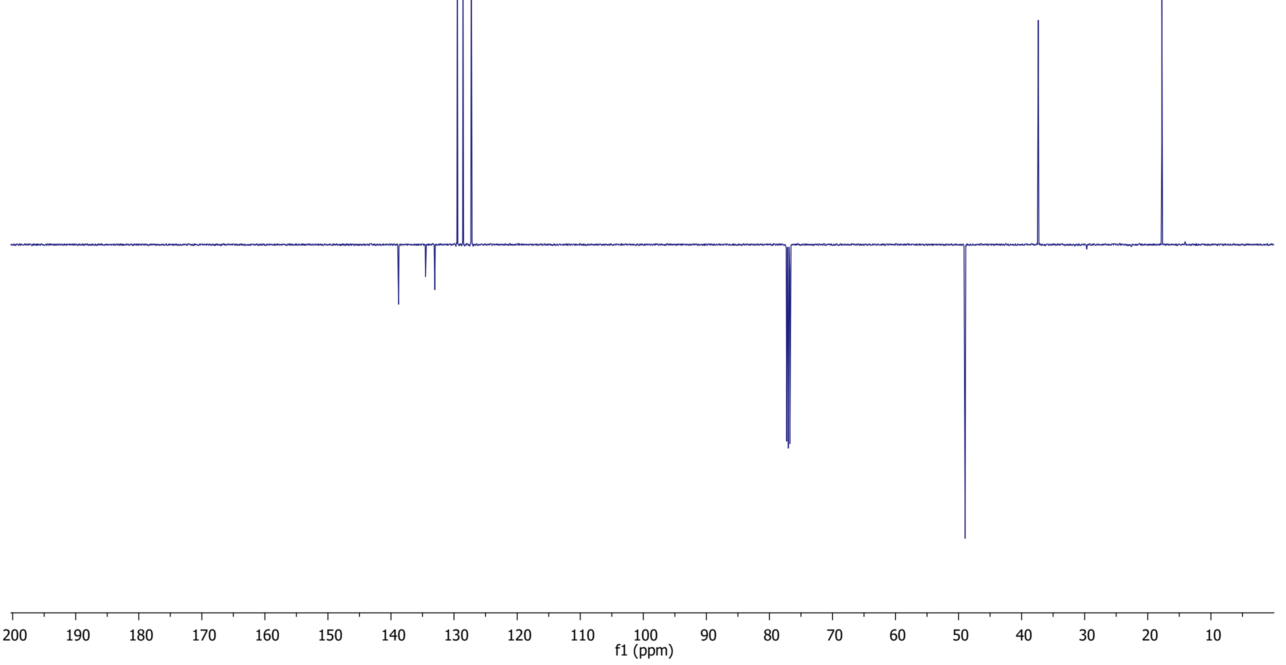

1-(1-Chloro-2propanyl)-4-fluorobenzene (28)

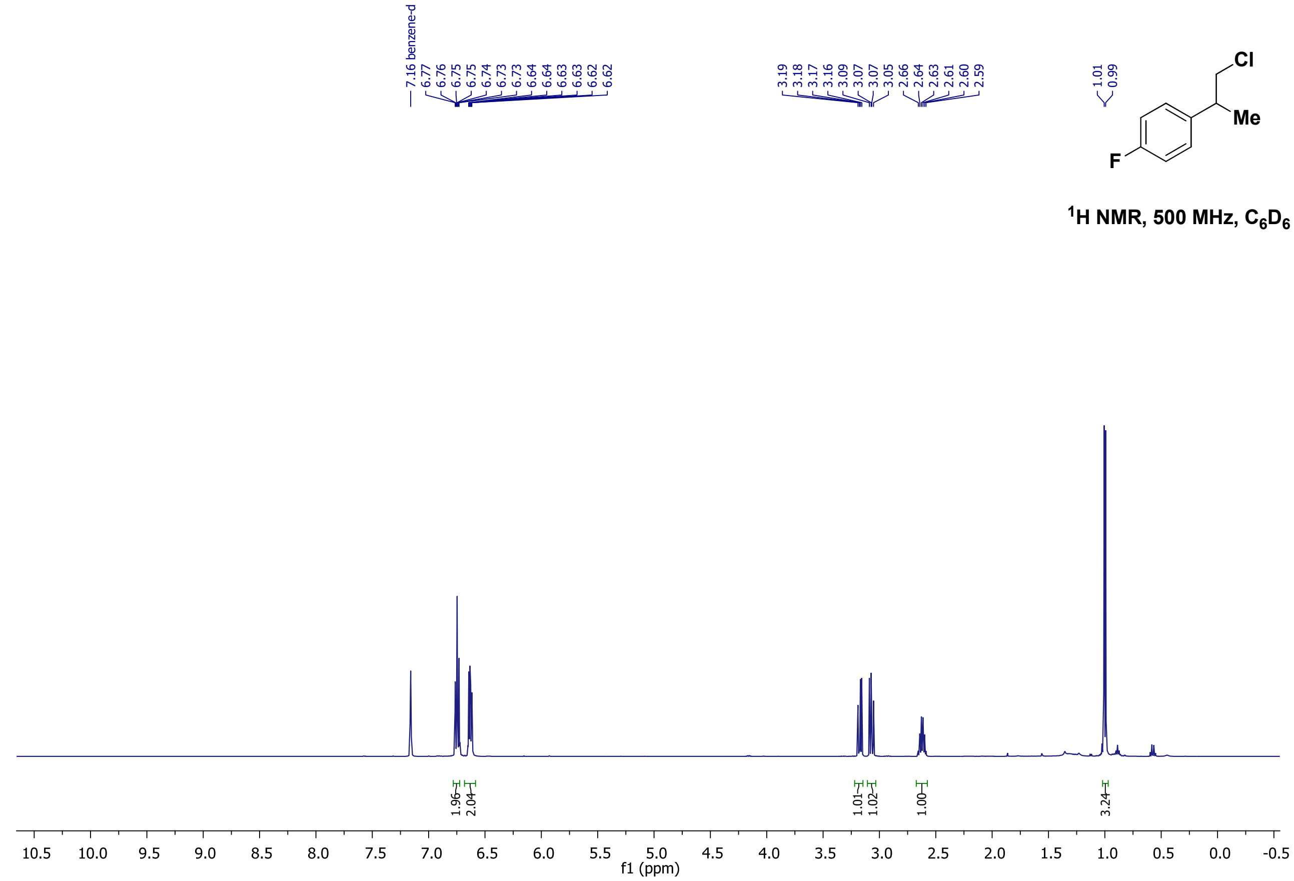

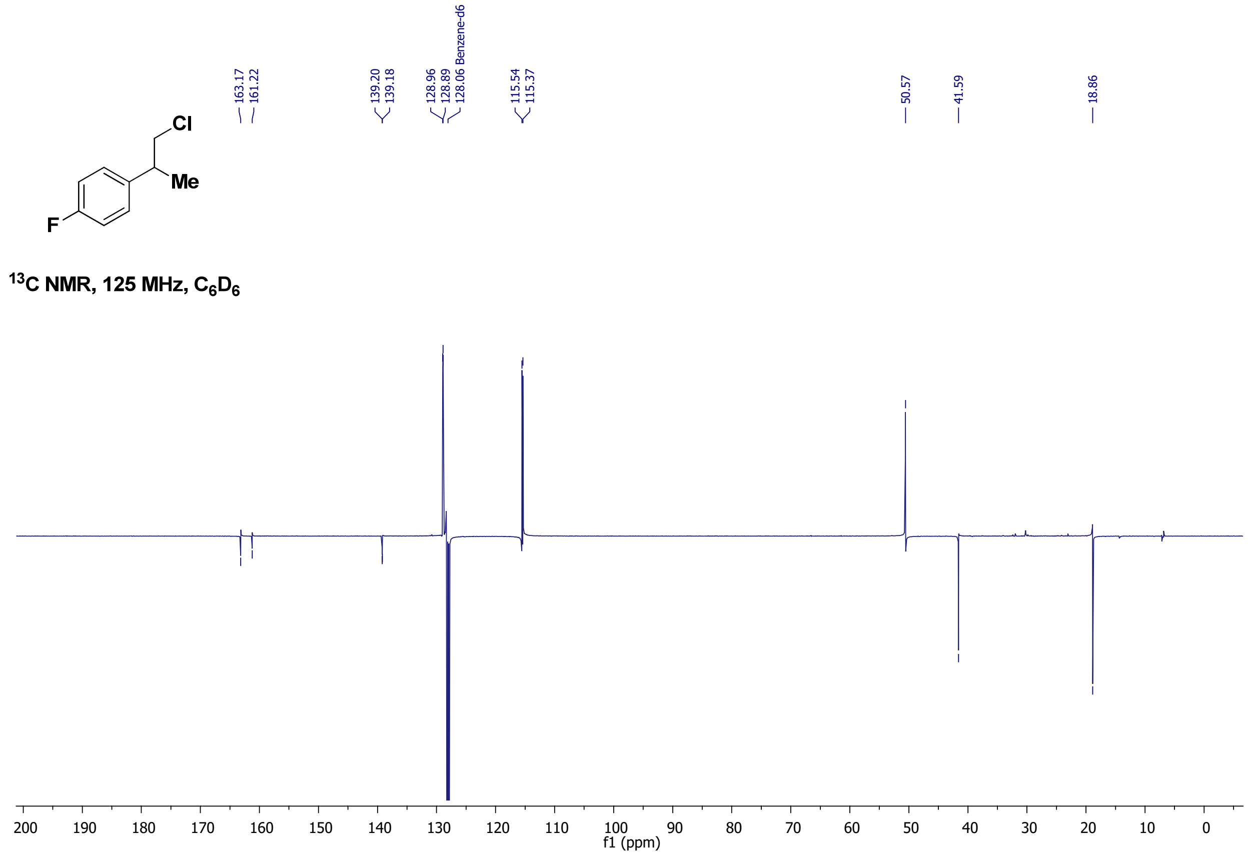

1-(1-Chloro-2-propanyl)-2,4-difluorobenzene (29)

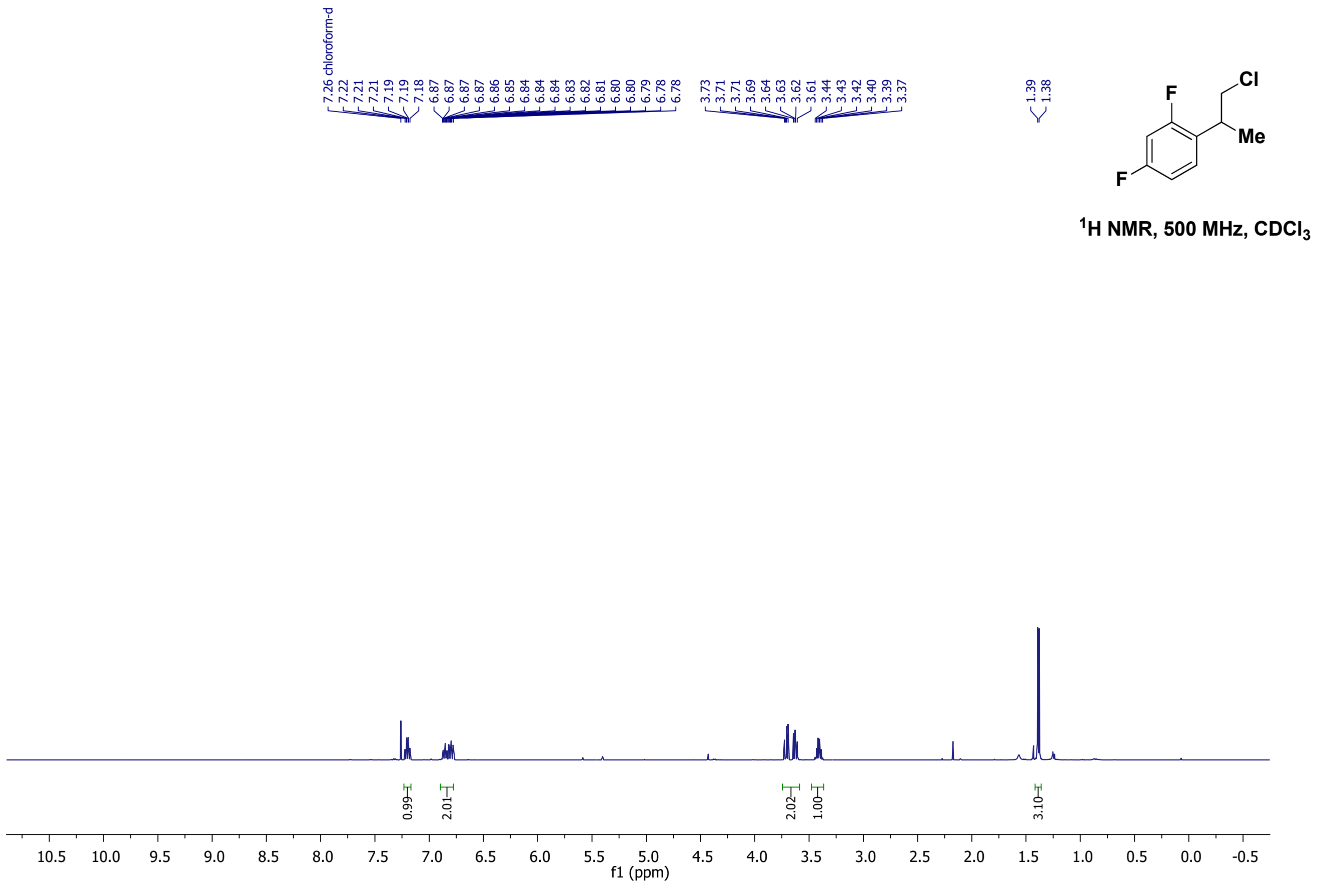

^{1}H NMR, 500 MHz, $CDCl_3$

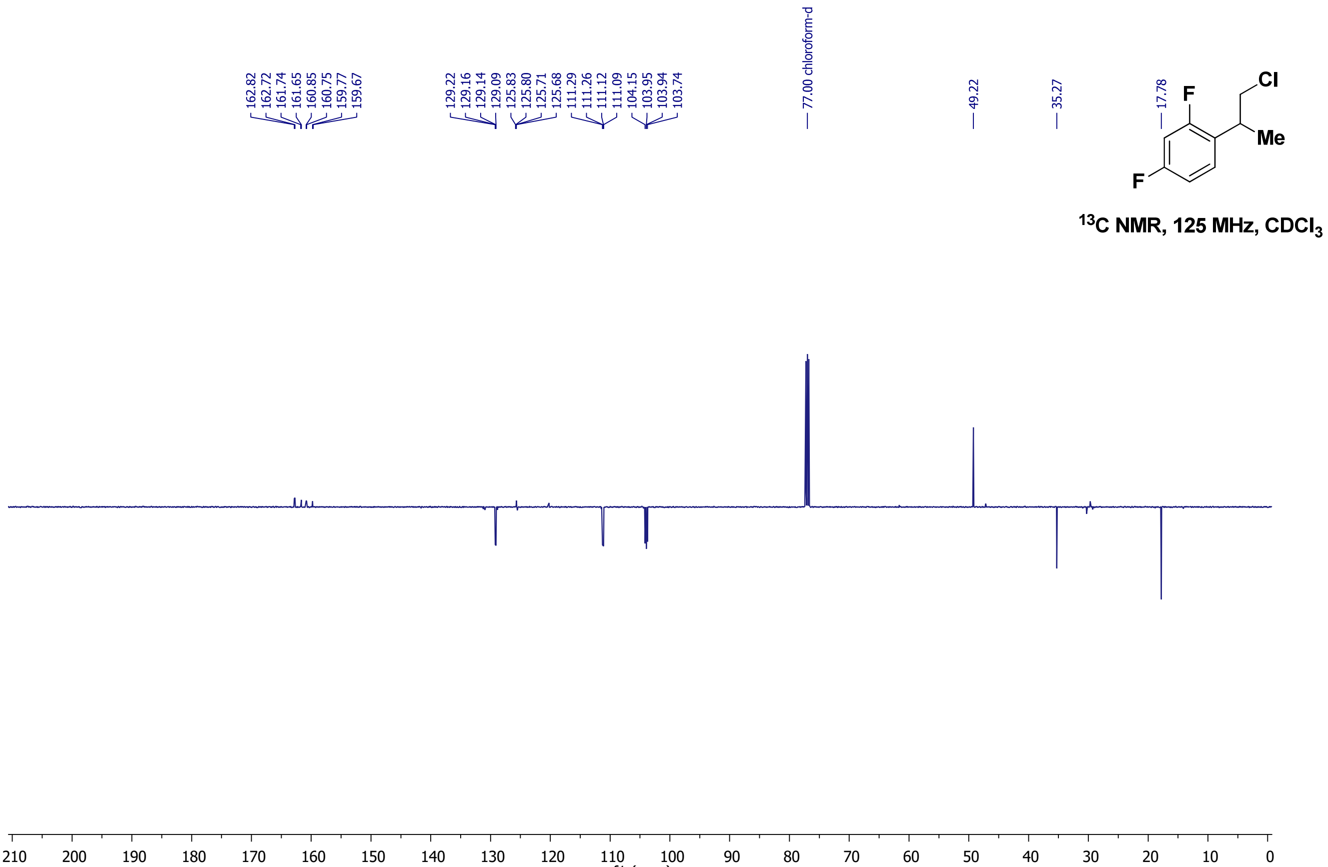

^{13}C NMR, 125 MHz, $CDCl_3$

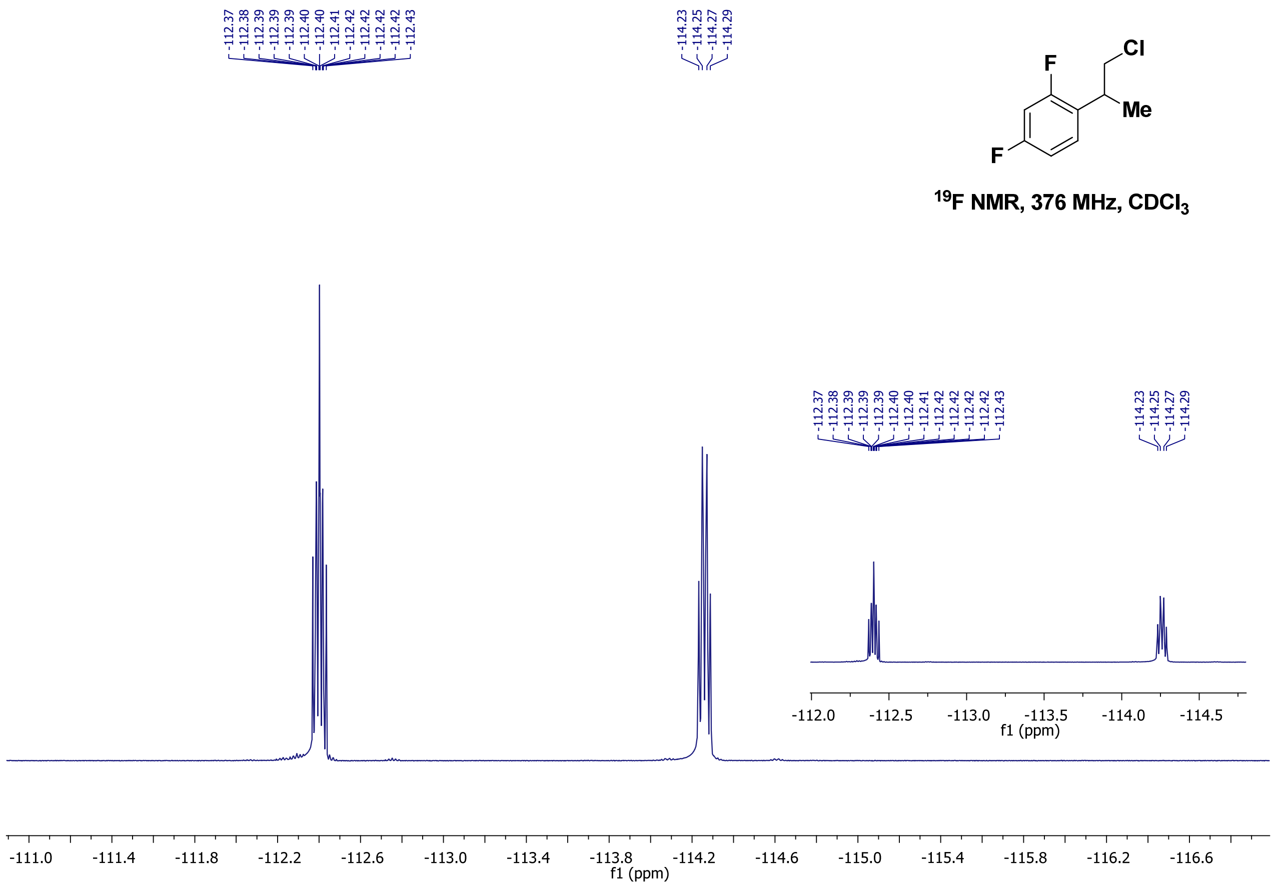

-112.37
-112.38
-112.39
-112.39
-112.39
-112.40
-112.40
-112.41
-112.42
-112.42
-112.42
-112.42
-112.42
-112.43
-114.23
-114.25
-114.27
-114.29
F
Cl
Me
F
¹⁹F NMR, 376 MHz, CDCl₃
-112.0
-112.5
-113.0
-113.5
-114.0
-114.5
f1 (ppm)
-111.0
-111.4
-111.8
-112.2
-112.6
-113.0
-113.4
-113.8
-114.2
-114.6
-115.0
-115.4
-115.8
-116.2
-116.6
f1 (ppm)

1-(1-Chloro-2-propanyl)-4-(trifluoromethyl)benzene (30)

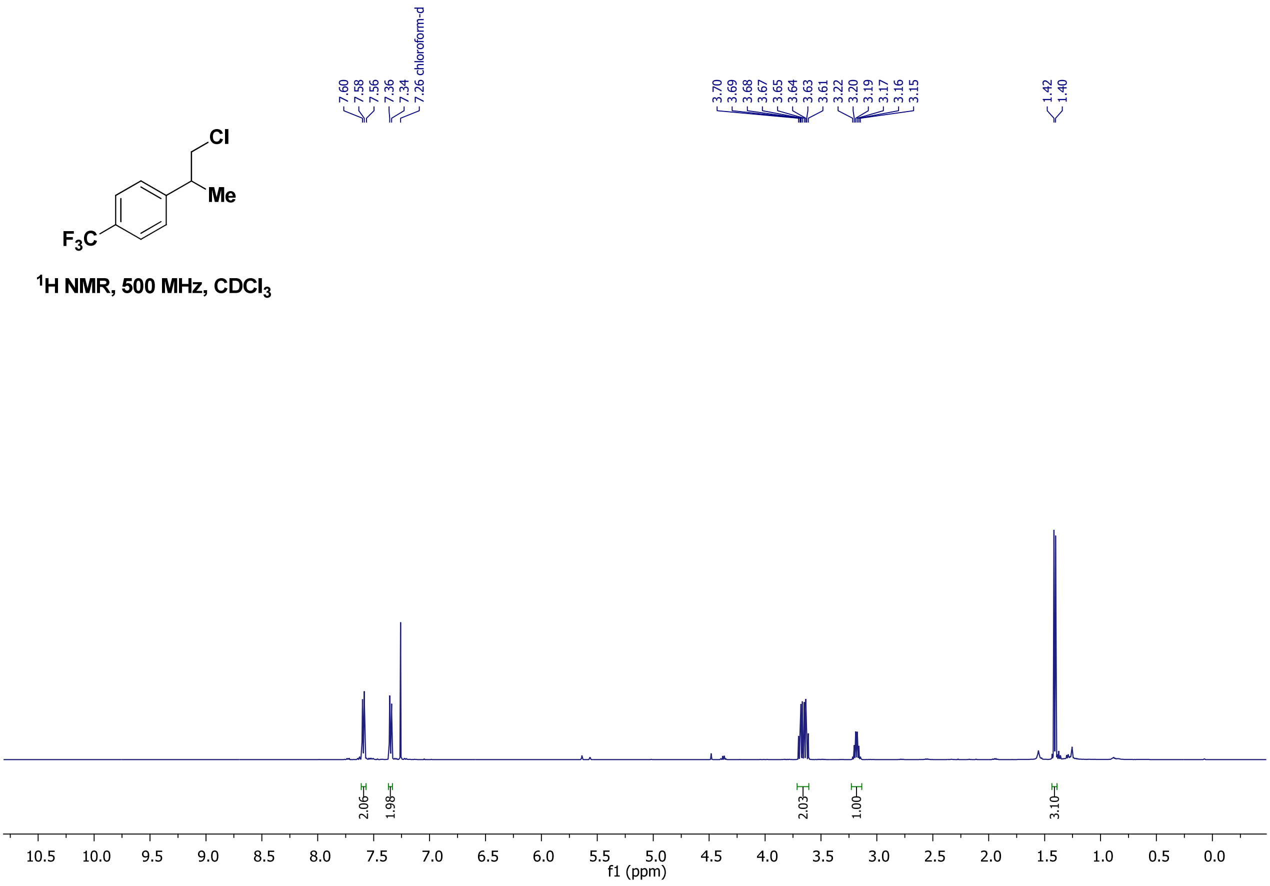

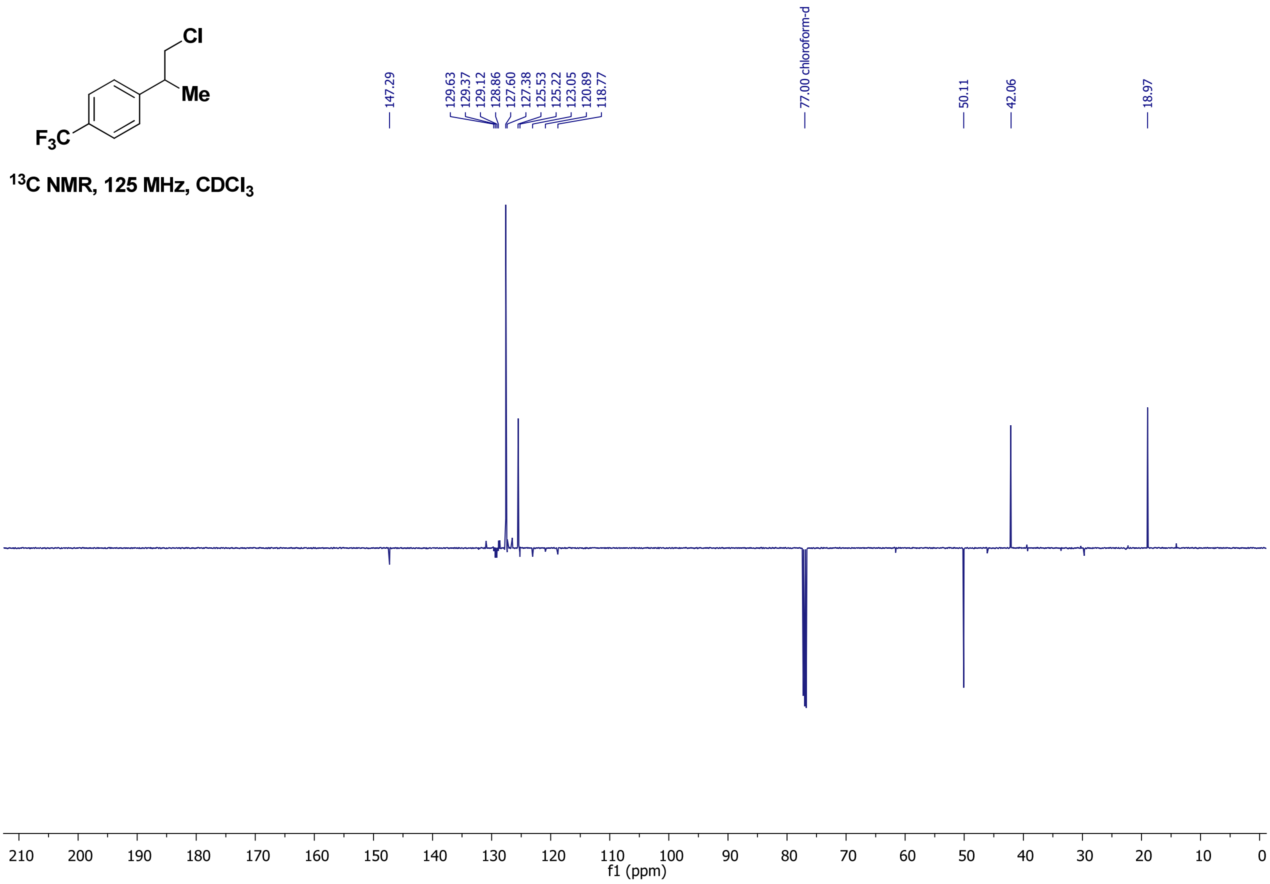

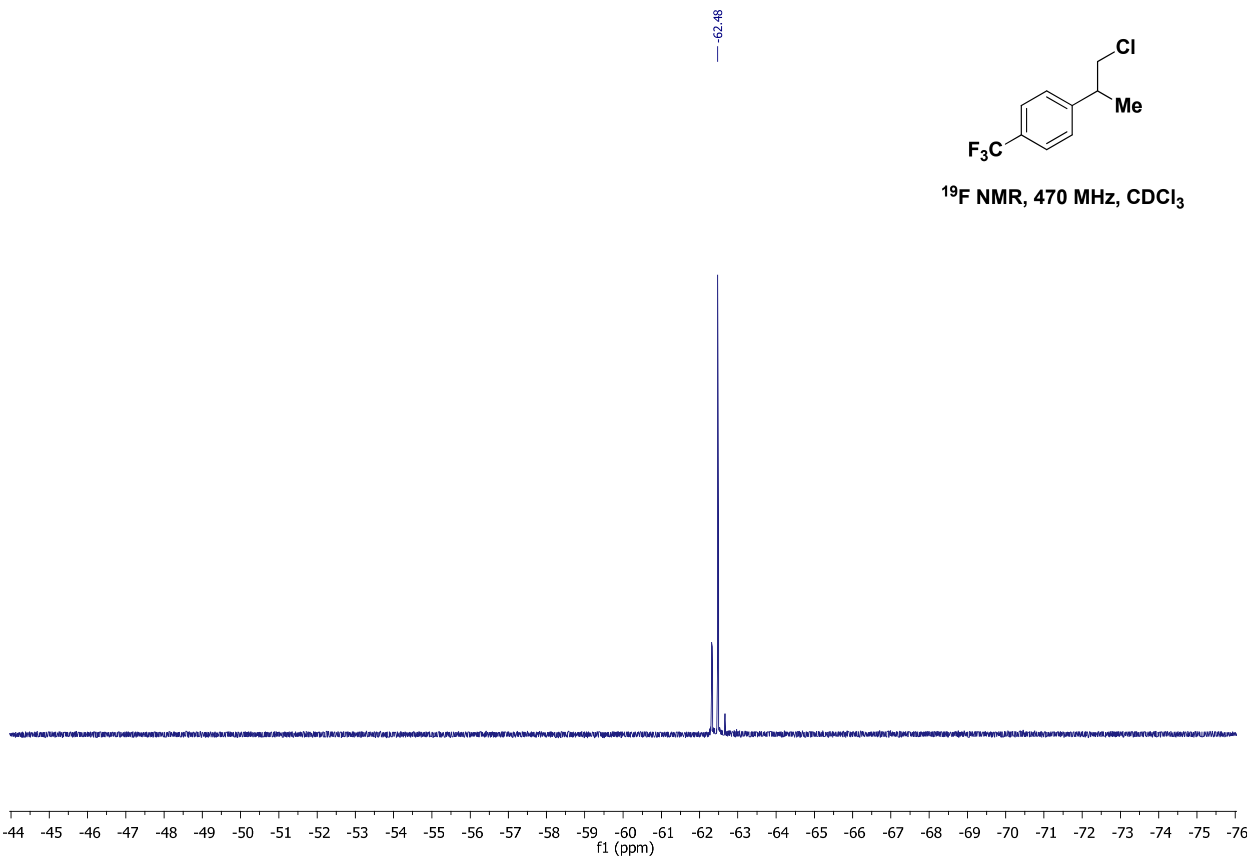
-62.48
Cl
Me
F3C
19F NMR, 470 MHz, CDCl3
-44 -45 -46 -47 -48 -49 -50 -51 -52 -53 -54 -55 -56 -57 -58 -59 -60 -61 -62 -63 -64 -65 -66 -67 -68 -69 -70 -71 -72 -73 -74 -75 -76
f1 (ppm)

1-(1-Chloro-2propanyl)-4-ethylbenzene (31)

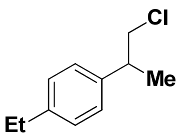

^{1}H NMR, 400 MHz, $CDCl_3$

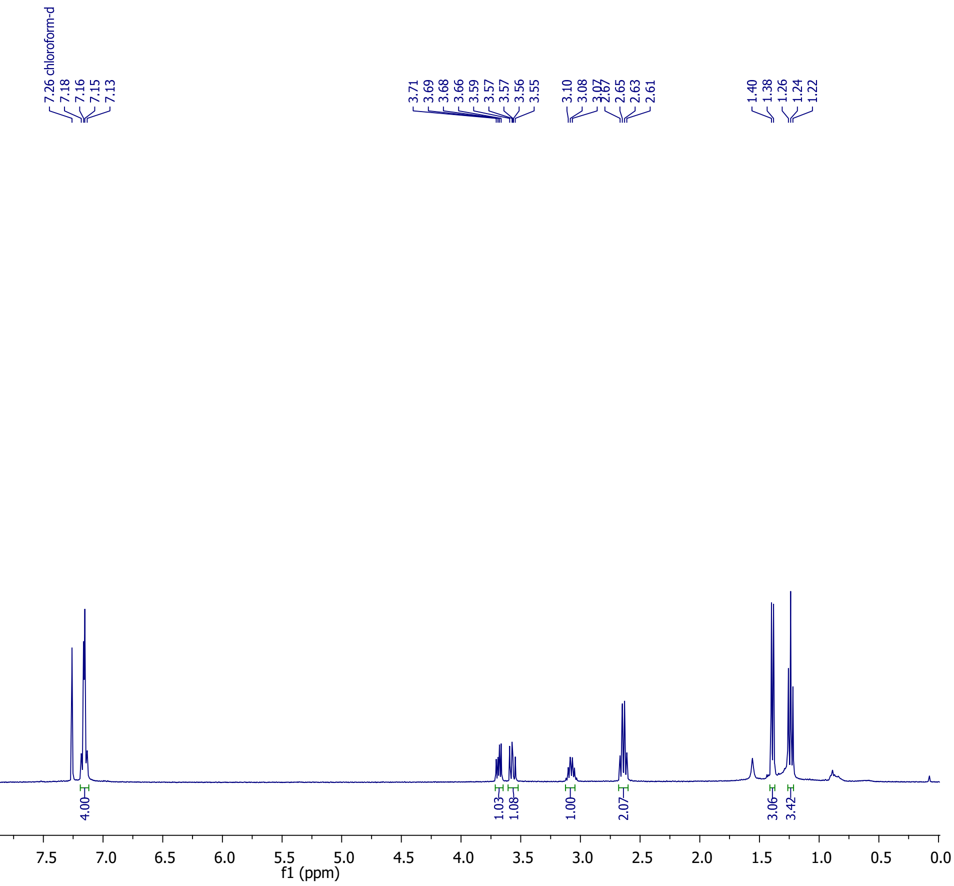

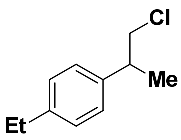

^{13}C NMR, 100 MHz, $CDCl_3$

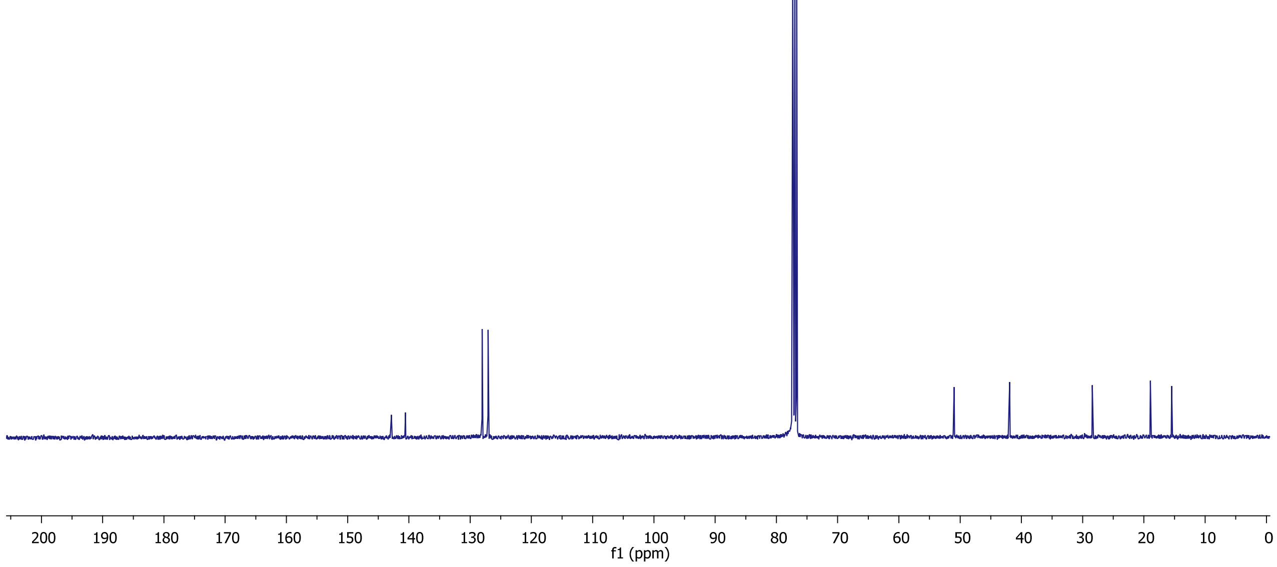

1-(1-Chloro-2-propanyl)-4-(2-methyl-2-propanyl)benzene (32)

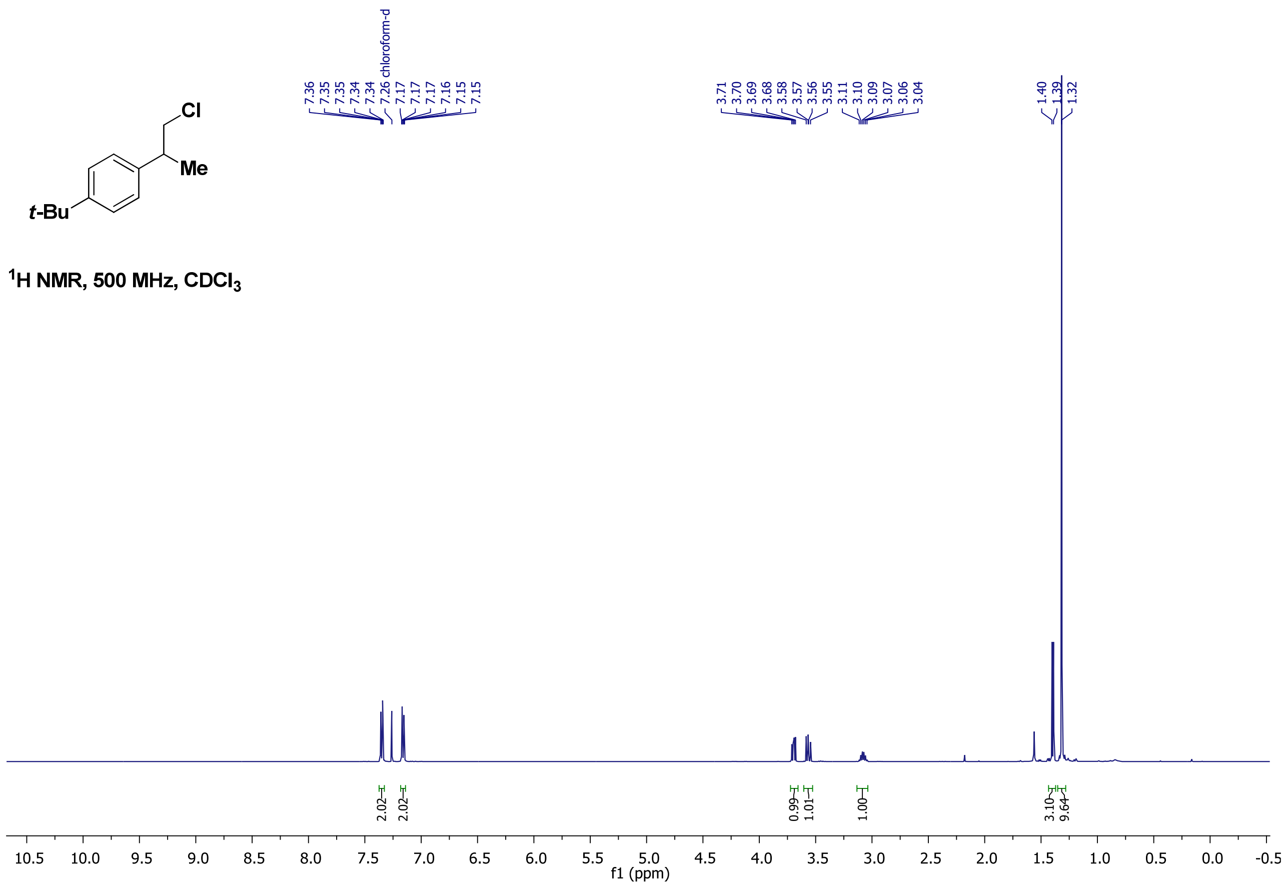

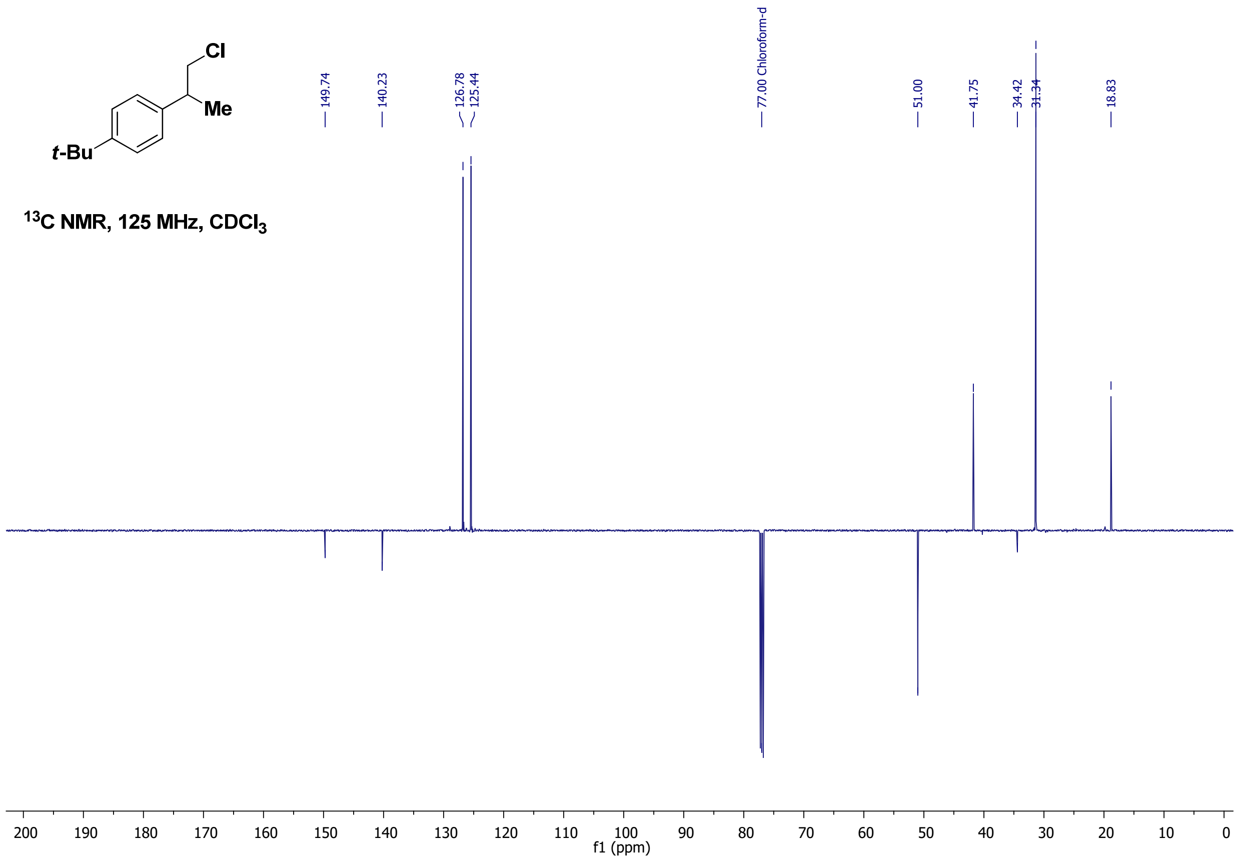

1-(1-Chloro-2-propanyl)-4-methoxybenzene (33)

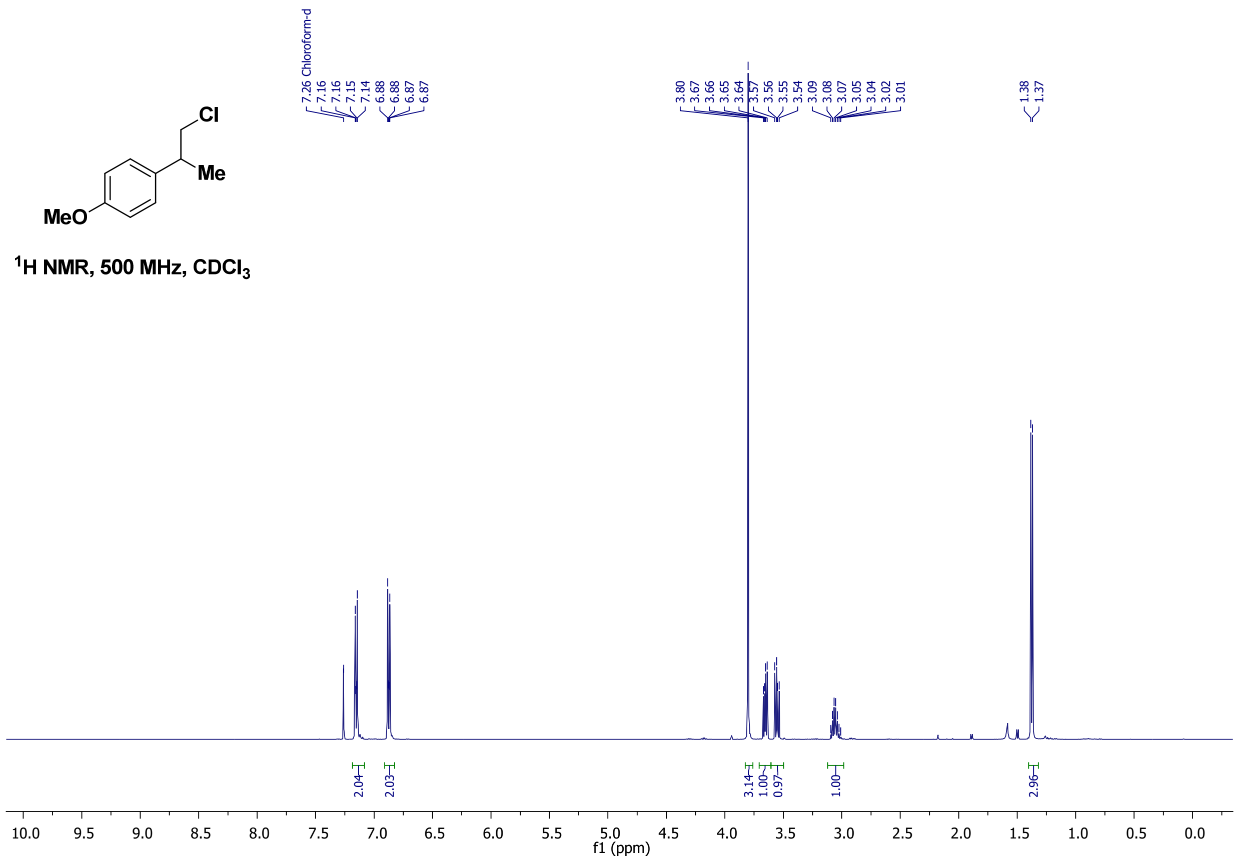

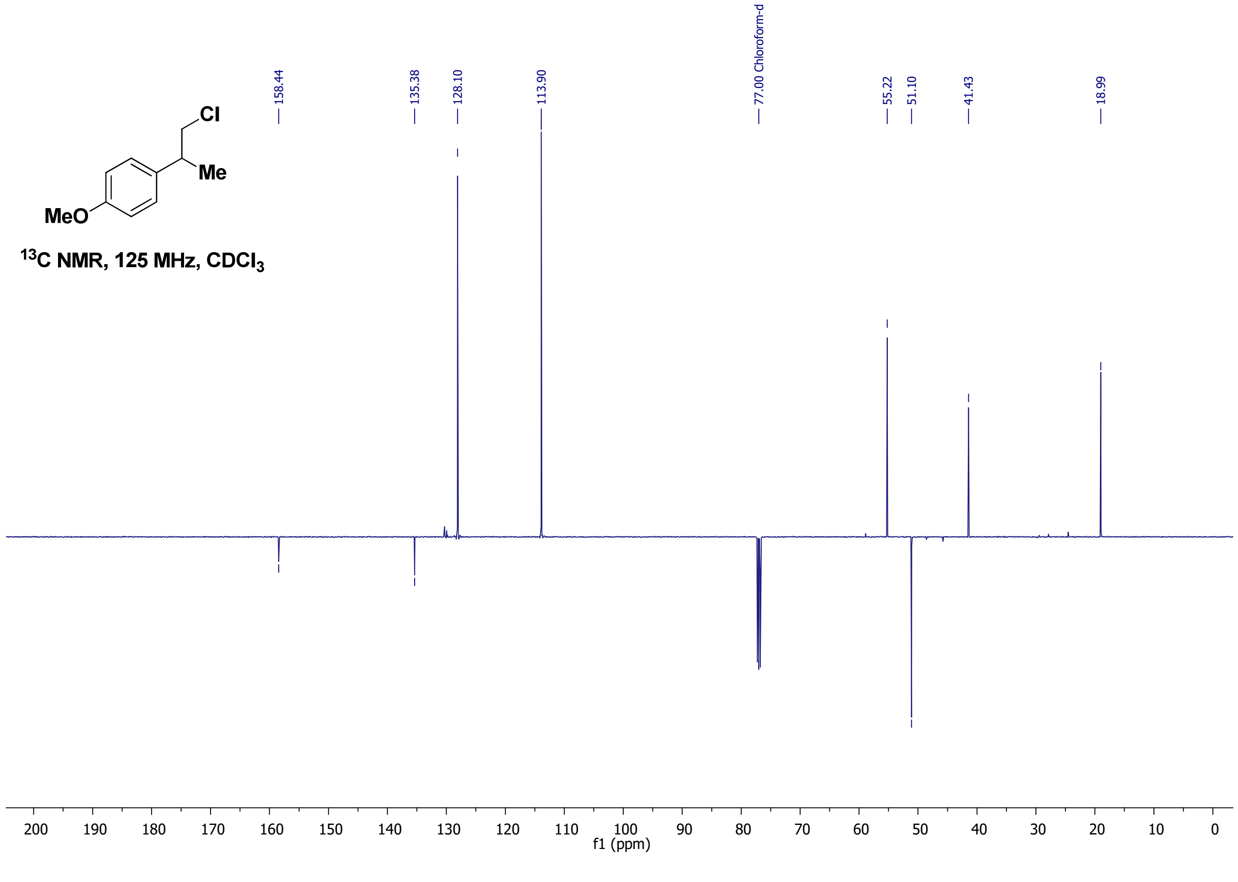

5-(1-Chloro-2-propanyl)-1,3-benzodioxole (34)

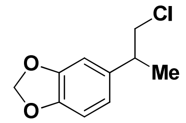

^{1}H NMR, 500 MHz, $CDCl_3$

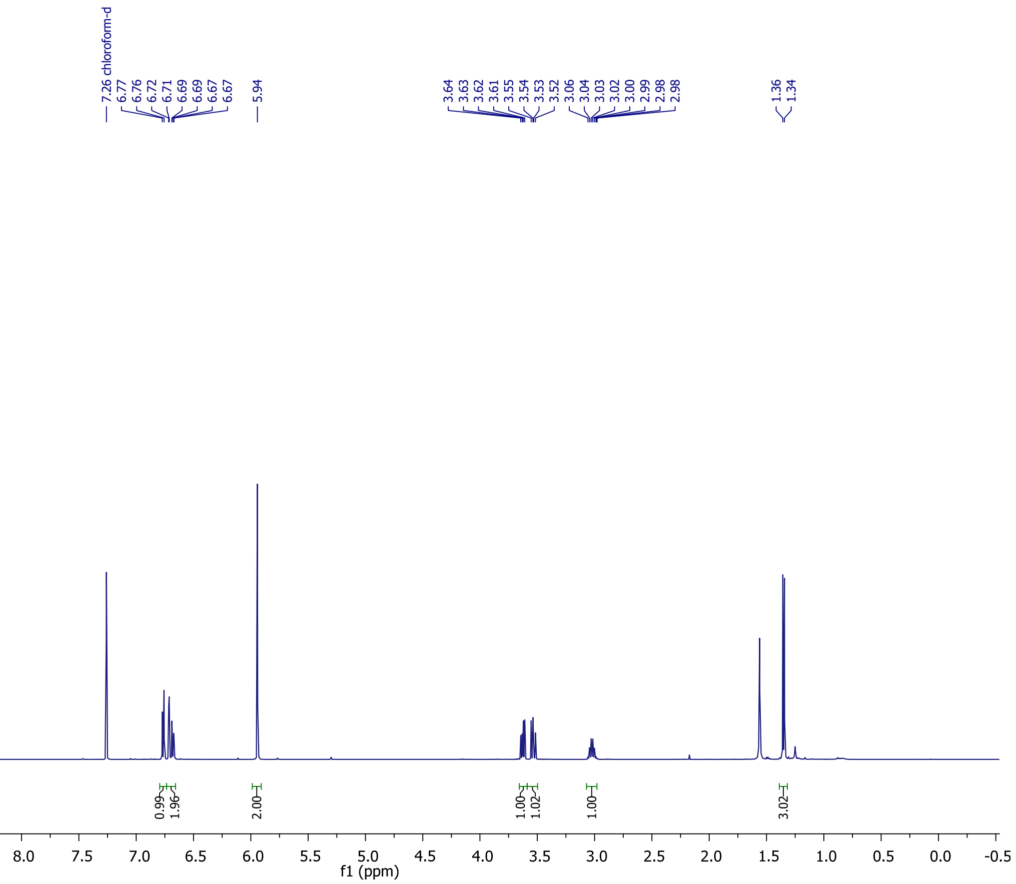

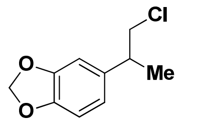

^{13}C NMR, 125 MHz, $CDCl_3$

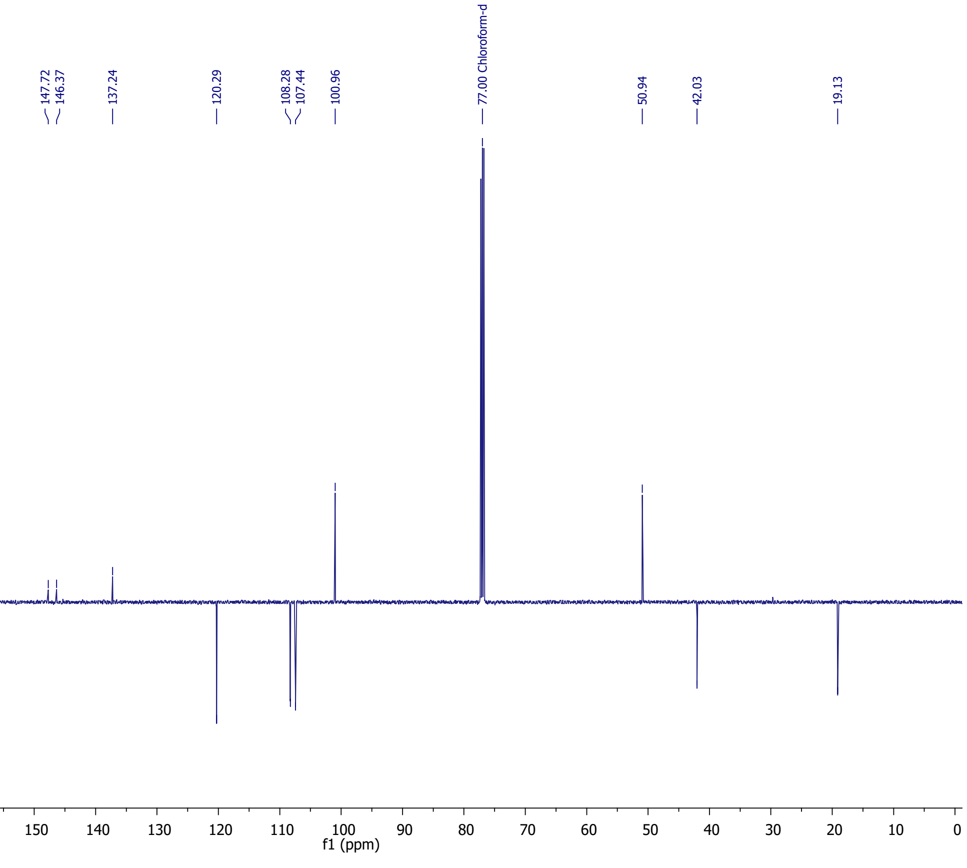

(1-Chloro-2-propanyl)benzene (35)

^{1}H NMR, 500 MHz, $CDCl_3$

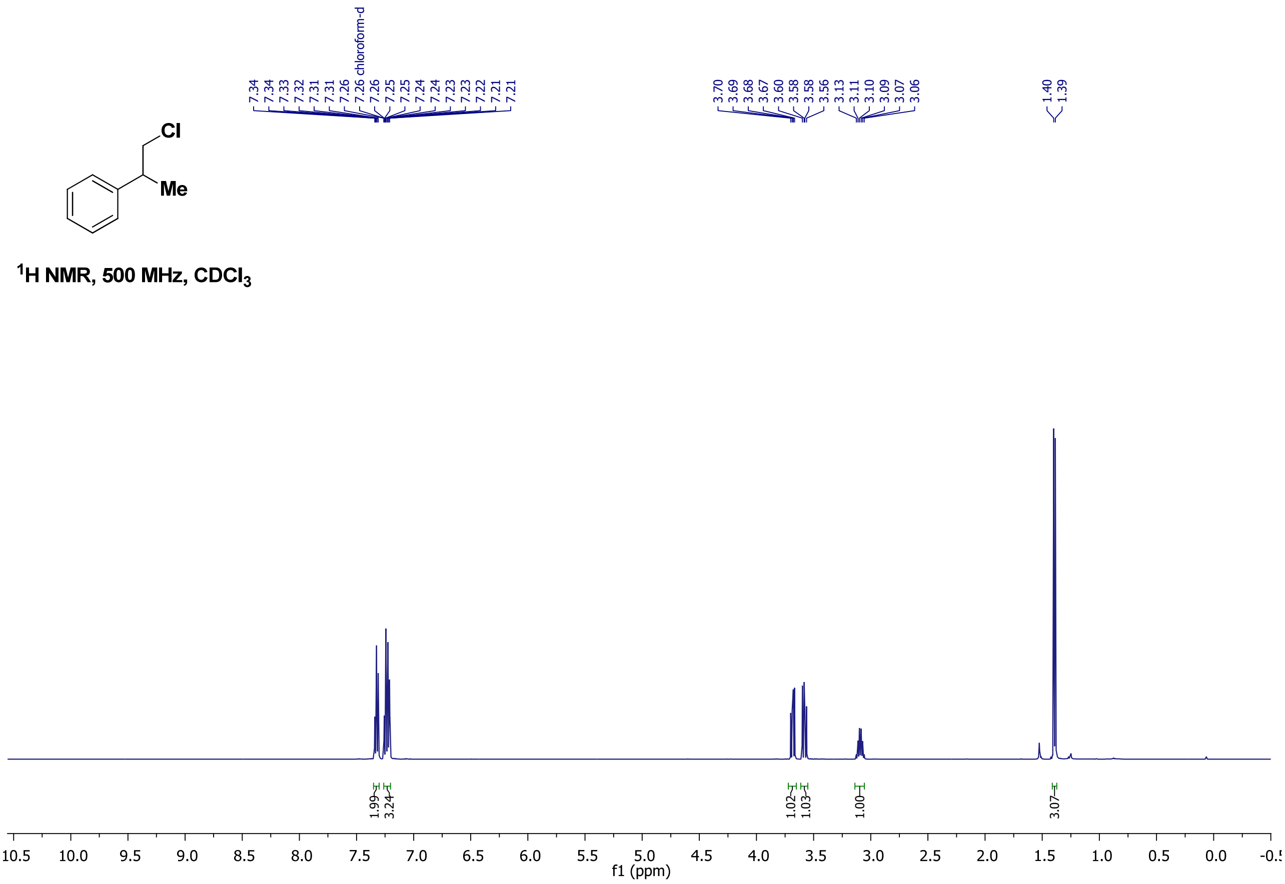

^{13}C NMR, 125 MHz, $CDCl_3$

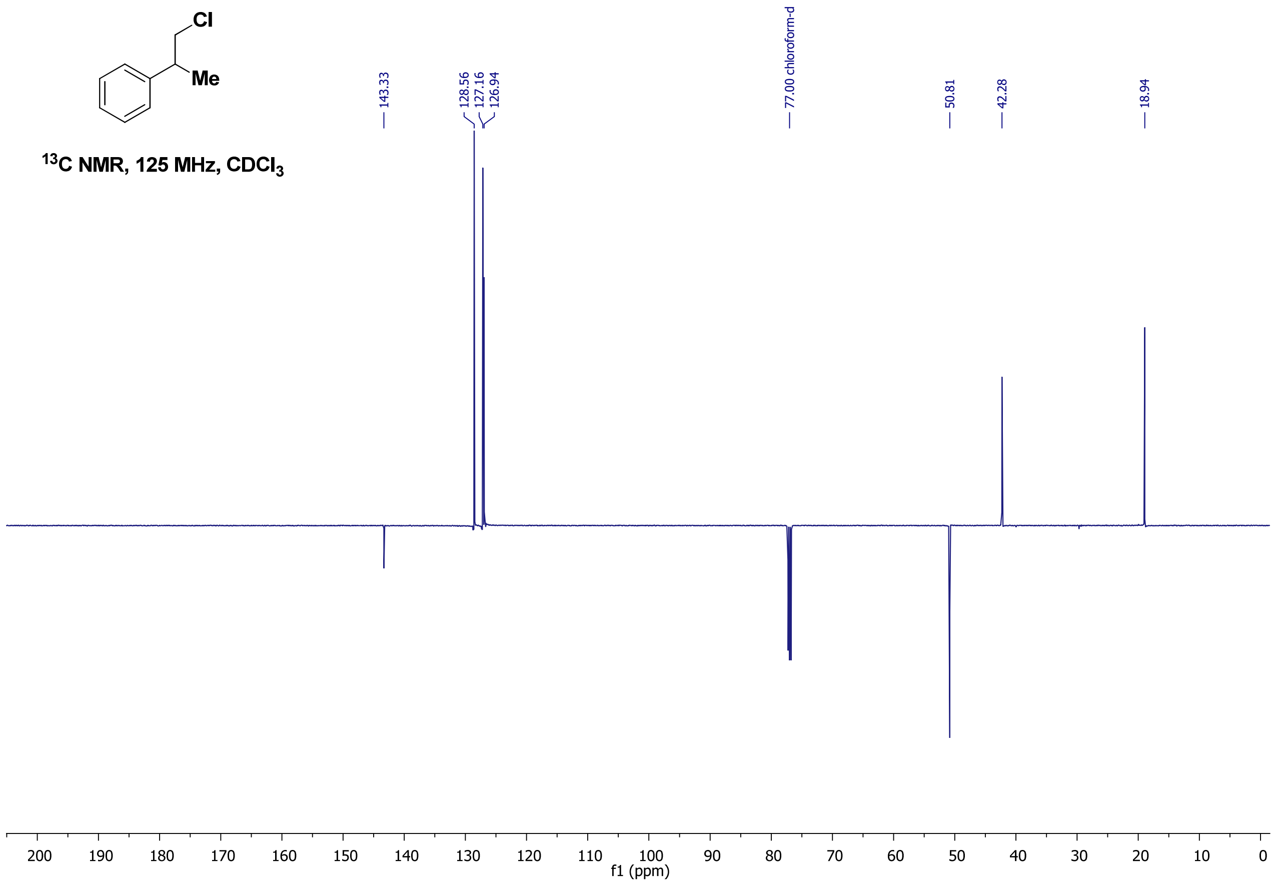

(1-Chloro-2-butanyl)benzene (36)

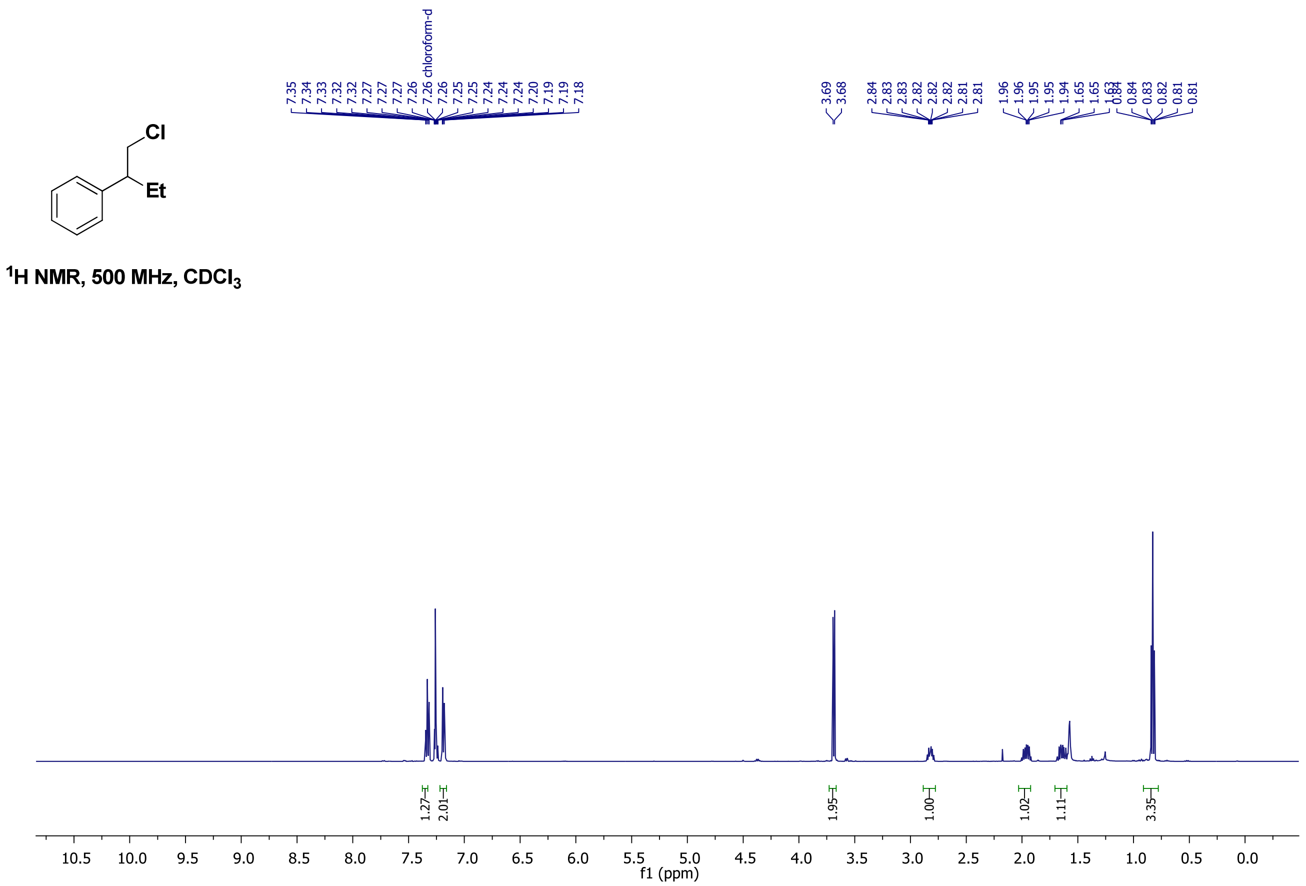

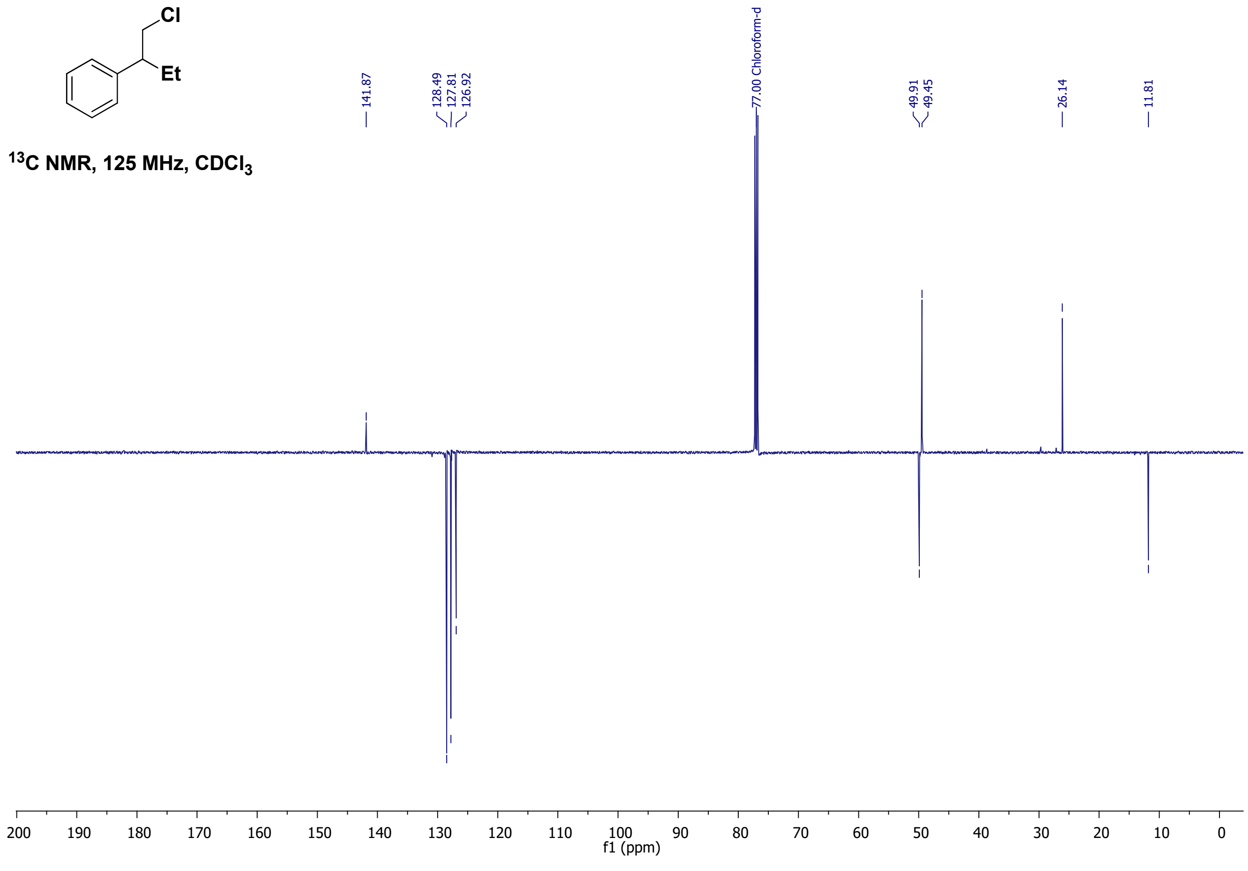

(1-Chloro-2-pentanyl)benzene (37)

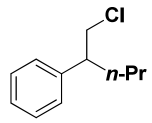

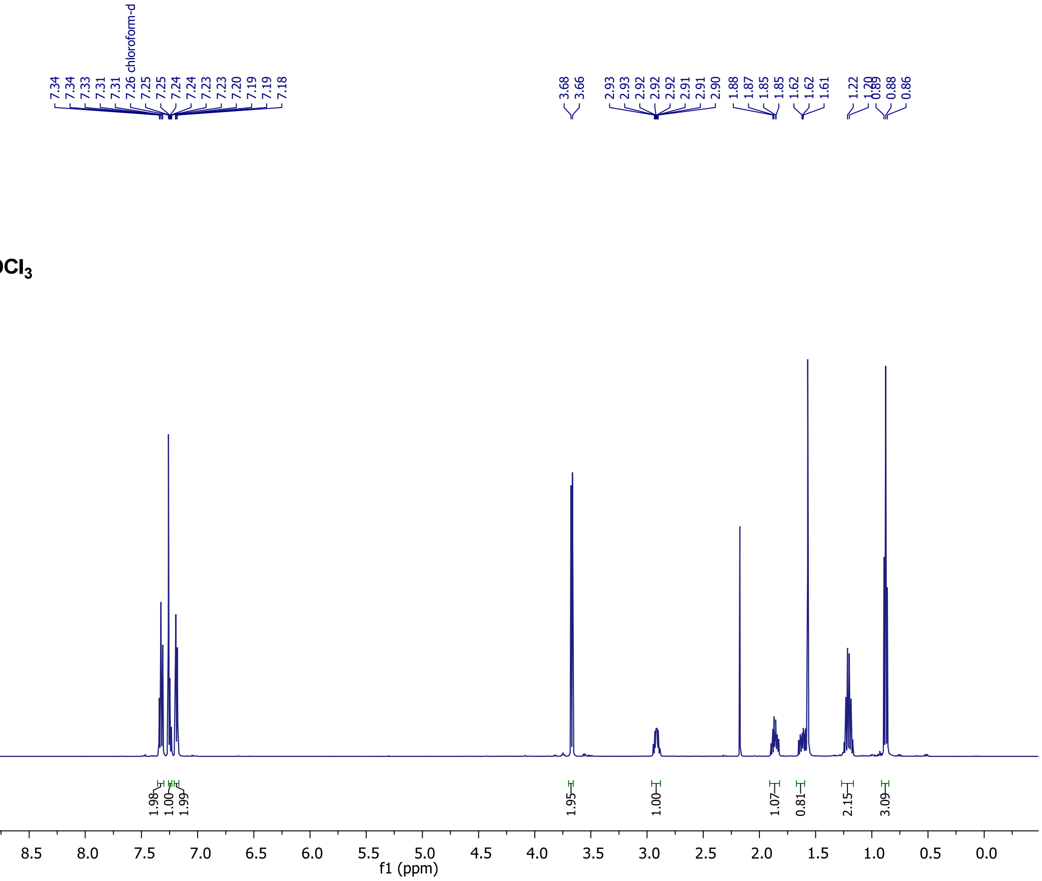

^{1}H NMR, 500 MHz, $CDCl_3$

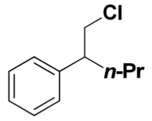

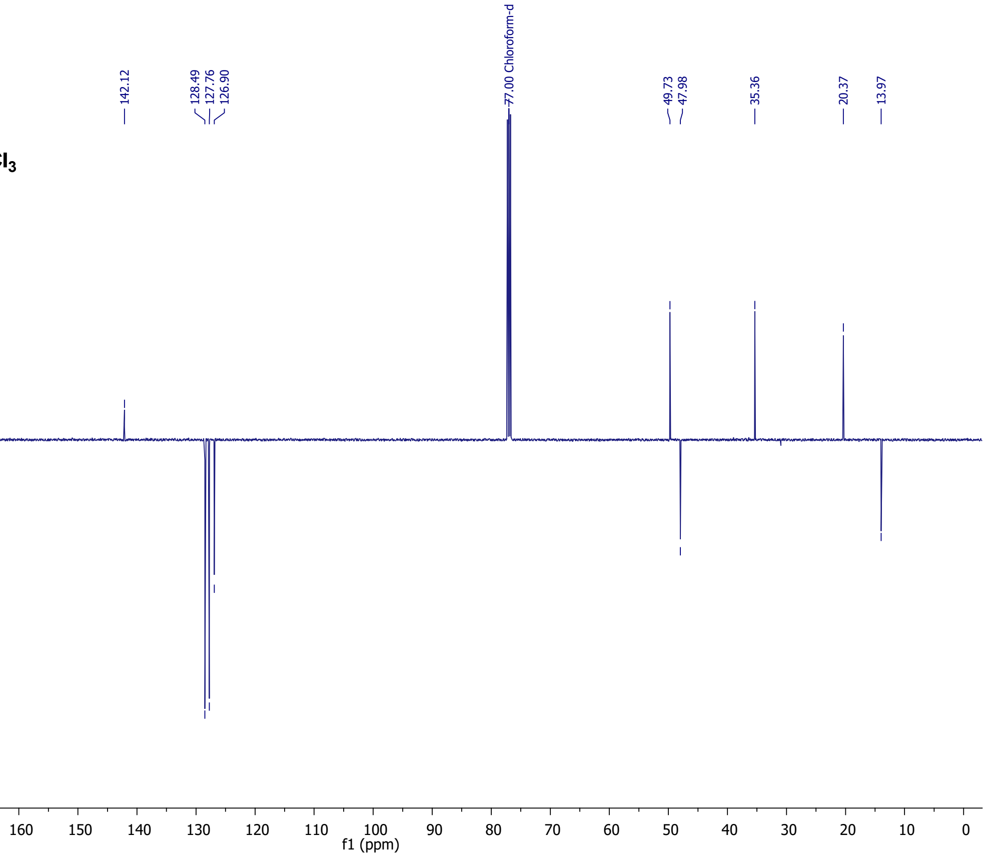

^{13}C NMR, 125 MHz, $CDCl_3$

(1-Chloro-2-hexanyl)benzene (38)

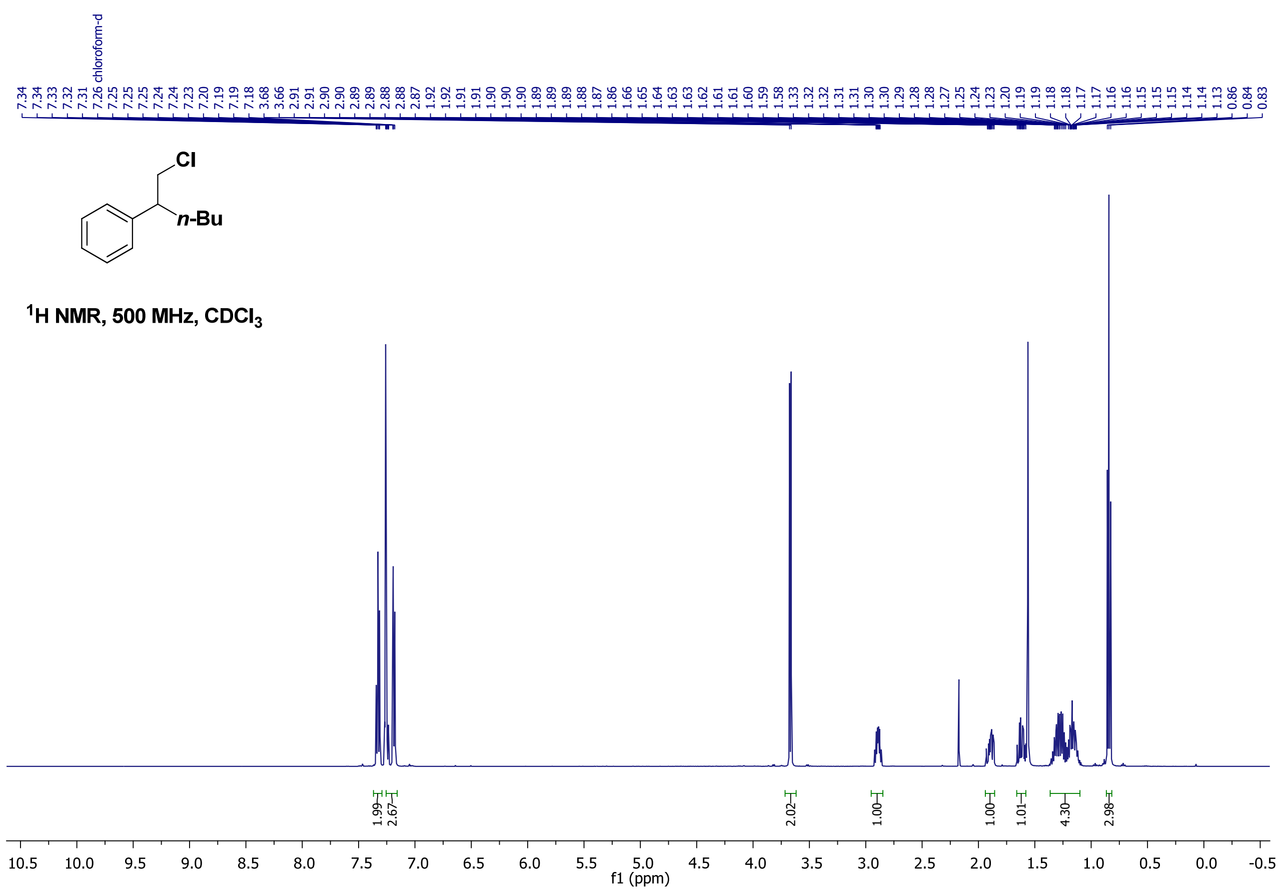

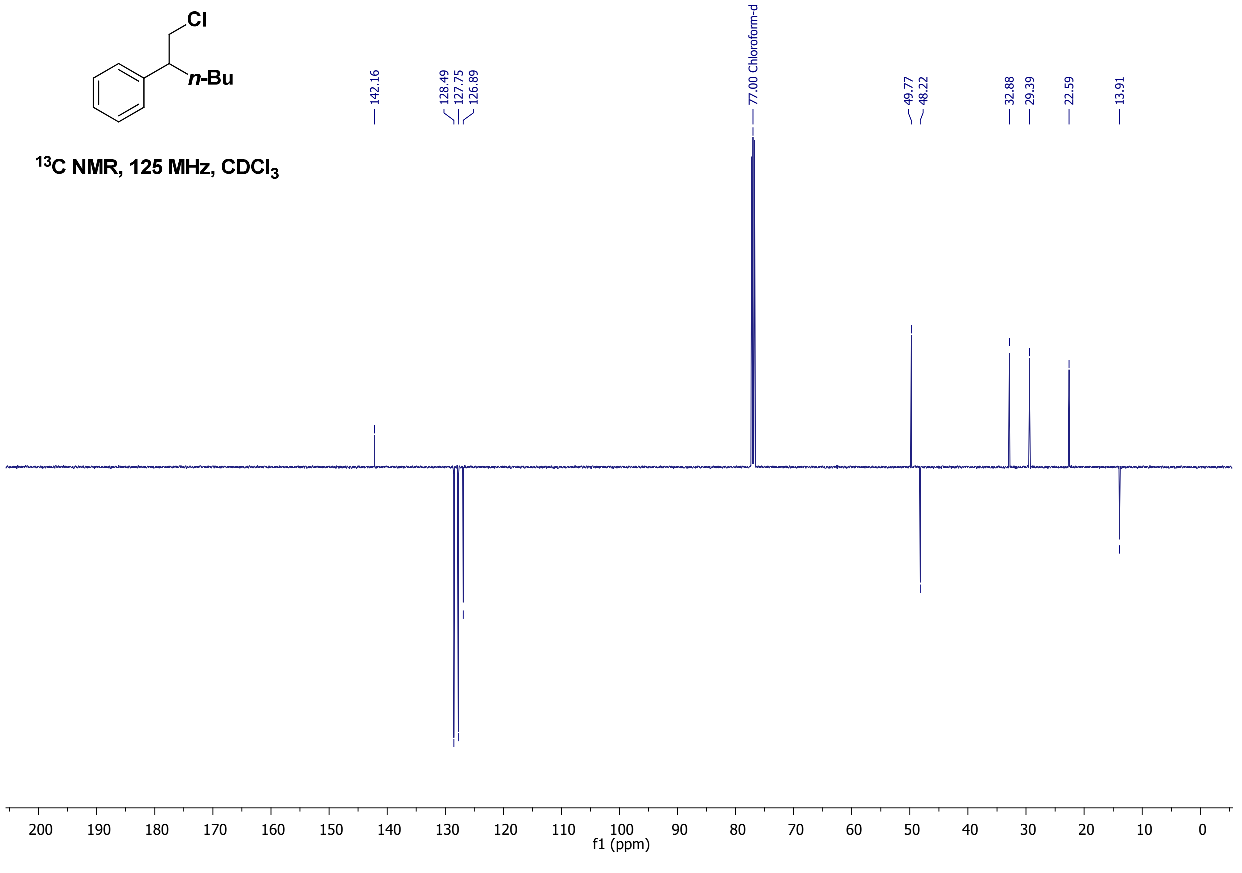

(1,4-Dichloro-2-butanyl)benzene (39)

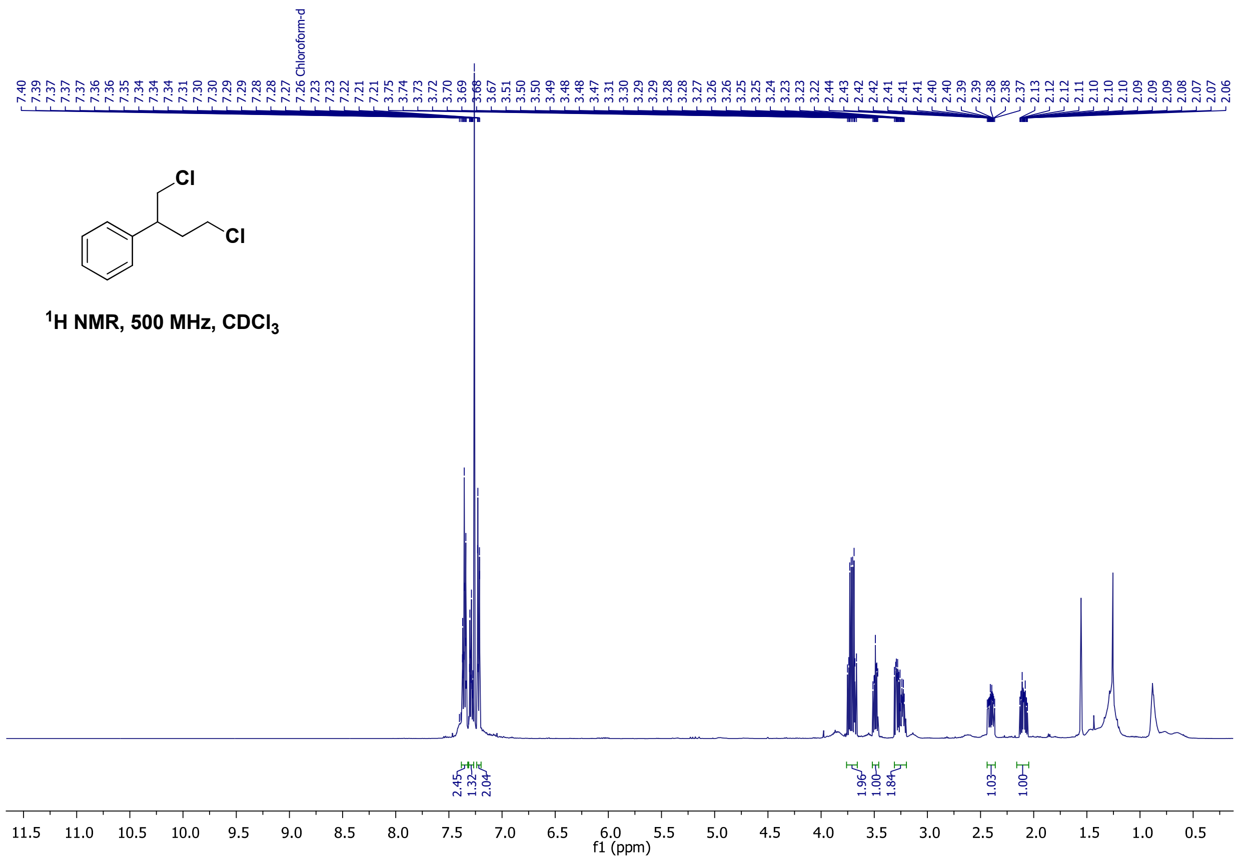

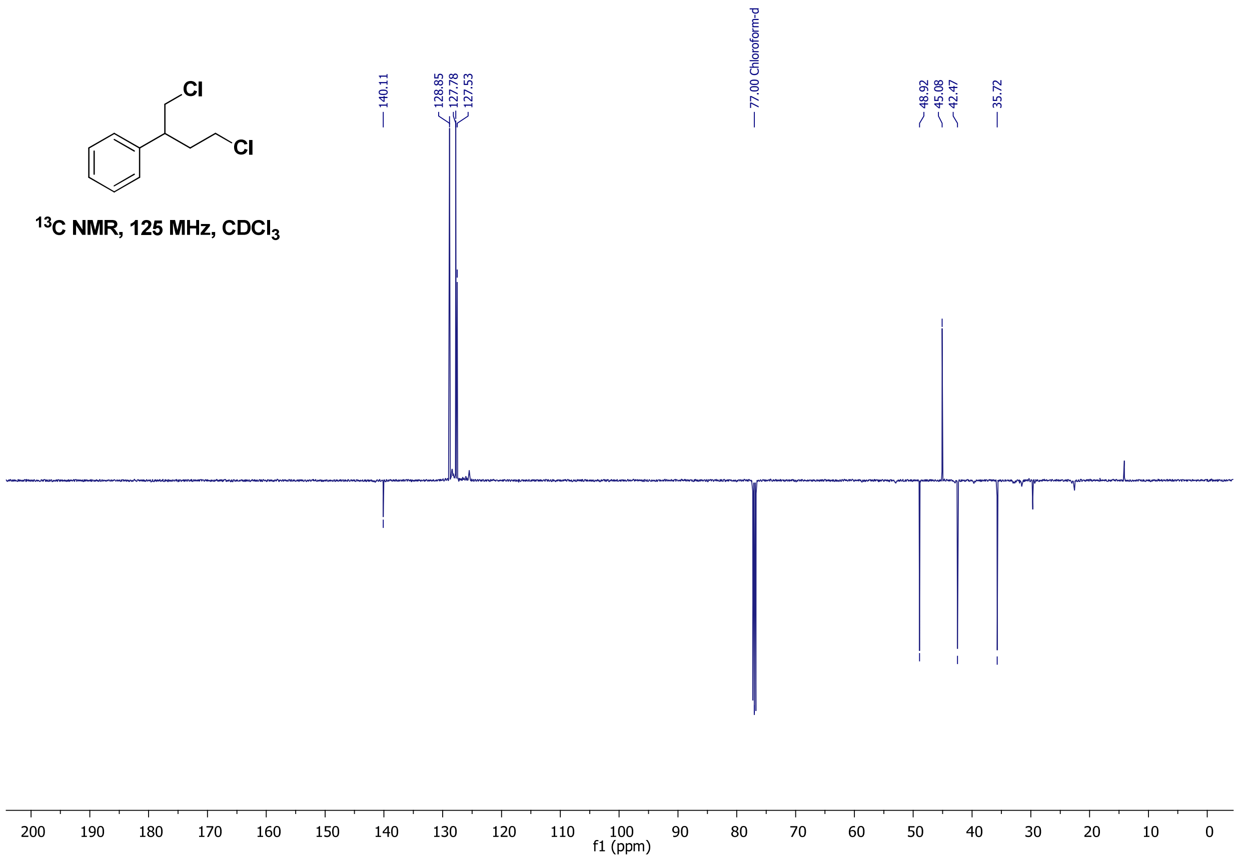

1,1'-(3-Chloro-1,2-propanediyl)dibenzene (40)

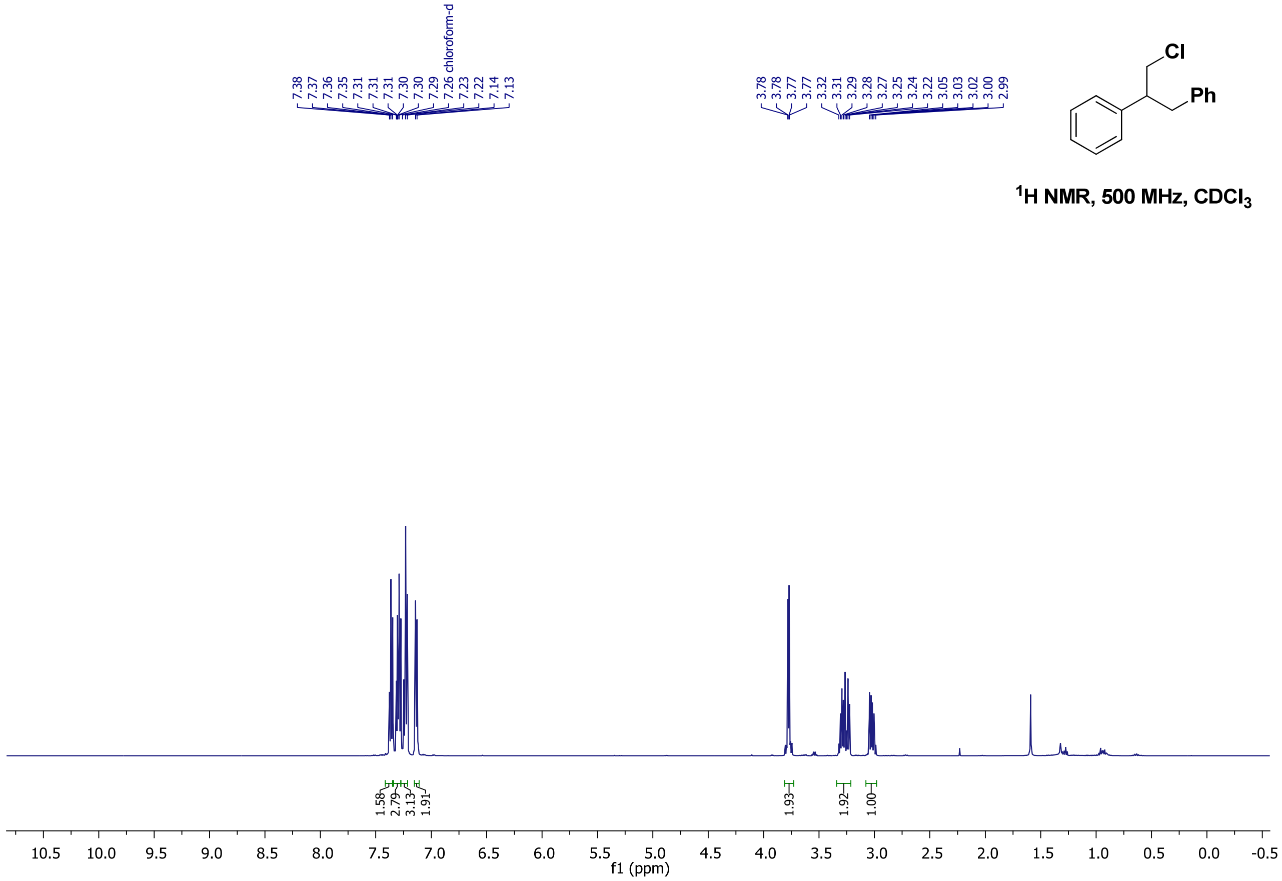

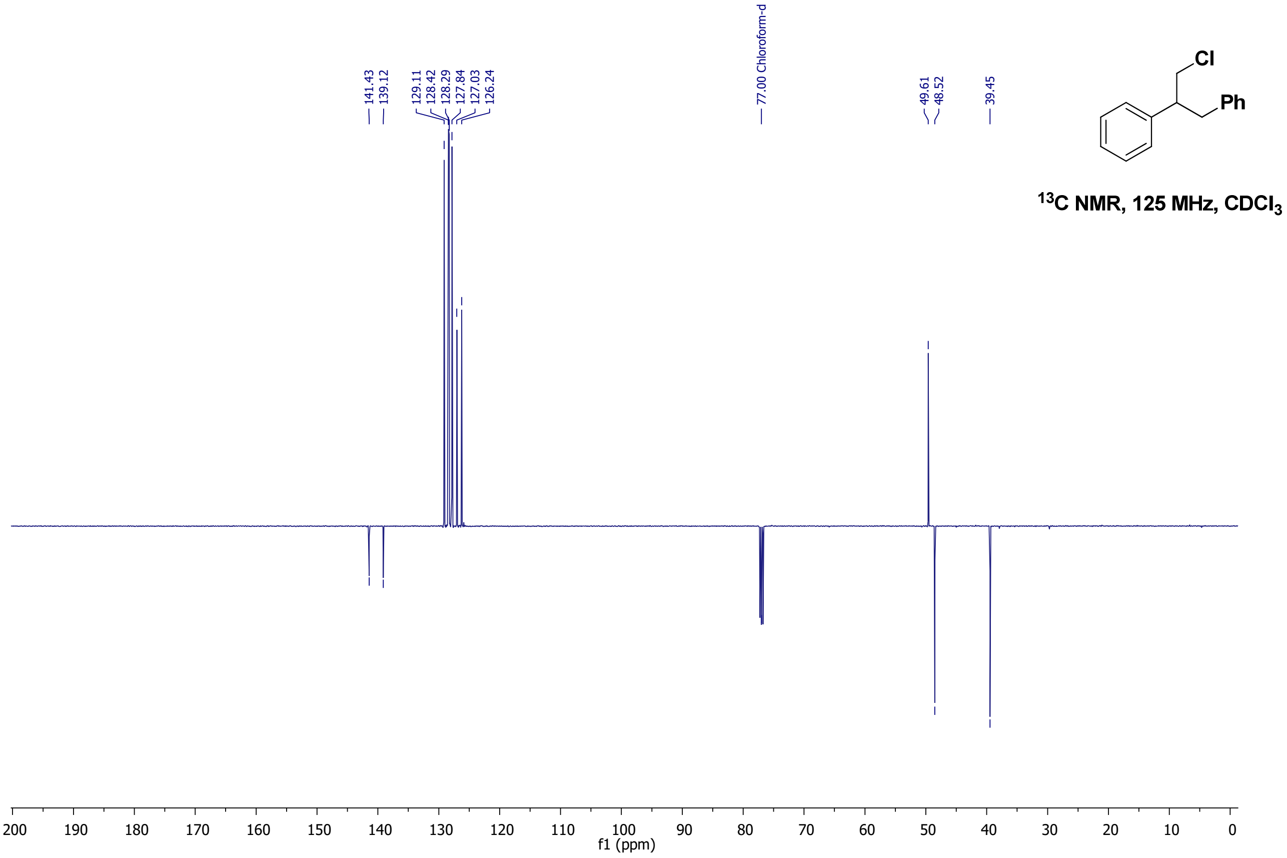

(2-Chloro-1-cyclohexylethyl)benzene (41)

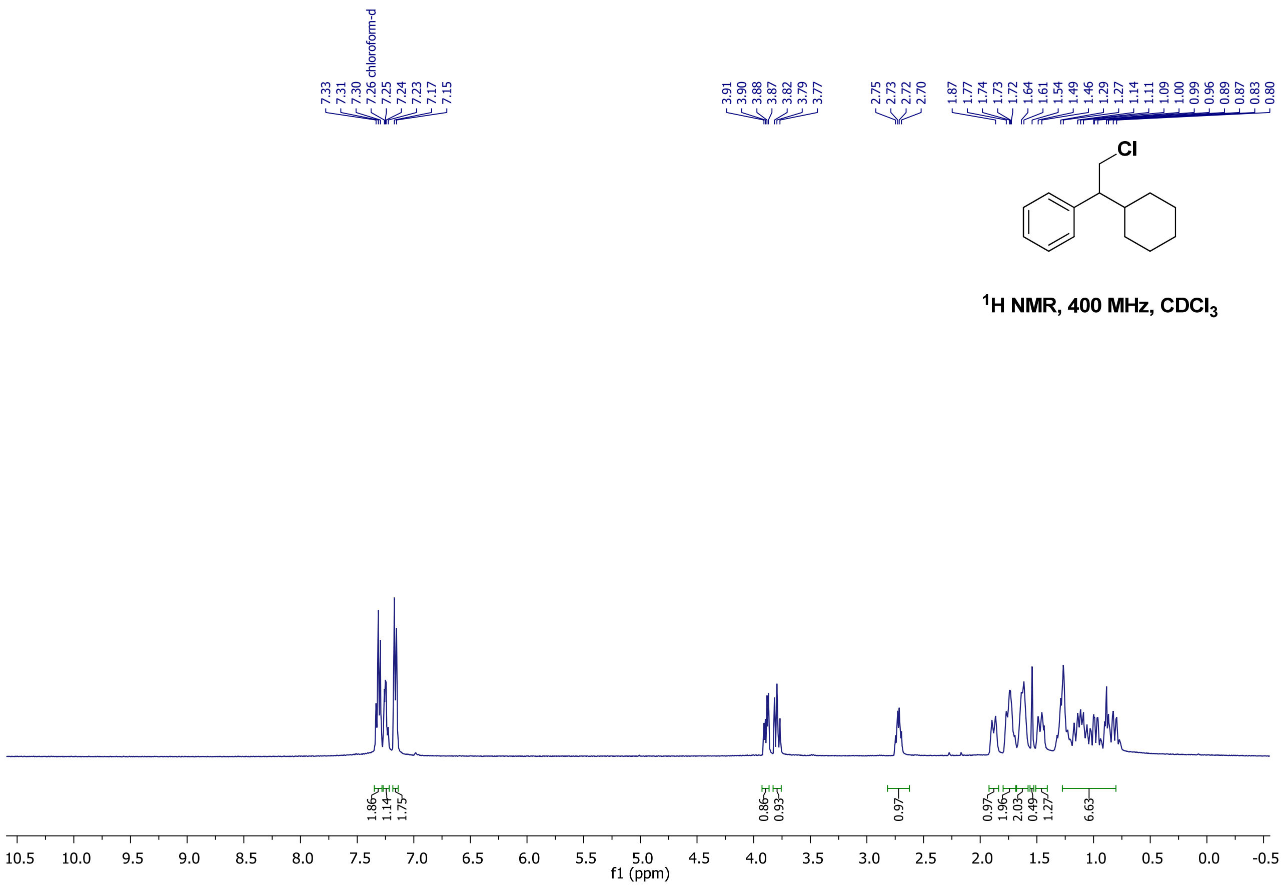

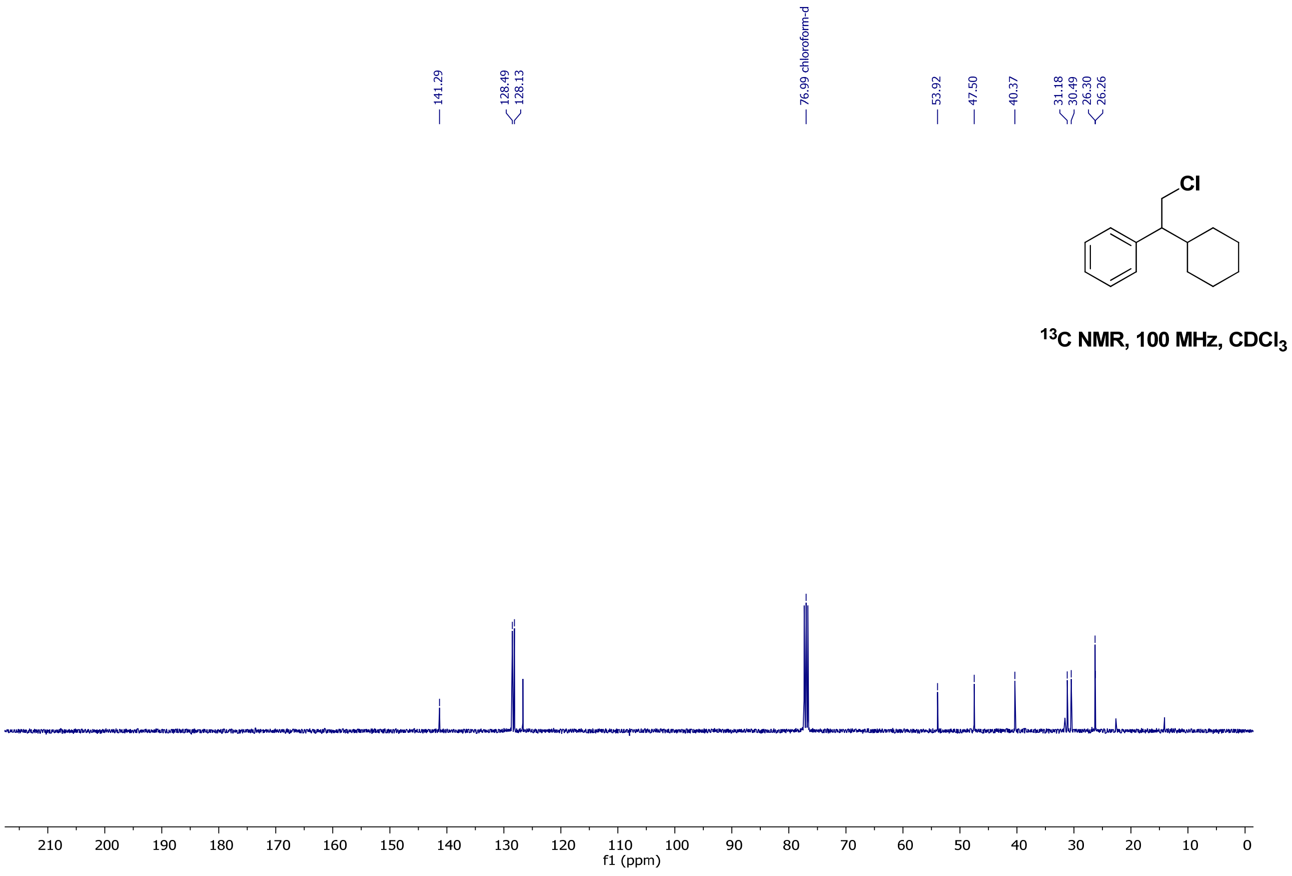

4-(1-Chloro-2-propanyl)-5-methyl-1-phenyl-1*H*-pyrazole (42)

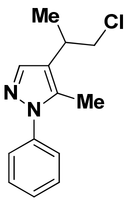

^{1}H NMR, 400 MHz, $CDCl_3$

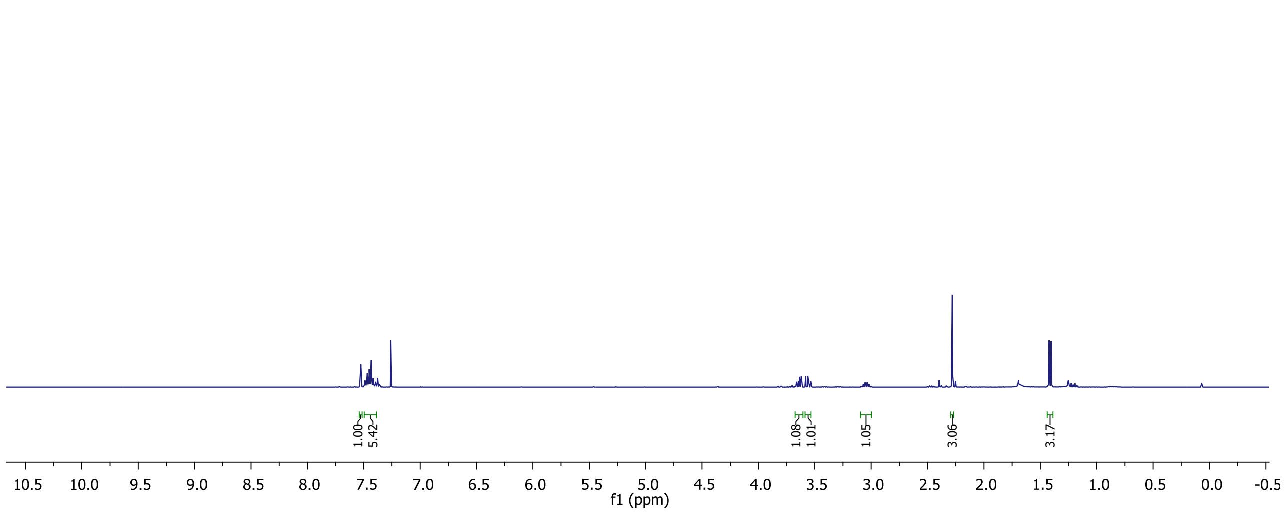

^{13}C NMR, 100 MHz, $CDCl_3$

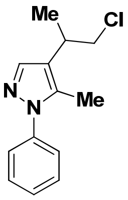

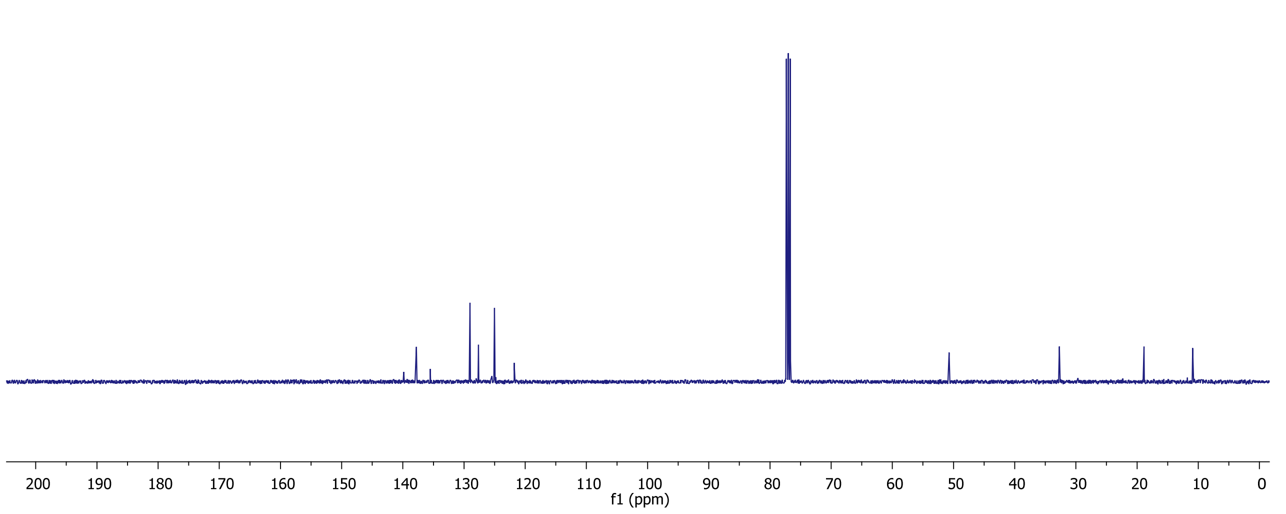

(4-Chloro-3-methyl-1-butyn-1-yl)benzene (43)

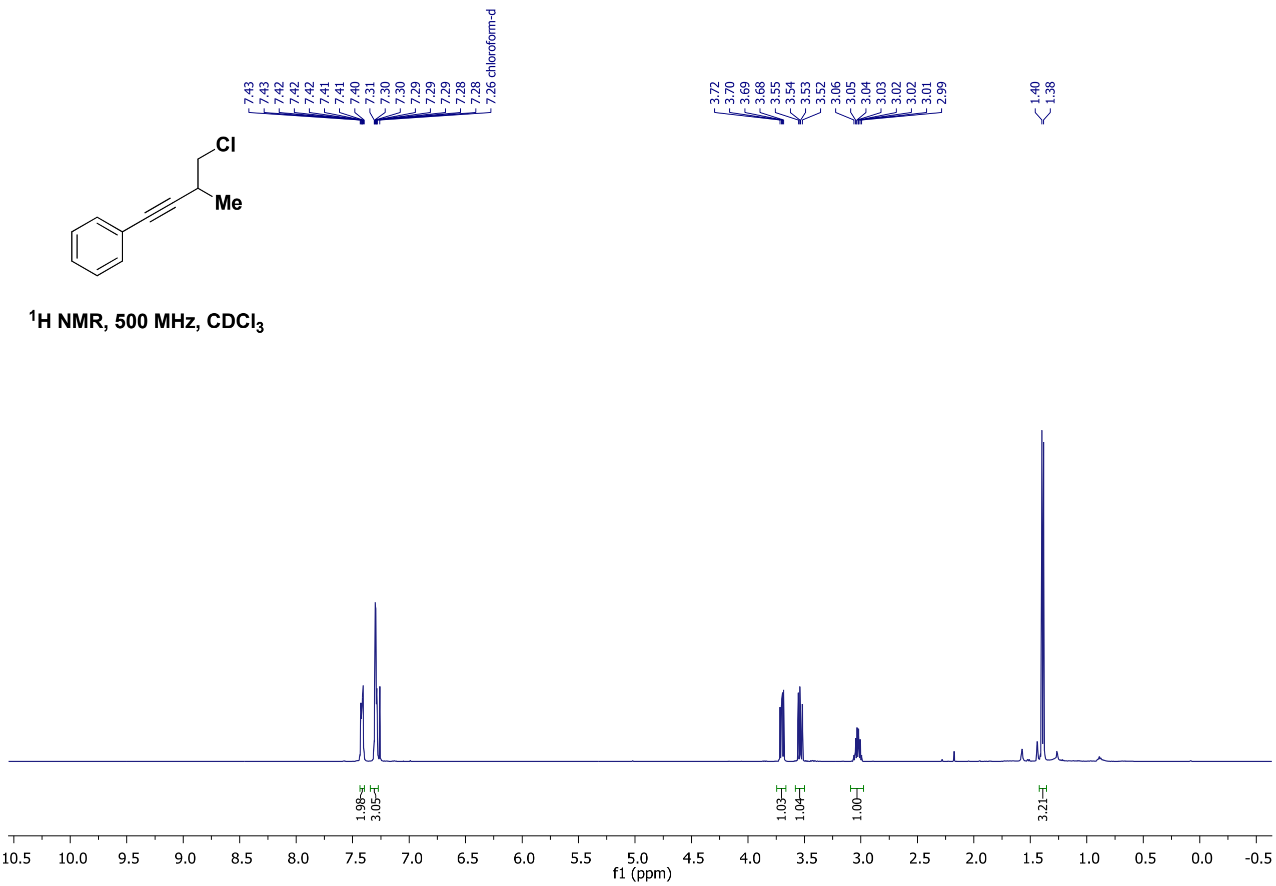

^{1}H NMR, 500 MHz, $CDCl_3$

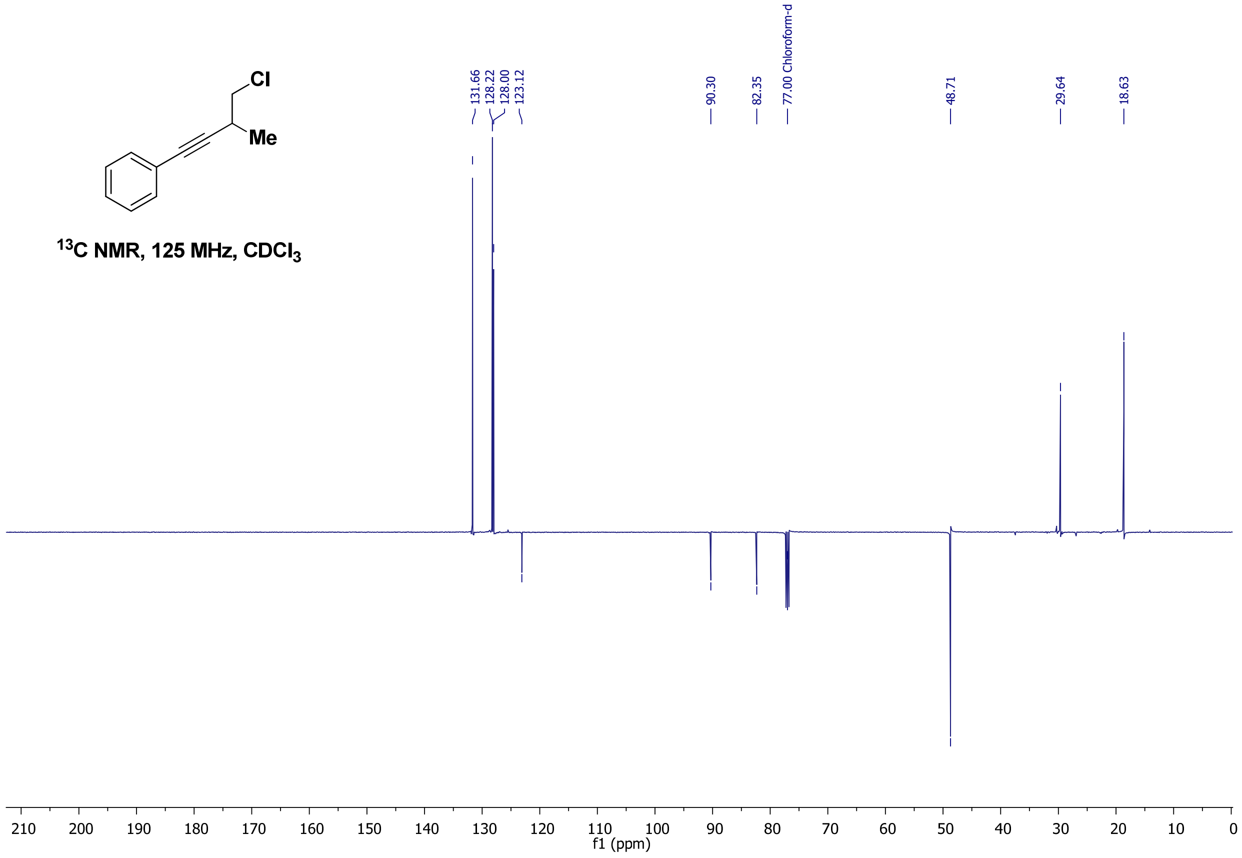

^{13}C NMR, 125 MHz, $CDCl_3$

1,1'-(2-Chloro-1,1-ethanediyl)dibenzene (44)

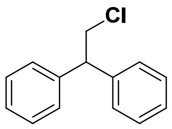

^{1}H NMR, 500 MHz, $CDCl_3$

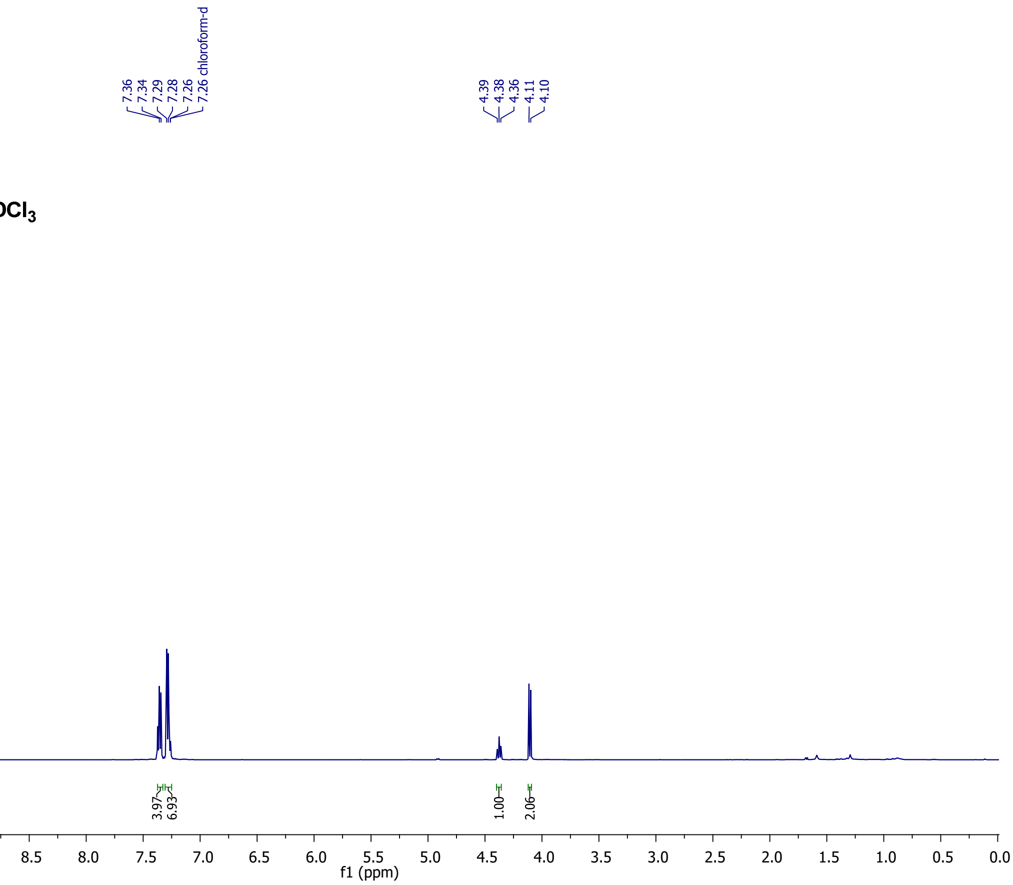

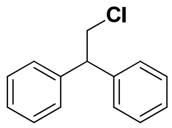

^{13}C NMR, 125 MHz, $CDCl_3$

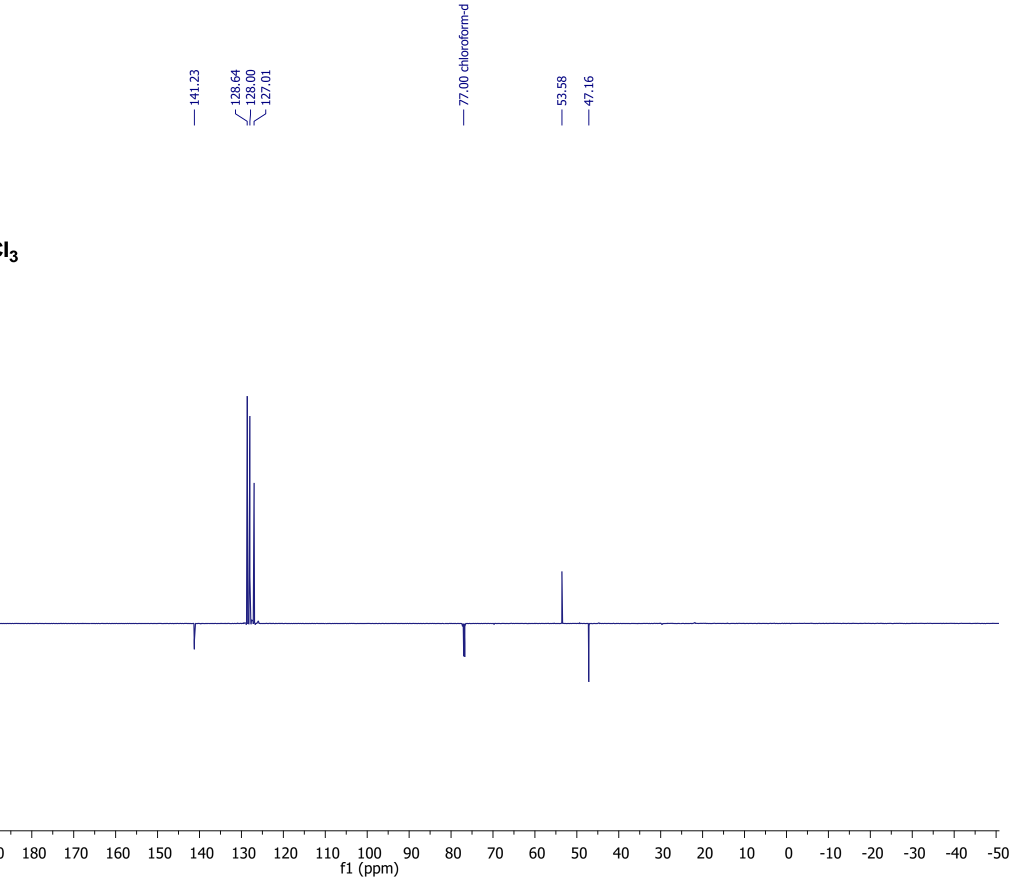

1-(2-Chloro-1-phenylethyl)-4-methylbenzene (45)

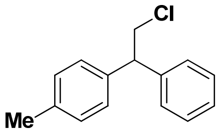

^{1}H NMR, 500 MHz, $CDCl_3$

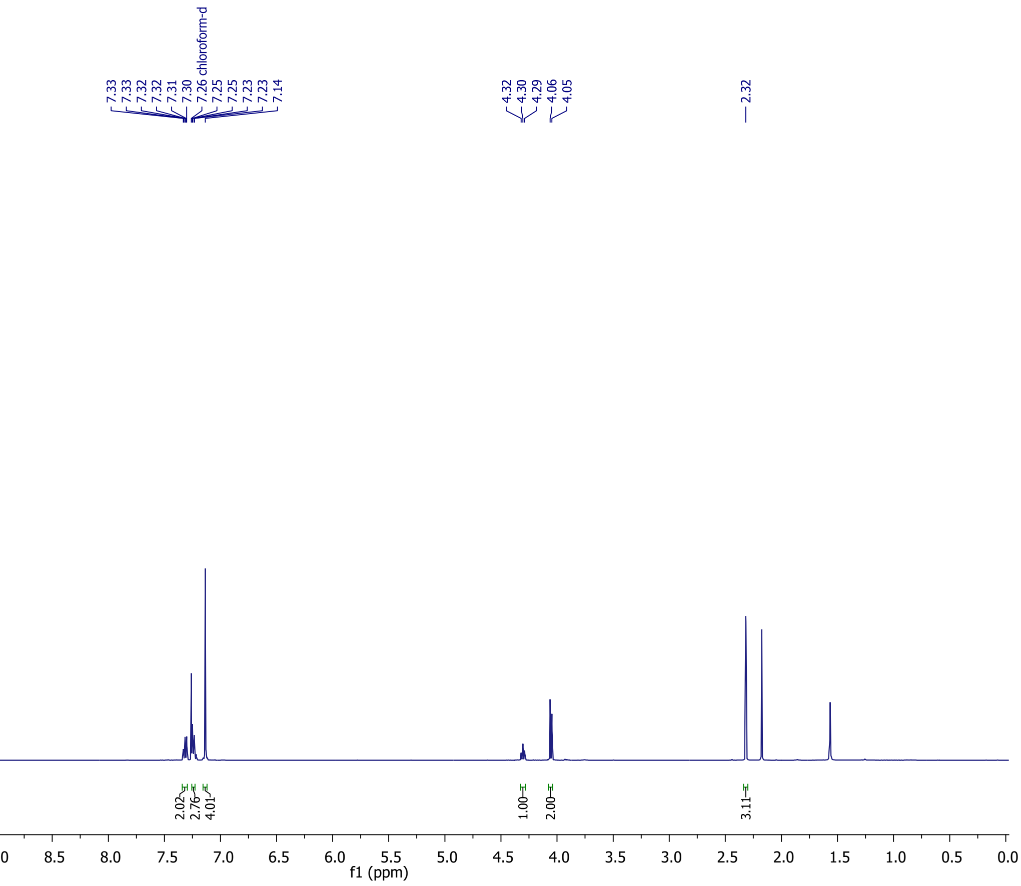

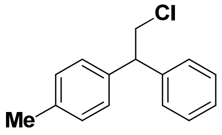

^{13}C NMR, 125 MHz, $CDCl_3$

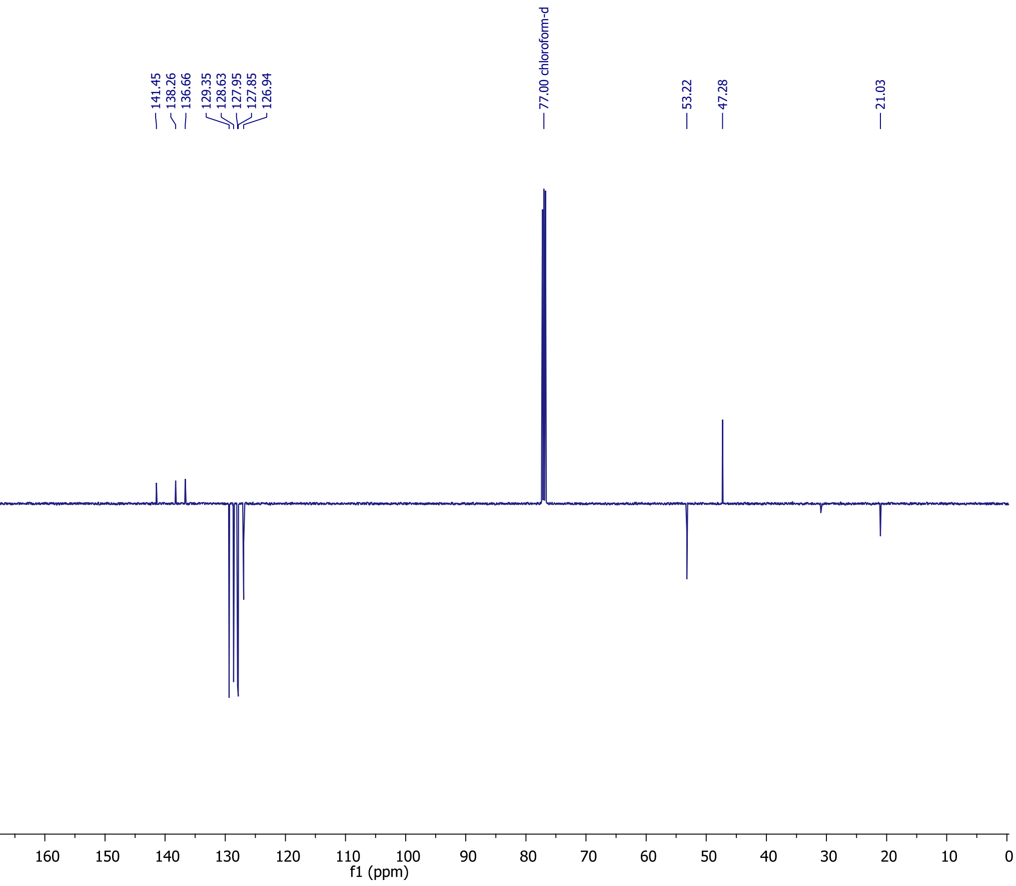

1-(2-Chloro-1-phenylethyl)-4-ethylbenzene (46)

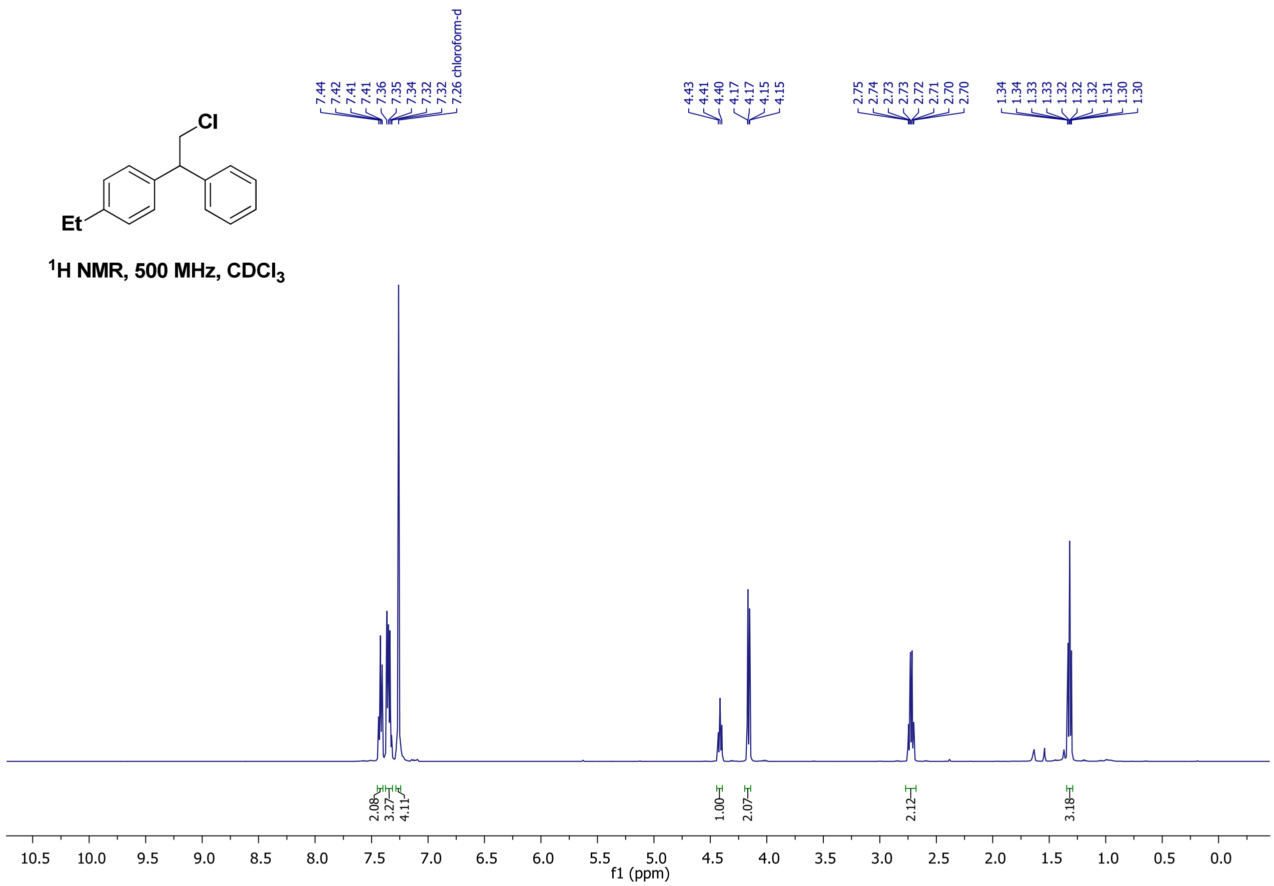

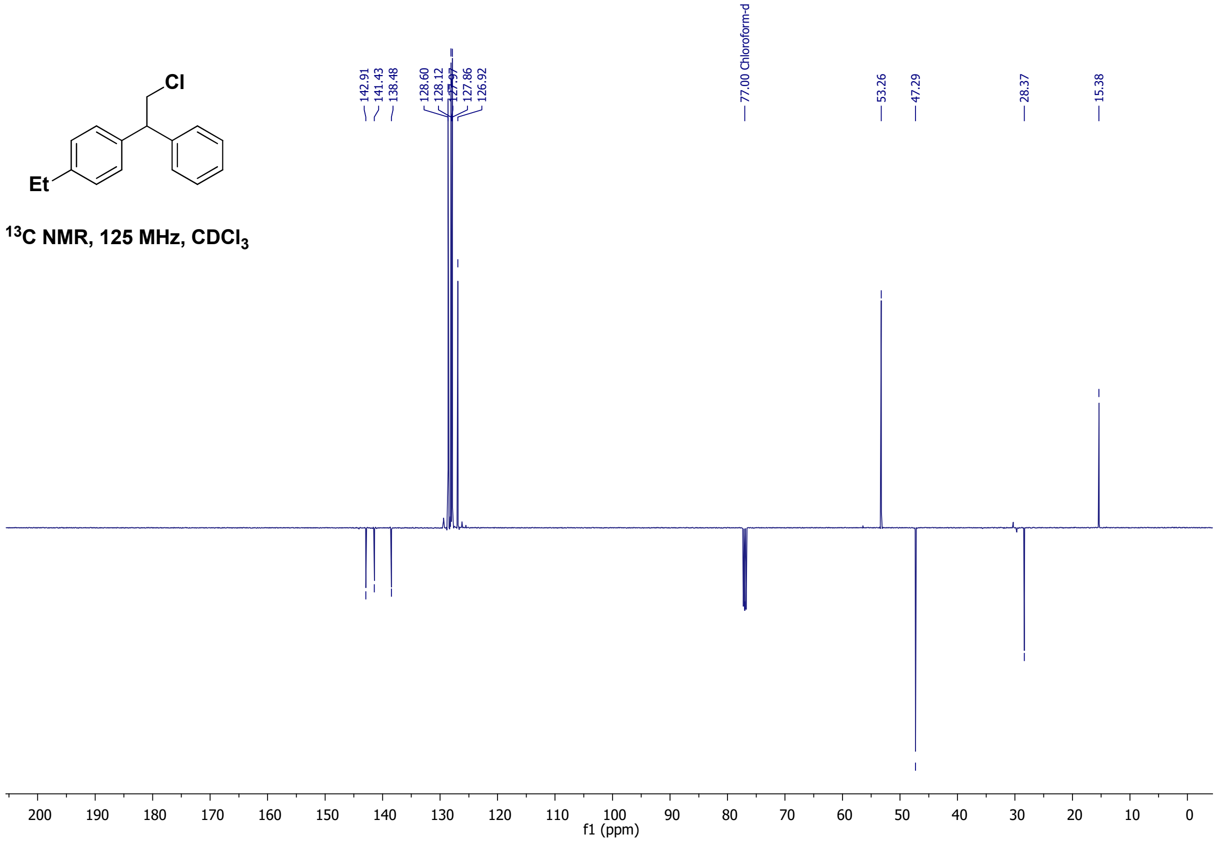

1-(2-chloro-1-phenylethyl)-4-(2-methyl-2-propanyl)benzene (47)

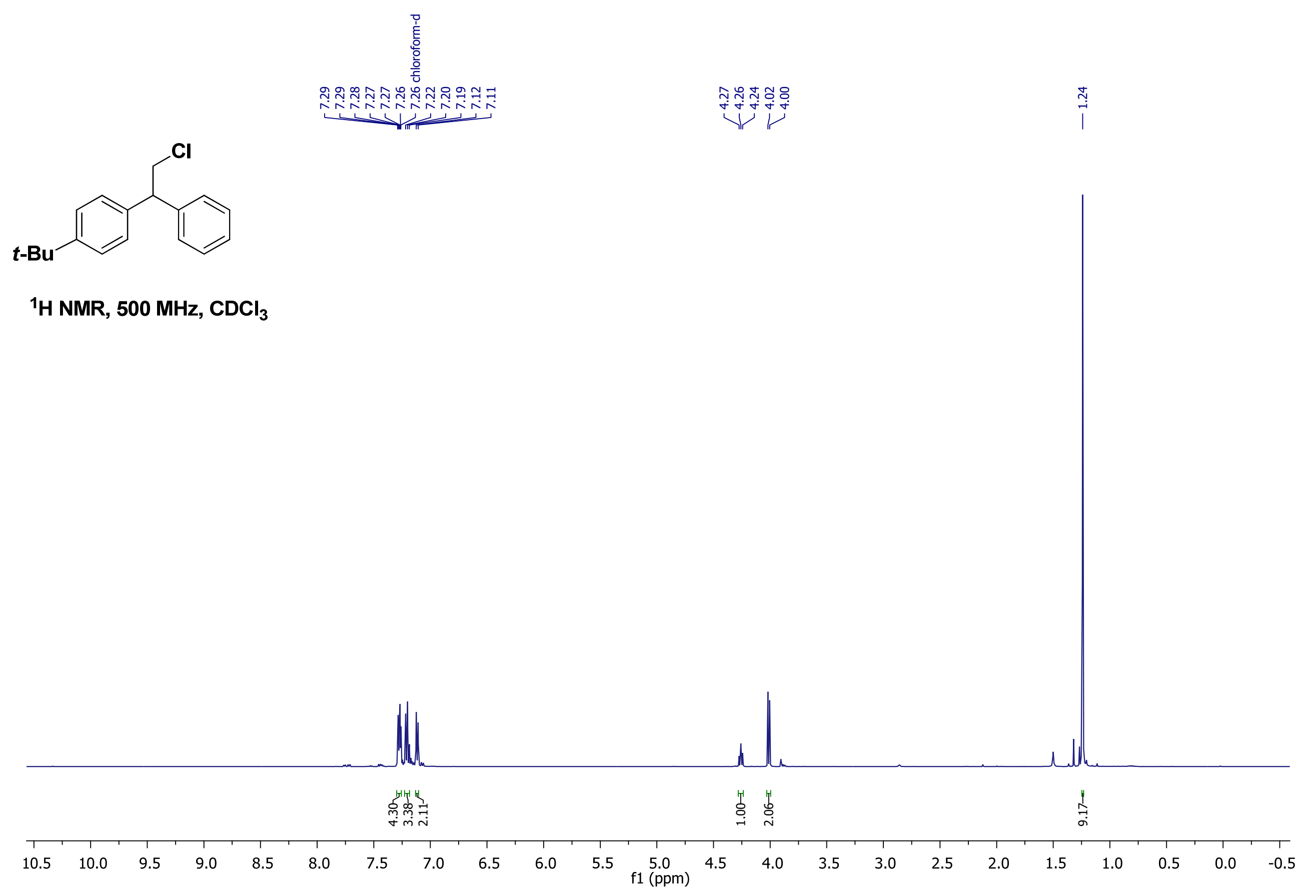

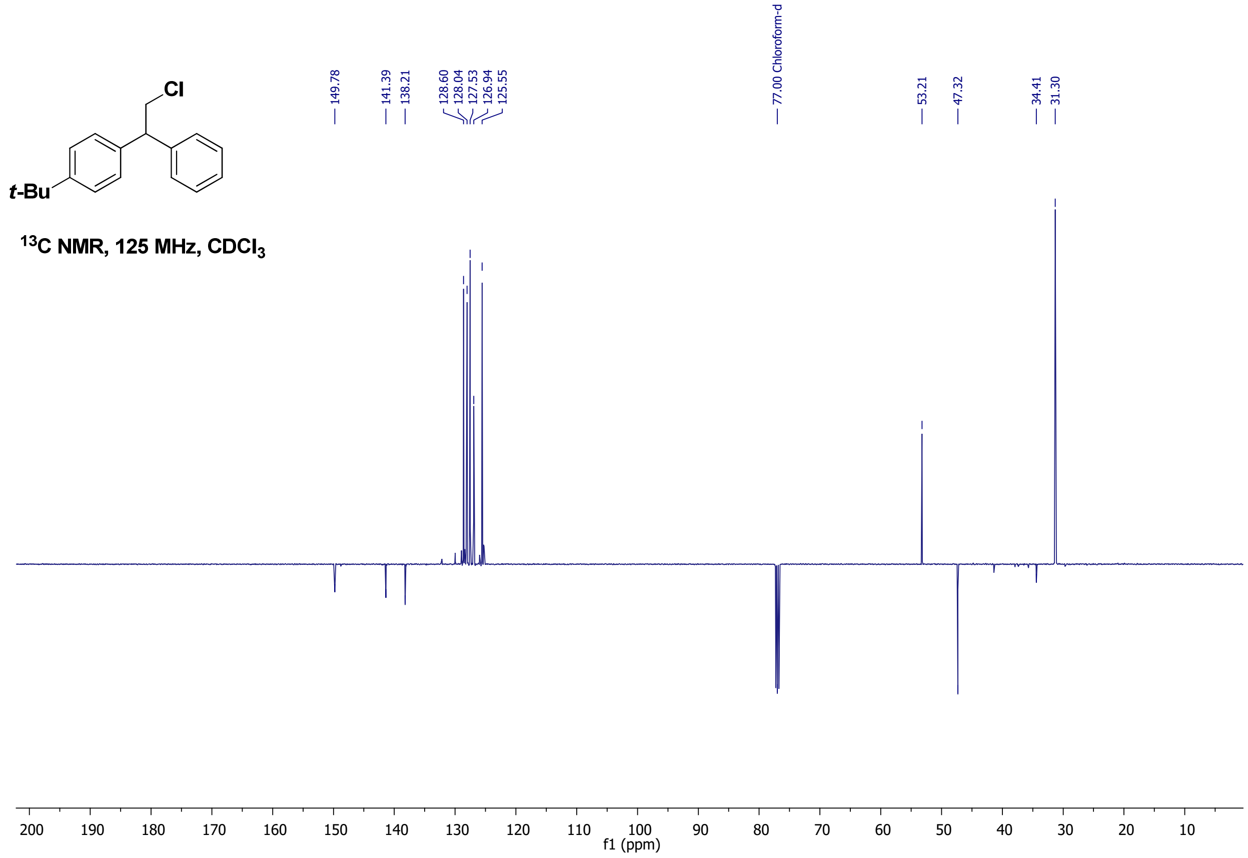

1-[4-(2-Chloro-1-phenylethyl)phenyl]adamantane (48)

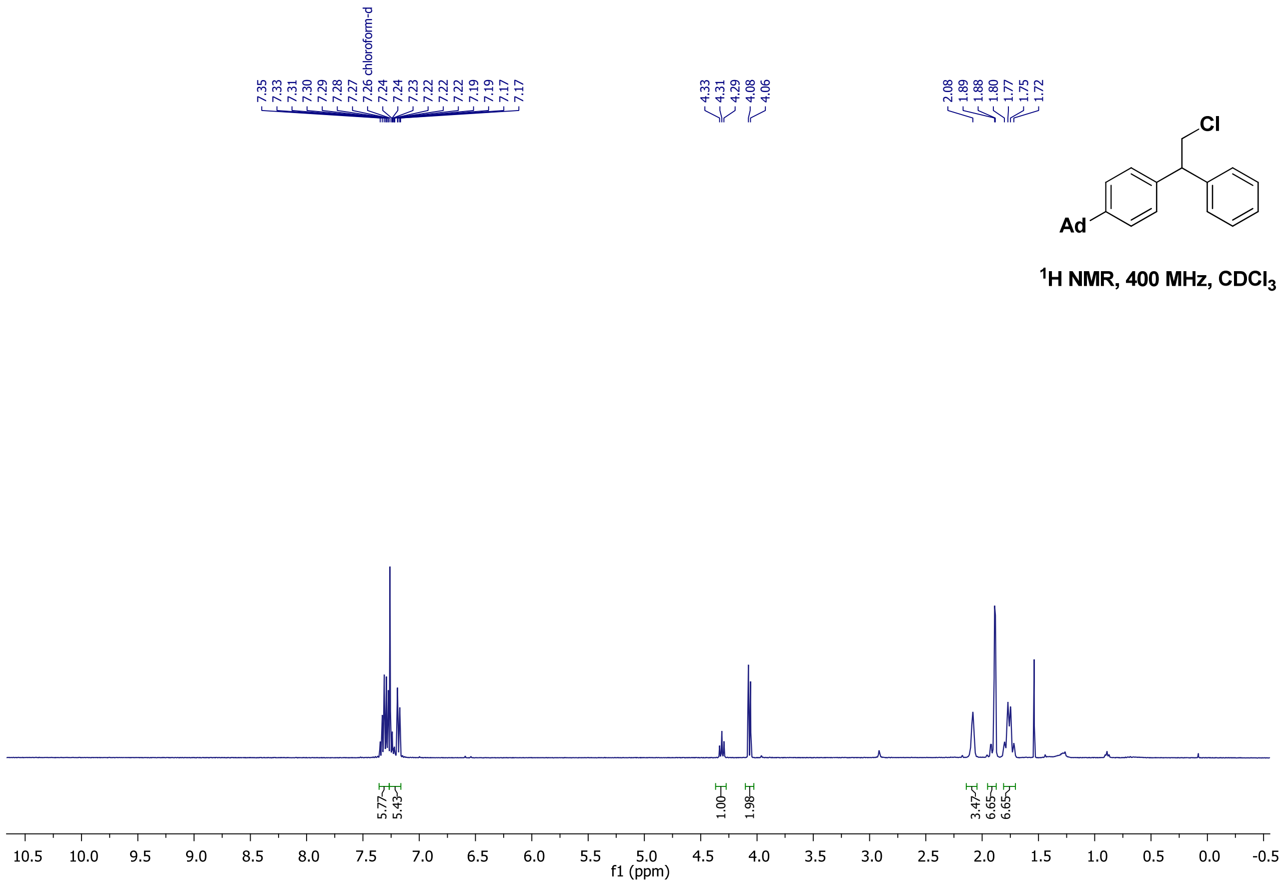

^{1}H NMR, 400 MHz, $CDCl_3$

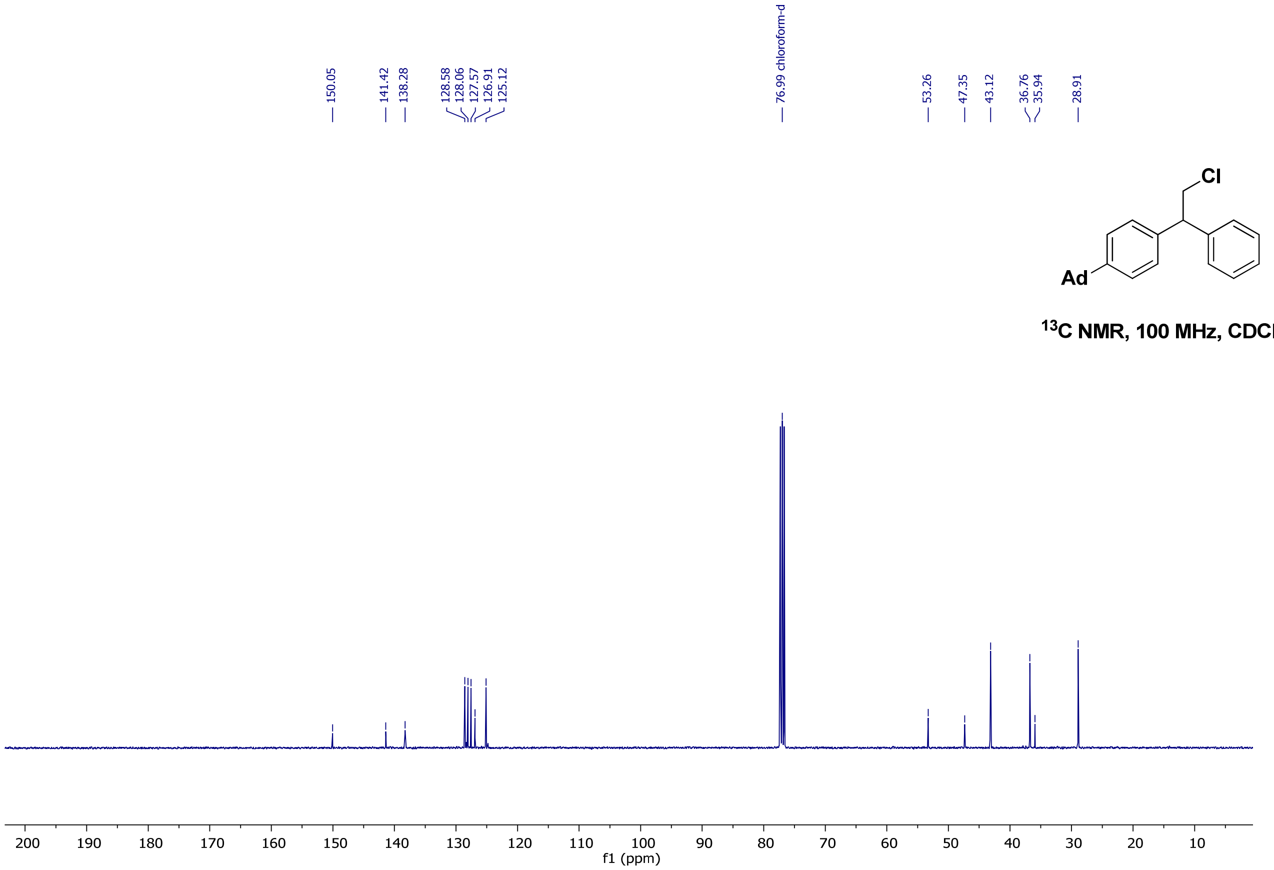

^{13}C NMR, 100 MHz, $CDCl_3$

4-(2-Chloro-1-phenylethyl)biphenyl (49)

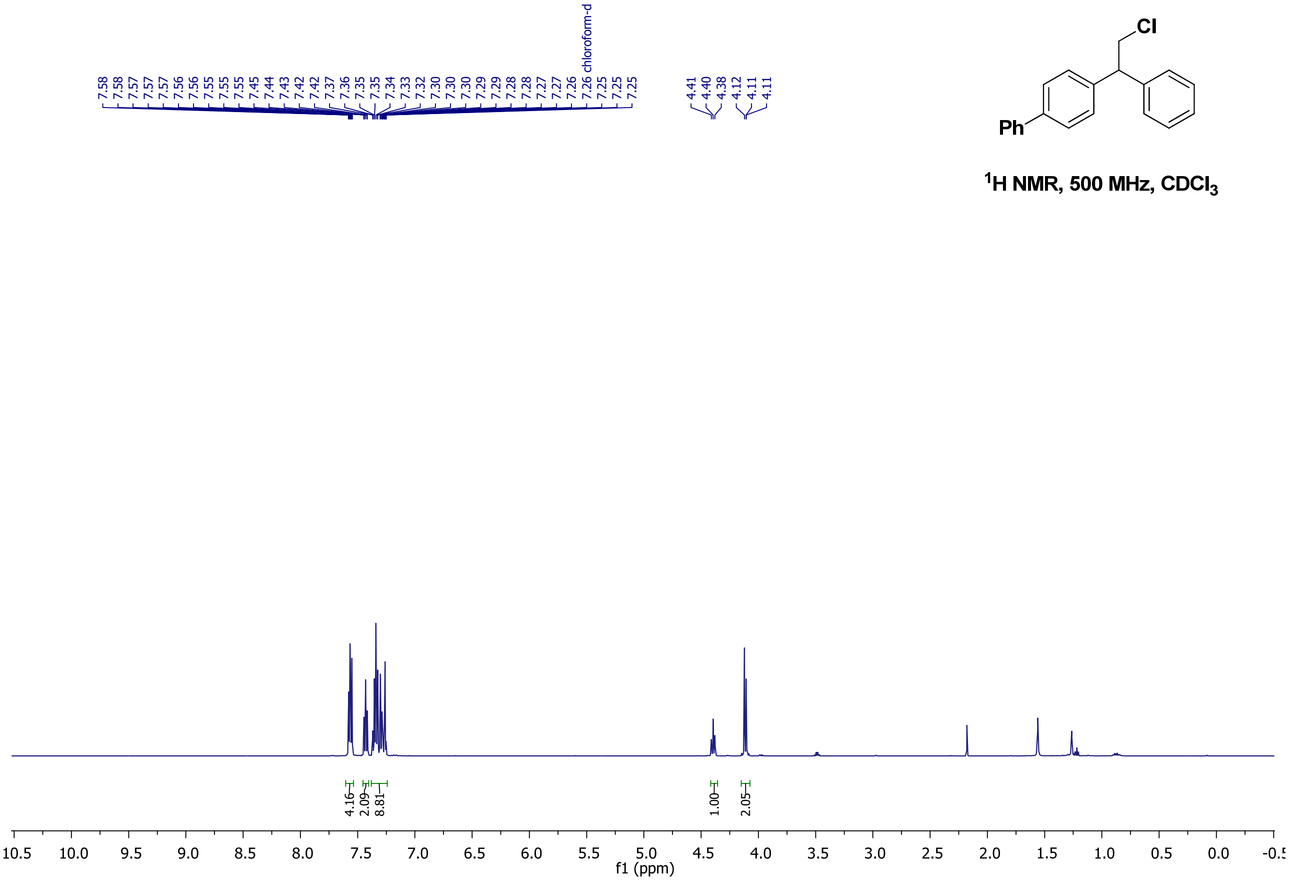

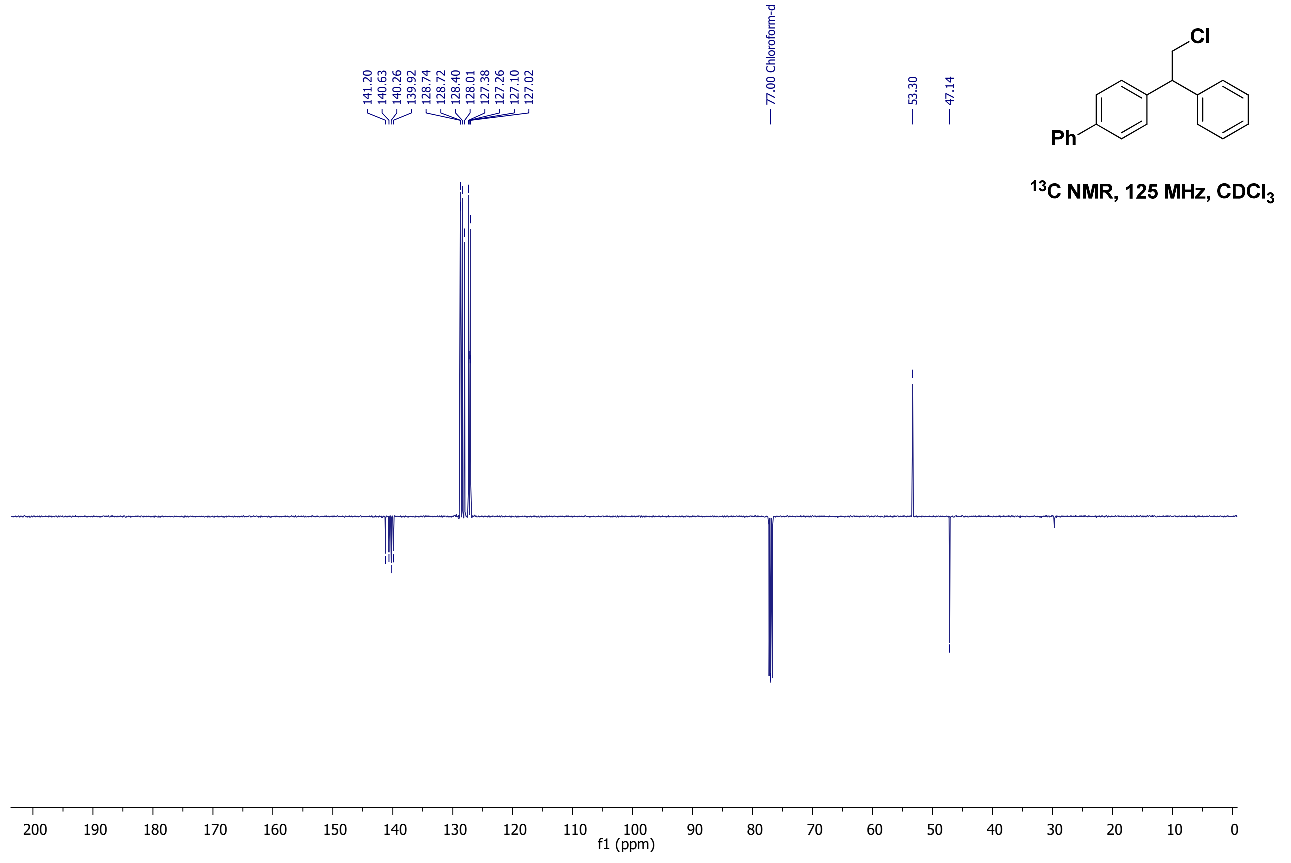

1-(2-Chloro-1-phenylethyl)-3-methoxybenzene (50)

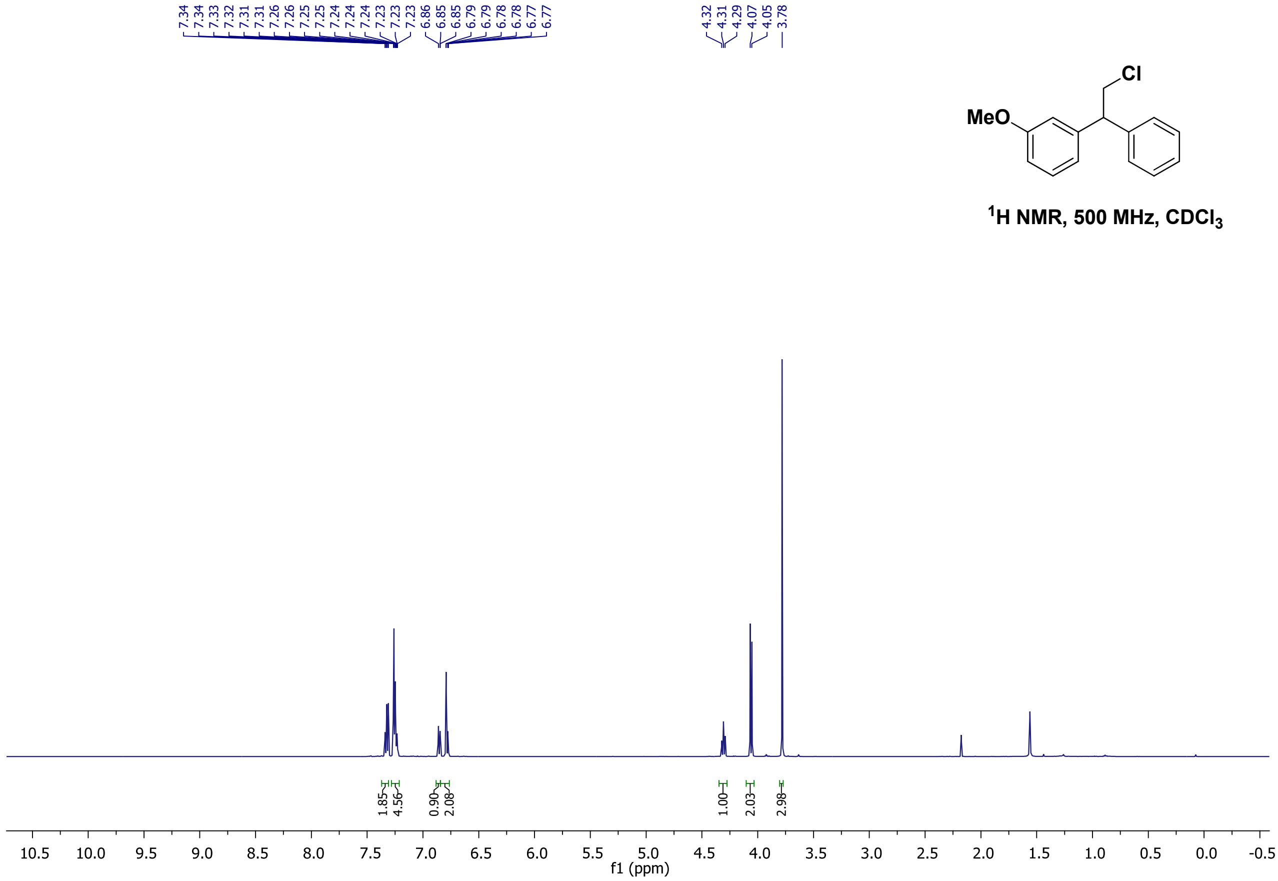

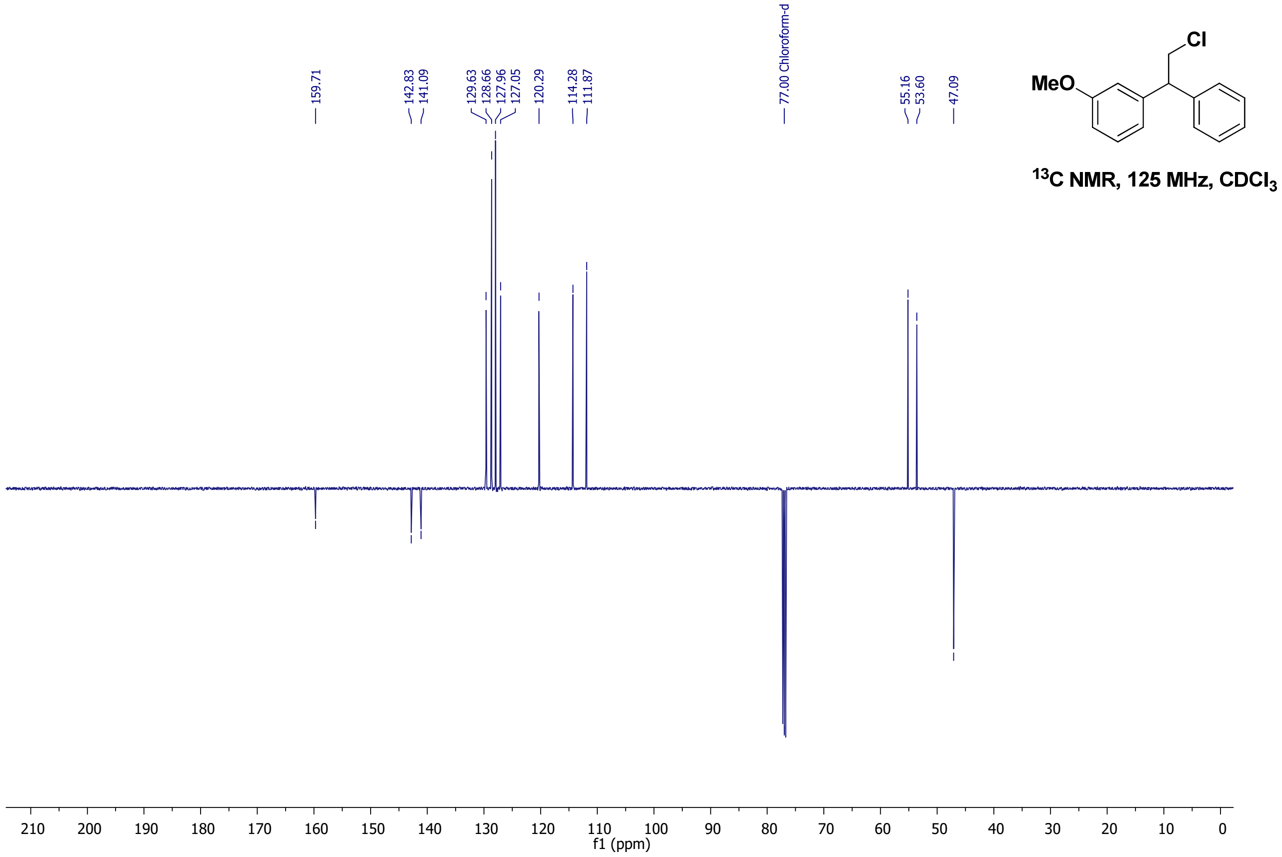

1-(2-Chloro-1-phenylethyl)-4-(methylsulfanyl)benzene (51)

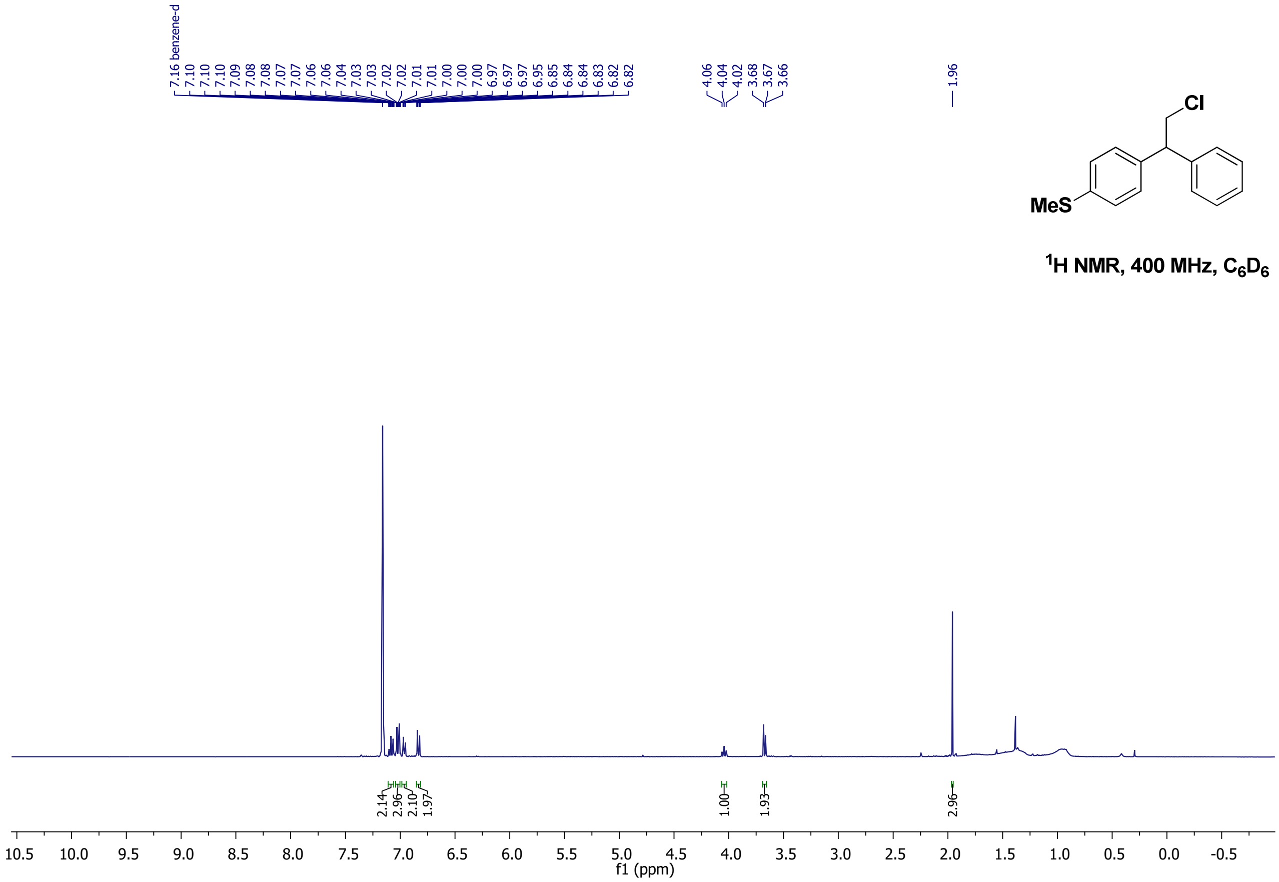

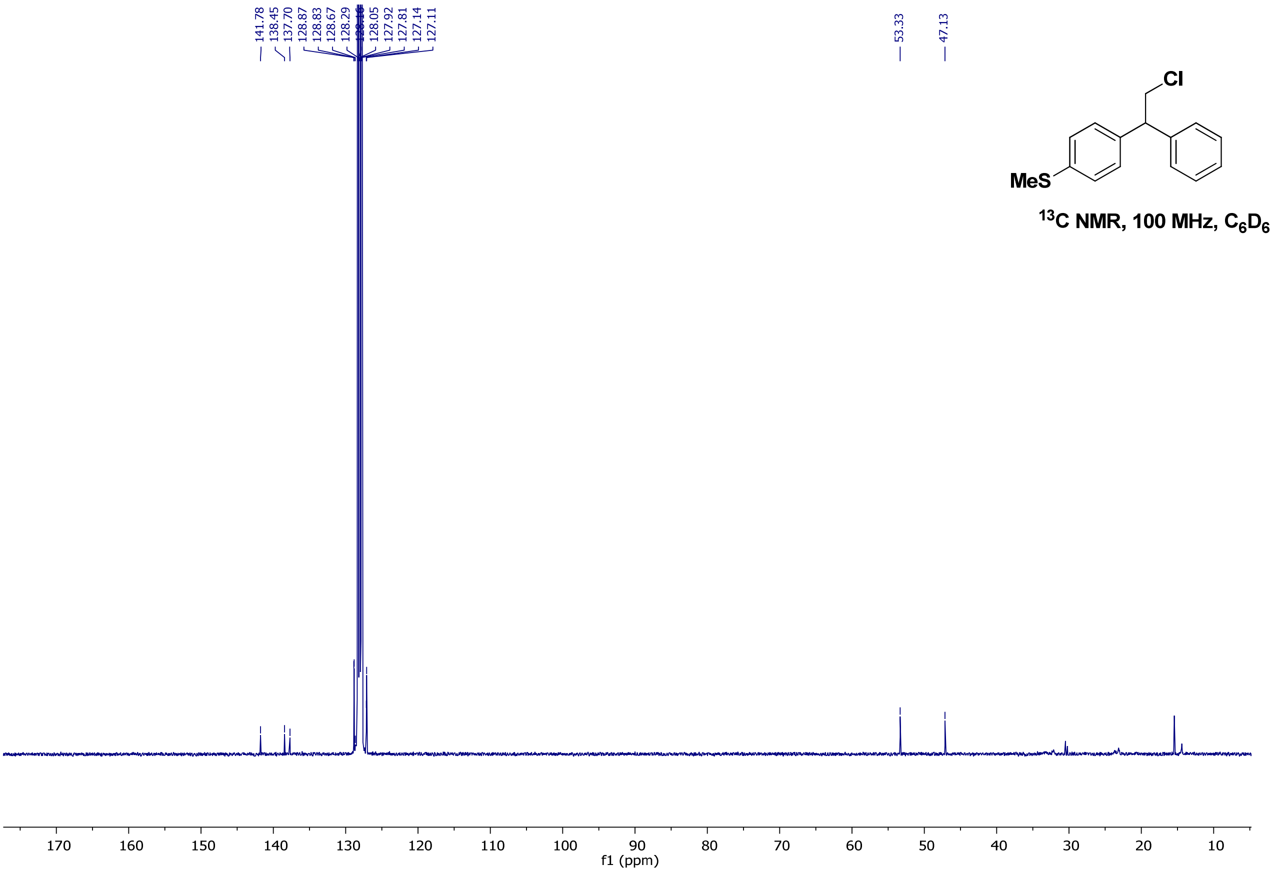

1-(2-Chloro-1-phenylethyl)-4-(phenylselanyl)benzene (52)

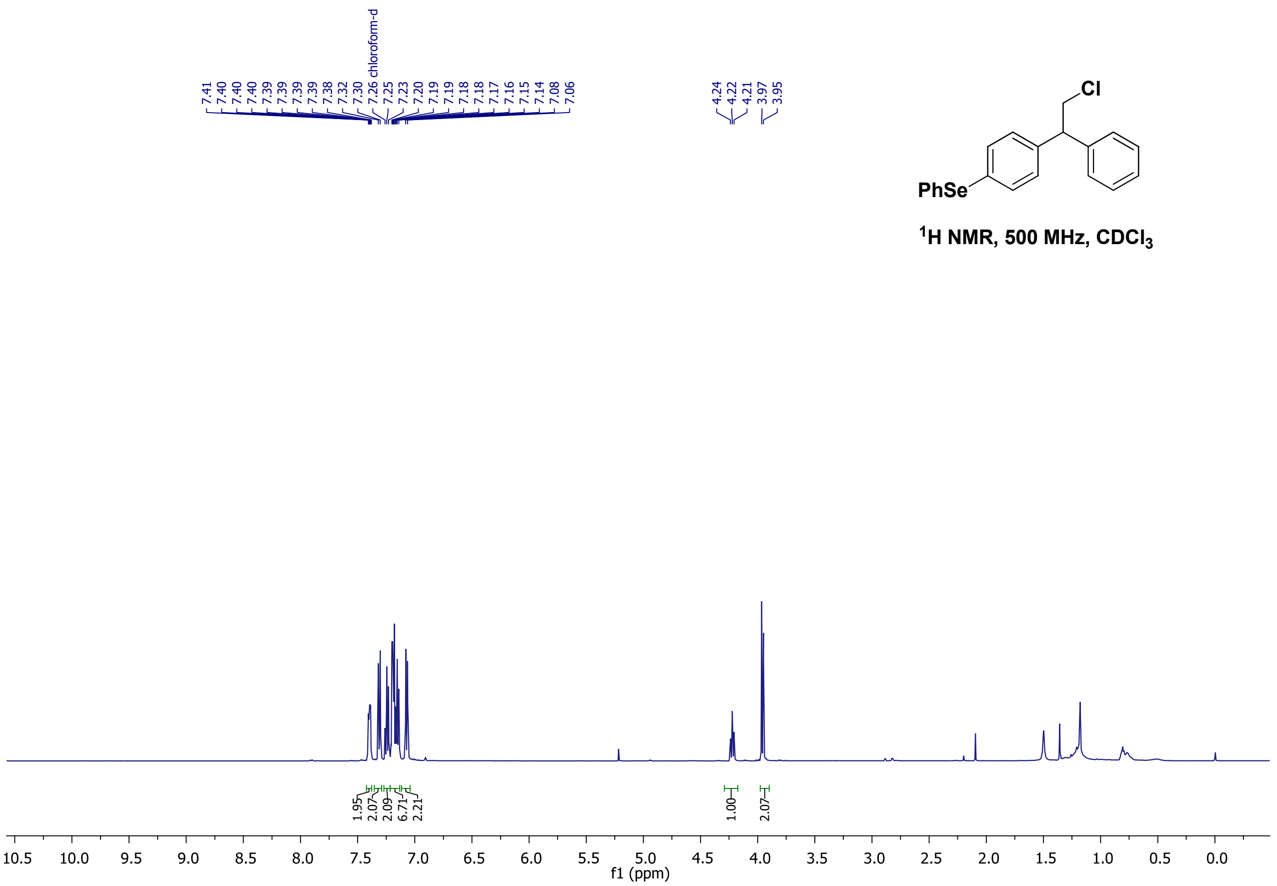

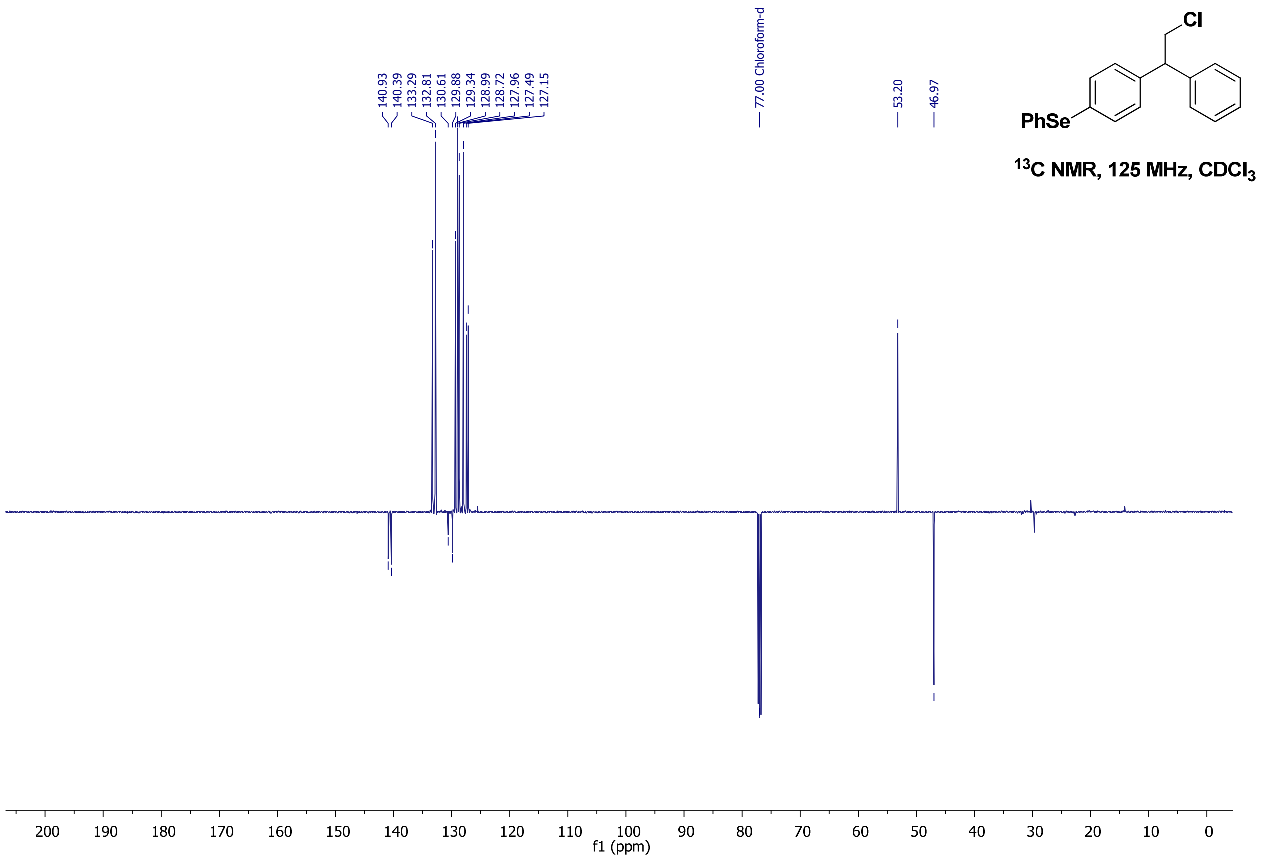

1-(2-Chloro-1-phenylethyl)-4-iodobenzene (53)

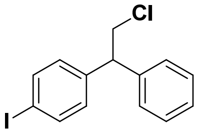

^{1}H NMR, 200 MHz, $CDCl_3$

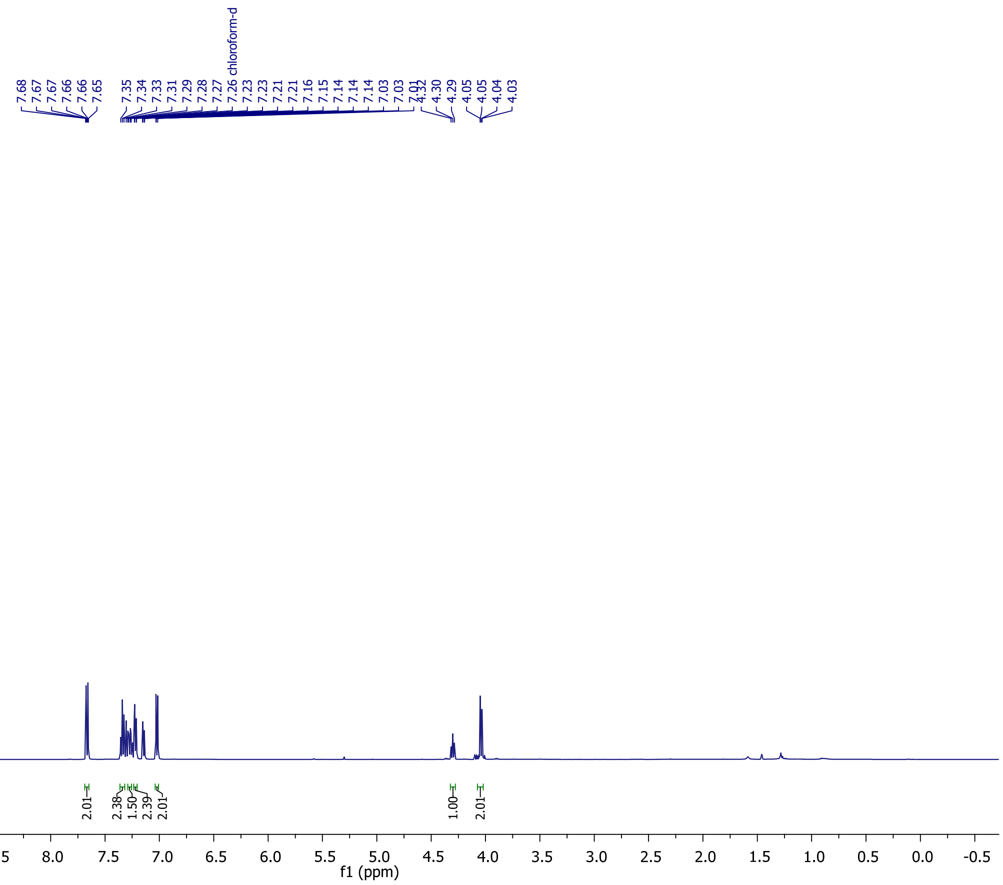

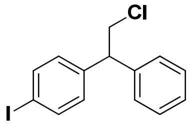

^{13}C NMR, 125 MHz, $CDCl_3$

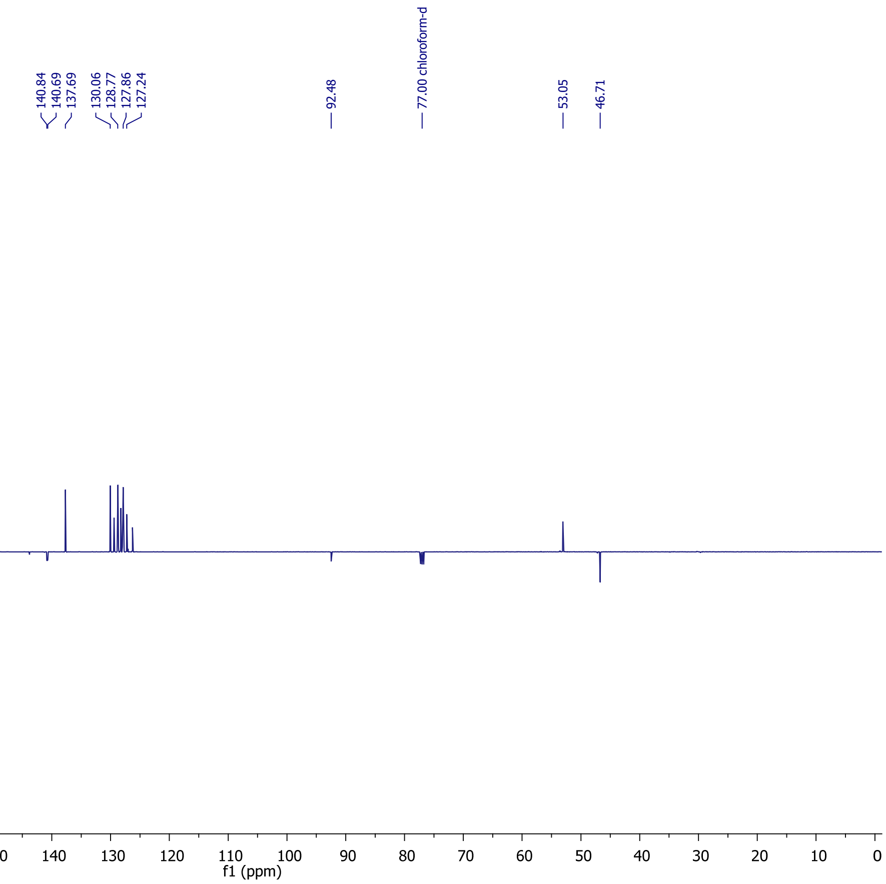

1-Bromo-3-(2-chloro-1-phenylethyl)benzene (54)

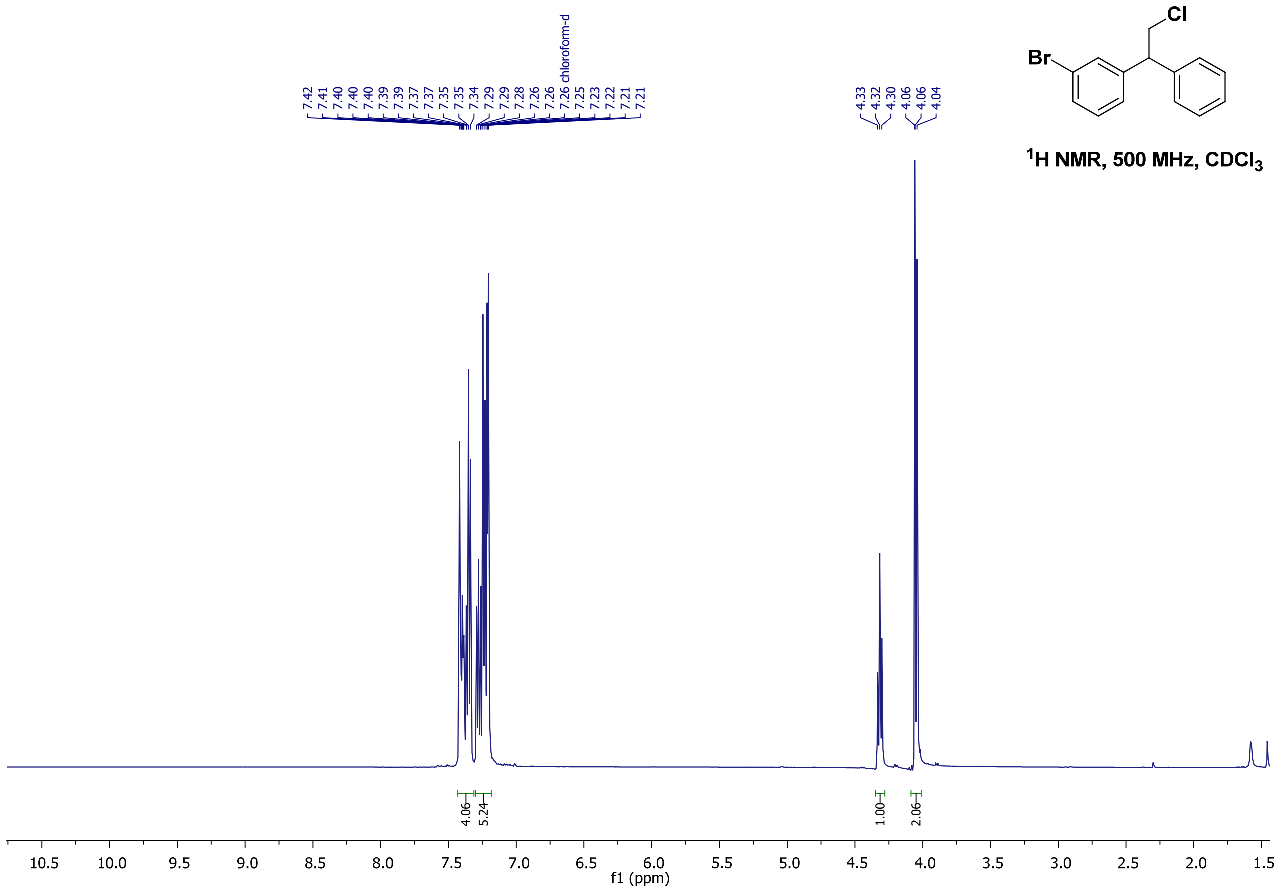

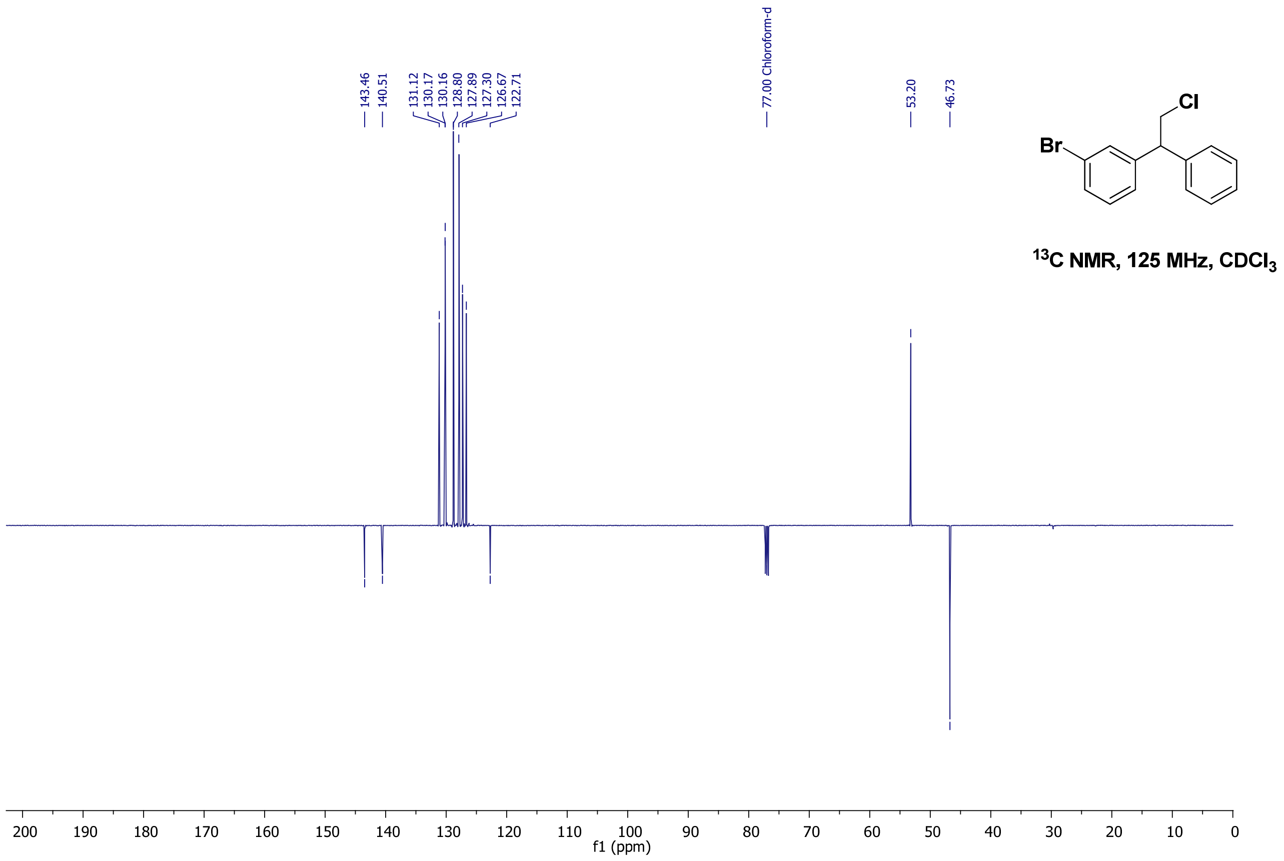

1-Chloro-3-(2-chloro-1-phenylethyl)benzene (55)

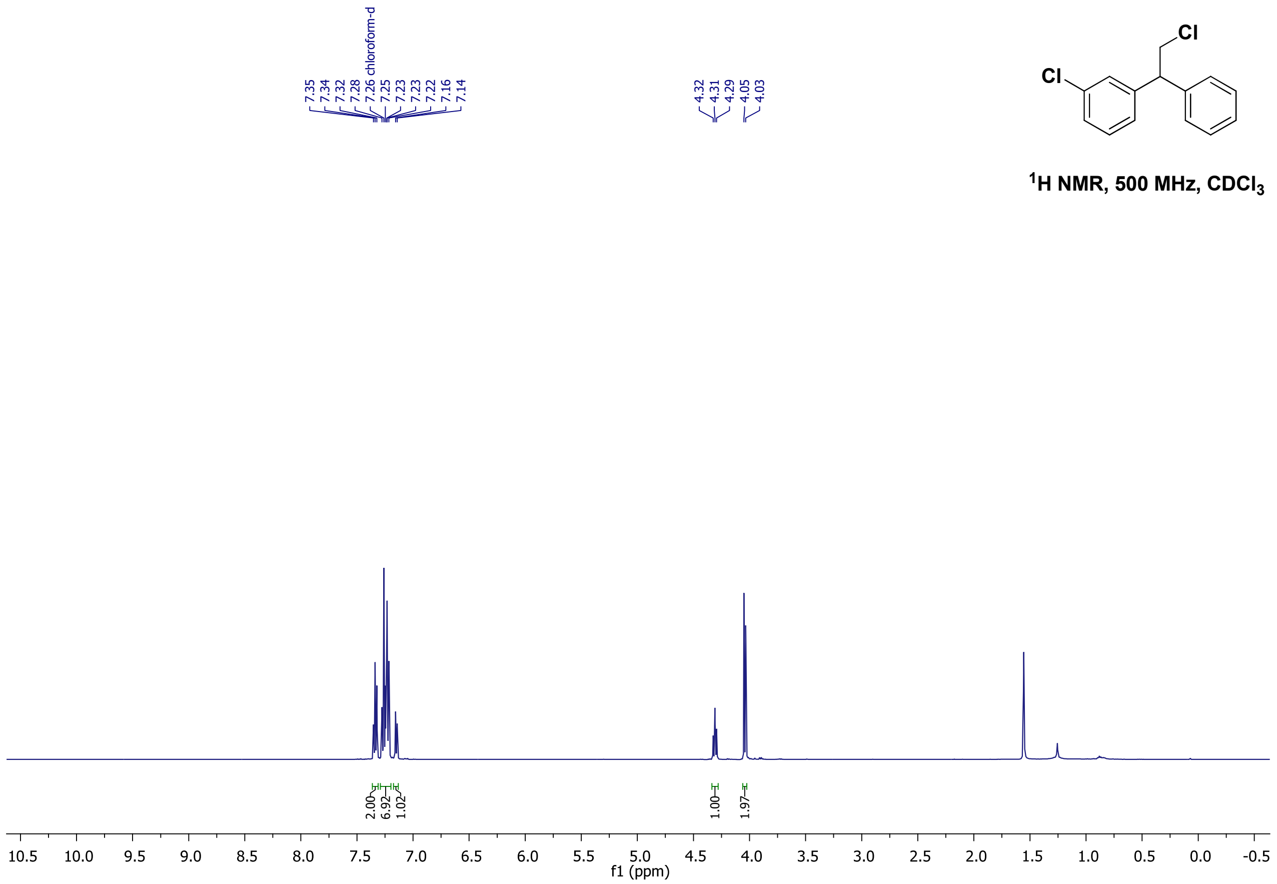

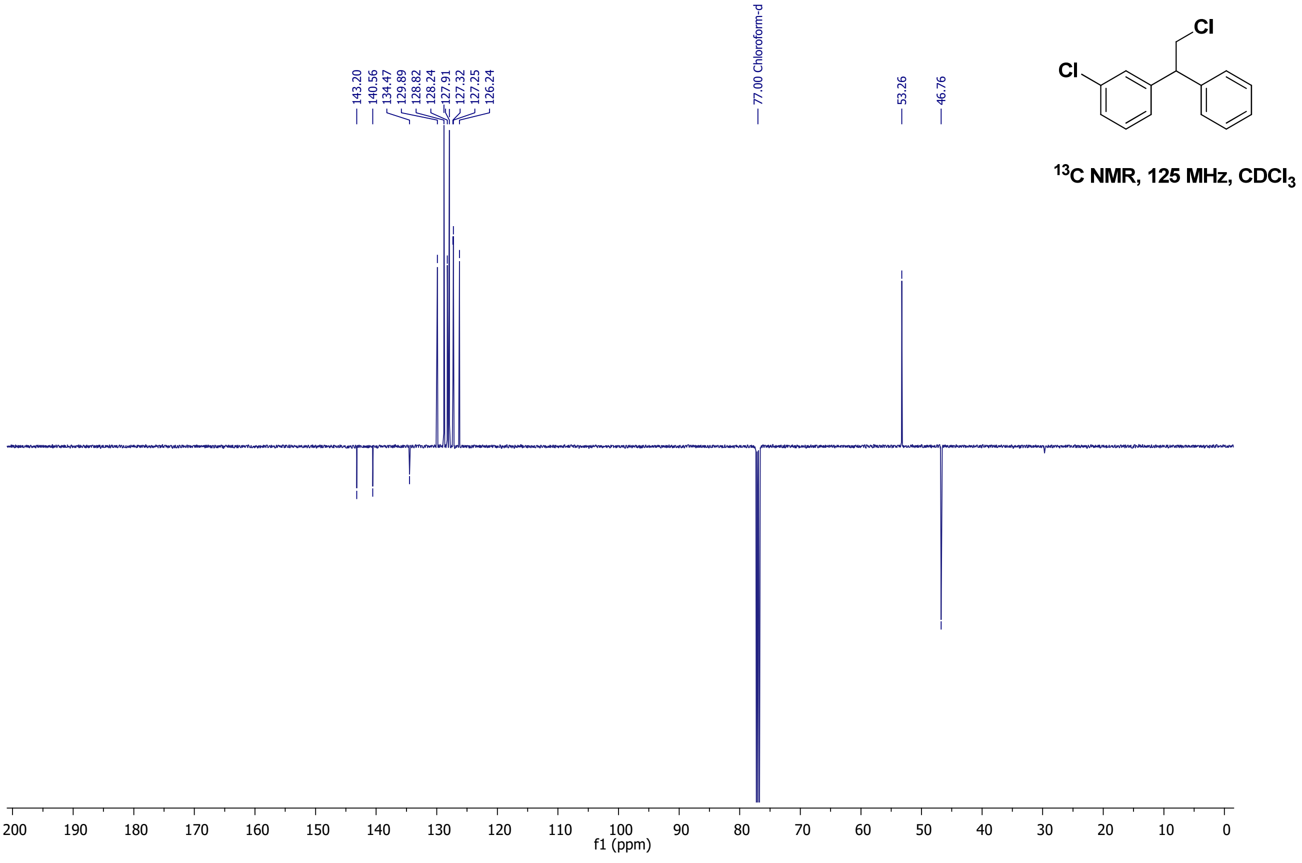

1-(2-Chloro-1-phenylethyl)-4-fluorobenzene (56)

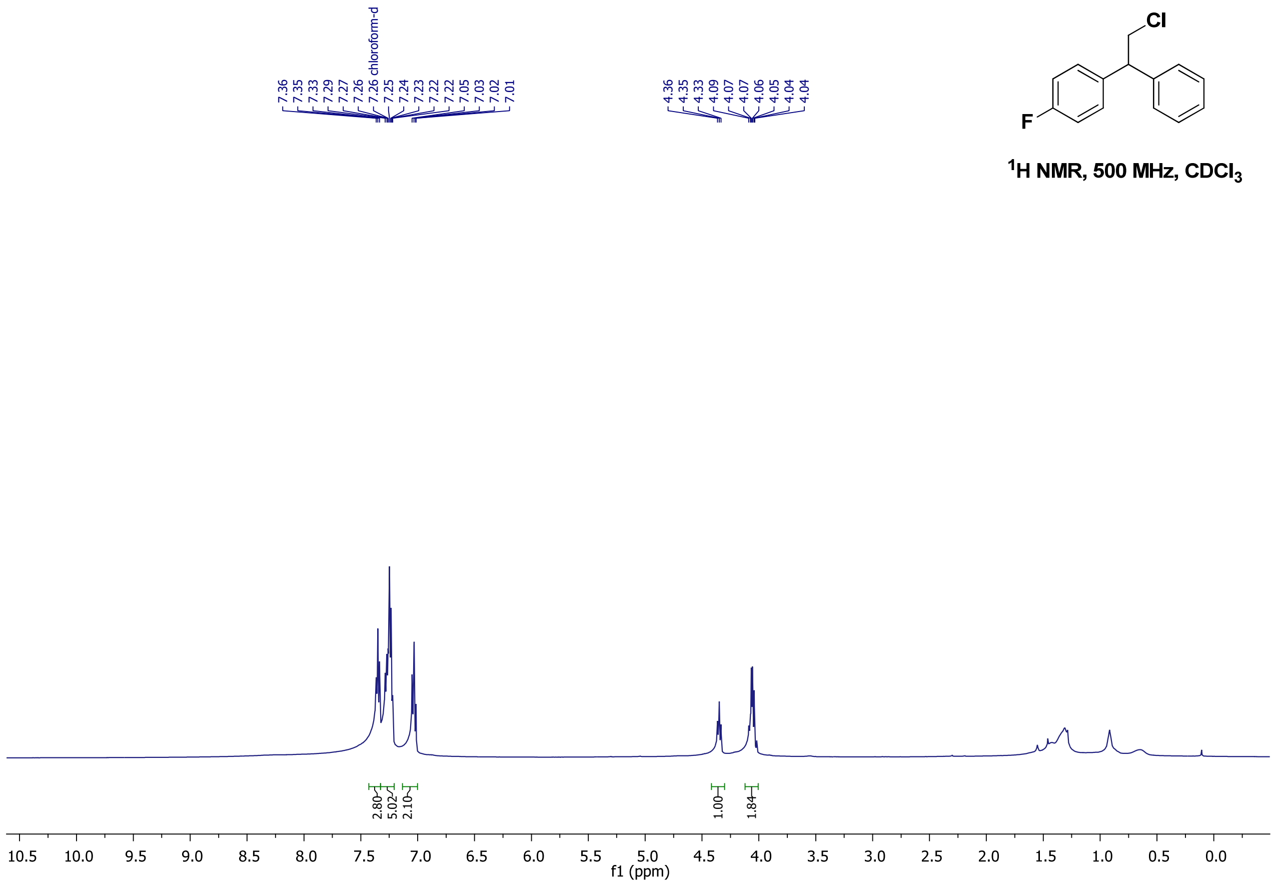

^{1}H NMR, 500 MHz, $CDCl_3$

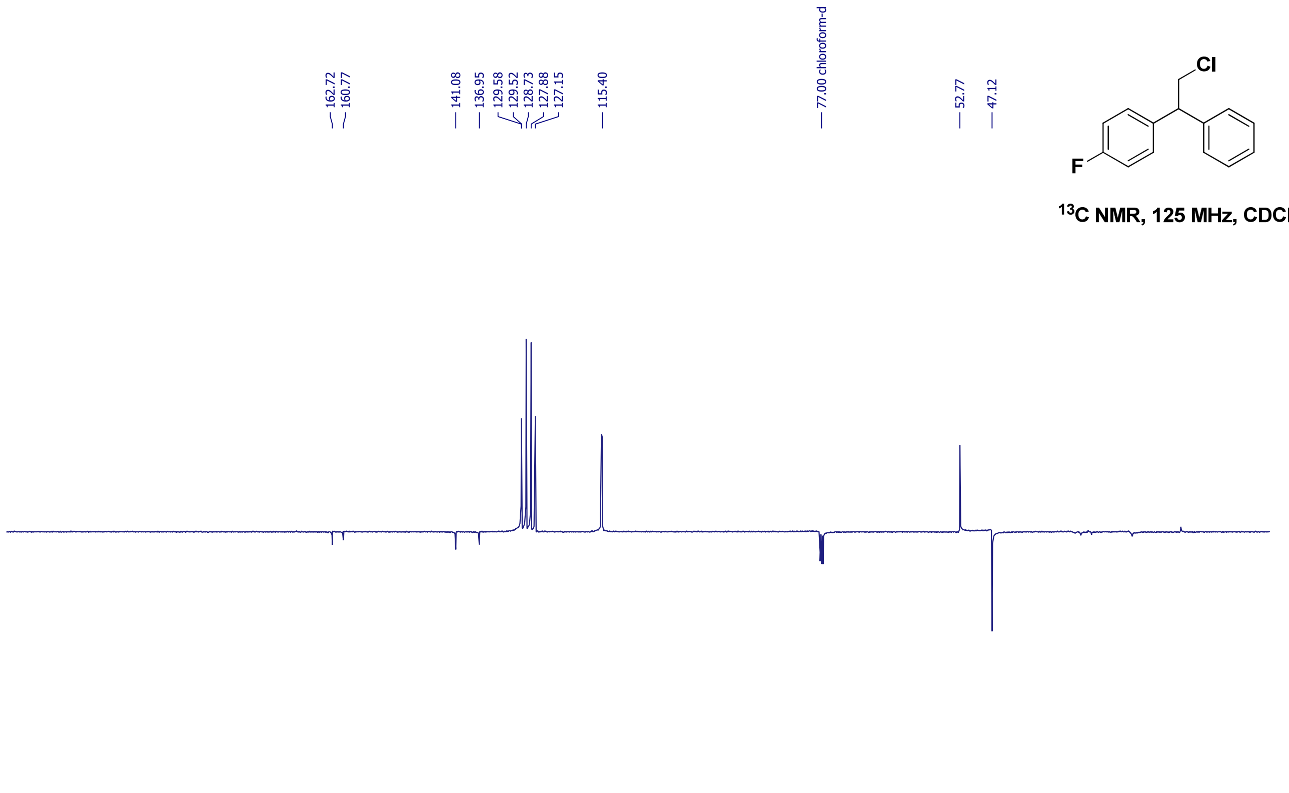

^{13}C NMR, 125 MHz, $CDCl_3$

1-(2-Chloro-1-phenylethyl)-2-fluorobenzene (57)

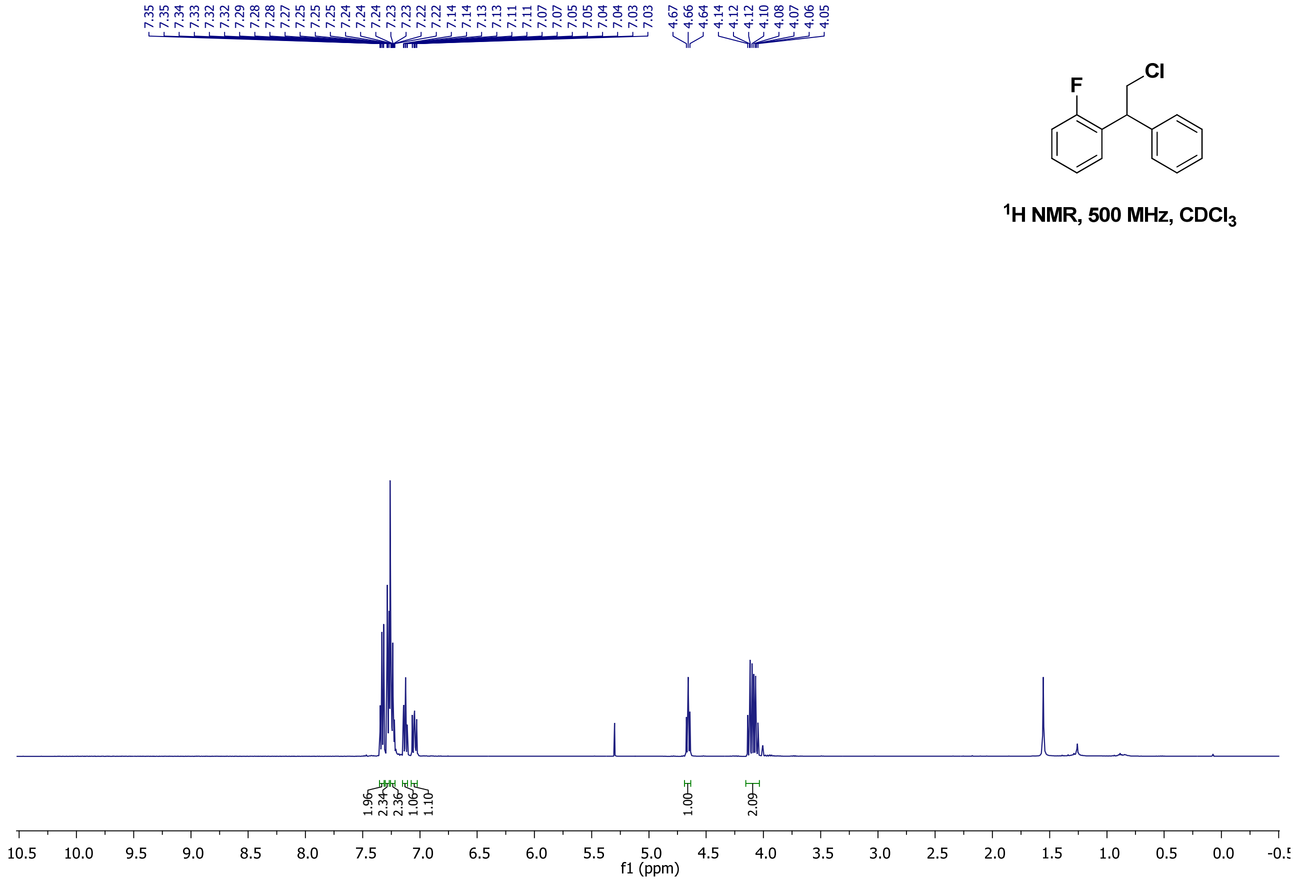

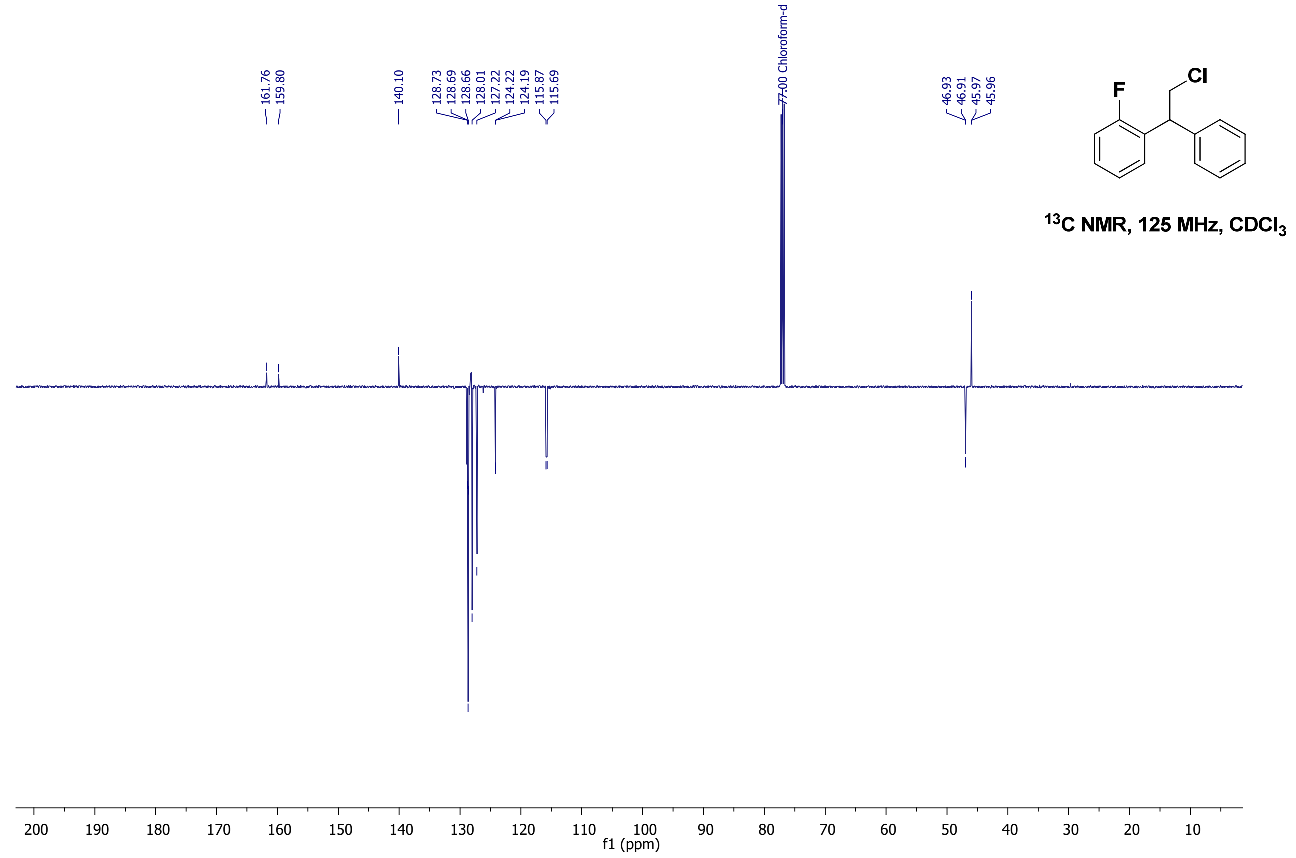

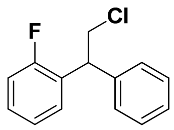

^{19}F NMR, 376 MHz, CDCl$_3$

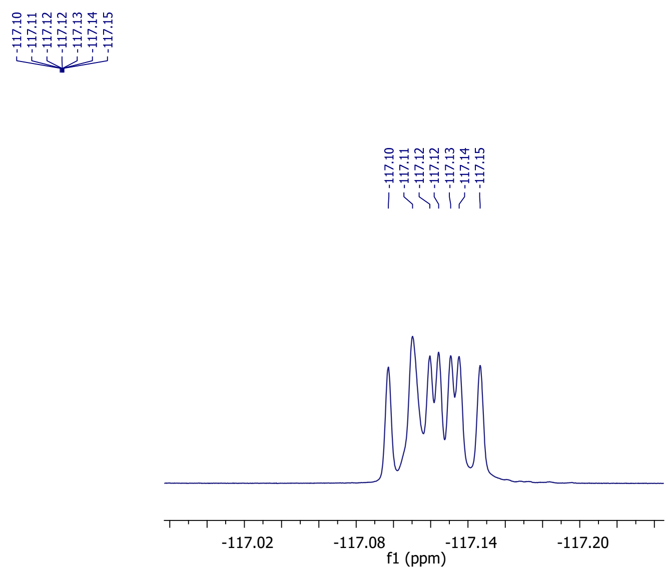

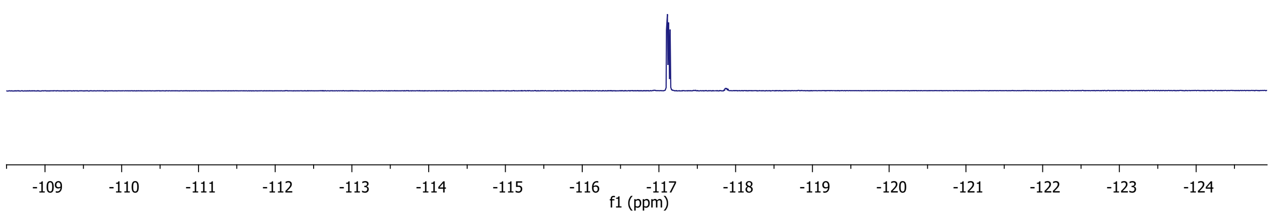

1-(2-Chloro-1-phenylethyl)-3,5-bis(trifluoromethyl)benzene (58)

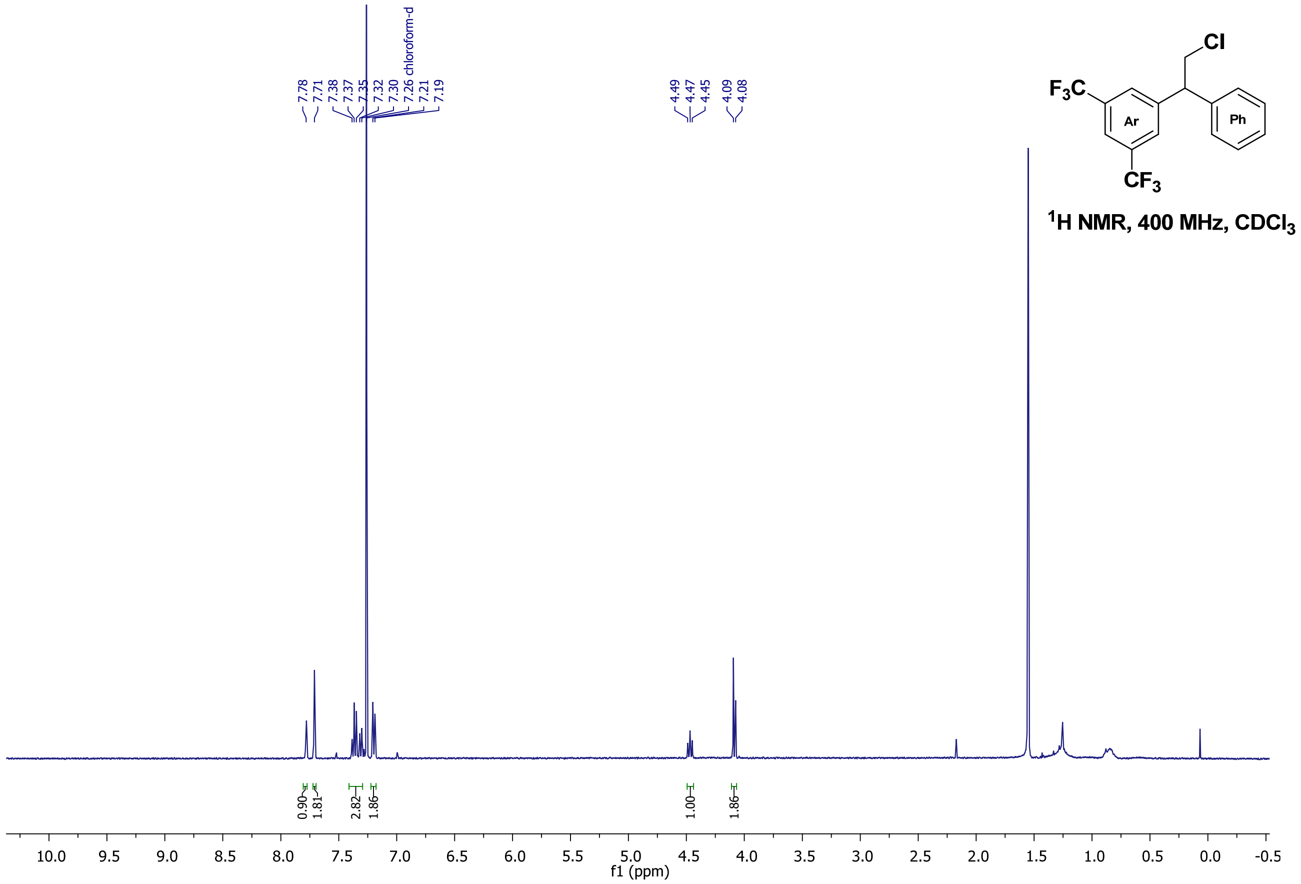

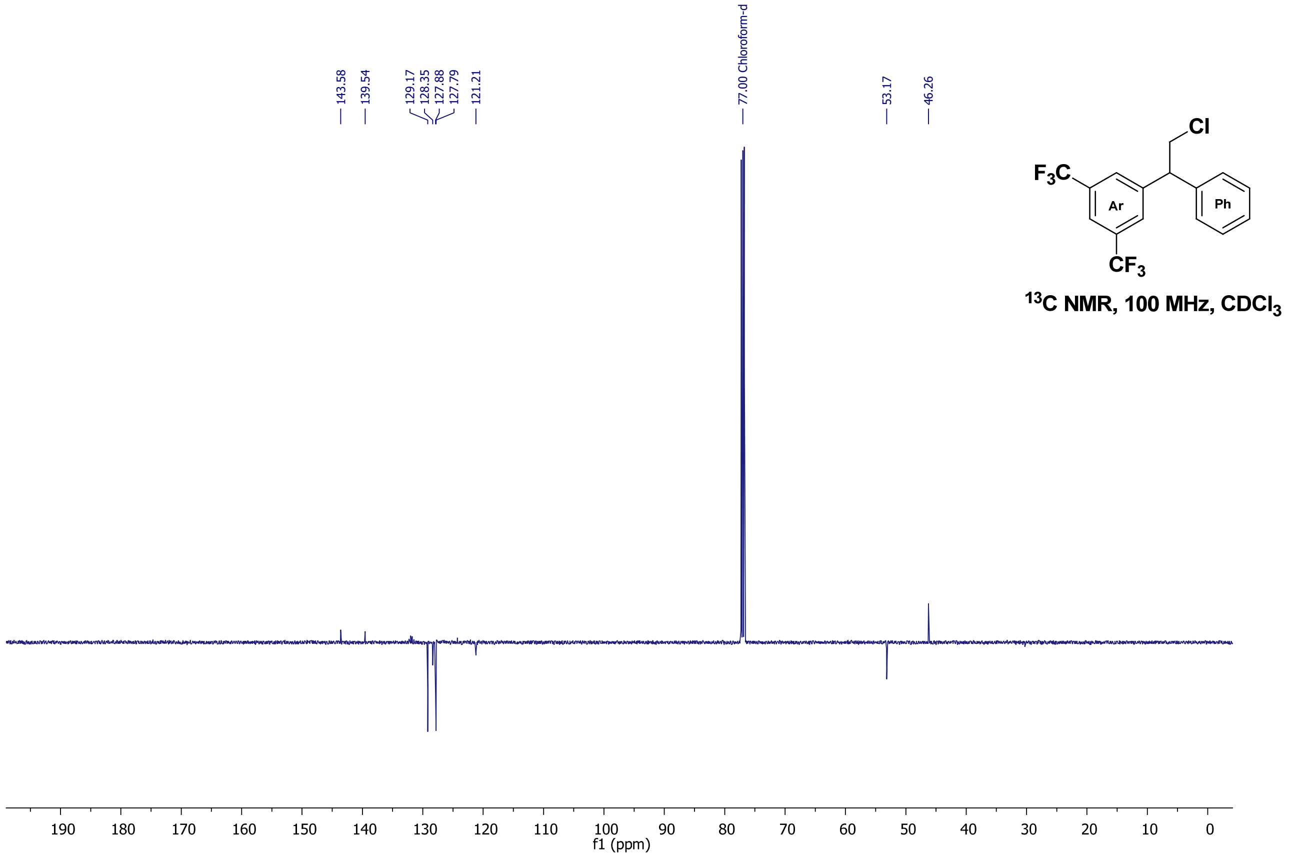

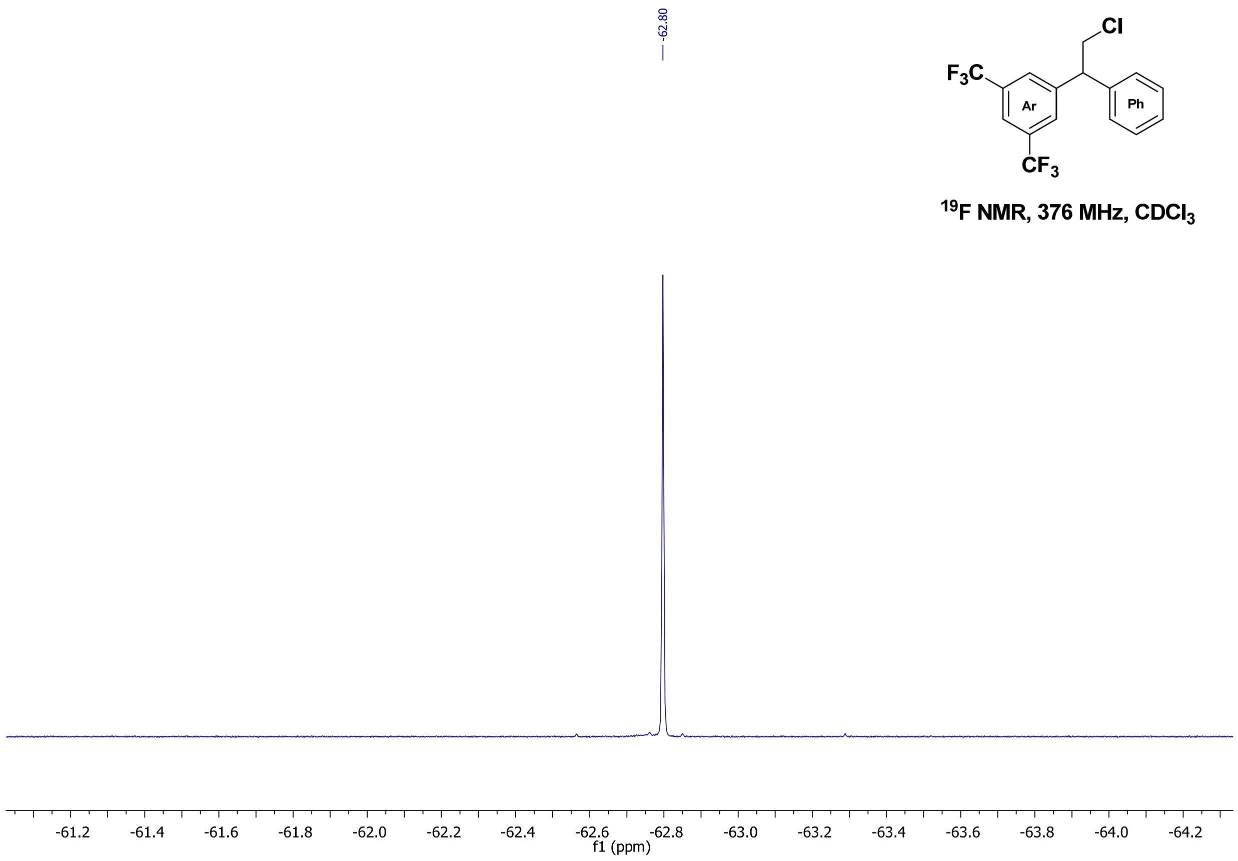
-62.80
Cl
F3C
Ar
Ph
CF3
19F NMR, 376 MHz, CDCl3
-61.2
-61.4
-61.6
-61.8
-62.0
-62.2
-62.4
-62.6
-62.8
-63.0
-63.2
-63.4
-63.6
-63.8
-64.0
-64.2
f1 (ppm)

1-Azido-4-(2-chloro-1-phenylethyl)benzene (59)

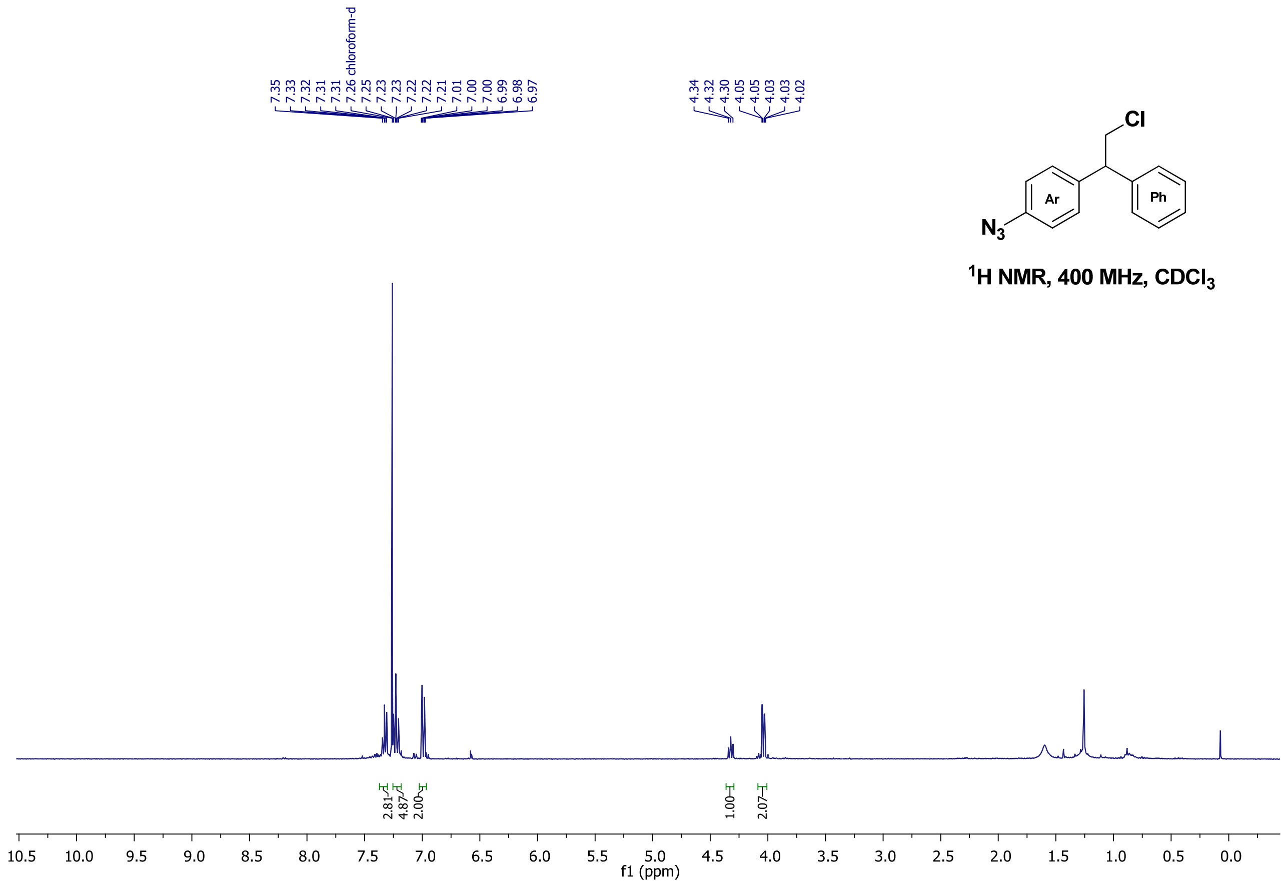

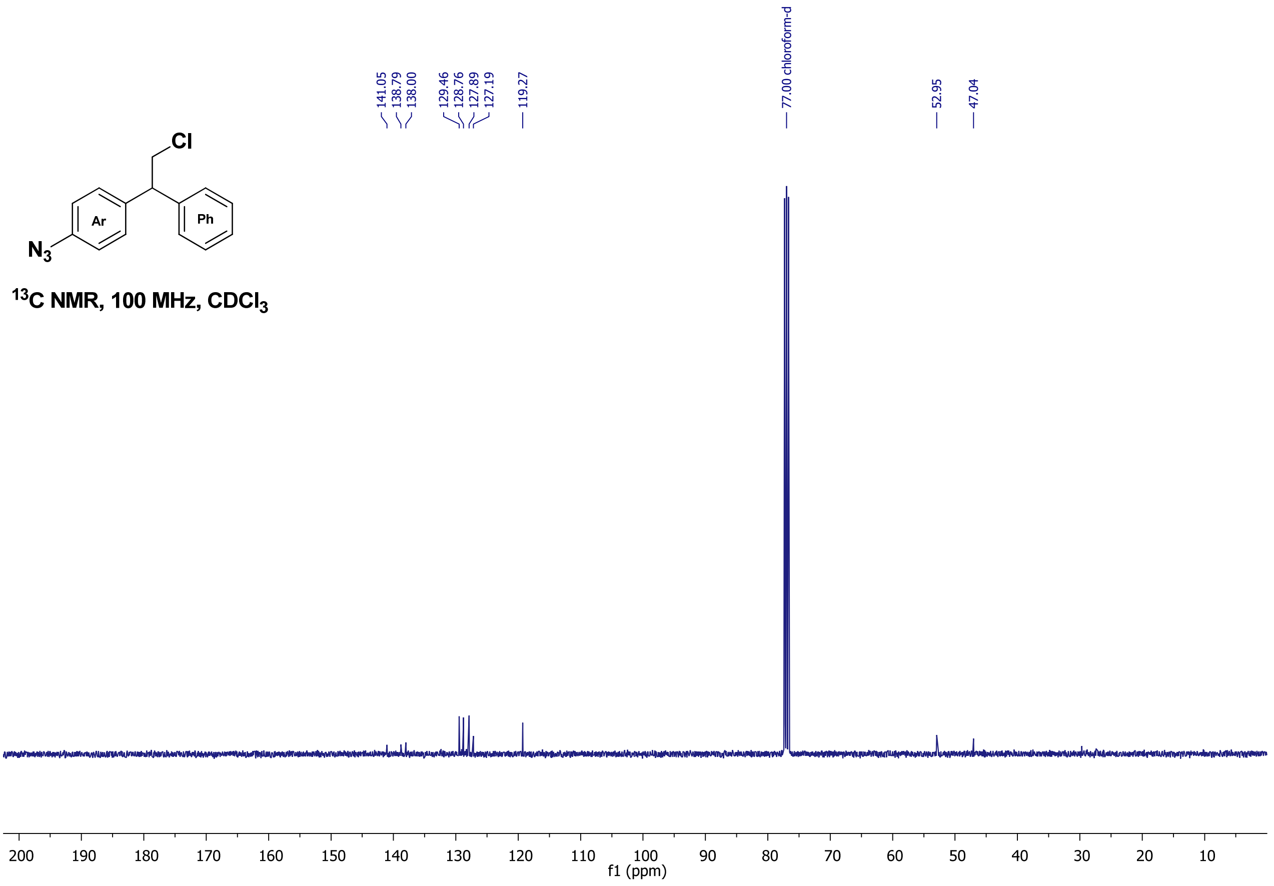

1,1'-(2-Chloro-1,1- ethanediyl)bis(4-methoxybenzene) (60)

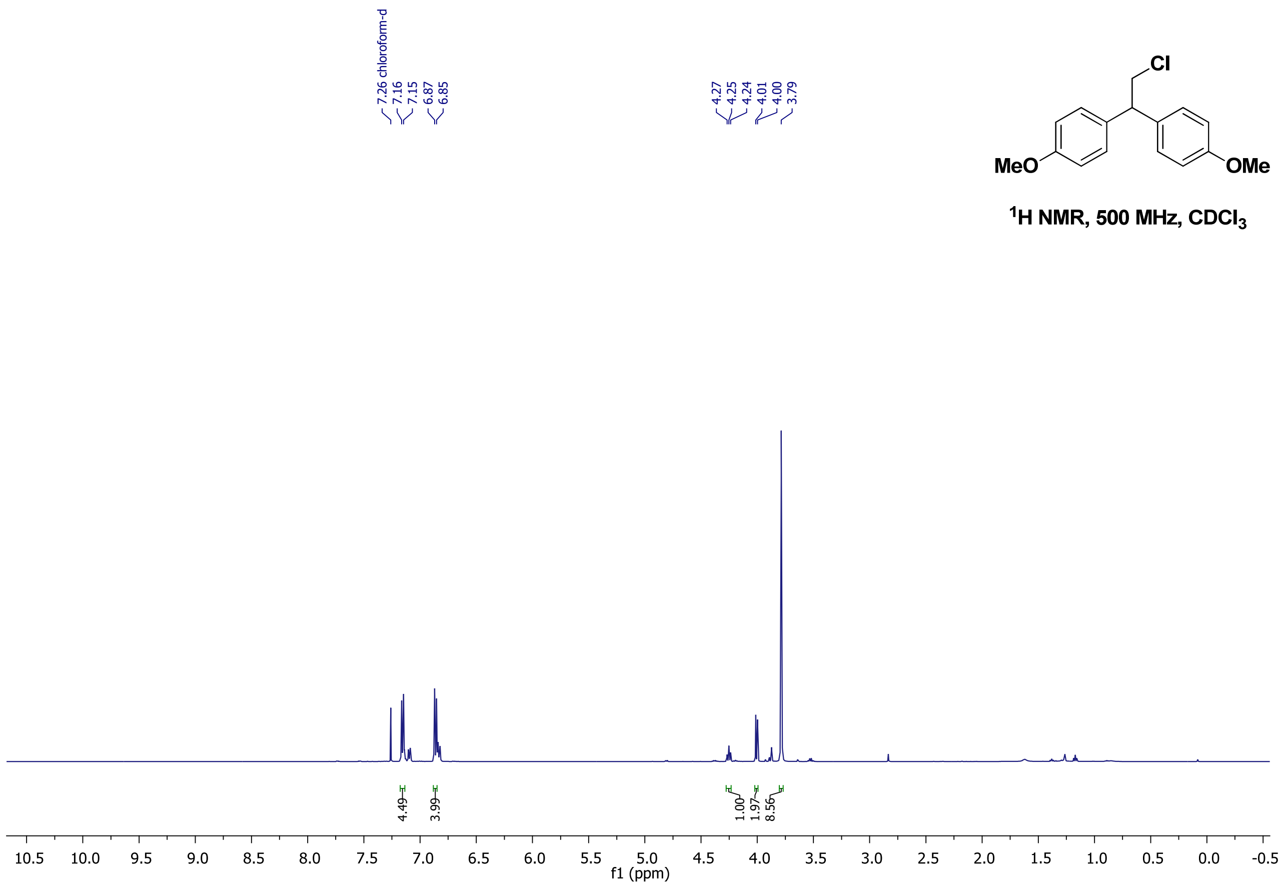

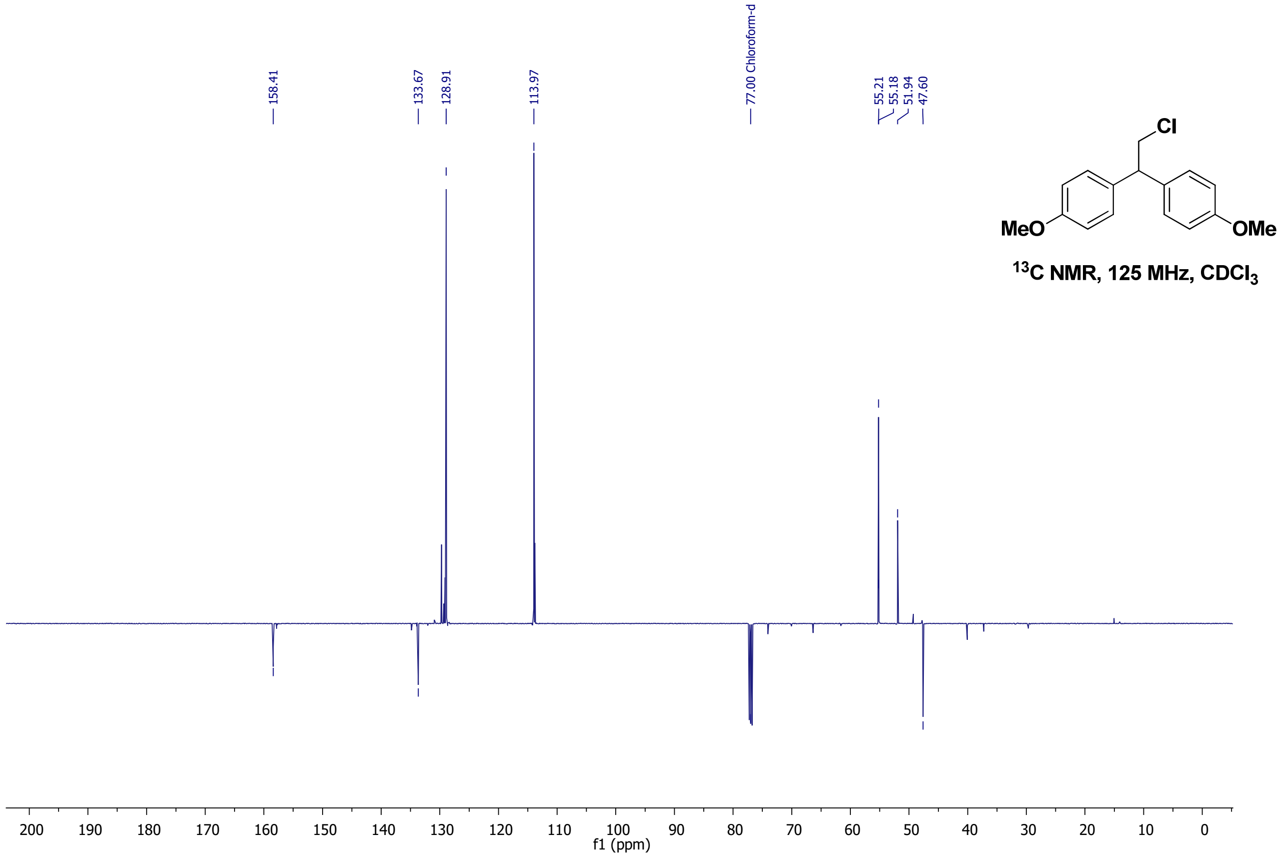

1,1'-(2-Chloro-1,1-ethanediyl)bis(4-chlorobenzene) (61)

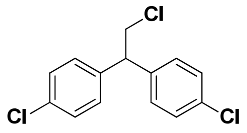

^1H NMR, 500 MHz, C_6D_6

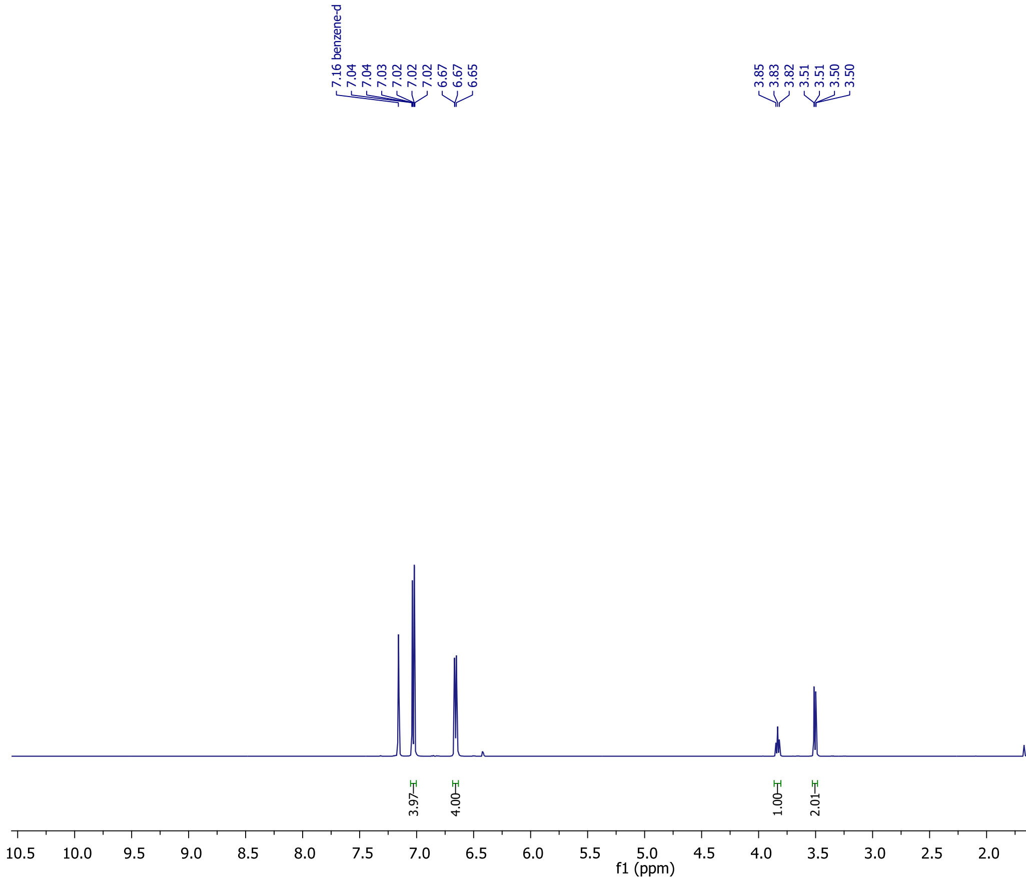

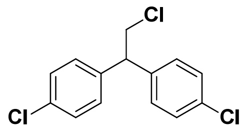

^{13}C NMR, 125 MHz, C_6D_6

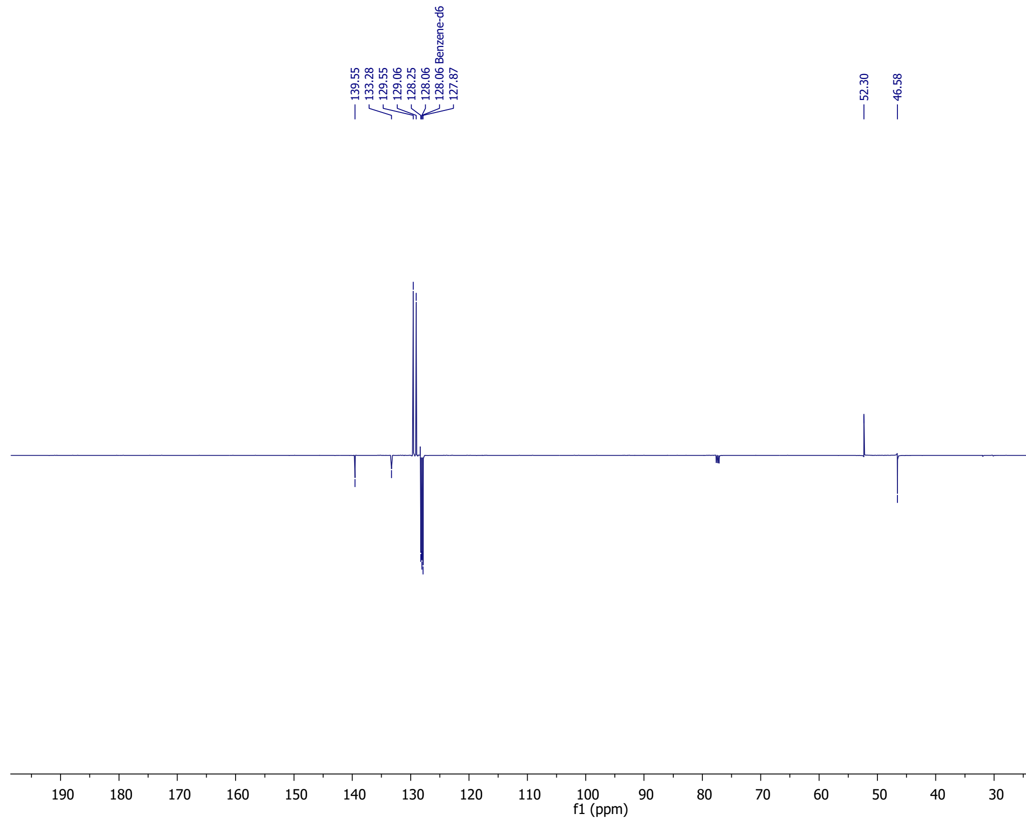

1-(2-Chloro-1-phenylethyl)naphthalene (62)

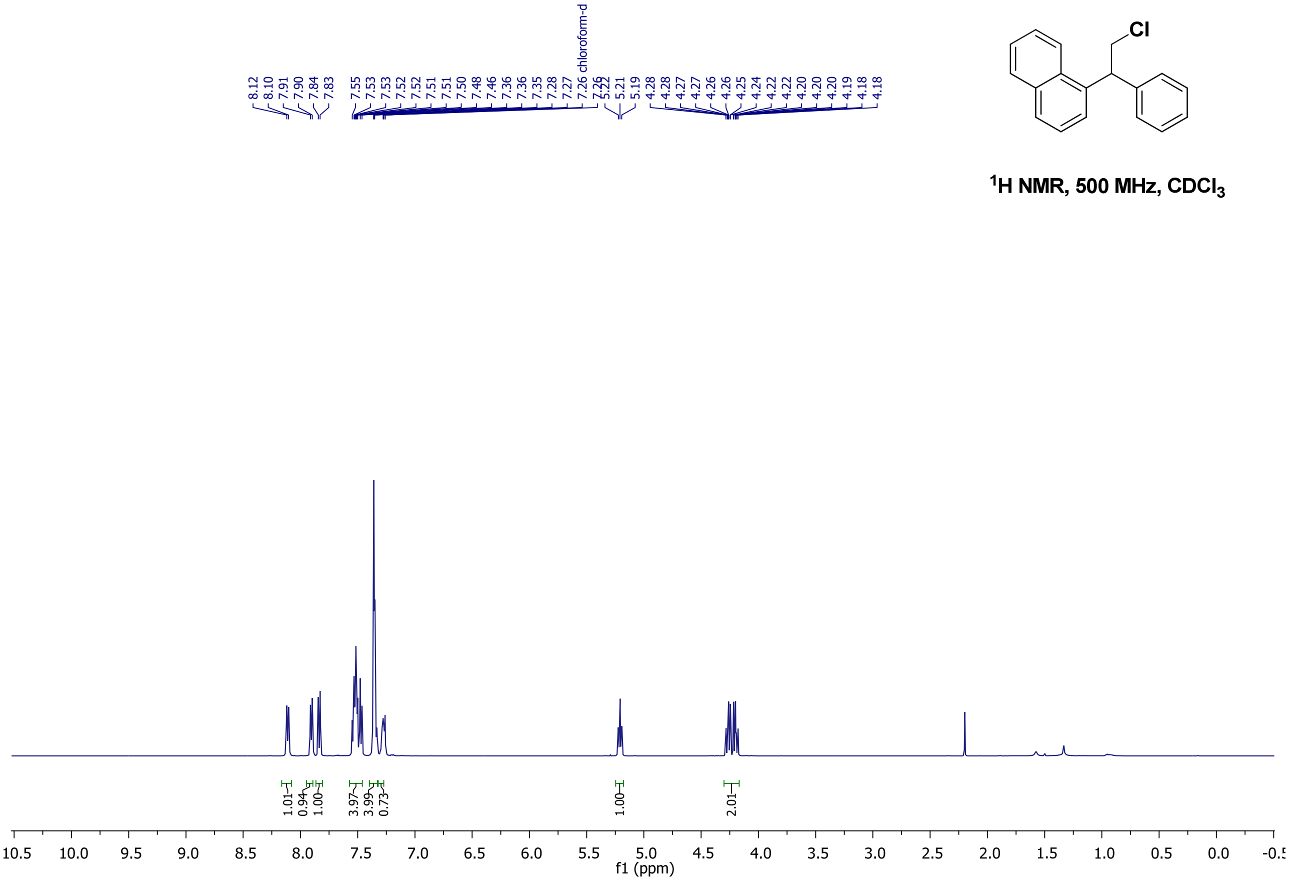

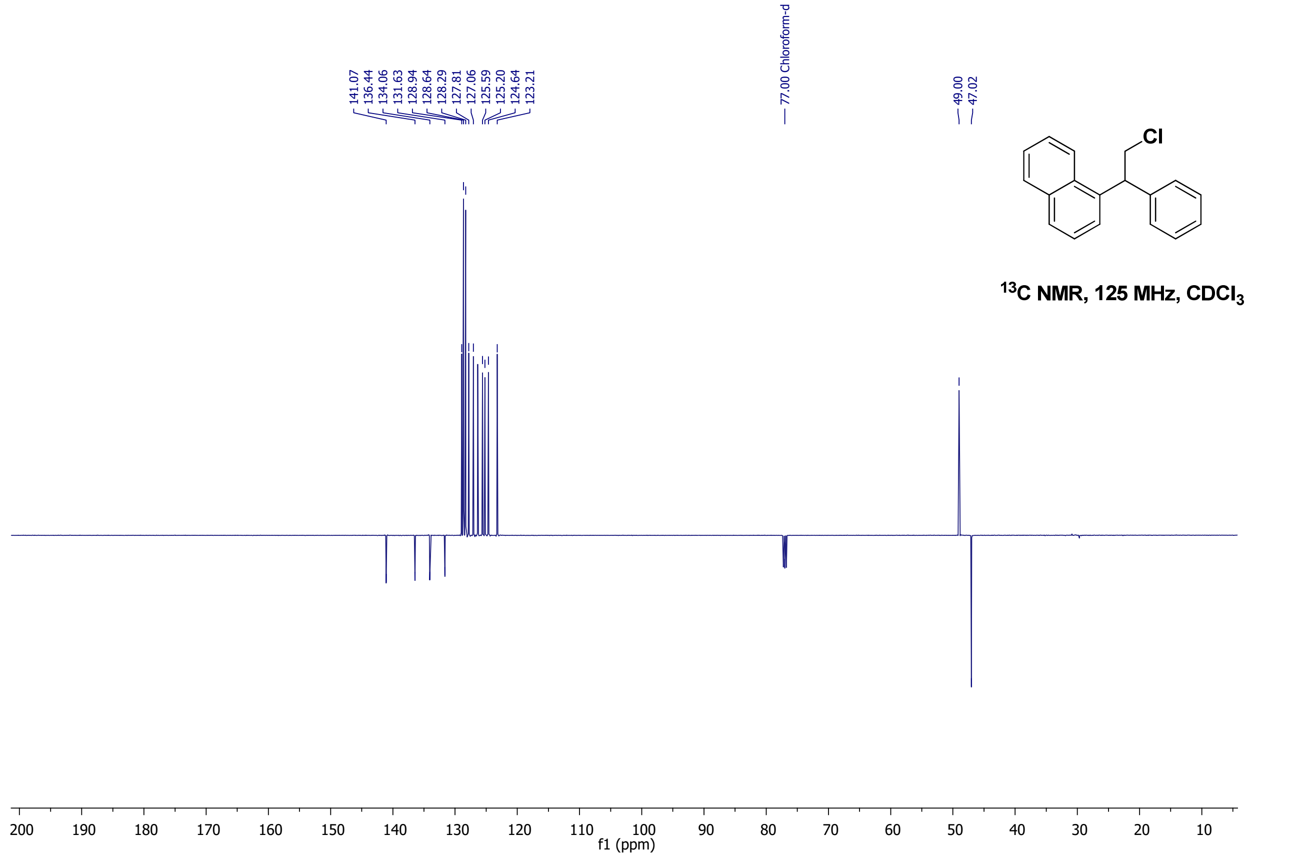

2-[2-Chloro-1-(4-fluorophenyl)ethyl]-6-methoxynaphthalene (63)

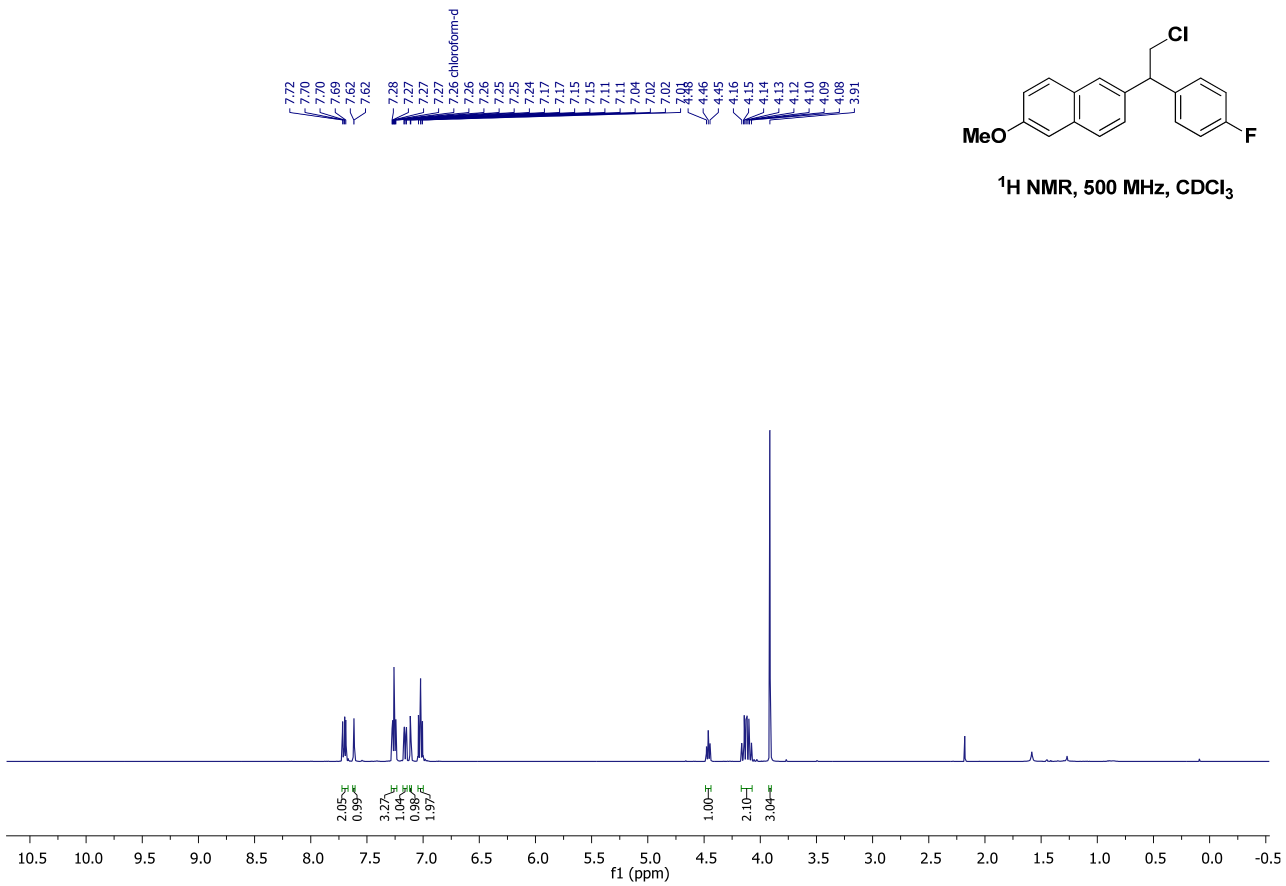

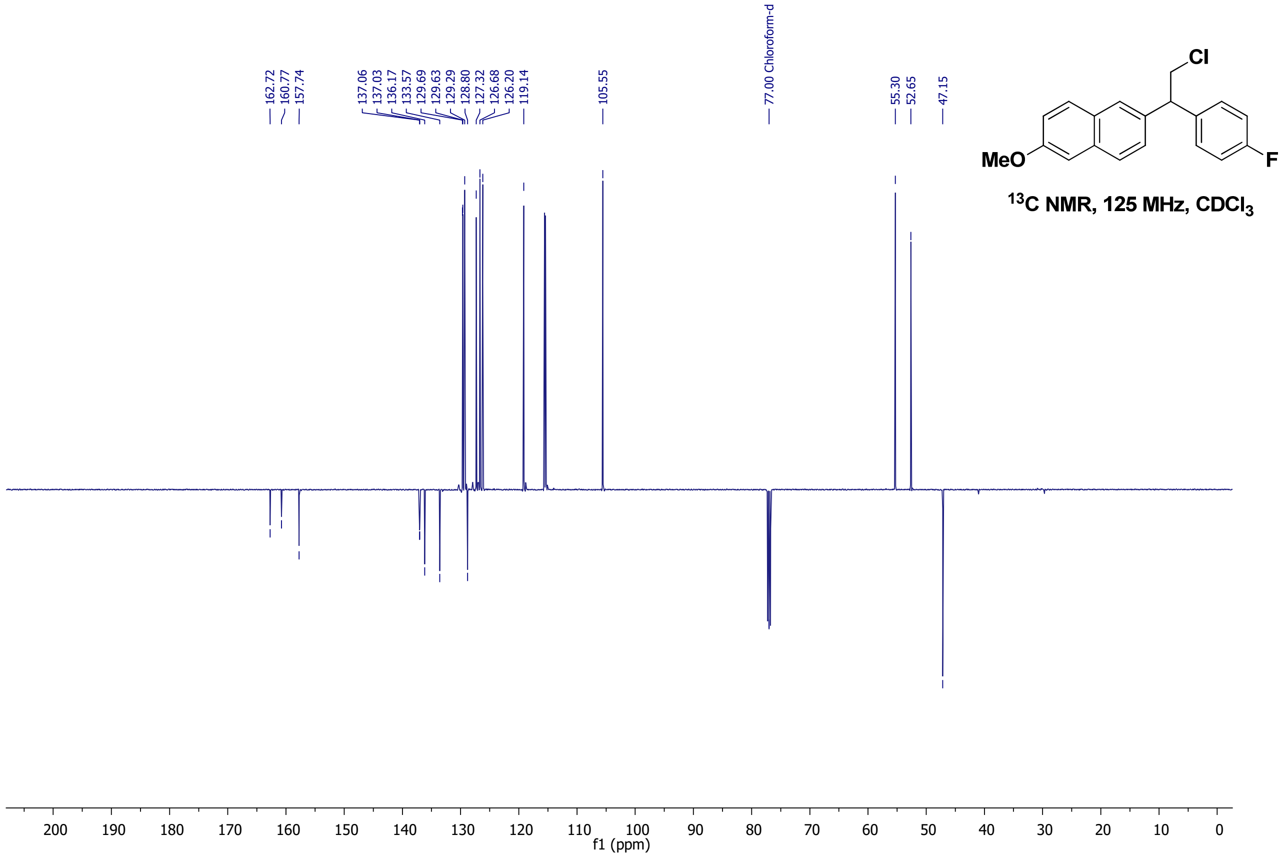

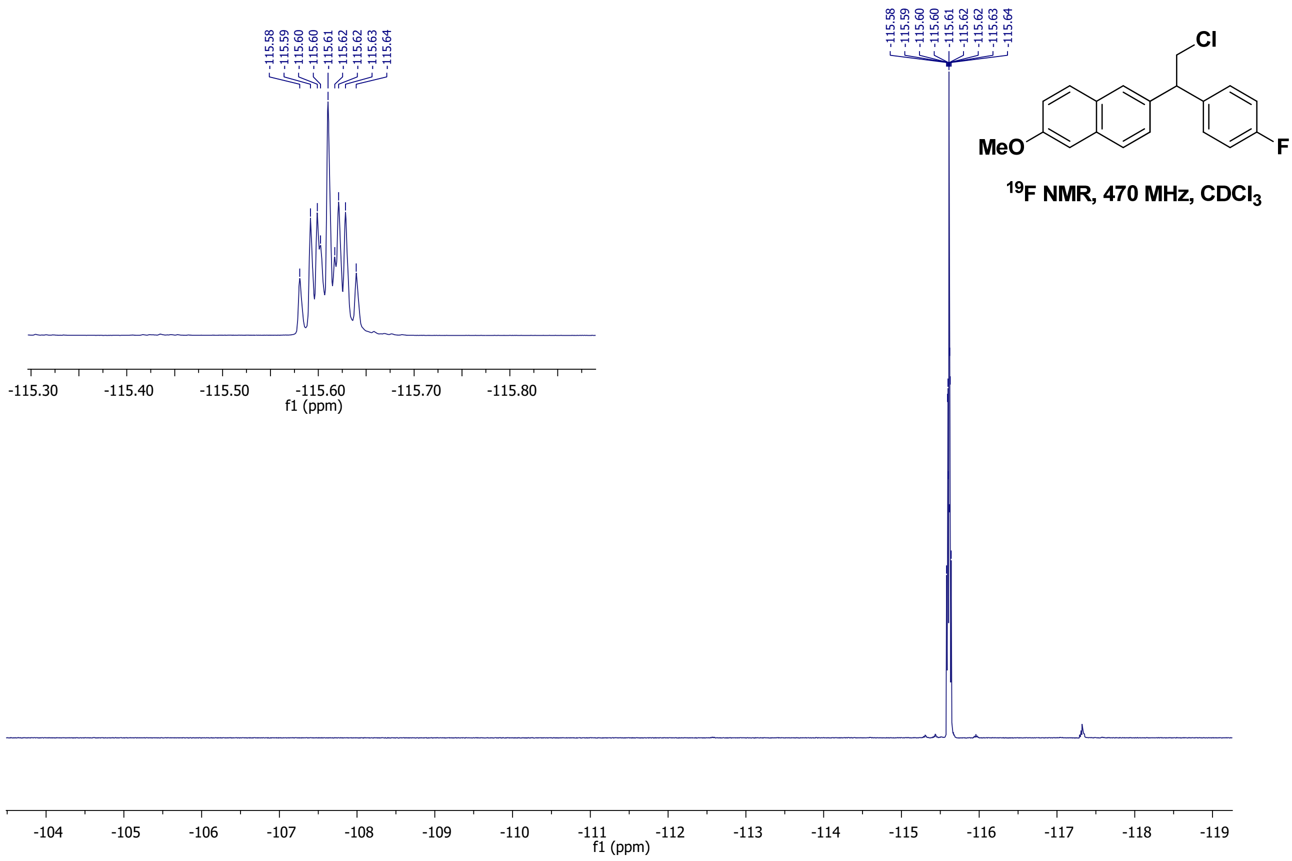

-115.58
-115.59
-115.60
-115.60
-115.61
-115.62
-115.62
-115.63
-115.64
Cl
MeO
F
^{19}F NMR, 470 MHz, CDCl$_3$
-115.30
-115.40
-115.50
-115.60
-115.70
-115.80
f1 (ppm)
-104
-105
-106
-107
-108
-109
-110
-111
-112
-113
-114
-115
-116
-117
-118
-119
f1 (ppm)

5-(2-Chloro-1-phenylethyl)-1,3-benzodioxole (64)

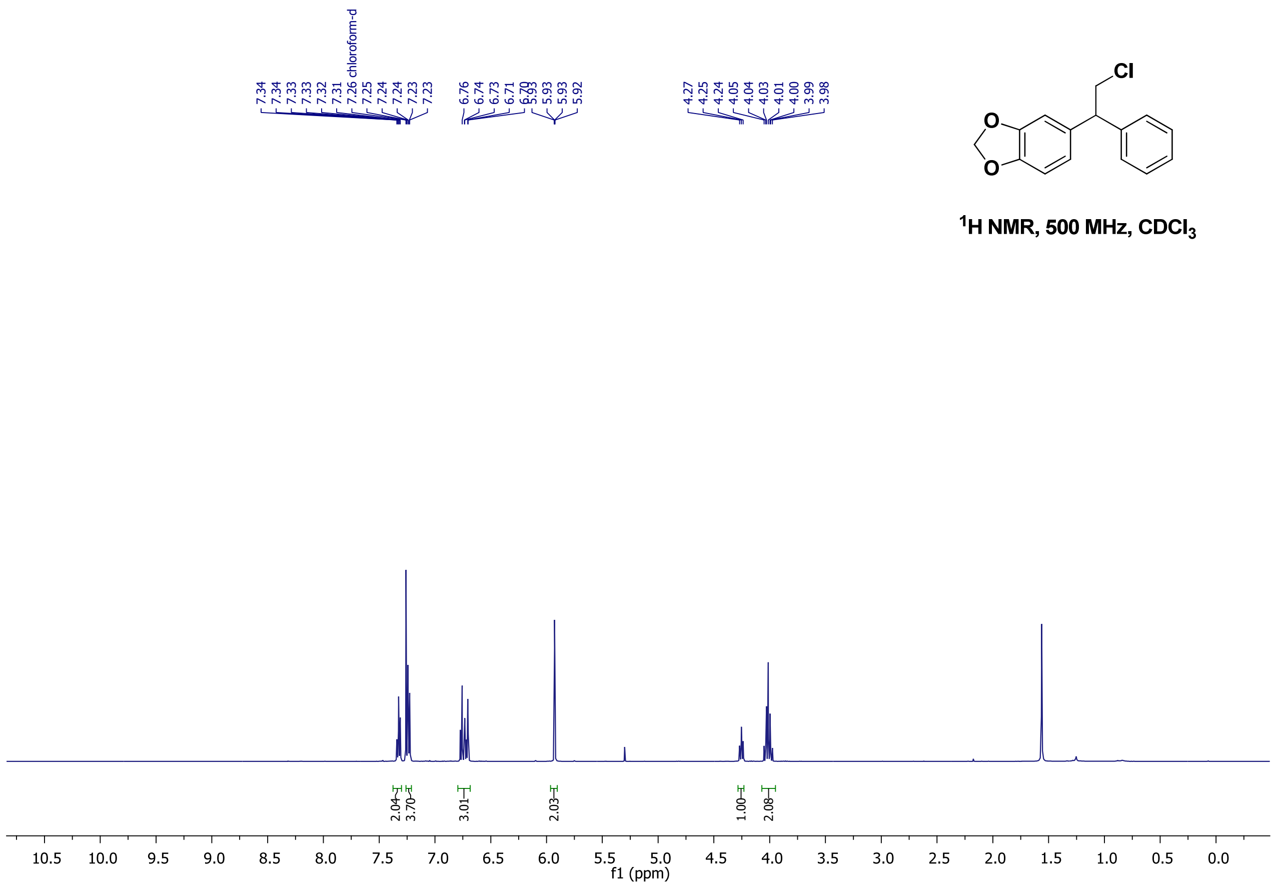

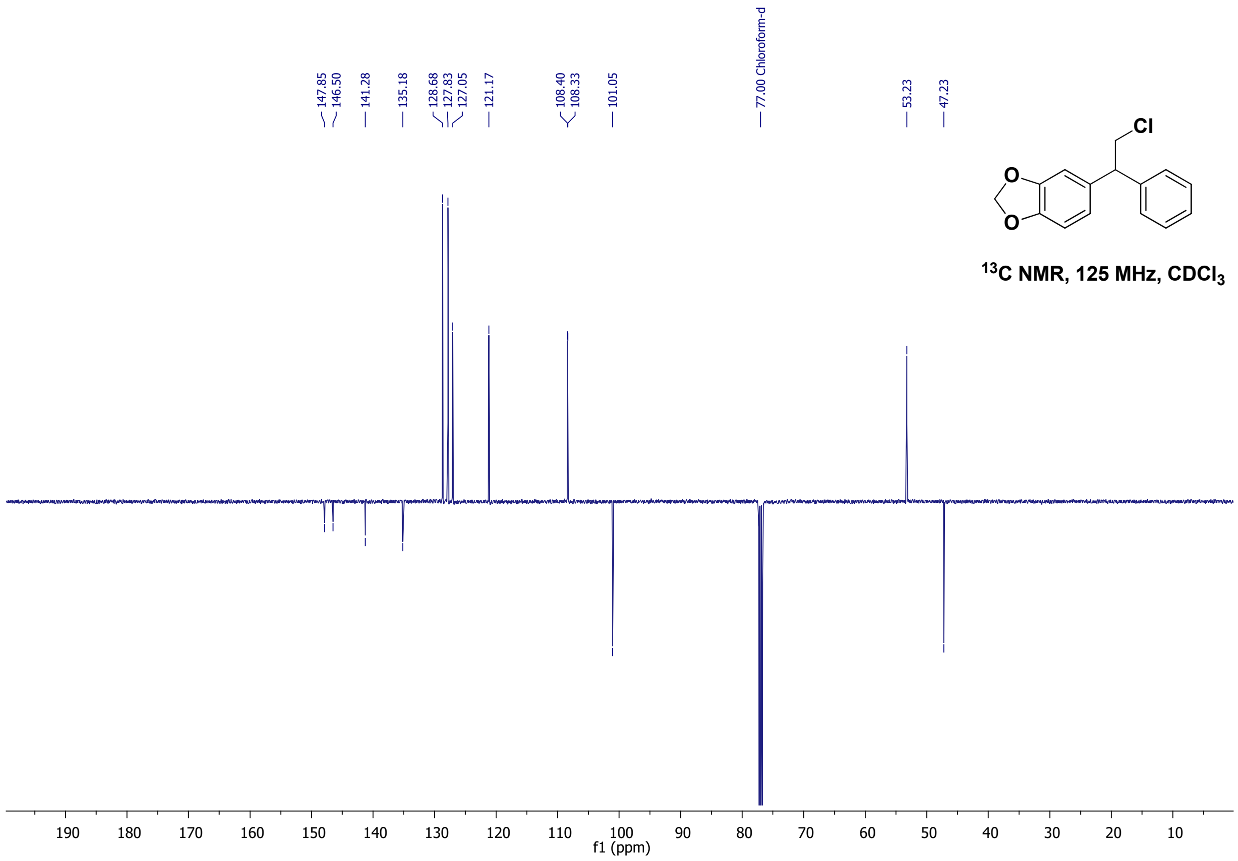

2,2'-(2-Chloro-1,1-ethanediyl)dithiophene (65)

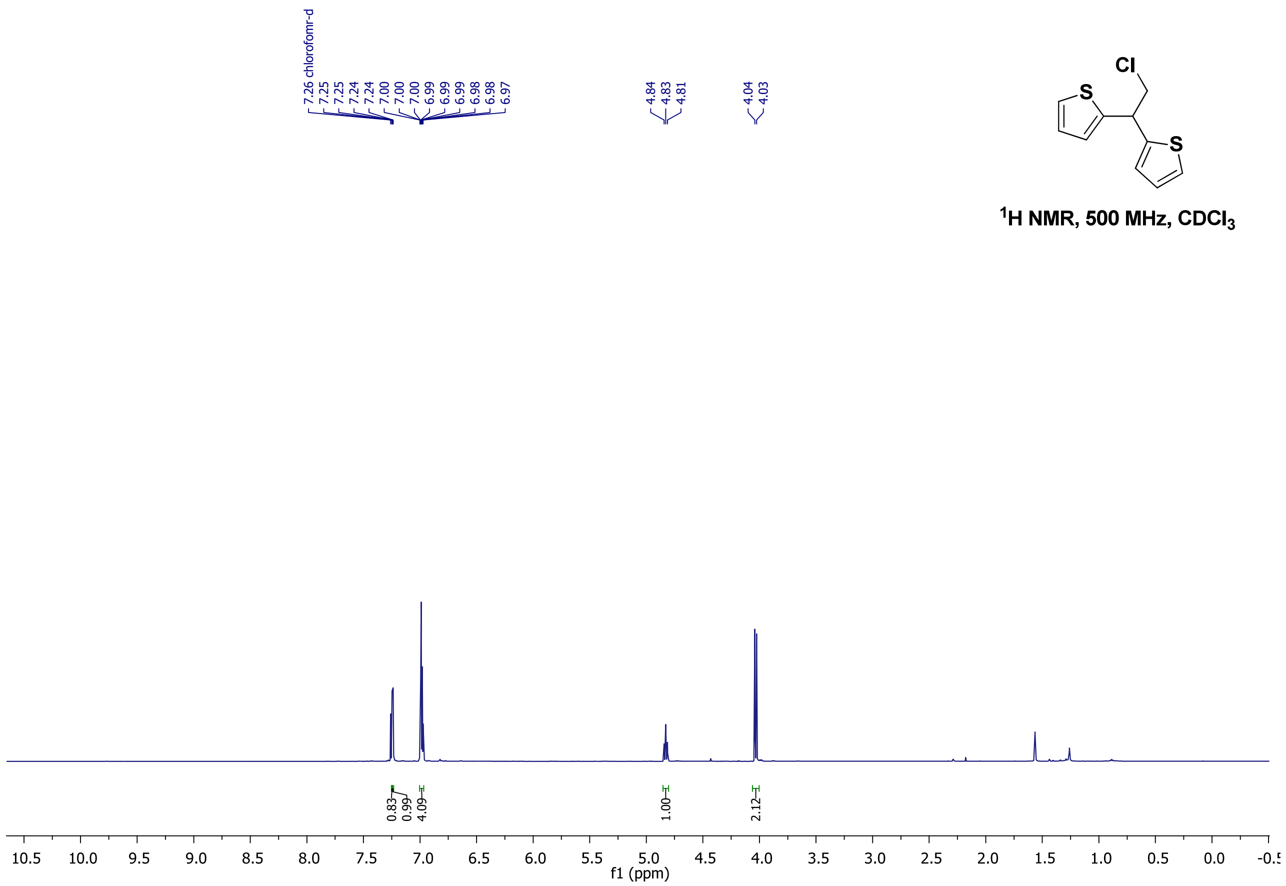

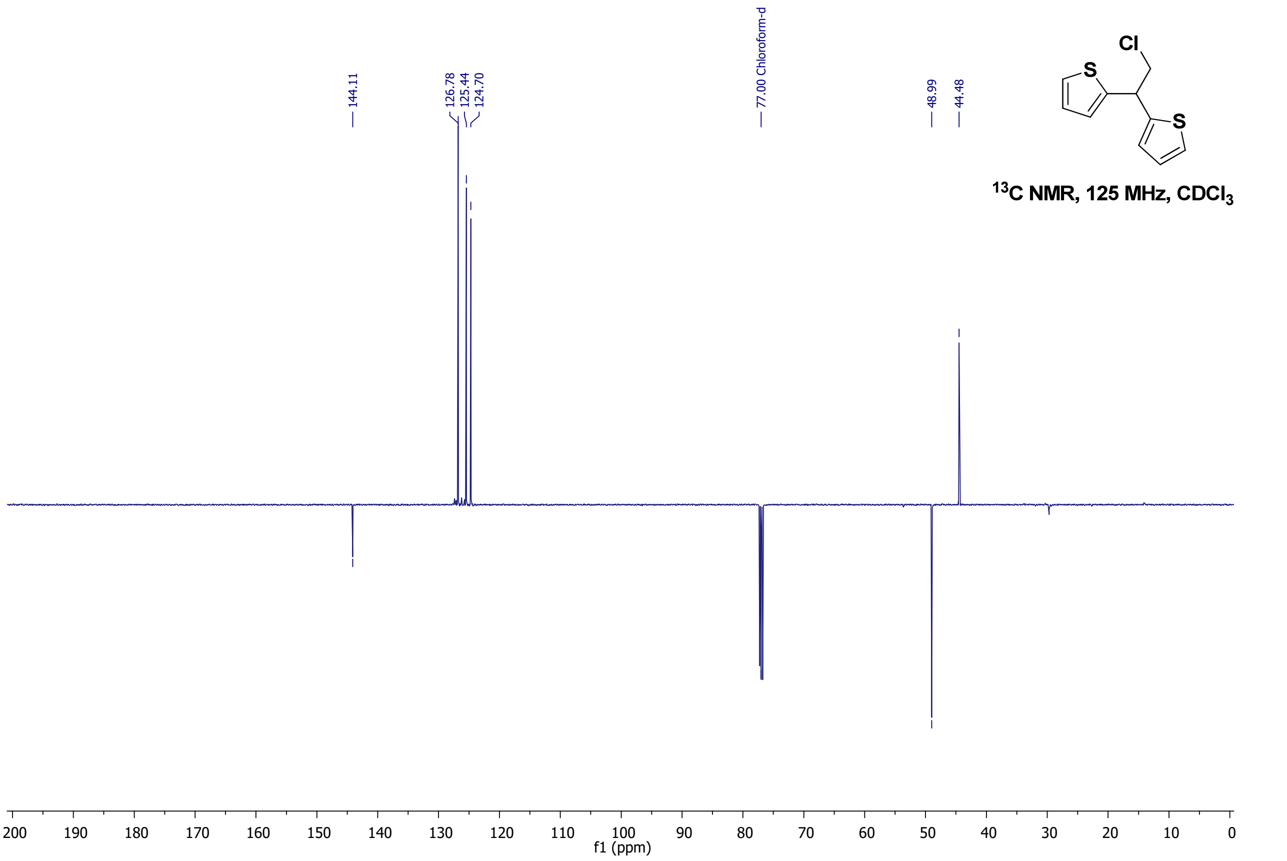

(1-Bromo-2-propanyl)benzene (66)

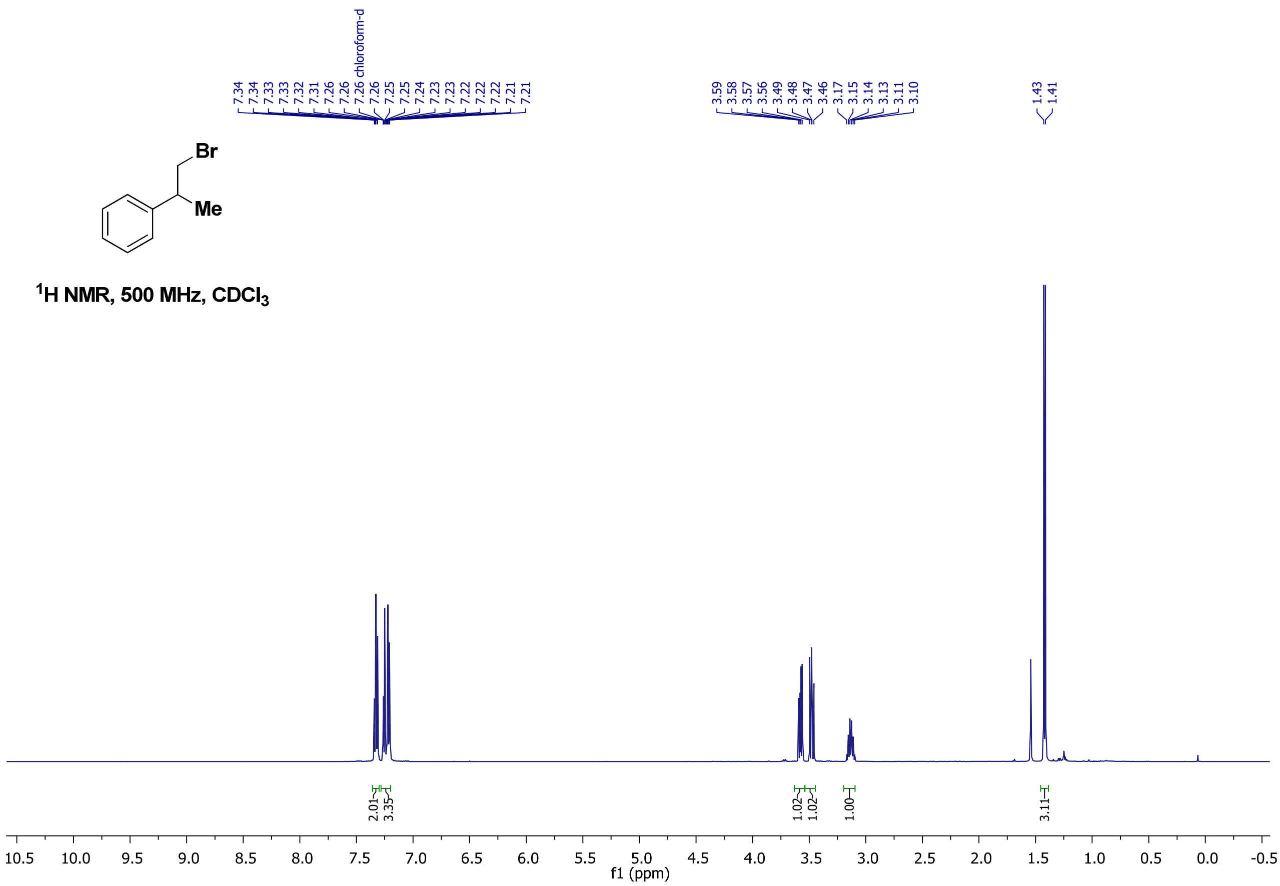

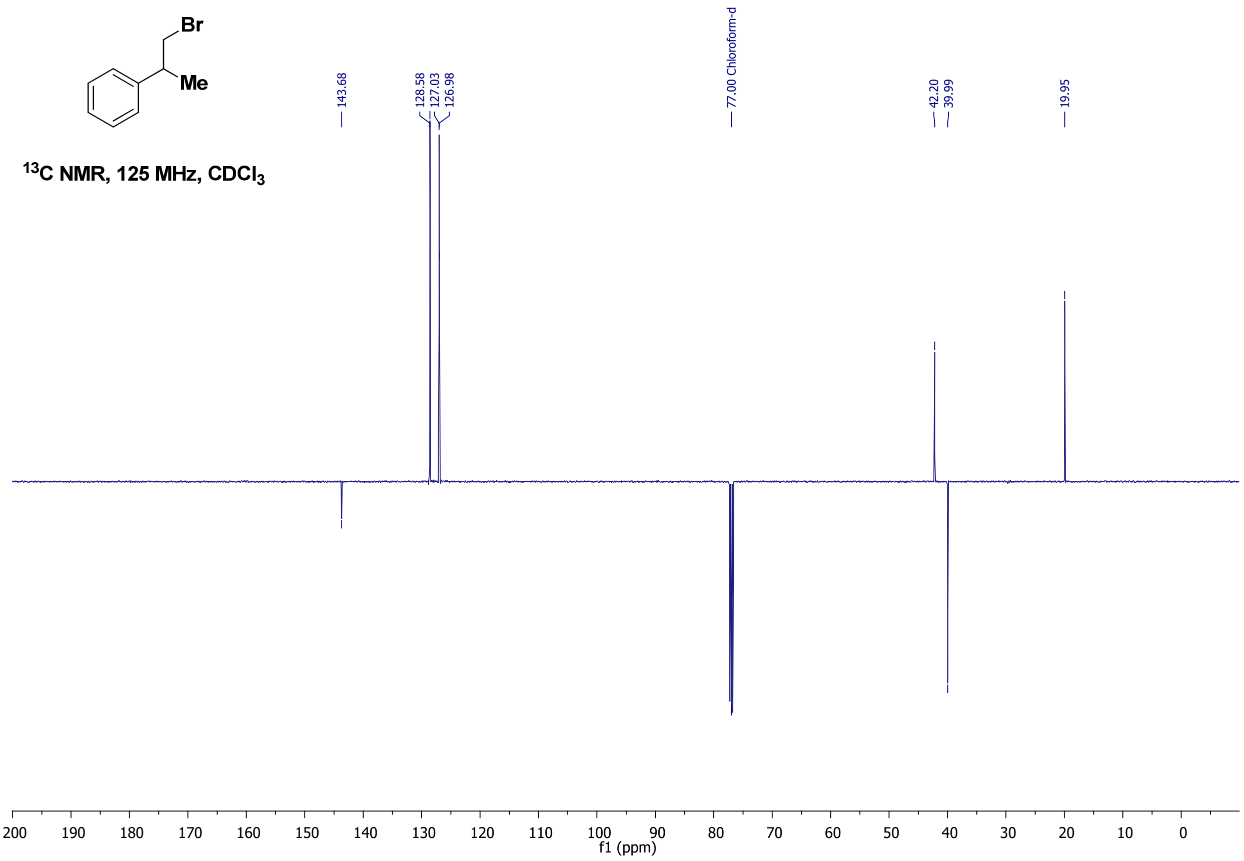

1,1'-(2-Bromo-1,1-ethanediyl)dibenzene (67)

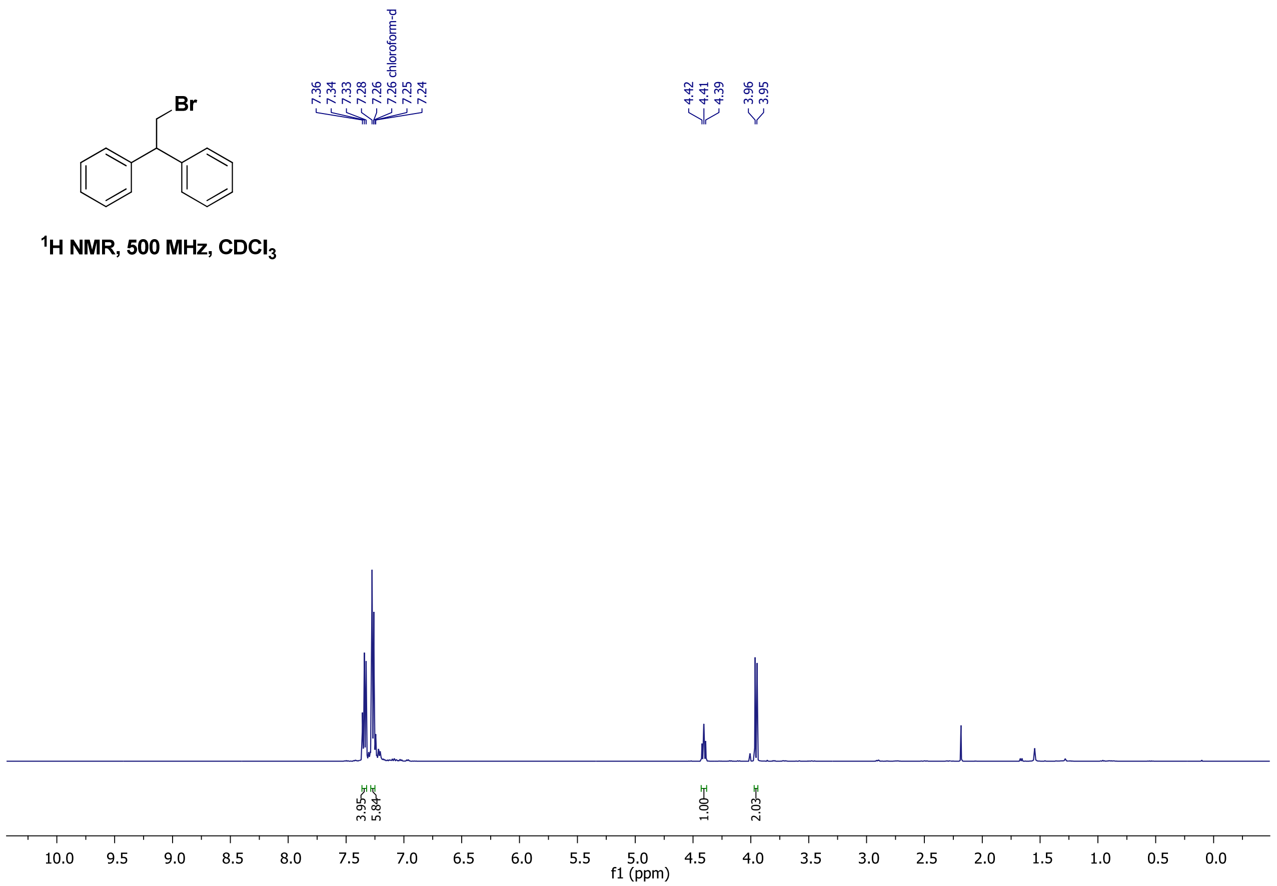

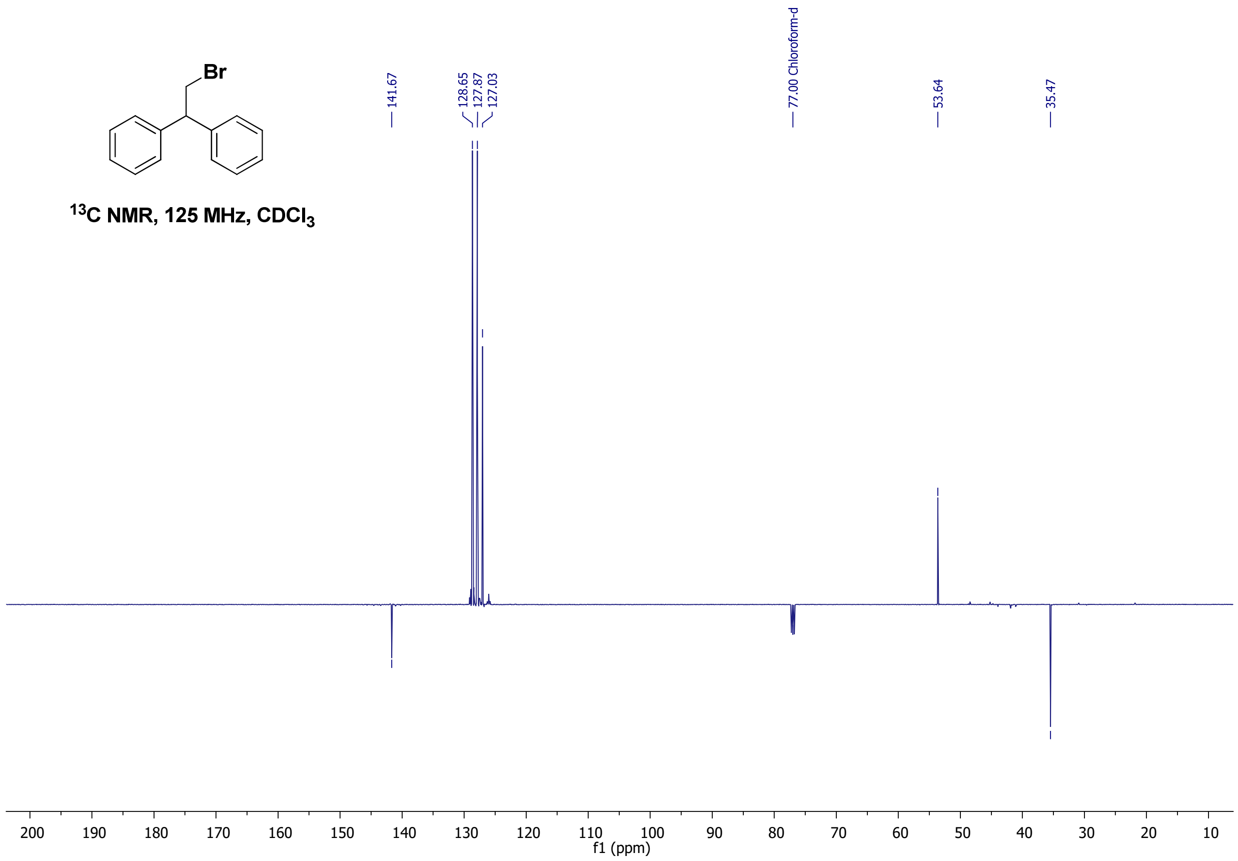

1,1'-(2-Fluoroethane-1,1-diyl)dibenzene (68)

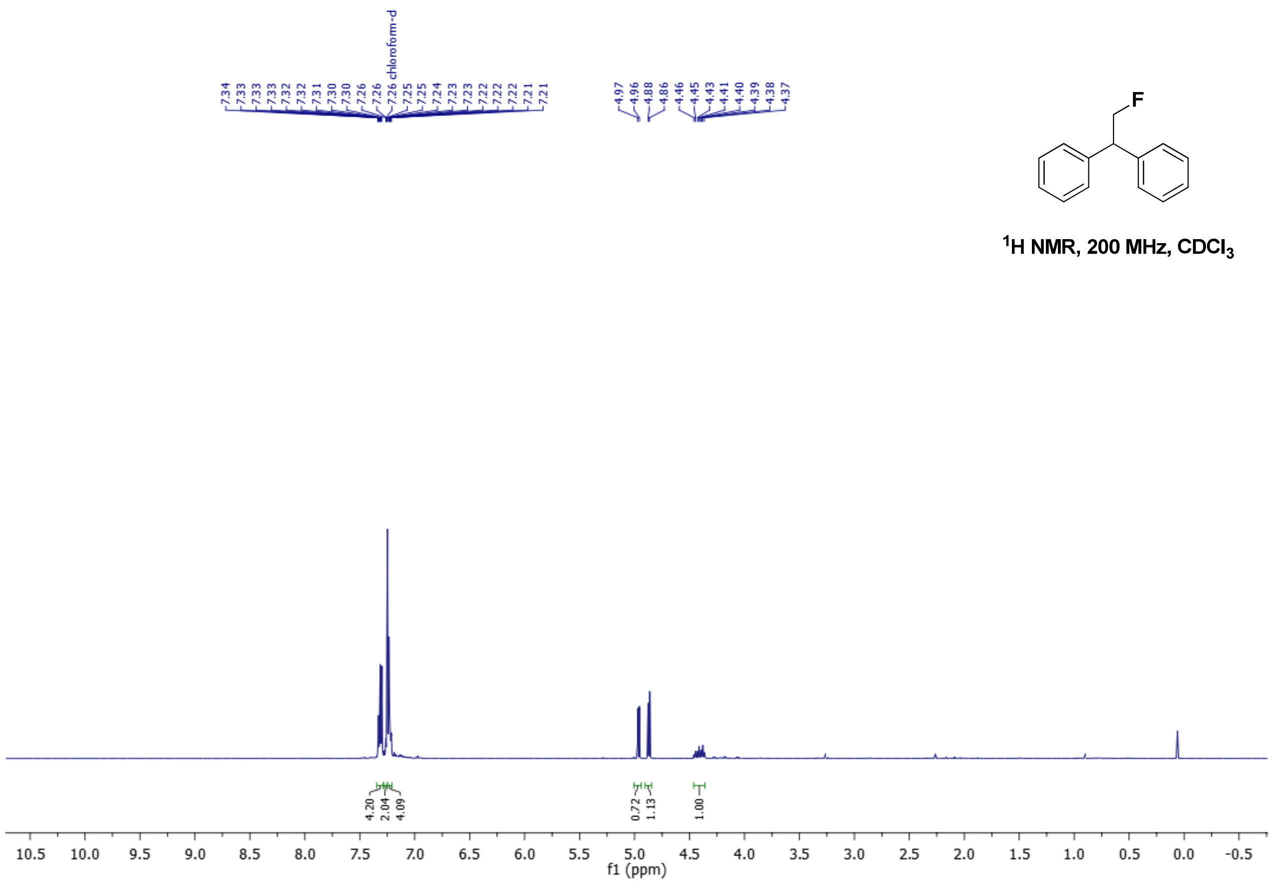

^{1}H NMR, 200 MHz, $CDCl_3$

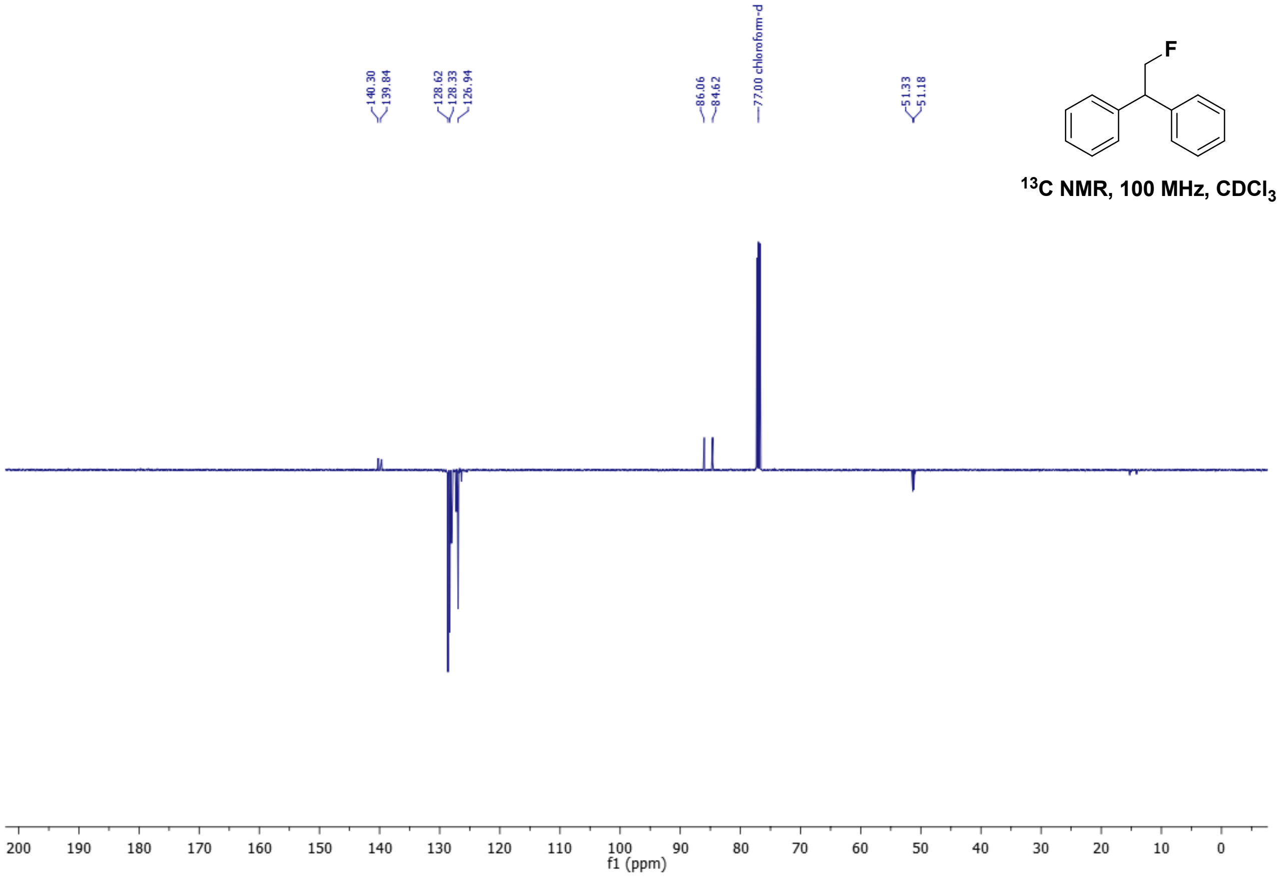

^{13}C NMR, 100 MHz, $CDCl_3$

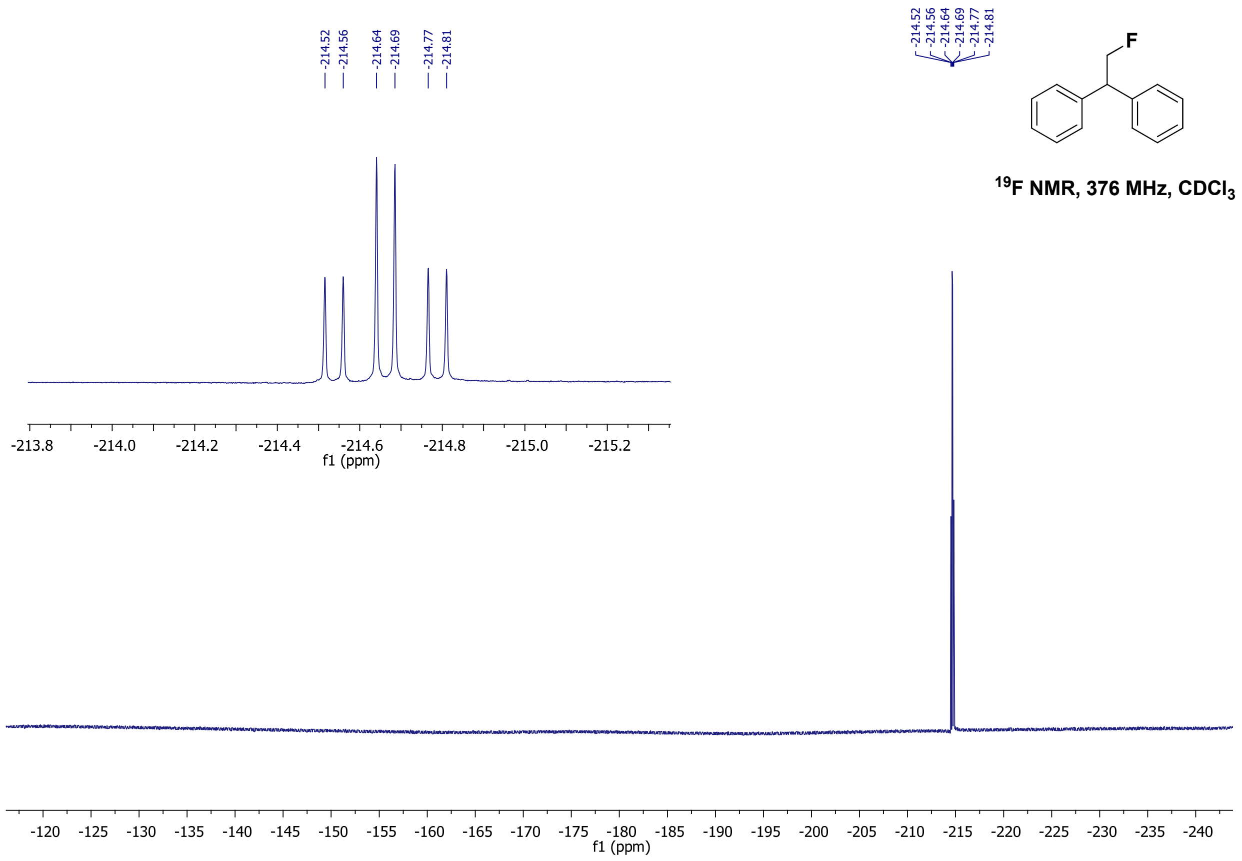
-214.52
-214.56
-214.64
-214.69
-214.77
-214.81
F
19F NMR, 376 MHz, CDCl3
-213.8 -214.0 -214.2 -214.4 -214.6 -214.8 -215.0 -215.2
f1 (ppm)
-120 -125 -130 -135 -140 -145 -150 -155 -160 -165 -170 -175 -180 -185 -190 -195 -200 -205 -210 -215 -220 -225 -230 -235 -240
f1 (ppm)

9-(Chloromethyl)-9*H*-xanthene (69)

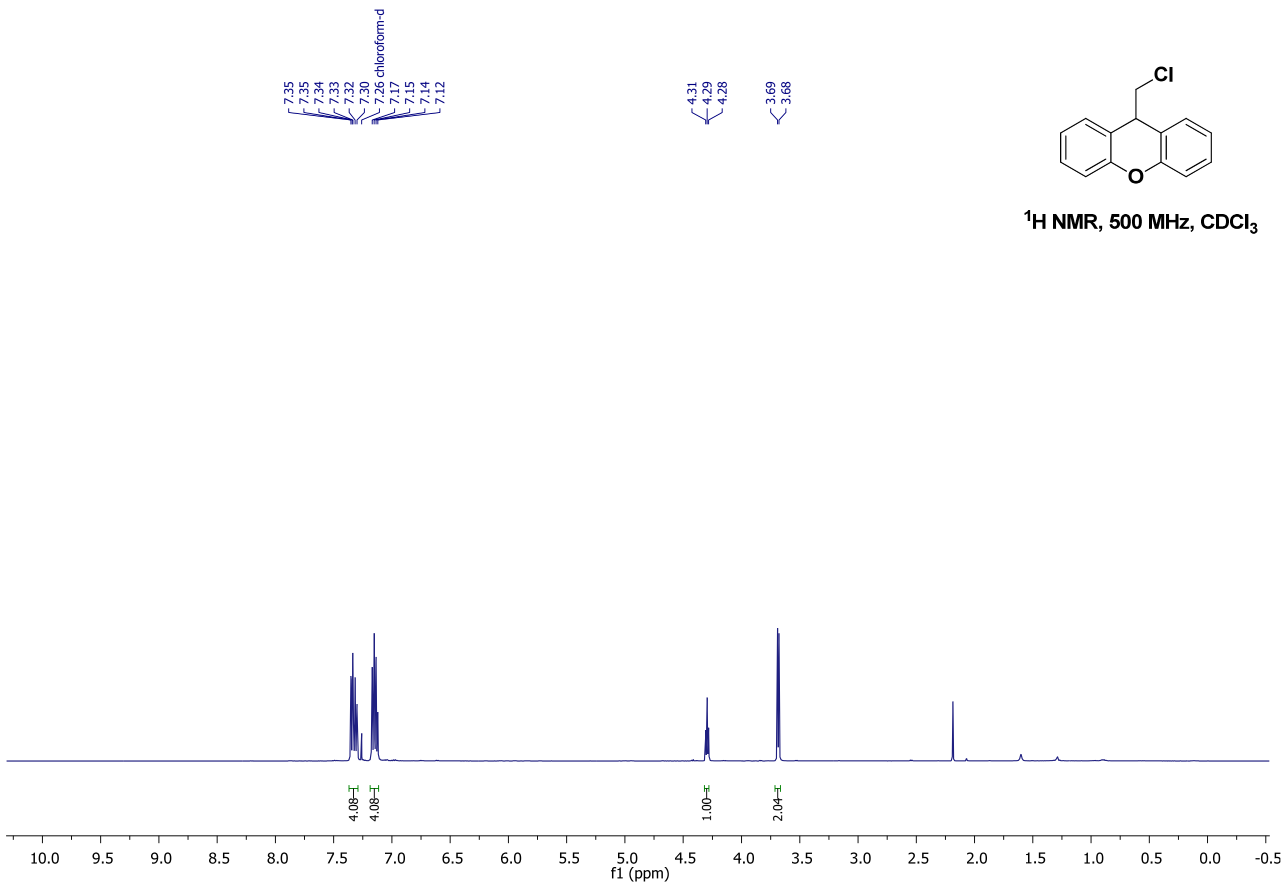

^{1}H NMR, 500 MHz, $CDCl_3$

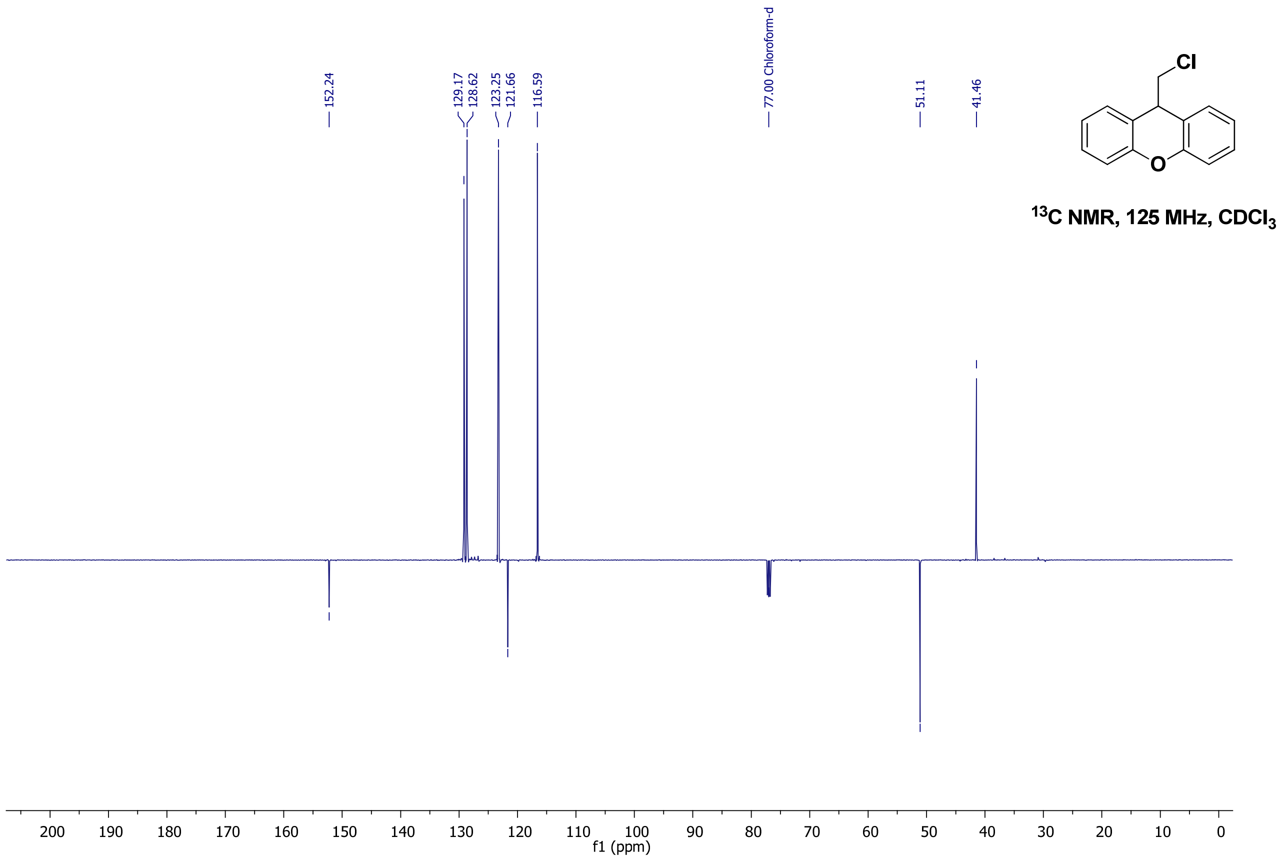

^{13}C NMR, 125 MHz, $CDCl_3$

9-(Chloromethyl)-9*H*-thioxanthene (70)

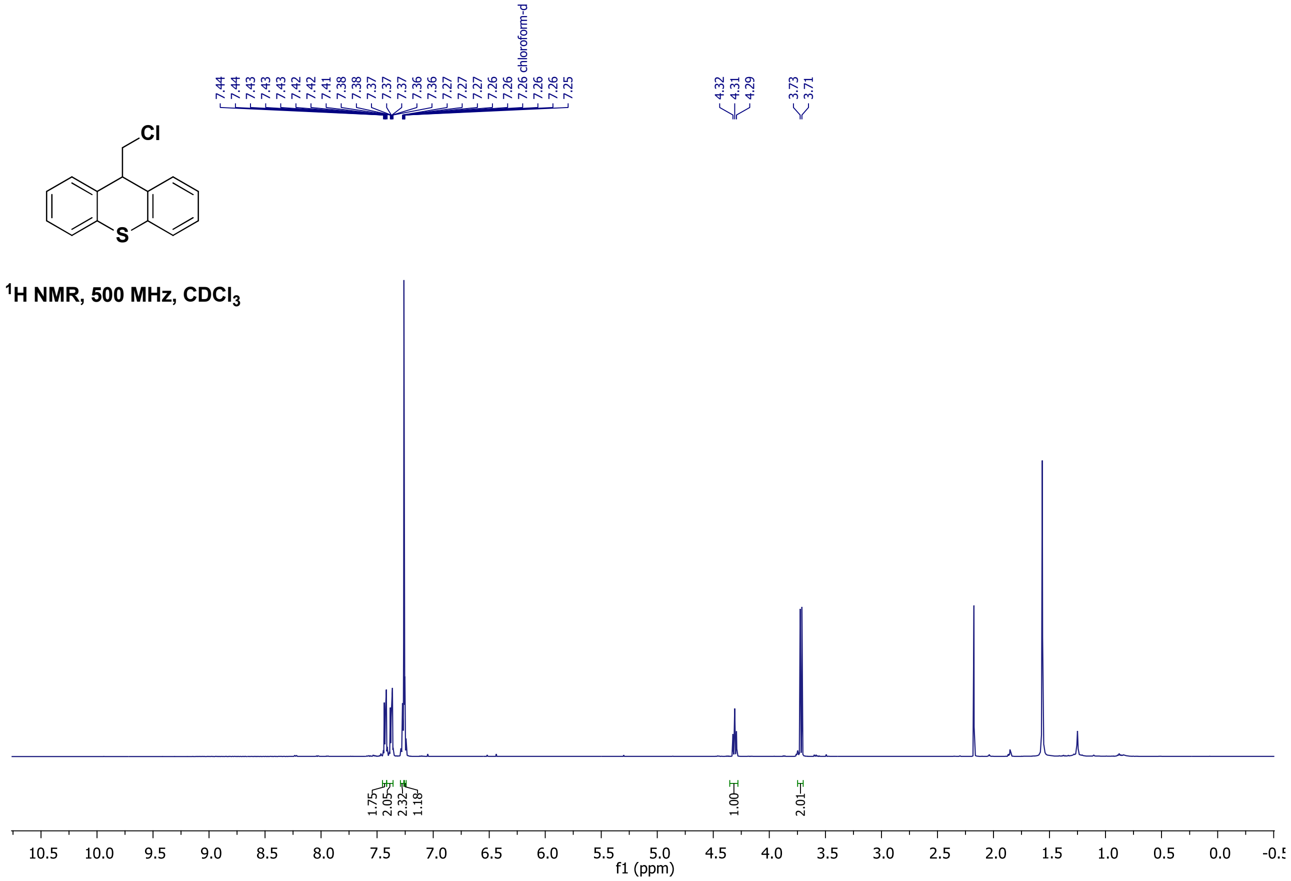

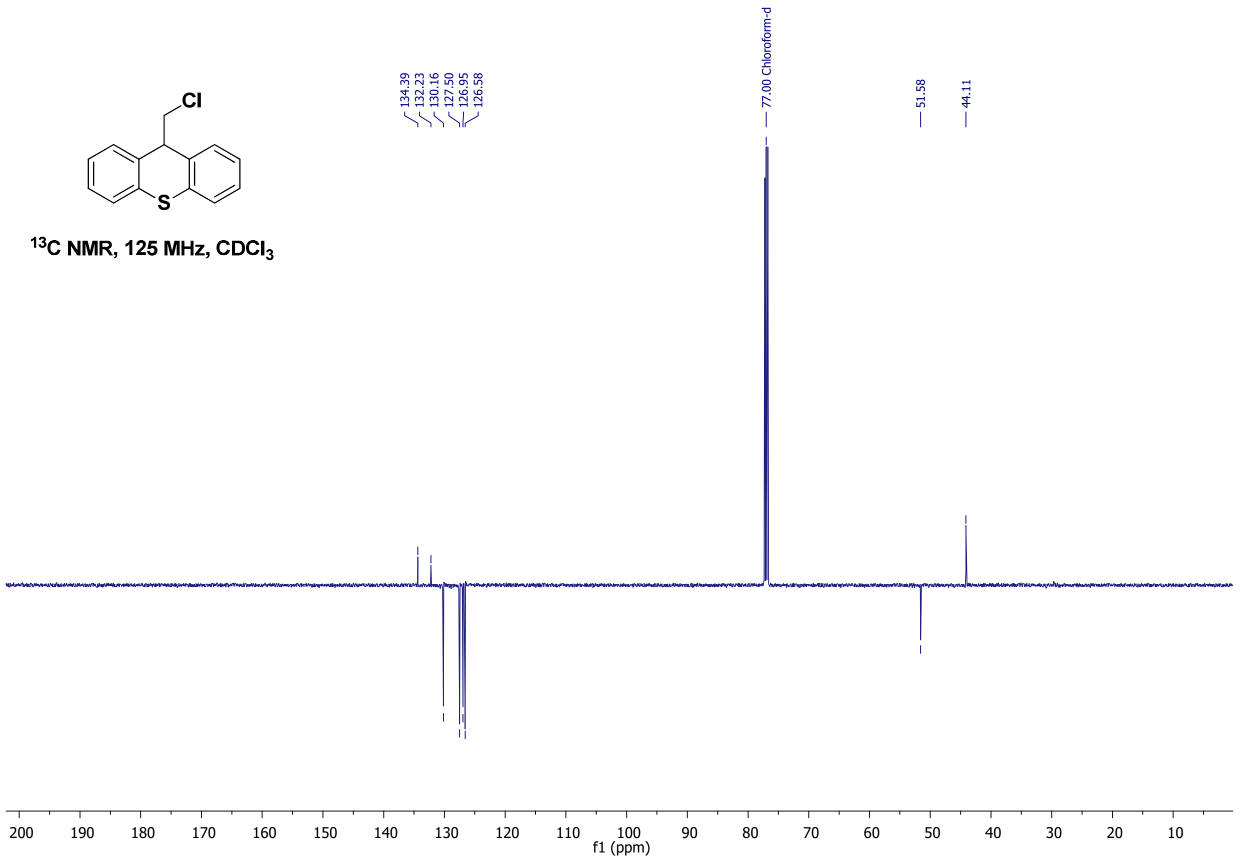

2-Chloro-9-(chloromethyl)-9*H*-thioxanthene (71)

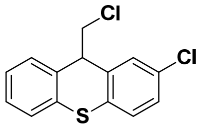

^{1}H NMR, 400 MHz, $CDCl_3$

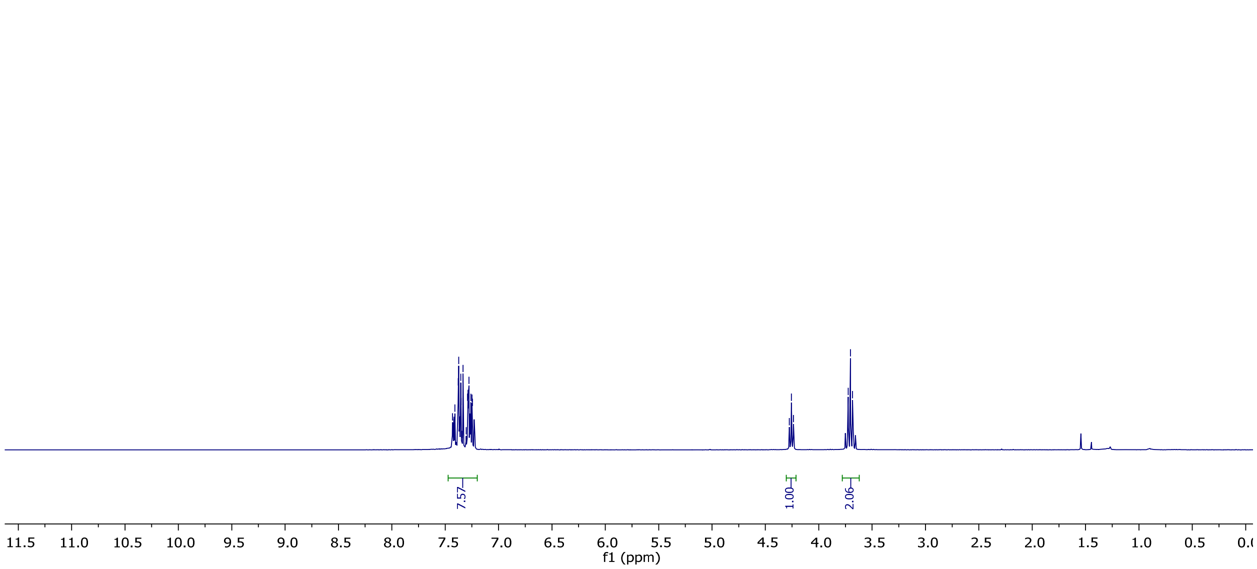

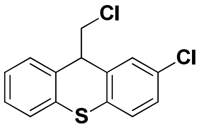

^{13}C NMR, 100 MHz, $CDCl_3$

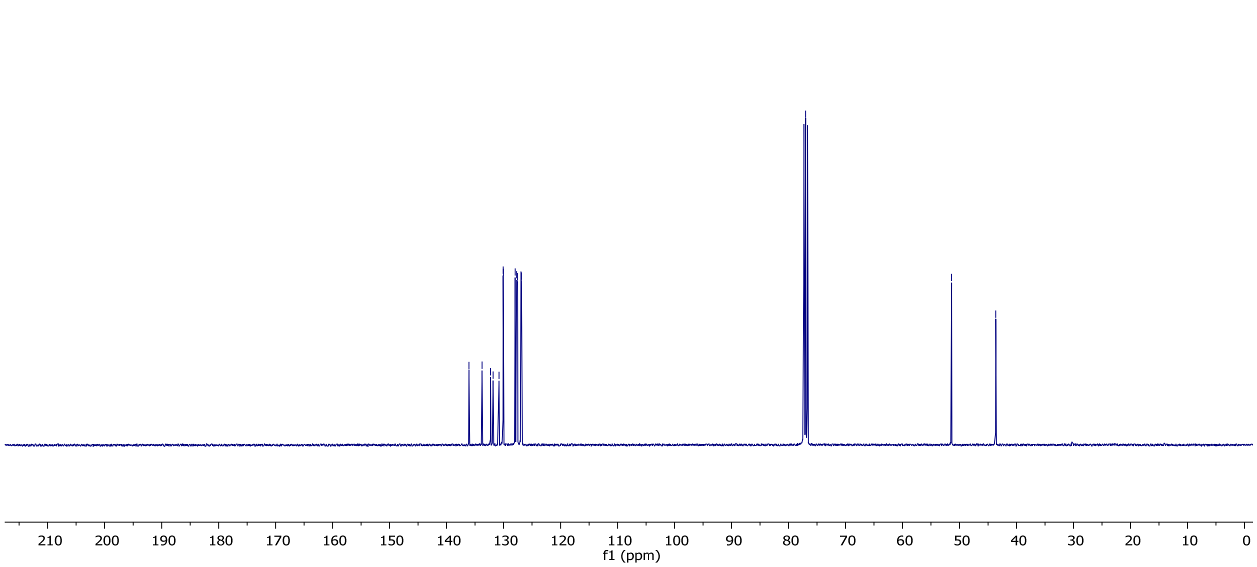

9-(Bromomethyl)-9*H*-thioxanthene (72)

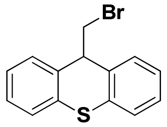

^{1}H NMR, 400 MHz, C_6D_6

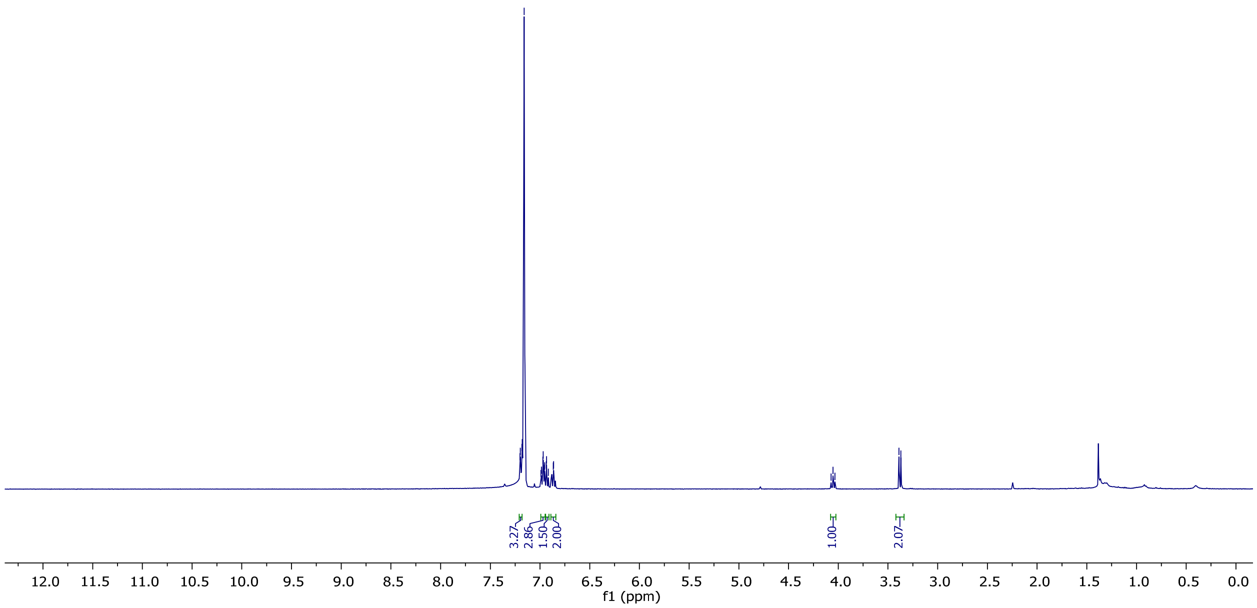

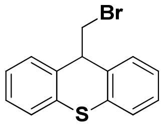

^{13}C NMR, 100 MHz, C_6D_6

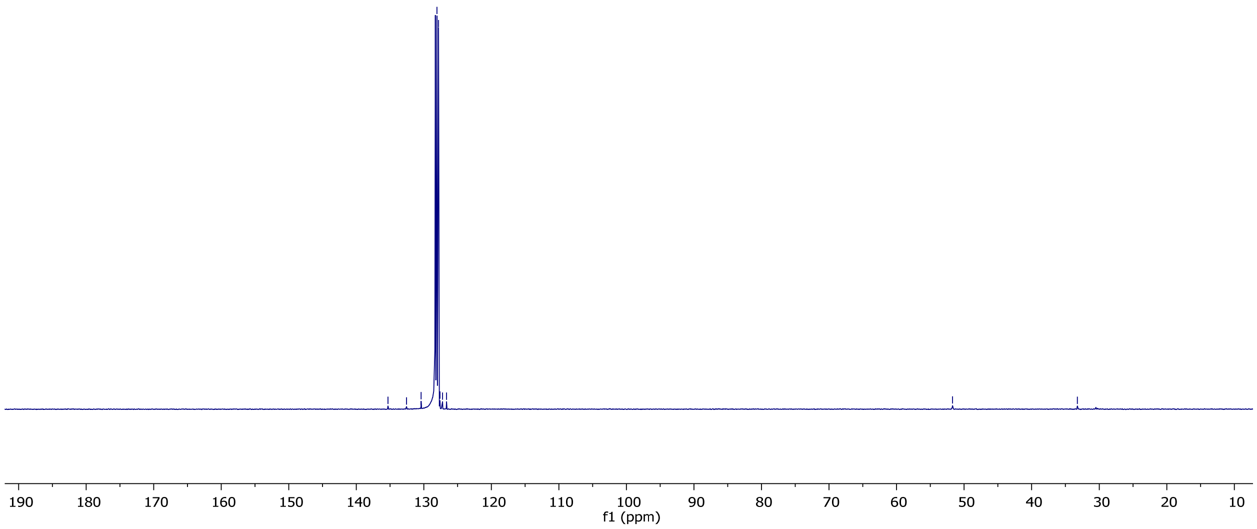

1,1'-(2,2-Dibromo-1,1-ethanediyl)dibenzene (73)

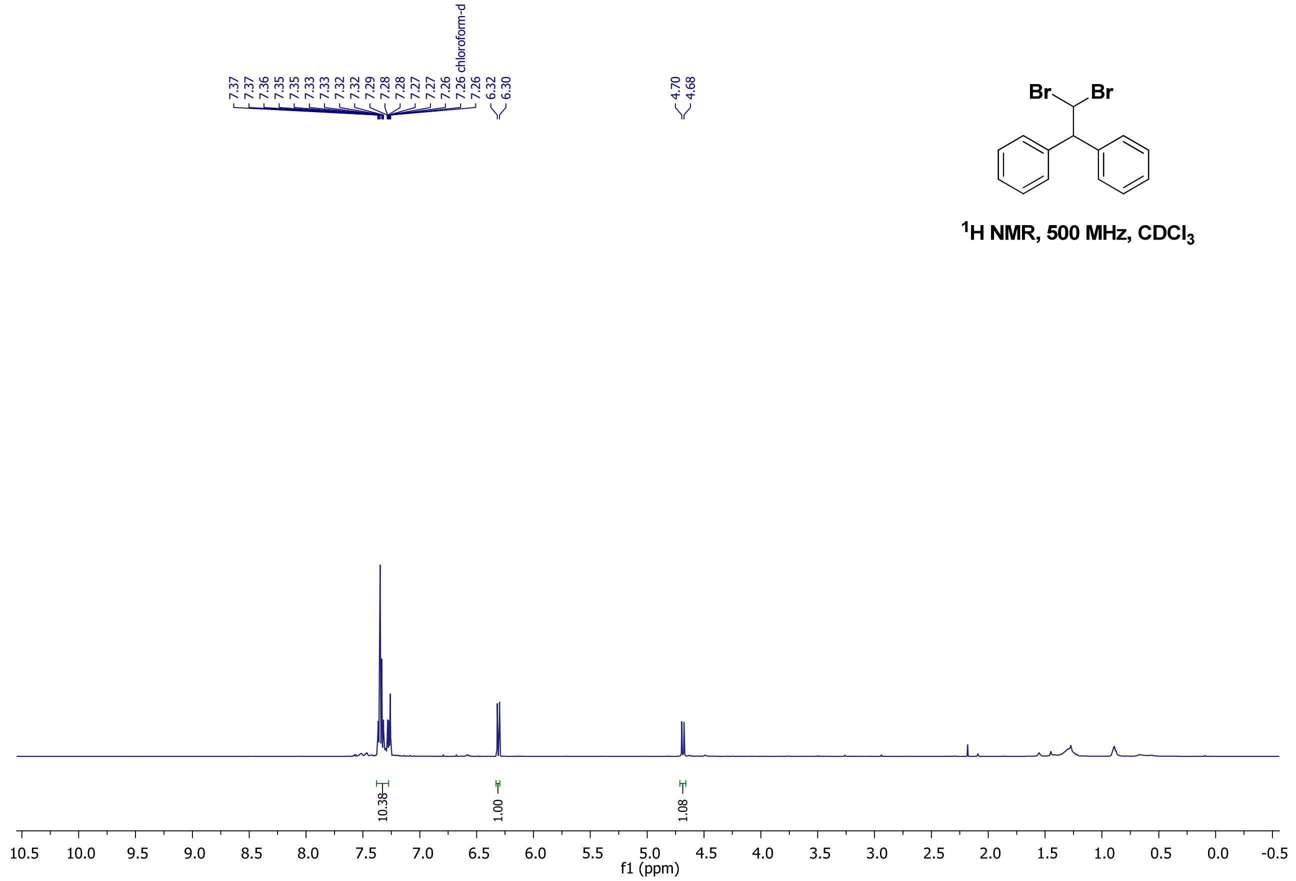

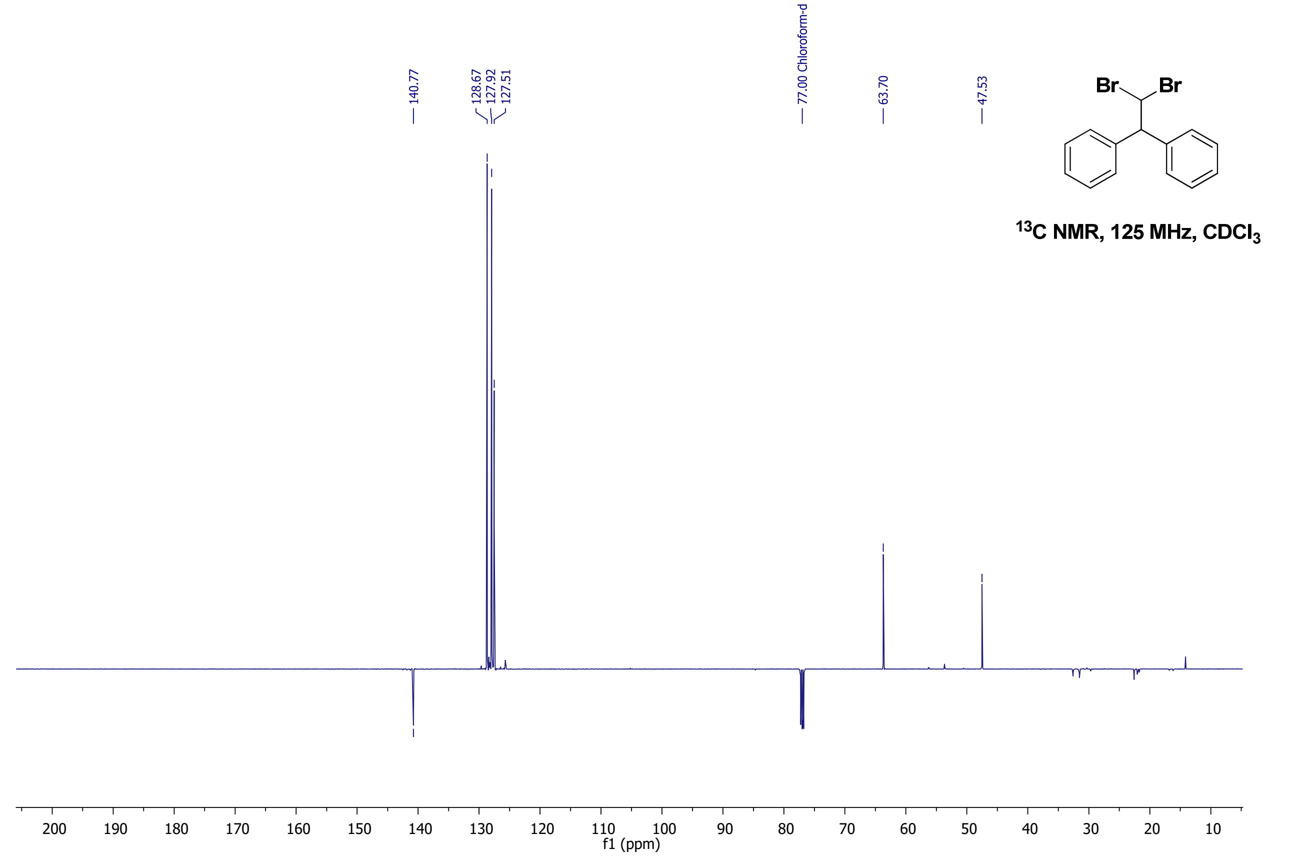

(2,2-Dibromoethyl)benzene (74)

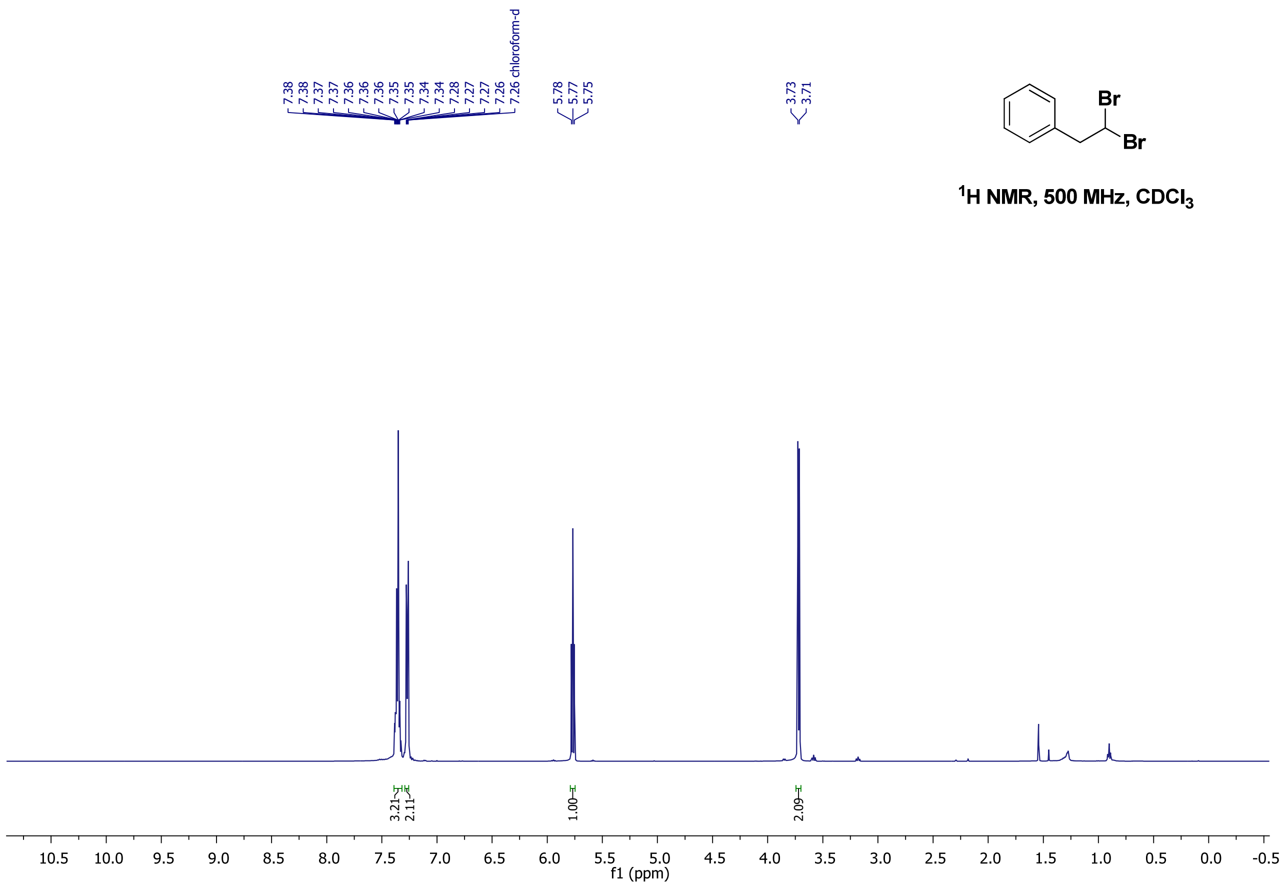

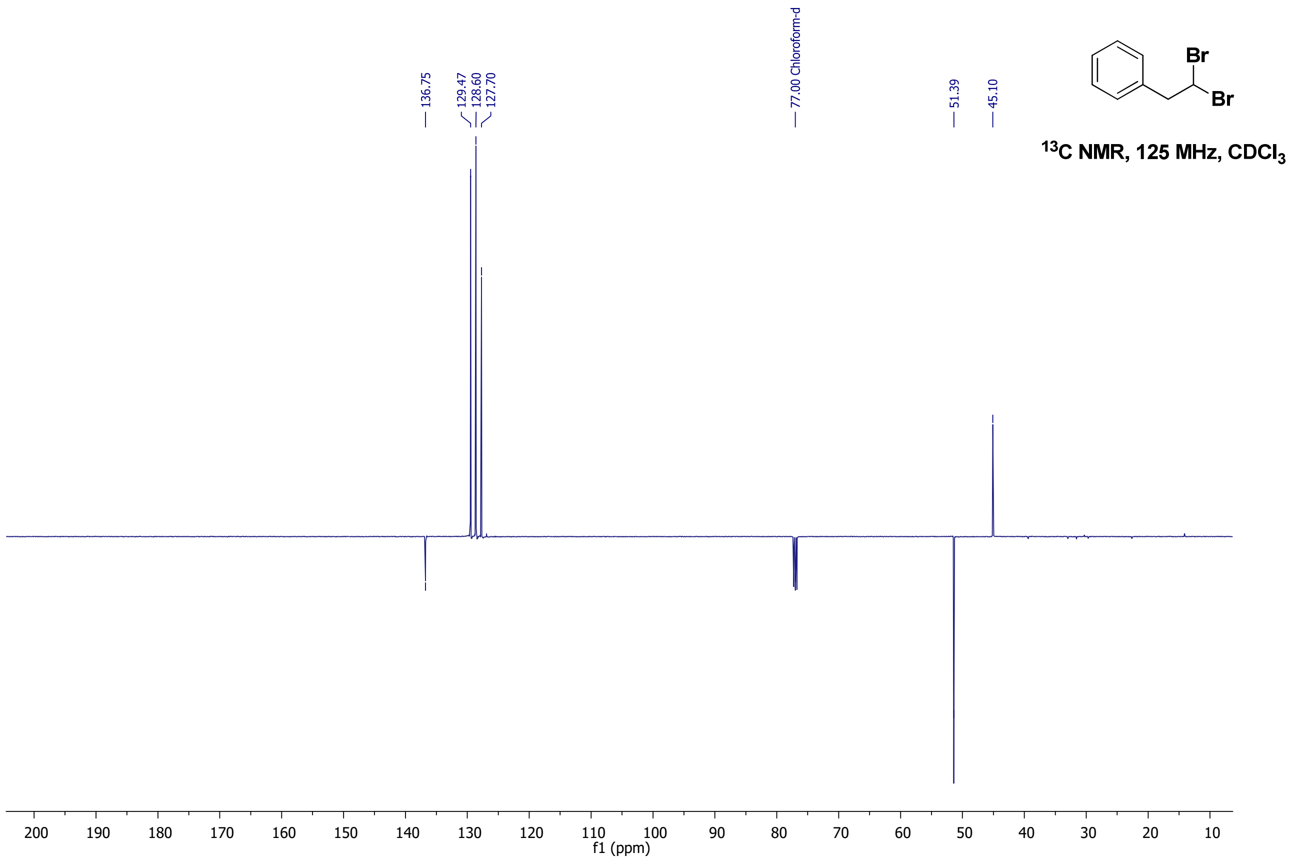

1,1'-(2,2-Dichloro-1,1-ethanediyl)dibenzene (75)

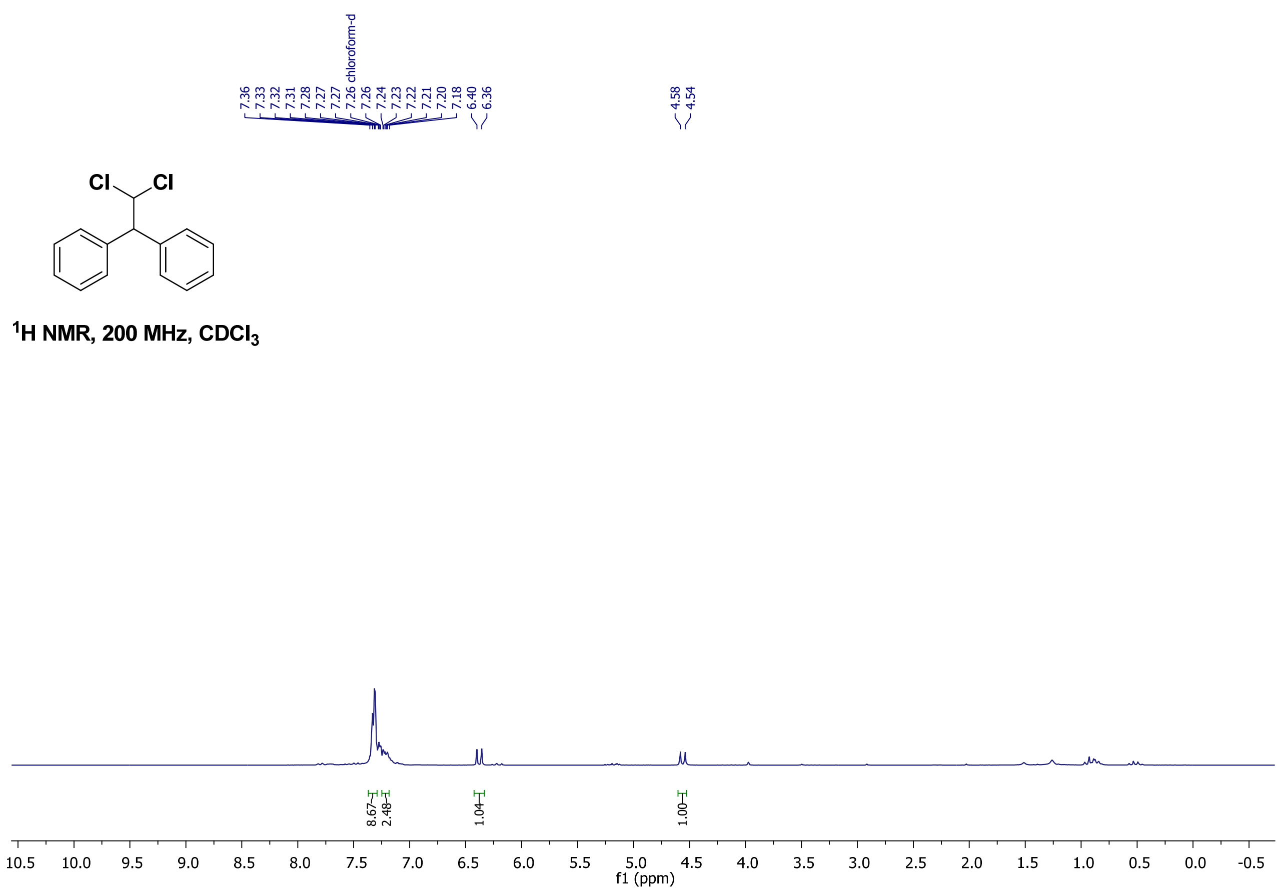

^{1}H NMR, 200 MHz, $CDCl_3$

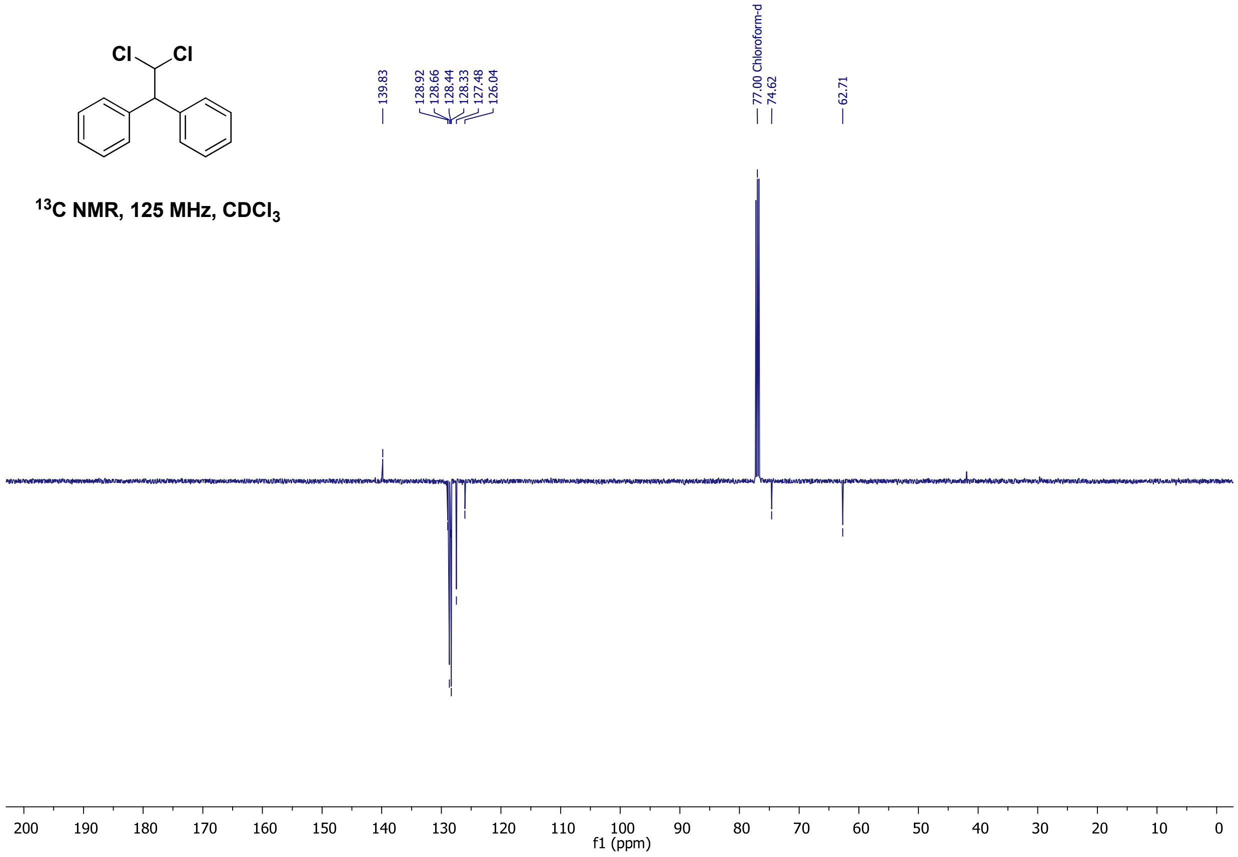

^{13}C NMR, 125 MHz, $CDCl_3$

1-Bromo-4-(1,1-dichloropropan-2-yl)benzene (76)

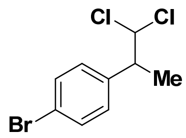

^1H NMR, 400 MHz, $CDCl_3$

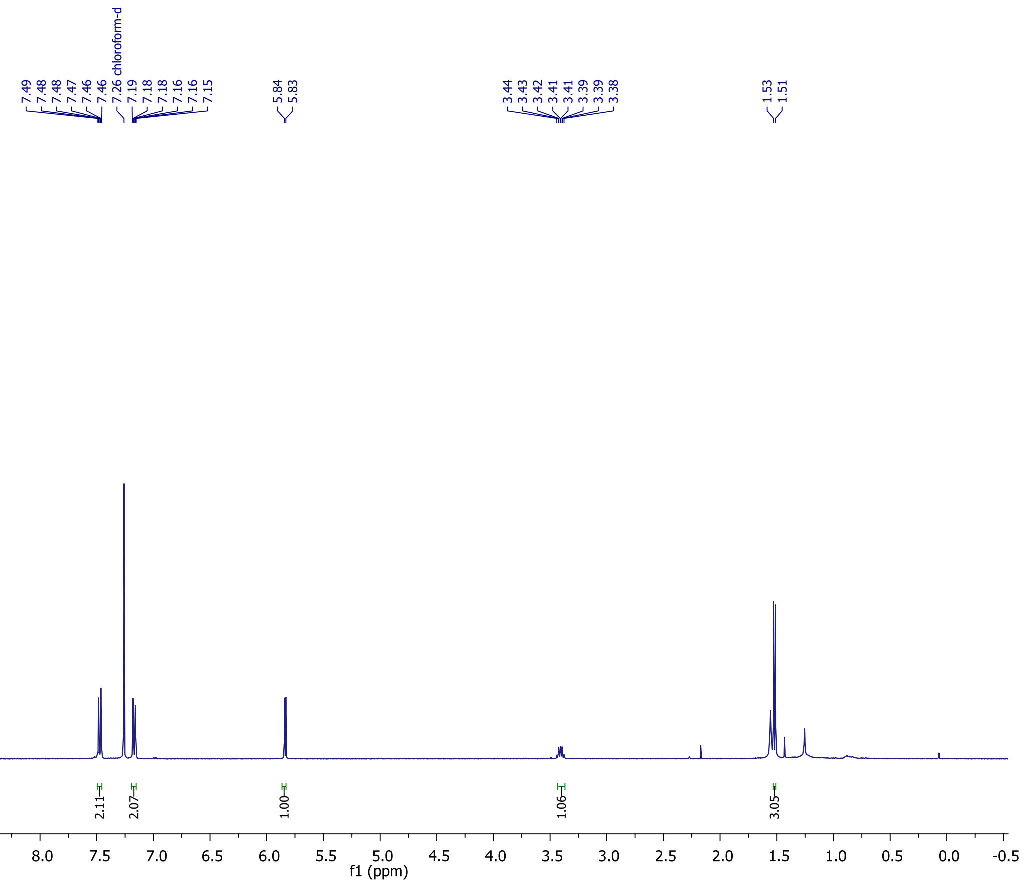

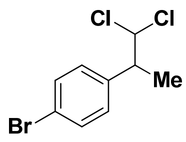

^{13}C NMR, 100 MHz, $CDCl_3$

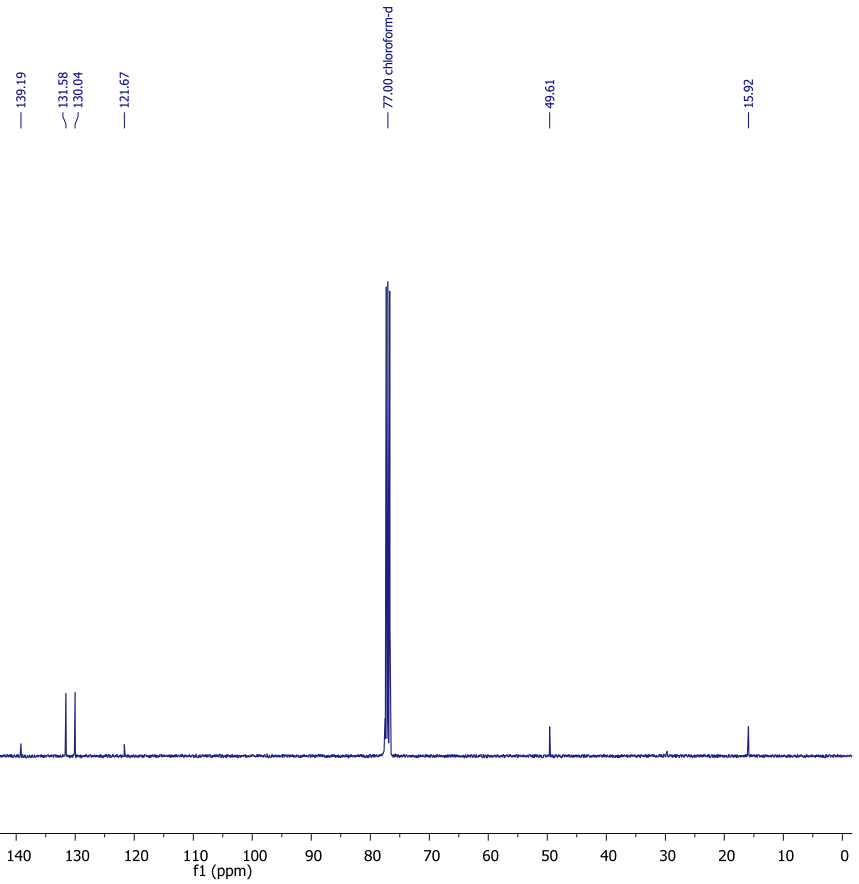

1-(2-Bromo-2-chloroethyl)-2,4-dichlorobenzene (77)

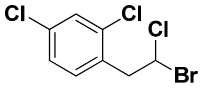

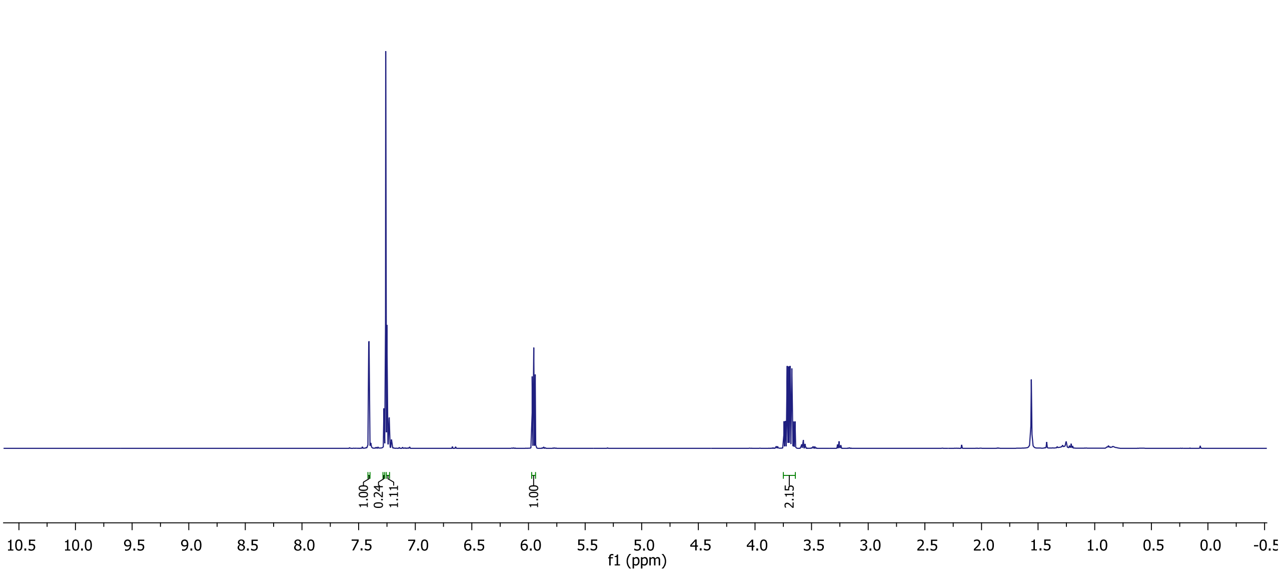

^{1}H NMR, 500 MHz, $CDCl_3$

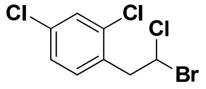

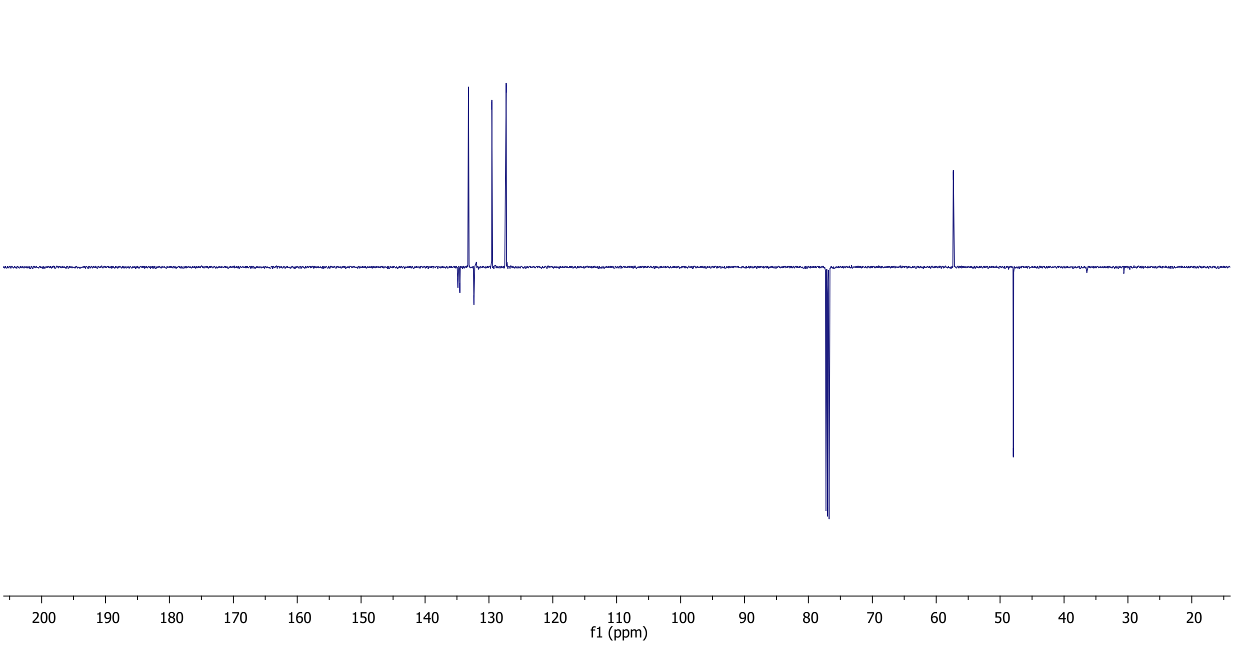

^{13}C NMR, 125 MHz, $CDCl_3$

2-(2-Bromo-2-chloroethyl)-1,3-dichlorobenzene (78)

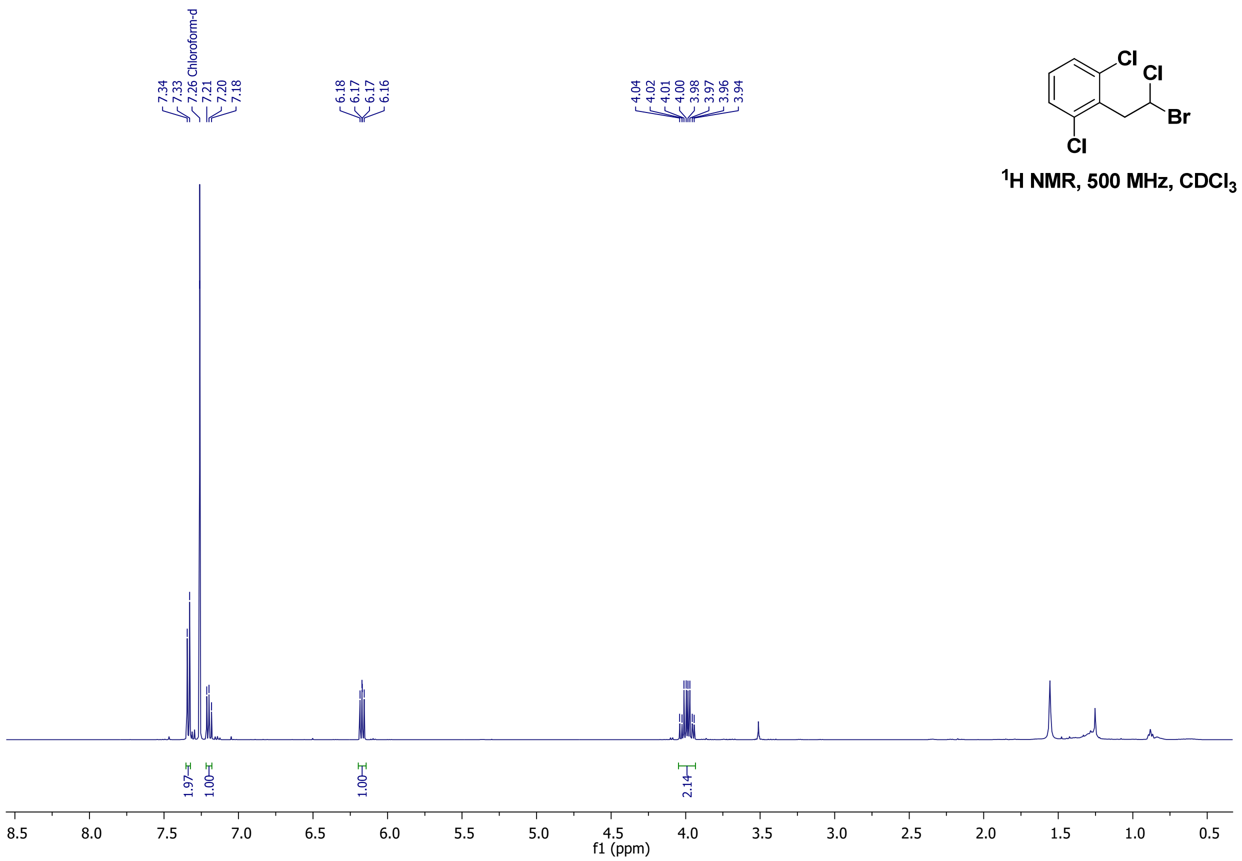

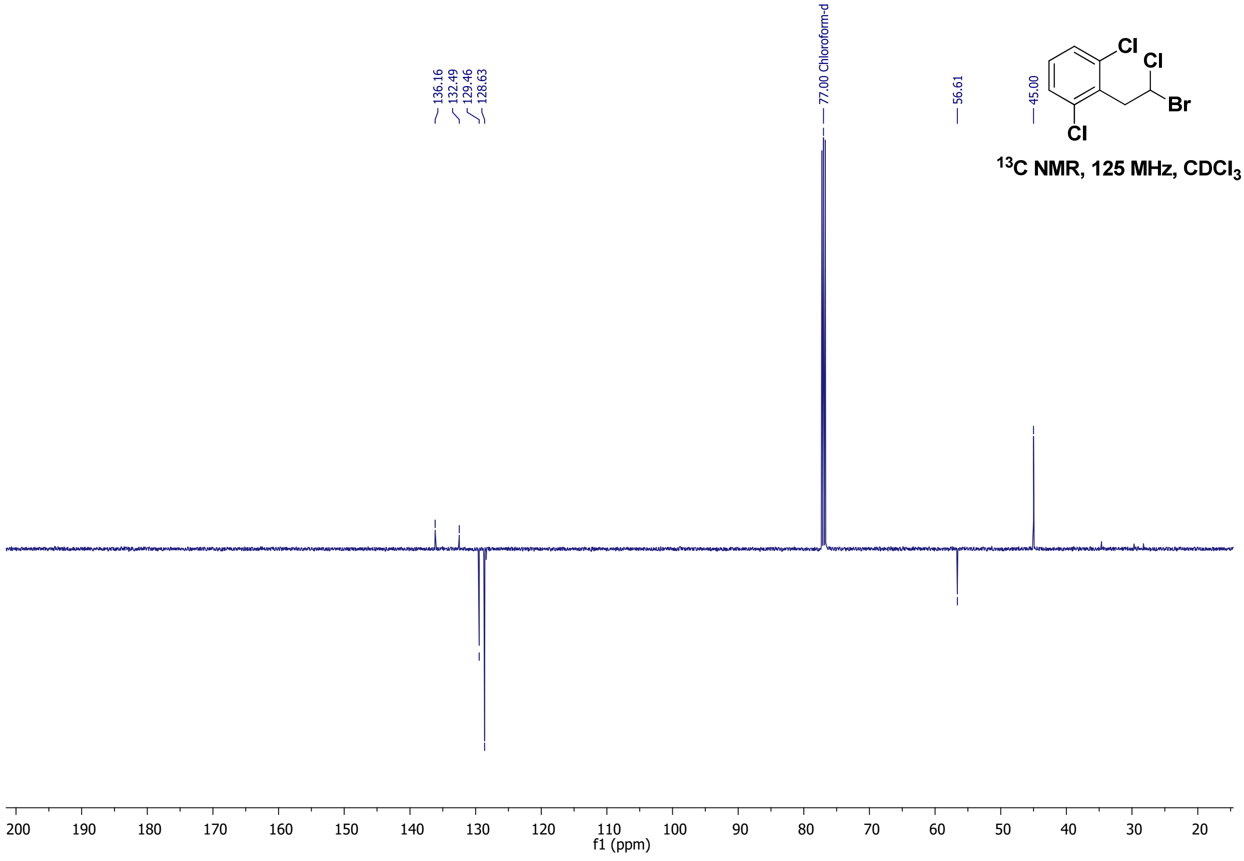

(2-Chloro-2-iodoethyl)benzene (79)

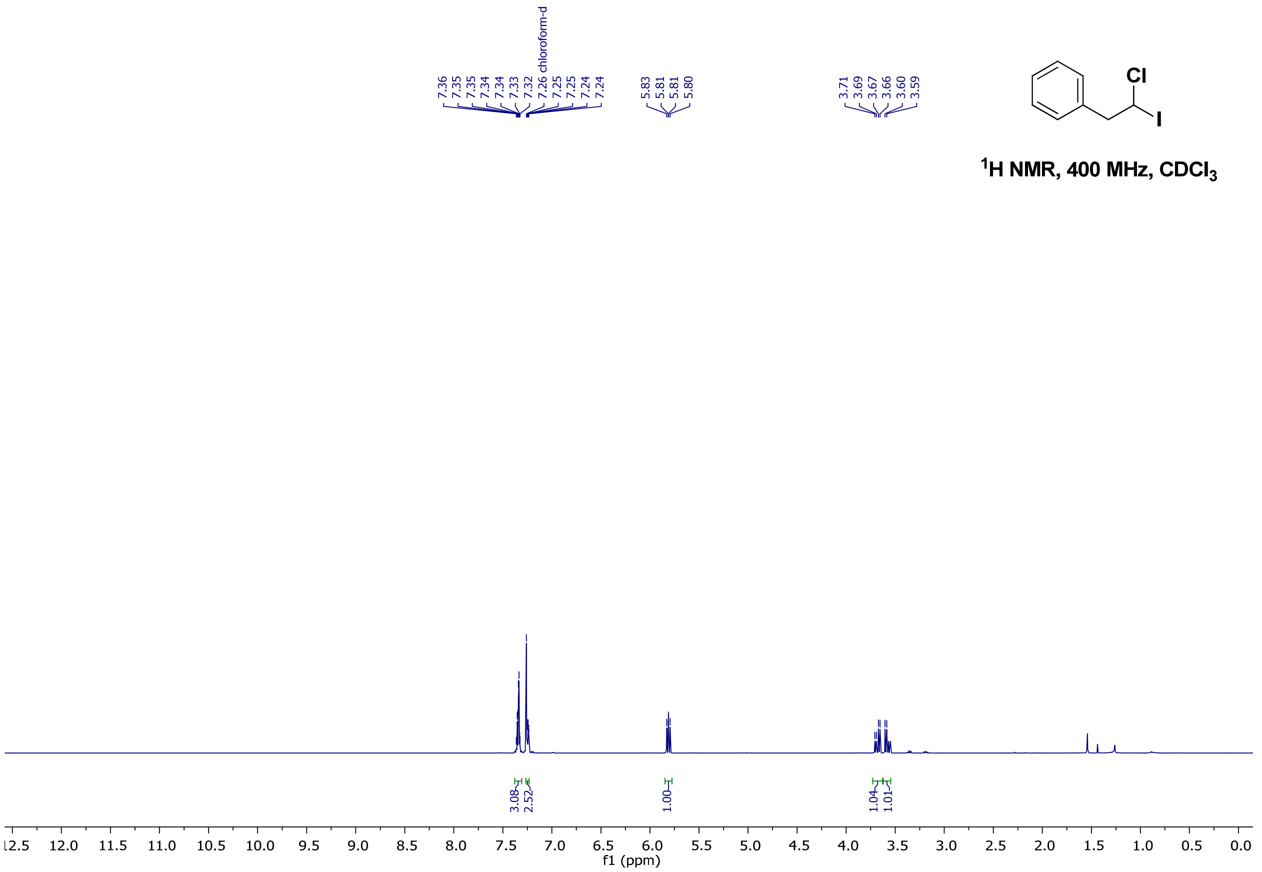

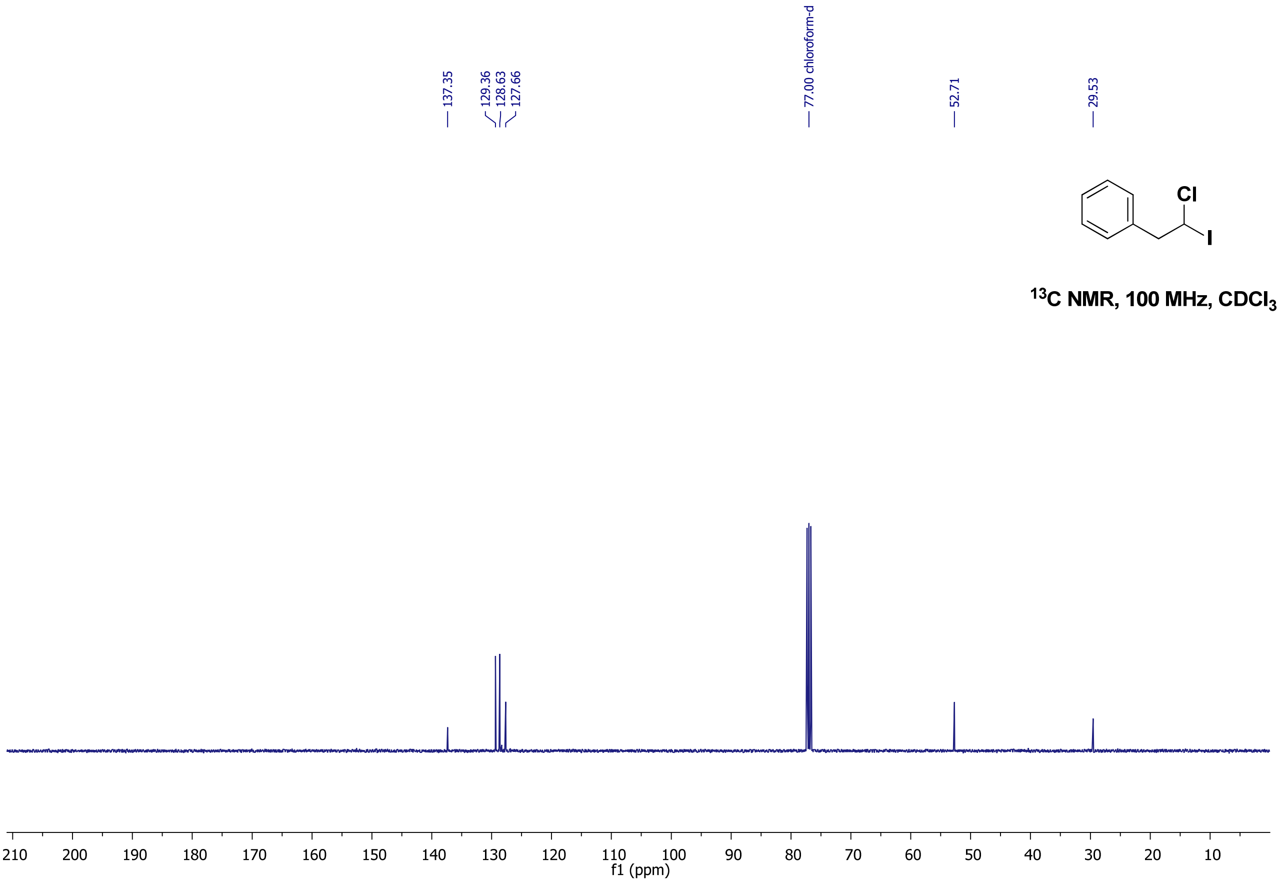

2-(2-Bromo-2-iodoethyl)-1,3-dimethoxybenzene (80)

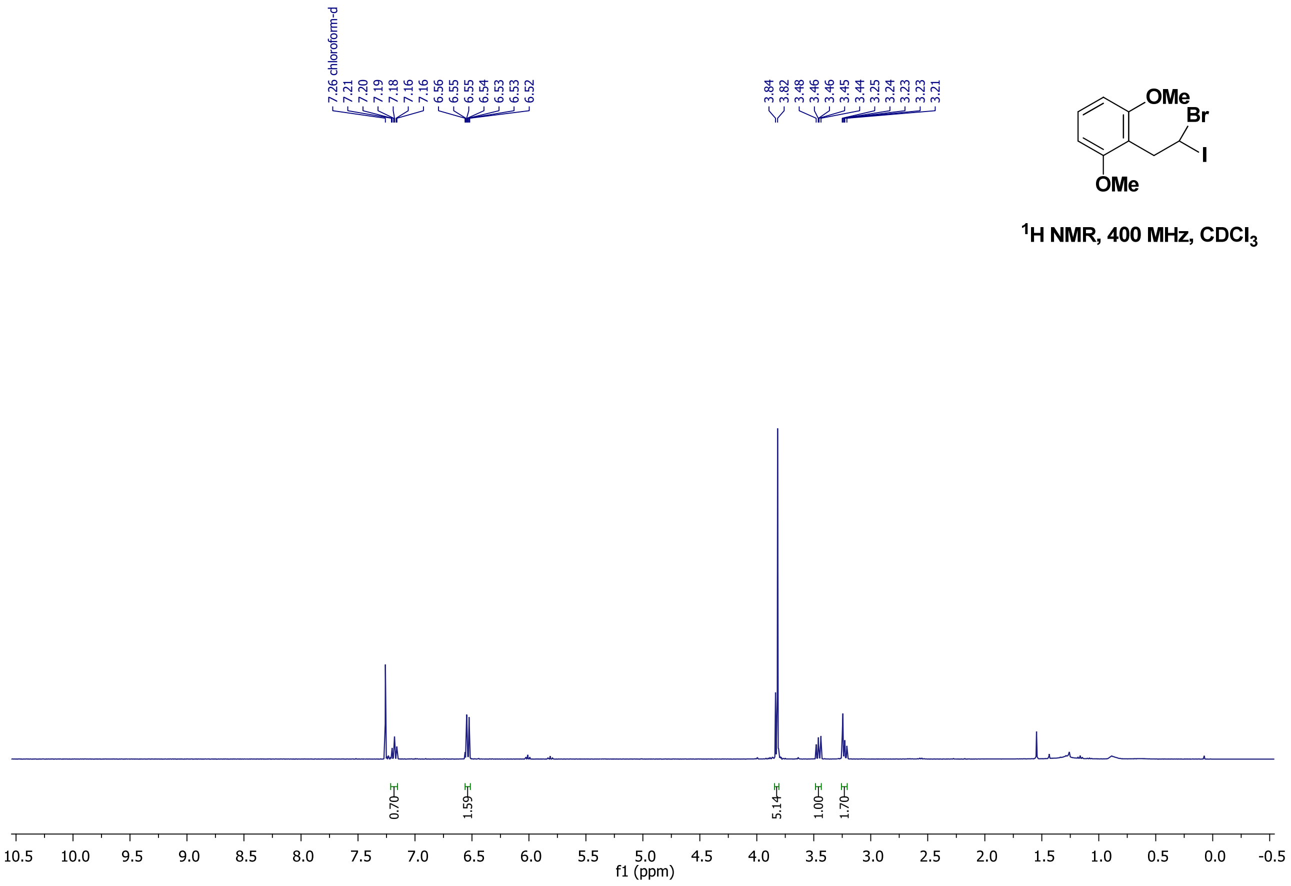

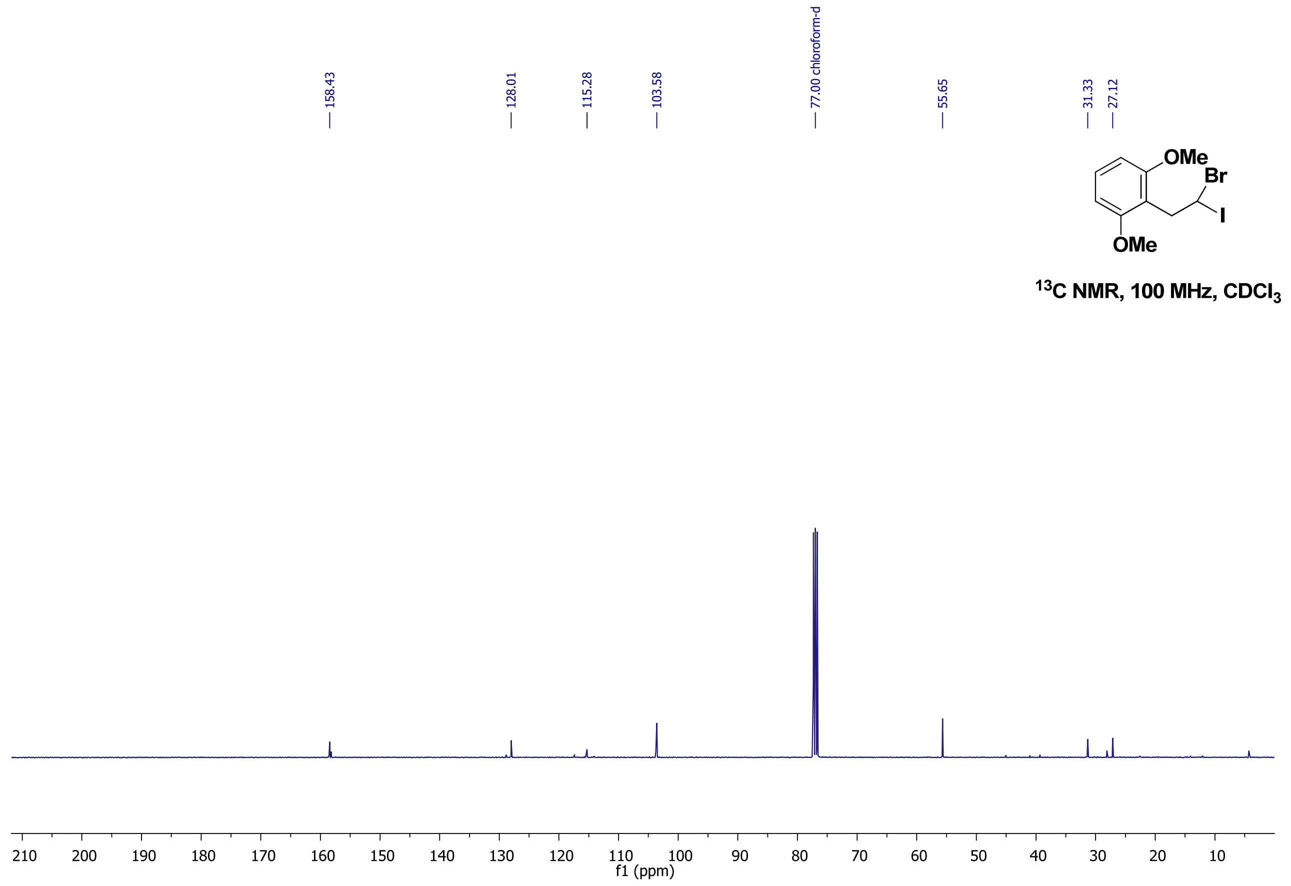

1,1'-(2,2-Difluoro-1,1-ethanediyl)dibenzene (81)

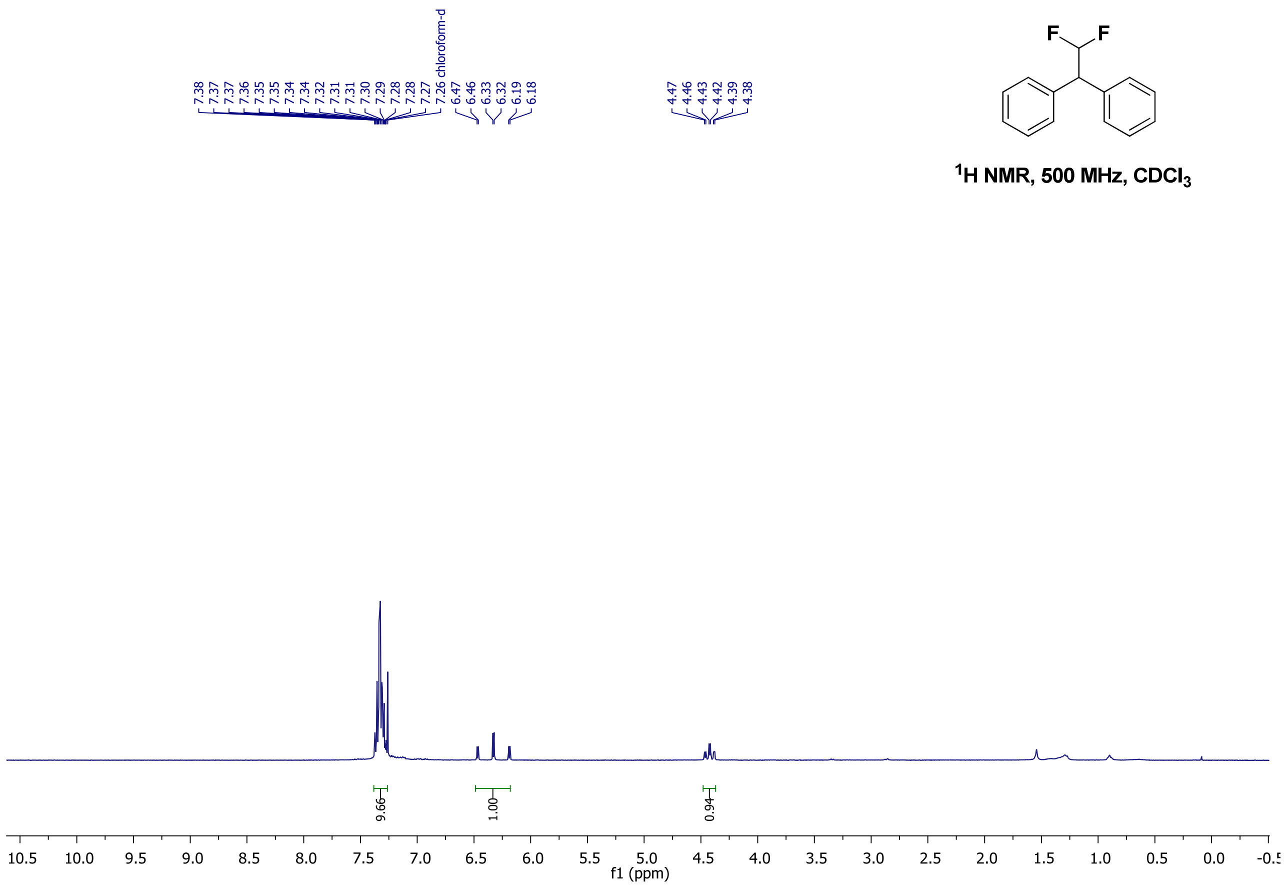

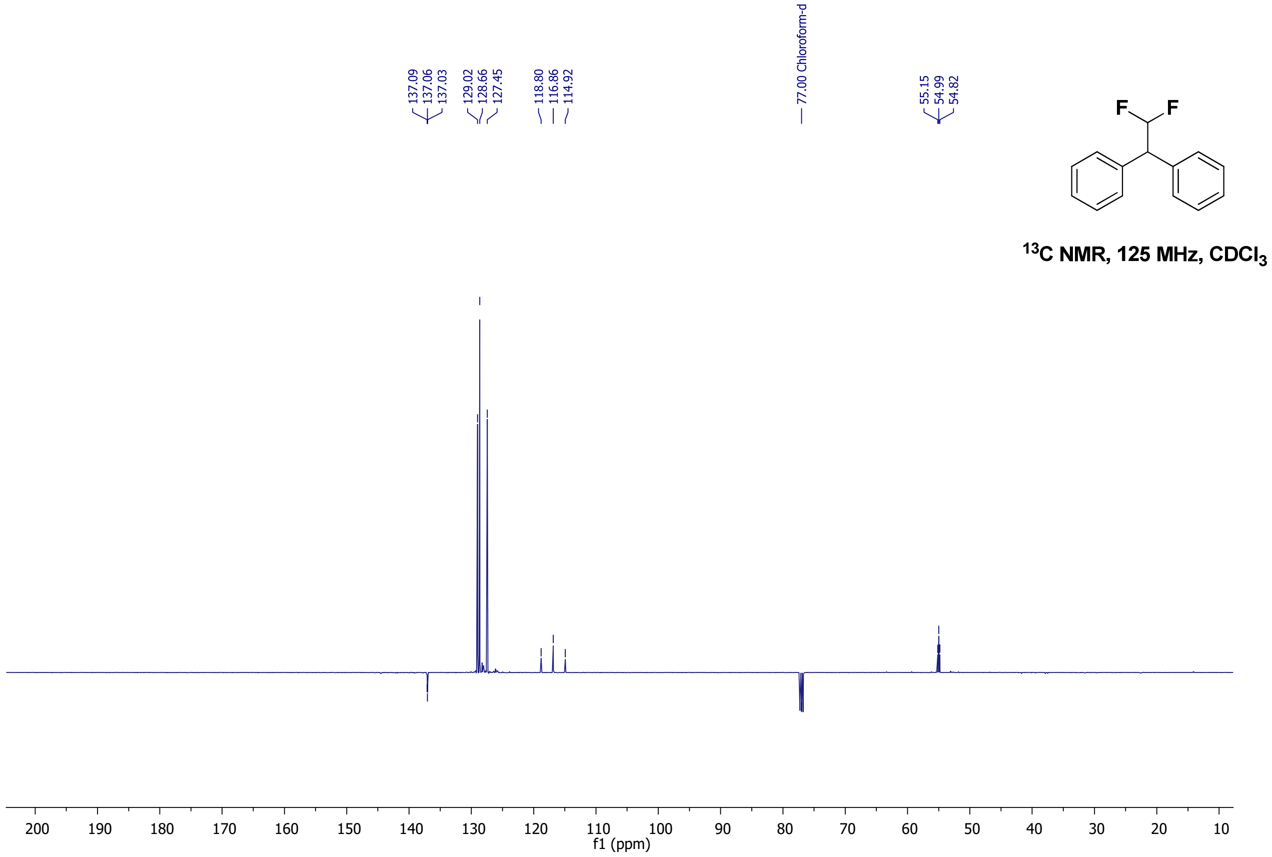

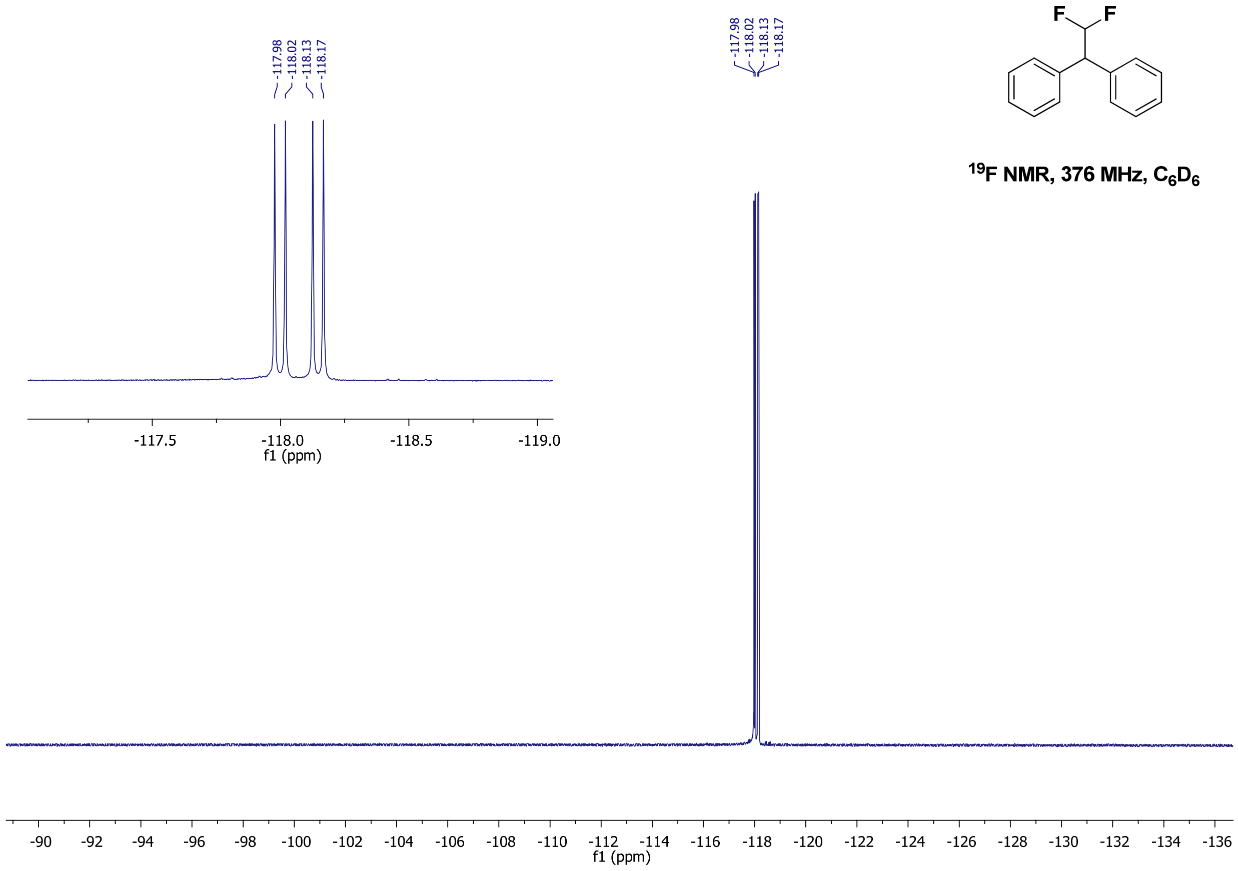

F
F
19F NMR, 376 MHz, C6D6
-117.98
-118.02
-118.13
-118.17
-117.5
-118.0
-118.5
-119.0
f1 (ppm)
-90
-92
-94
-96
-98
-100
-102
-104
-106
-108
-110
-112
-114
-116
-118
-120
-122
-124
-126
-128
-130
-132
-134
-136
f1 (ppm)

1,1'-(2,2,2-Trifluoro-1,1-ethanediyl)dibenzene (82)

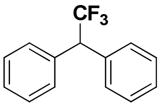

^{1}H NMR, 400 MHz, $CDCl_3$

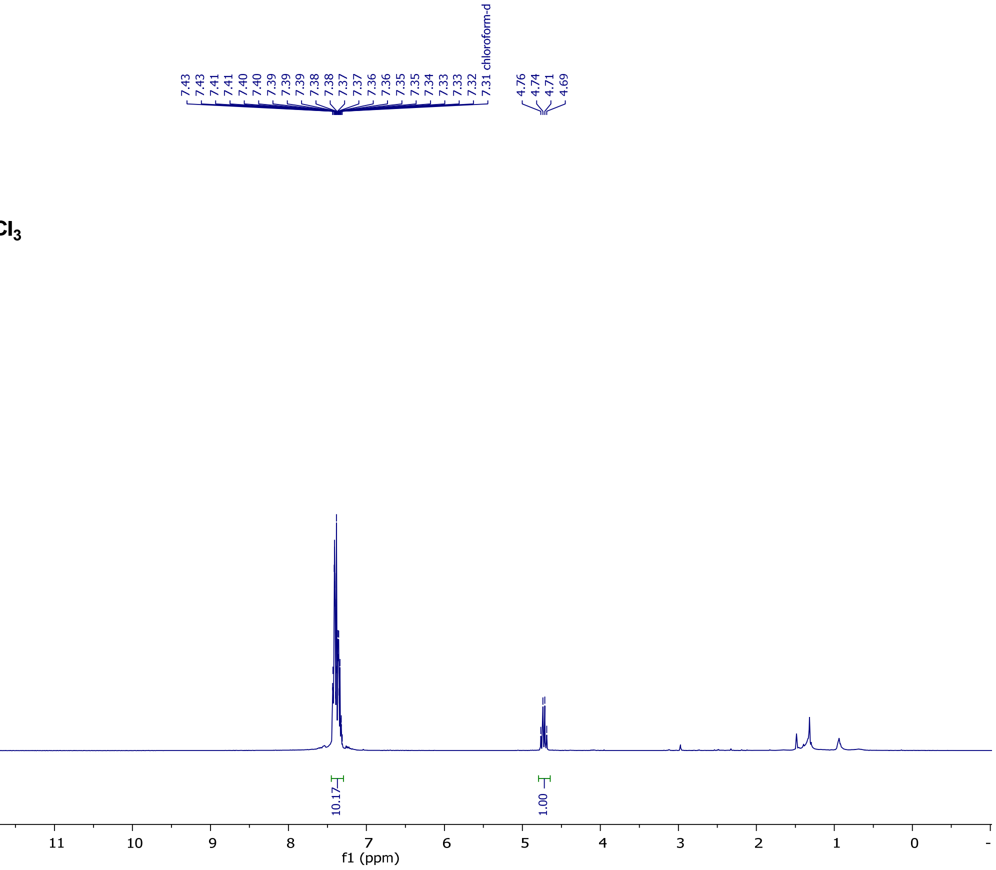

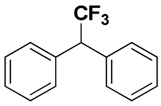

^{13}C NMR, 100 MHz, $CDCl_3$

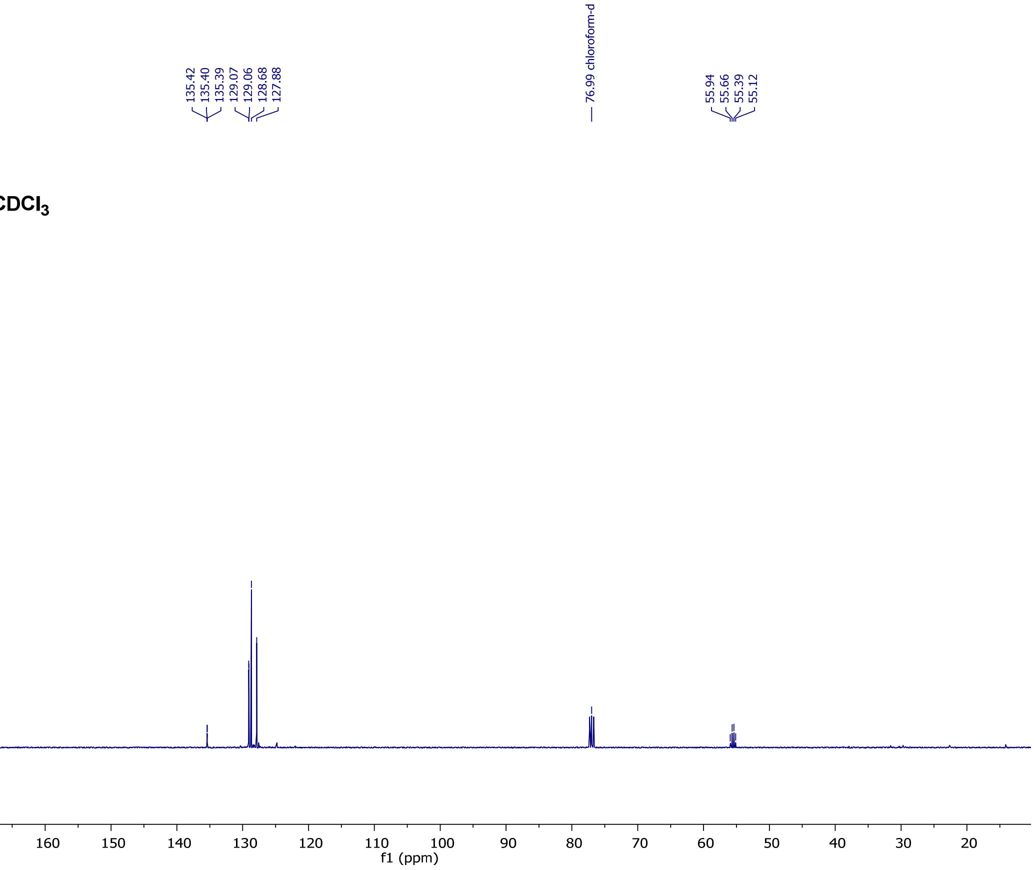

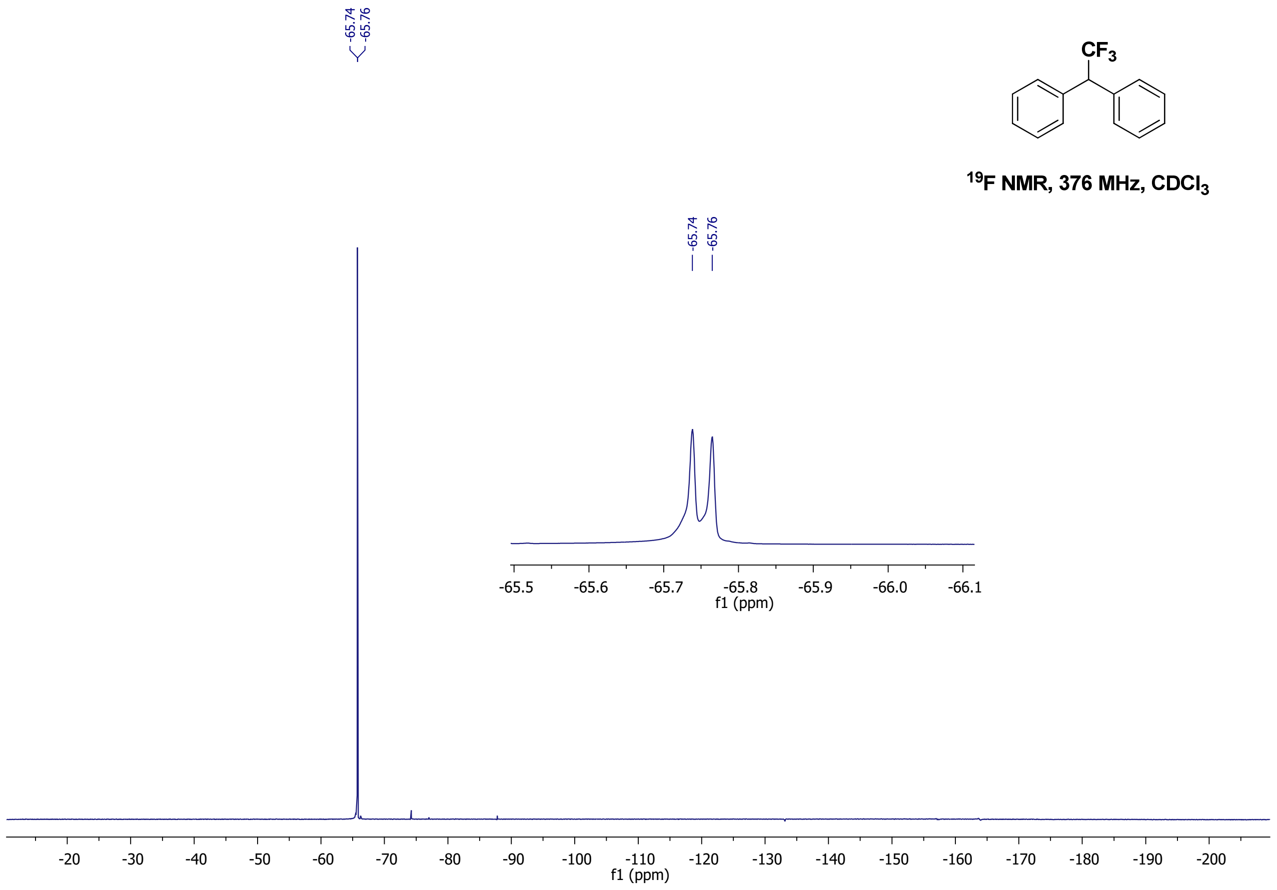

-65.74
-65.76
CF3
19F NMR, 376 MHz, CDCl3
-65.74
-65.76
-65.5
-65.6
-65.7
-65.8
-65.9
-66.0
-66.1
f1 (ppm)
-20
-30
-40
-50
-60
-70
-80
-90
-100
-110
-120
-130
-140
-150
-160
-170
-180
-190
-200
f1 (ppm)

[2-(4-Chlorophenyl)ethyl](trimethyl)silane (83)

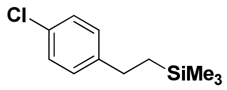

^{1}H NMR, 500 MHz, $CDCl_3$

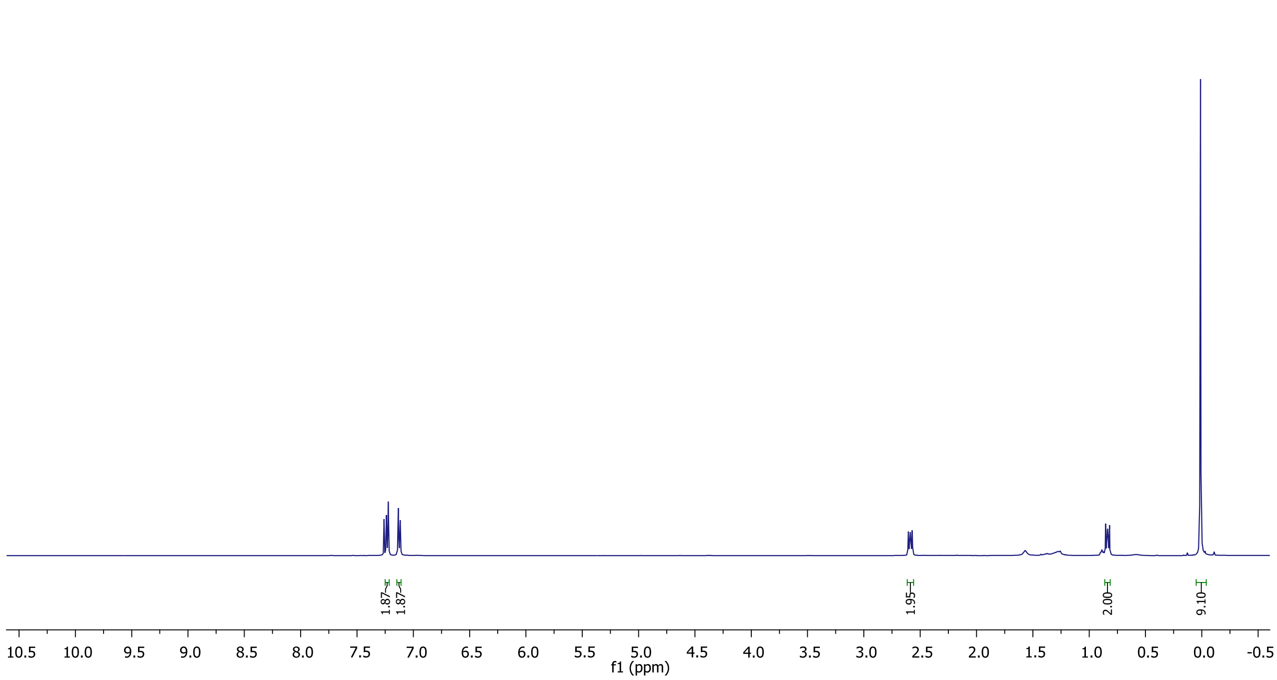

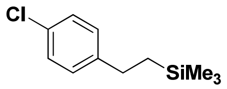

^{13}C NMR, 125 MHz, $CDCl_3$

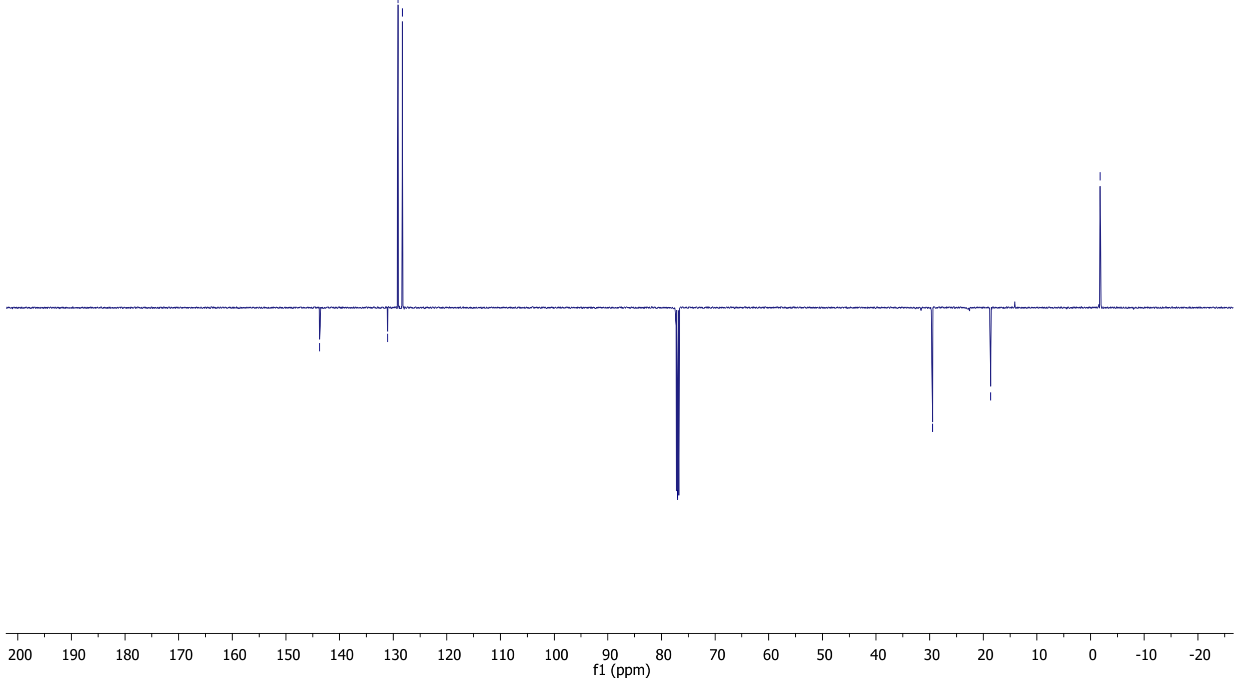

(2,2-diphenylethyl)trimethylsilane (84)

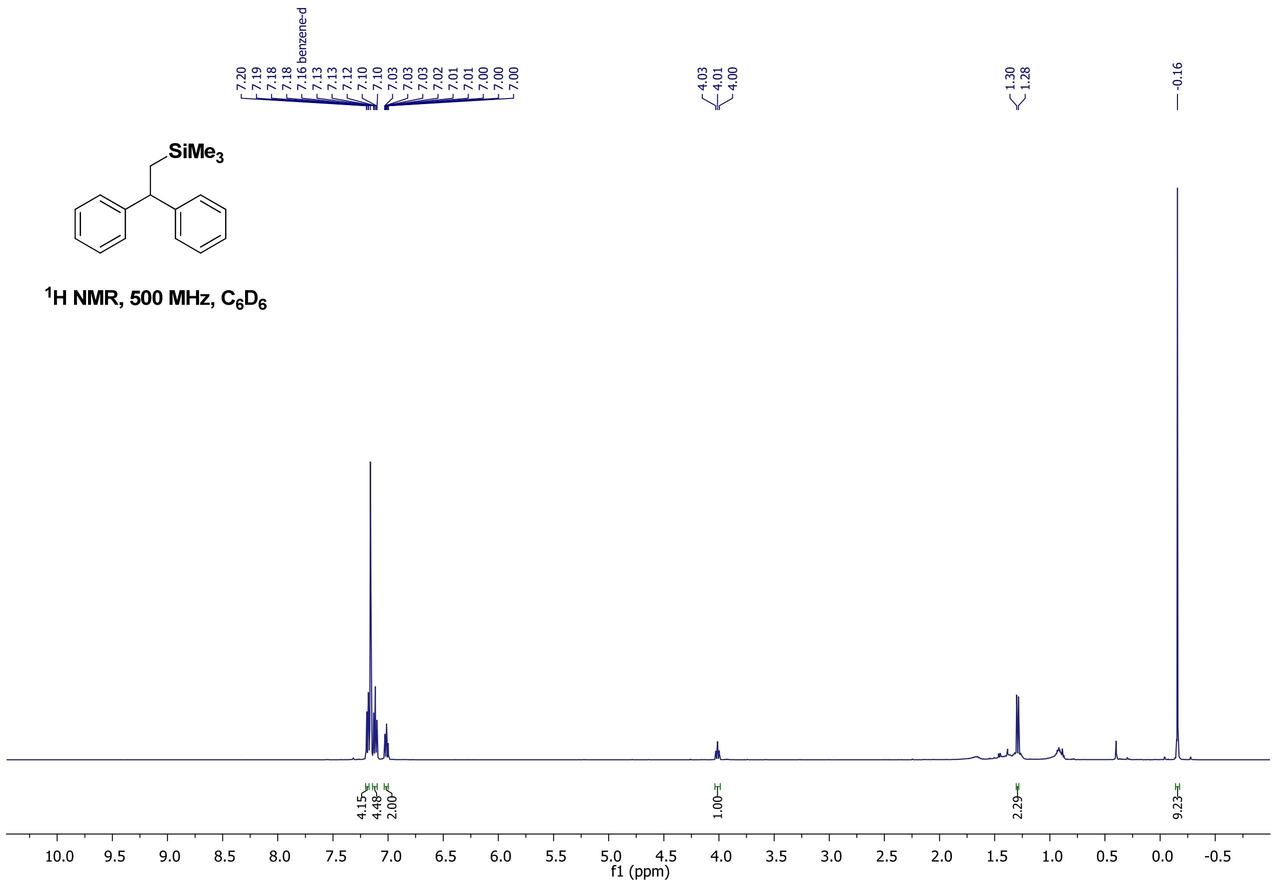

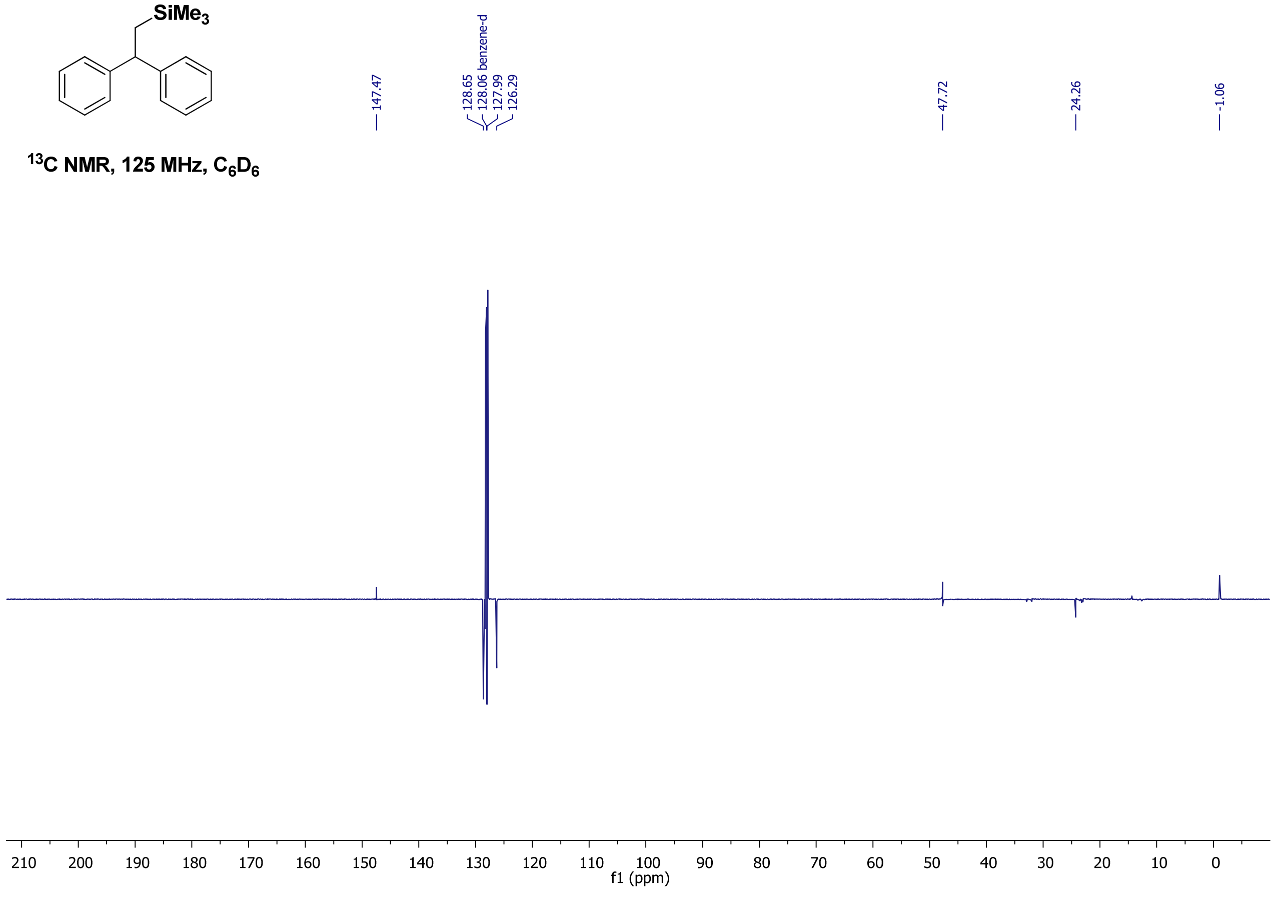

[(2-Phenylethyl)sulfanyl]benzene (85)

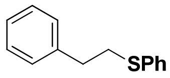

^{1}H NMR, 500 MHz, C_6D_6

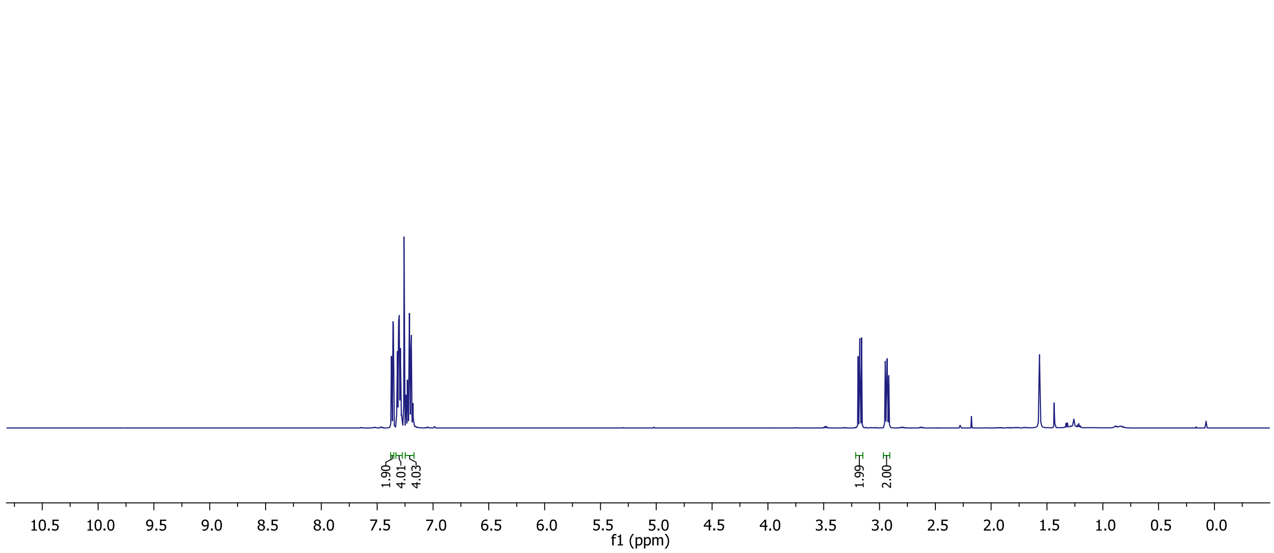

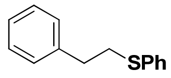

^{13}C NMR, 125 MHz, C_6D_6

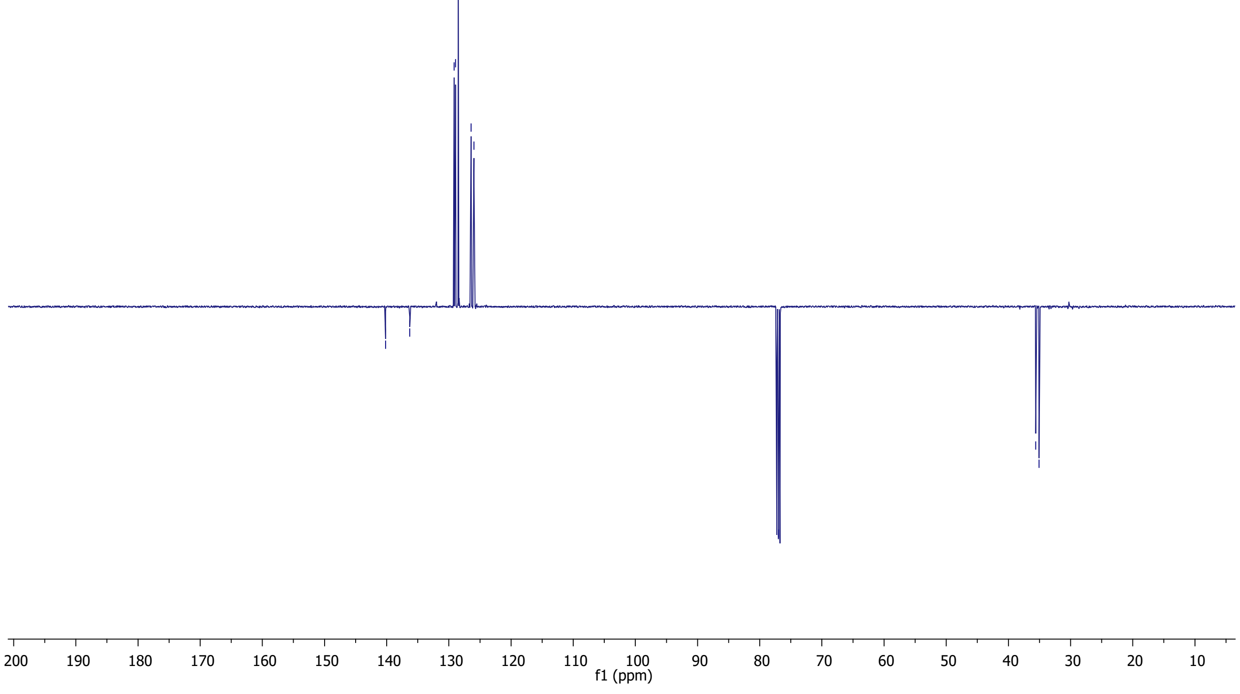

[(2-Phenylpropyl)sulfanyl]benzene (86)

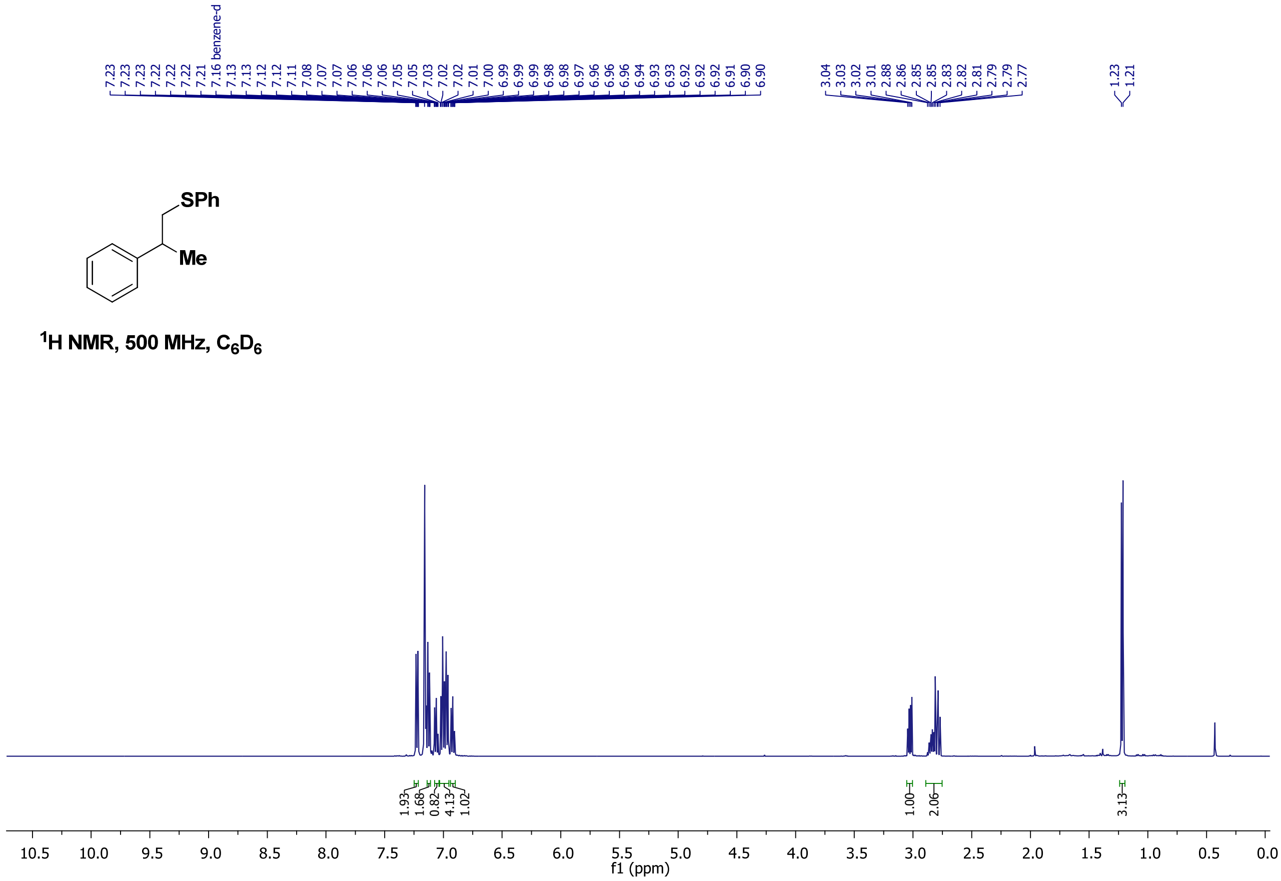

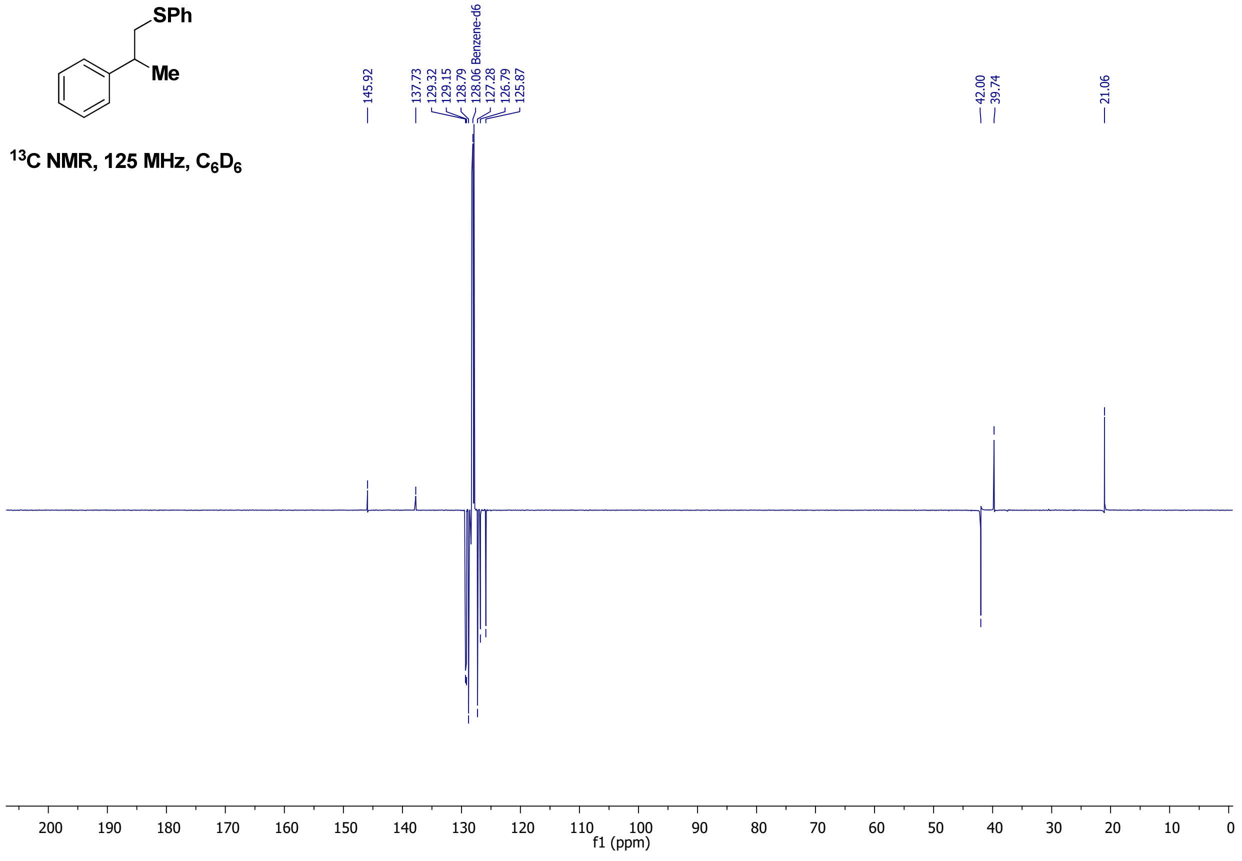

1,1'-[2-(Phenylsulfanyl)-1,1-ethane diyl]dibenzene (87)

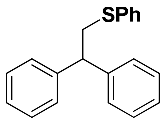

^{1}H NMR, 400 MHz, $CDCl_3$

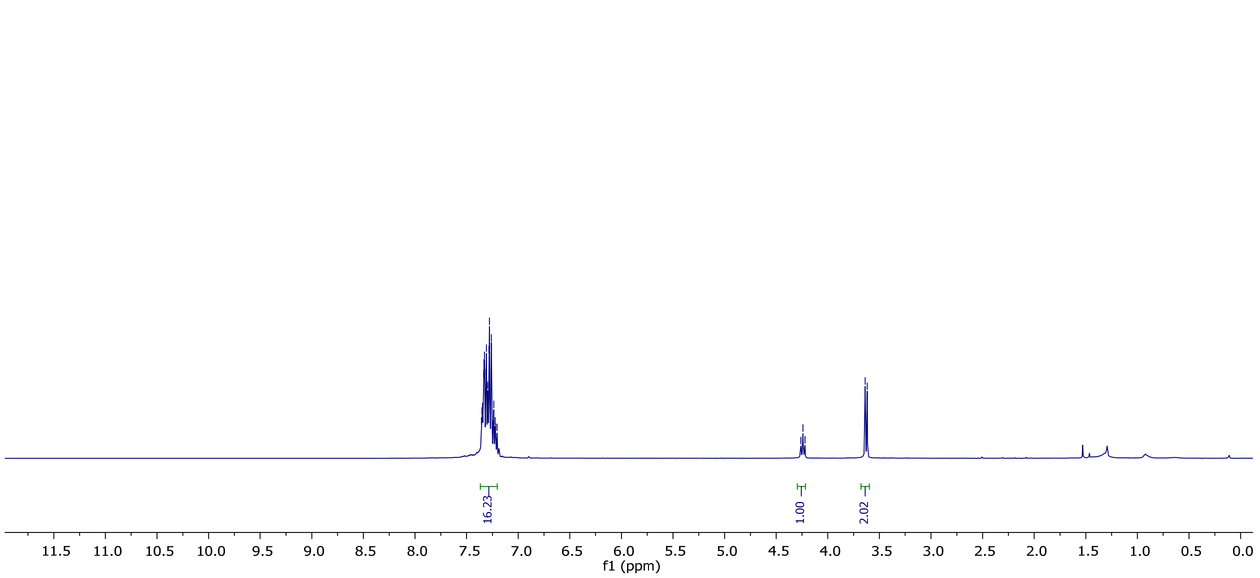

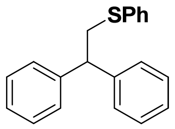

^{13}C NMR, 100 MHz, $CDCl_3$

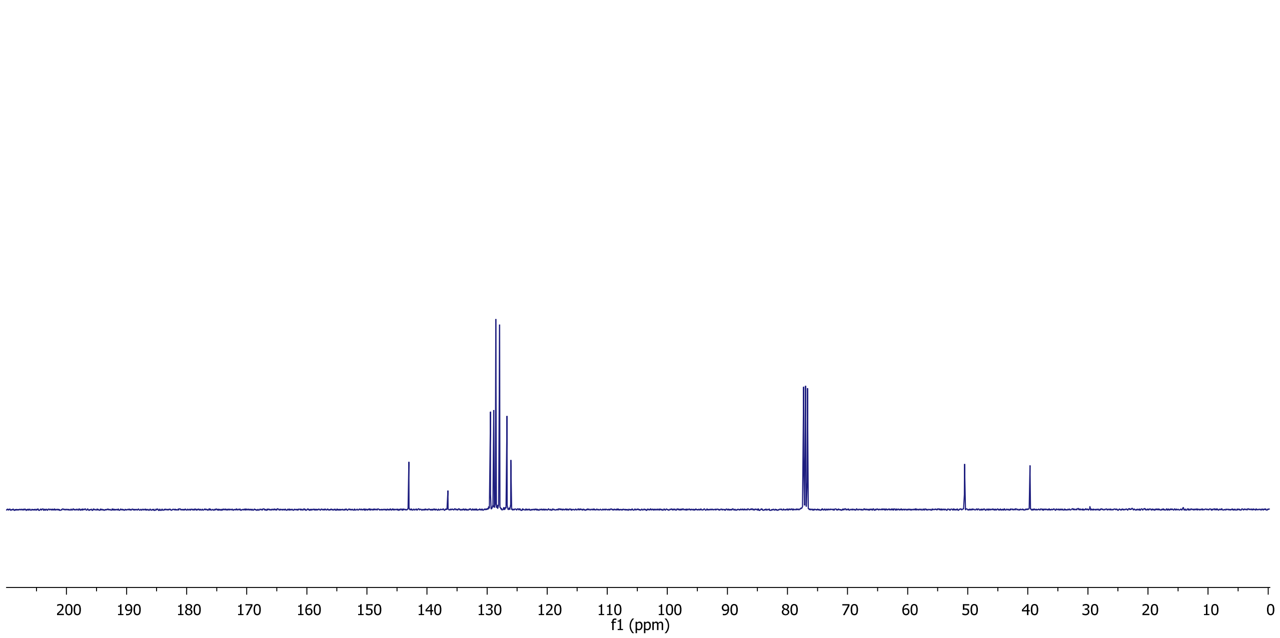

1,1'-(1,1-Ethanediyl)dibenzene (88)

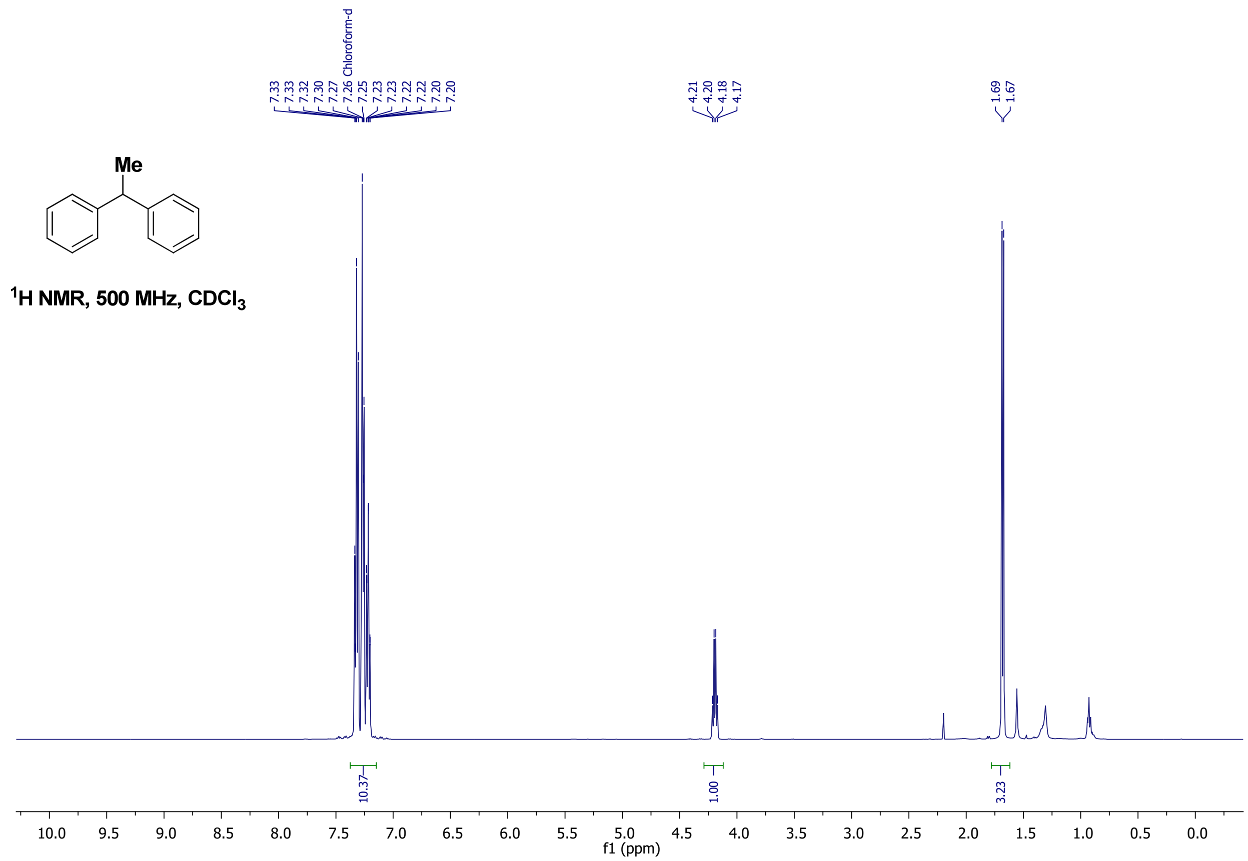

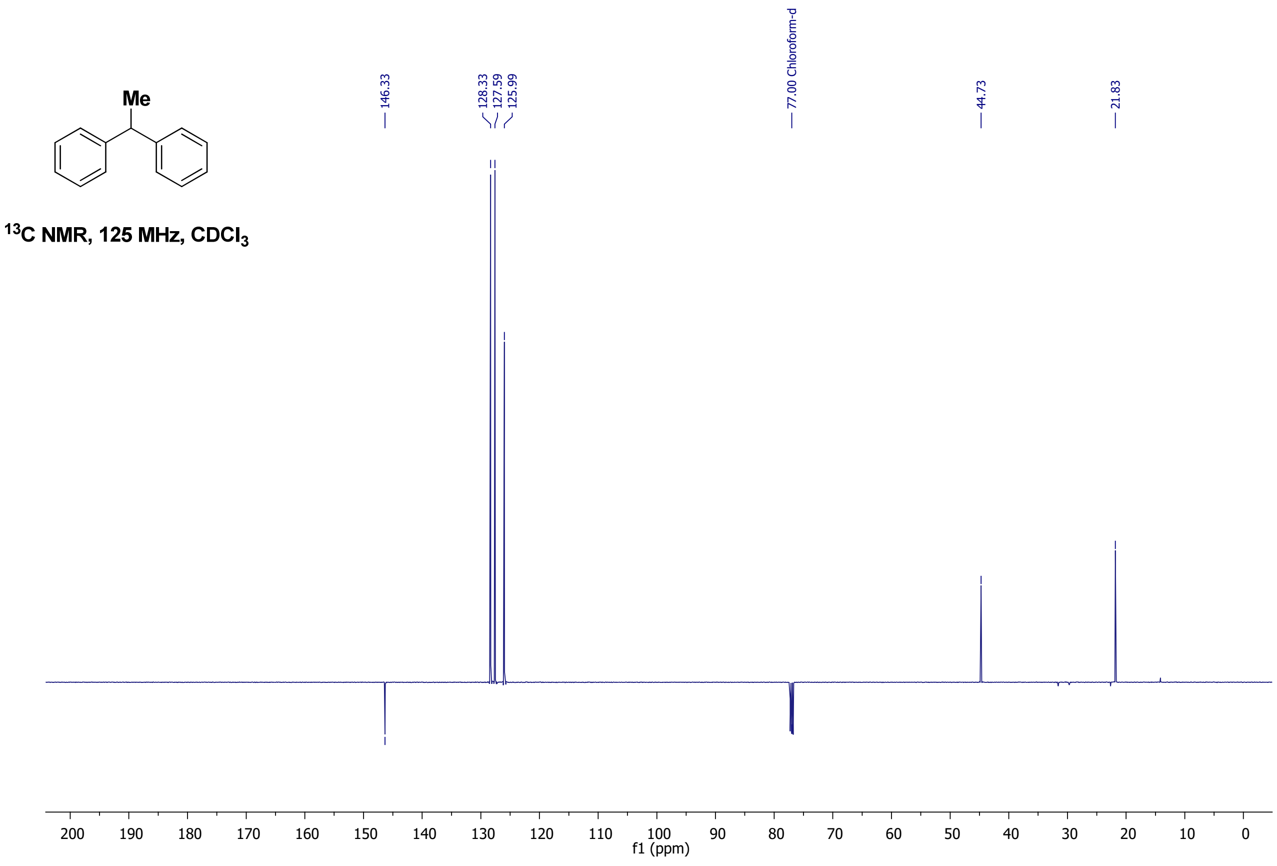

Triphenylmethane (89)

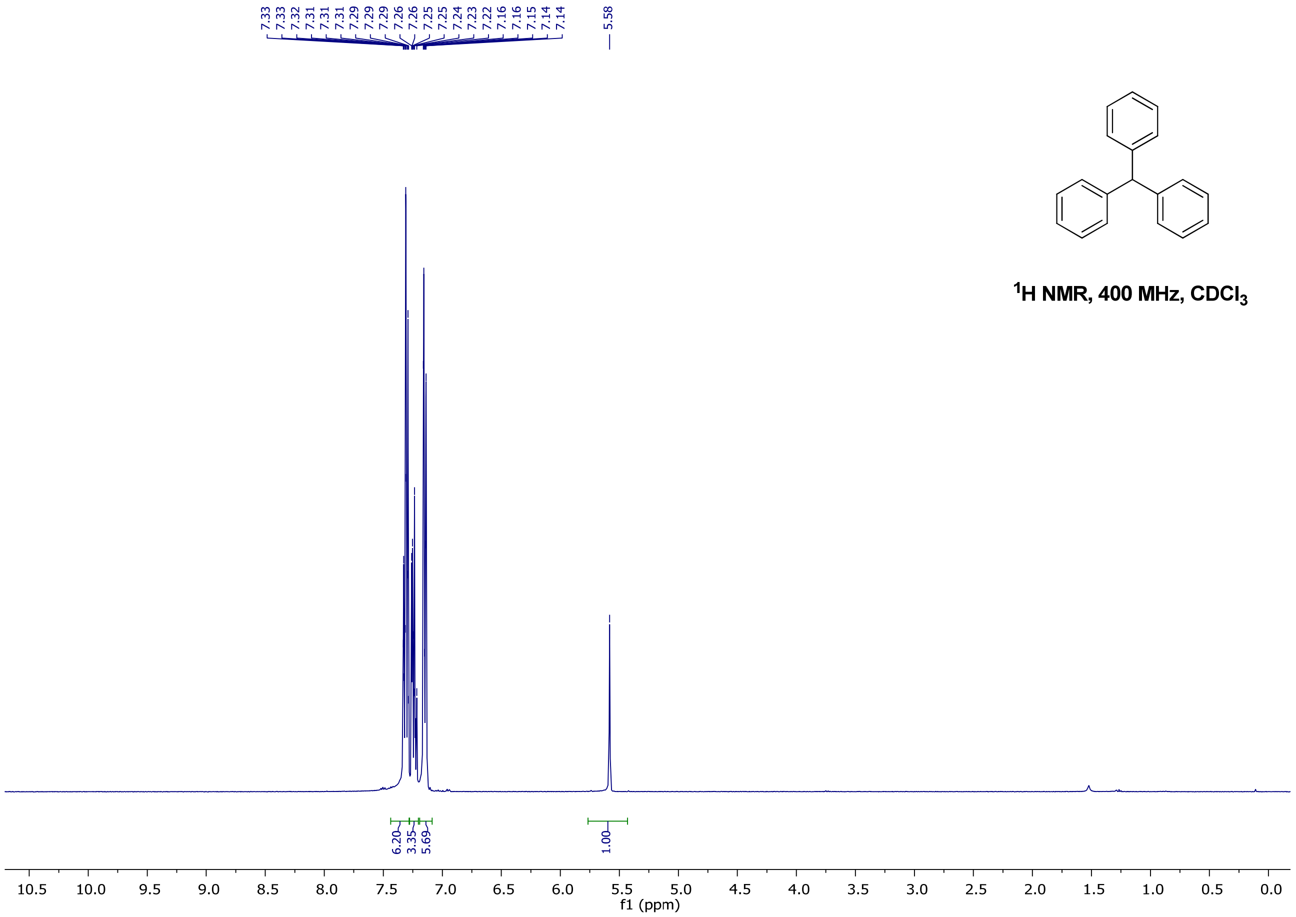

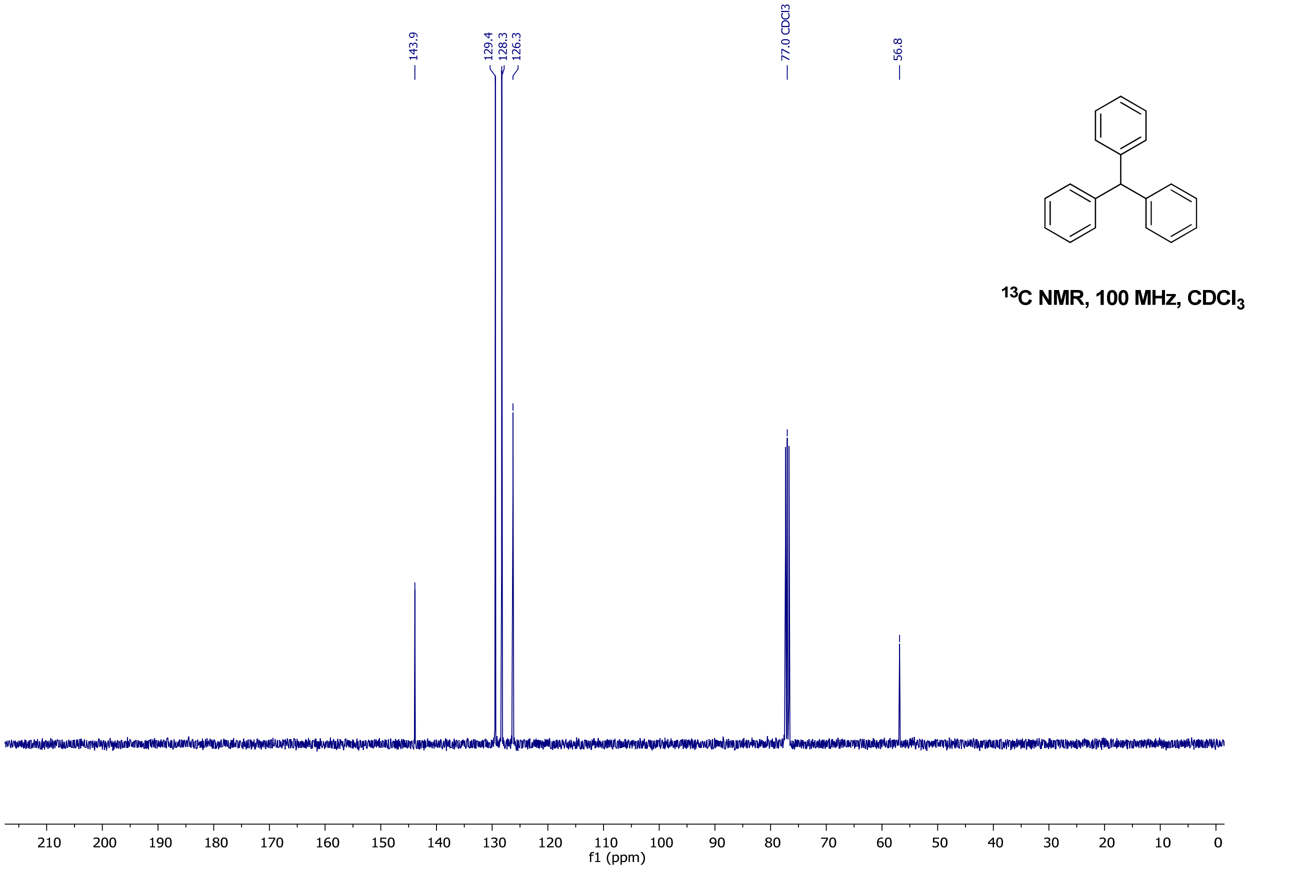

Supplement: Supplementary file 1 — ol0c02831_si_001.pdf [file ol0c02831_si_001.pdf]
